# Morphologic and molecular correlates of EZH2 as a predictor of platinum resistance in high-grade ovarian serous carcinoma

**DOI:** 10.1186/s12885-021-08413-3

**Published:** 2021-06-17

**Authors:** Brett M. Reid, Shraddha Vyas, Zhihua Chen, Ann Chen, Peter A. Kanetsky, Jennifer B. Permuth, Thomas A. Sellers, Ozlen Saglam

**Affiliations:** 1 Cancer Epidemiology, Moffitt Cancer Center, Tampa, FL USA; 2 Biostatistics and Bioinformatics, Moffitt Cancer Center, Tampa, FL USA; 3 Independent researcher, Tampa, USA; 4 Department of Pathology, Moffitt Cancer Center, 12902 USF Magnolia Dr, Tampa, FL 33612 USA

**Keywords:** High-grade ovarian serous carcinoma, EZH2, Chemotherapy response, Survival, TIL

## Abstract

**Background:**

Enhancer of zesta homologue 2 (EZH2) is an essential component of polycomb repressive complex 2 (PRC2) that contributes to tumor progression and chemo-resistance. The aim of this study was to comprehensively assess the prognostic value of EZH2 across the morphologic and molecular spectra of high-grade serous ovarian carcinoma (HGSOC) by utilizing both immunohistochemistry (IHC) and proteogenomic technologies.

**Methods:**

IHC of EZH2 was performed using a tissue microarray of 79 HGSOC scored (+/−) for lymphovascular invasion (LVI), tumor-infiltrating lymphocytic aggregates ≥1 mm (TIL) and architectural growth patterns. The association of EZH2 H-score with response to therapy and overall survival was evaluated by tumor features. We also evaluated EZH2 transcriptional (RNA sequencing) and protein (mass spectrometry) expression from bulk tumor samples from 336 HGSOC from The Cancer Genome Atlas (TCGA). EZH2 expression and co-expression networks were compared by clinical outcomes.

**Results:**

For HGSOC without TIL (58%), EZH2 expression was almost 2-fold higher in platinum resistant tumors (*P* = 0.01). Conversely, EZH2 was not associated with platinum resistance among TIL+ HGSOC (*P* = 0.41). EZH2 expression was associated with reduced survival for tumors with LVI (*P* = 0.04). Analysis of TCGA found higher *EZH2* expression in immunoreactive and proliferative tumors (*P* = 6.7 × 10^− 5^) although protein levels were similar across molecular subtypes (*P* = 0.52). Both mRNA and protein levels of EZH2 were lower in platinum resistant tumors although they were not associated with survival. Co-expression analysis revealed EZH2 networks totaling 1049 mRNA and 448 proteins that were exclusive to platinum sensitive or resistant tumors. The EZH2 network in resistant HGSOC included CARM1 which was positively correlated with EZH2 at both mRNA (*r =* 0.33, *p* = 0.003) and protein (*r =* 0.14, *P* = 0.01) levels. Further, EZH2 co-expression with CARM1 corresponded to a decreased prognostic significance of *EZH2* expression in resistant tumors.

**Conclusions:**

Our findings demonstrate that EZH2 expression varies based on its interactions with immunologic pathways and tumor microenvironment, impacting the prognostic interpretation. The association between high EZH2 expression and platinum resistance in TIL- HGSOC warrants further study of the implications for therapeutic strategies.

**Supplementary Information:**

The online version contains supplementary material available at 10.1186/s12885-021-08413-3.

## Background

Epithelial ovarian cancers (EOCs) make up the fourth leading cause of cancer related deaths for women between ages 40–59 [1]. Since the disease is typically diagnosed at advanced stages, survival remains poor, with little improvement since the introduction of platinum-based treatment almost 40 years ago [2]. High-grade serous ovarian carcinoma (HGSOC) is the most common (60%) histotype and also very aggressive, representing more than 70% of all EOC deaths [3]. Standard first-line treatment is surgical cytoreduction combined with platinum-based chemotherapy. Although several new and promising agents have been investigated in recent years, systemic or intraperitoneal platinum-based cytotoxic chemotherapy remains the standard initial approach to advanced and recurrent ovarian cancer. While the majority of HGSOC tumors are optimally debulked and display initial sensitivity to platinum-based regimens, most ultimately recur and become refractory to treatment [4].

Enhancer of Zesta Homologue 2 (EZH2) is the catalytic unit of Polycomb Repressive Complex 2 (PRC2) which functions as a highly conserved histone methyltransferase targeting H3K27 to induce target gene silencing [5]. EZH2 is involved in the regulation of cell cycle progression and its dysregulation results in accelerated cell proliferation and cancer development. Additionally, EZH2 contributes to tumor invasion and metastasis through the modulation of angiogenesis and the epithelial-to-mesenchymal transition [6]. Overexpression of EZH2 has been observed in a wide variety of cancers including ovarian cancer where its upregulation has been shown to occur with acquired cisplatin resistance in ovarian cancer cell lines and xenografts [7]. Conversely, knockdown of EZH2 re-sensitizes drug-resistant ovarian cancer cells to cisplatin [7].

EZH2 overexpression has been associated with poor survival in breast [8], prostate [9], and colorectal cancers [10], and studies for EOC have yielded similar results [11, 12]. EOC studies have found that EZH2 expression is upregulated with advancing stage and grade of disease, and among HGSOC with lymph node involvement [11, 13]. However, the prognostic significance of EZH2 has not been evaluated across the diverse morphologic and molecular features of HGSOC. Studies have shown tumor features such as tumor-infiltrating lymphocytes [14] and architecture pattern [15, 16] correspond with favorable prognosis and chemosensitivity. Furthermore, HGSOC have been differentiated into at least four molecular subtypes with differential patterns of gene expression [17, 18]. We therefore questioned whether associations between EZH2 expression and clinical outcomes varied by morphologic or molecular features of HGSOC. We performed an immunohistochemical analysis of EZH2 protein expression in a tissue microarray (TMA) of HGSOC with detailed morphological assessment. In addition, we used The Cancer Genome Atlas (TCGA) data to explore EZH2 mRNA and protein expression among molecular subtypes of HGSOC and the associations with prognosis. Finally, we performed co-expression analysis to identify differential co-expression networks underlying the prognostic relationship.

## Methods

### Study participants and clinical data

This study included data and specimens from women diagnosed with pathologically-confirmed HGSOC at Moffitt Cancer Center and Research Institute (Tampa, Florida). All women received cytoreductive surgery and subsequent first-line platinum-based chemotherapy and were followed for response to therapy (RTT). Participants provided written informed consent for research use of data and biospecimens through Total Cancer Care [19] protocol and usage for this study (#Pro00026247) was approved by the IRB of the University of South Florida. Demographic, clinical, and pathologic data were obtained from the Moffitt Cancer Registry (pathological diagnosis, degree of debulking/cytoreduction, histology, stage, grade), National Death Index (vital status), and medical record abstraction (chemotherapy regimens and response, BRCA status). Patient response to therapy was classified as platinum sensitive for complete responders with disappearance of all measurable disease, or in the absence of measurable lesions, a normalization of CA-125 level for 4 weeks, based on established guidelines [20]. Incomplete responders with partial response (defined as a 50% or greater reduction in tumor burden obtained from measurement of each bi-dimensional lesion for at least 4 weeks, or a drop in CA-125 by > 50% for at least 4 weeks), stable disease, or progression were considered platinum resistant.

### Tissue microarray and immunohistochemistry

We constructed a tissue microarray (TMA) of formalin-fixed paraffin embedded primary neoplastic tissue and unaffected benign ovarian tissues. Duplicate 1.0 mm cores were sampled to account for tissue heterogeneity. In total there were 96 HGSOC subjects, 29 of which had paired adjacent normal ovarian epithelium, non-neoplastic stroma, and/or fallopian epithelium sampled across five TMA slides. Slides were stained using a Ventana Discovery XT automated system (Ventana Medical Systems, Tucson, AZ) as per manufacturer’s protocol with proprietary reagents. Briefly, after deparaffinization (EZ Prep), heat-induced antigen retrieval was performed (standard CC1; Tris-EDTA buffer pH 7.8 at 95 °C for 44 min). Slides were incubated with rabbit anti-EZH2 (#790–4651) at predilute concentration followed by anti-rabbit antibody (OmniMap anti-Rt HRP, 760–4311). Slides were then immunostained with diaminobenzidine (ChromoMap DAB kit, 760–159) and counterstained with Hematoxylin. Negative control slides omitting the primary antibody were included in all assays.

EZH2 nuclear expression was manually reviewed and scored using histoscore (H-score; range 0–300) that was calculated by multiplying the percentage of positively stained cells by nuclear staining intensity (1+, 2+, 3+). Readings from multiple cores were averaged. IHC stained slides were also imaged with a Leica Aperio AT2 digital pathology scanner (Leica Biosystems, Vista, California) through a 20X/0.7NA objective lens. The percentage of positively stained nuclei was determined using Aperio’s Nuclear algorithm with default settings. Nuclei were binned by stain intensity (0, 1+, 2+, 3+) and digital H-score was calculated.

### Pathology review

Comprehensive pathologic review of all available H&E slides was performed to annotate morphological features. Histological subtypes other than HGSOC (e.g. mixed carcinomas and low-grade serous carcinomas) were excluded from further analysis (*n* = 17). For tumor grading, a previously described binary system was used [21]. Morphologic features were evaluated in the primary and metastatic anatomical sites with an average of 22 slides reviewed per case. Papillary architecture was defined as tumor cells arranged around finger-like vascular connective tissue. Micropapillary architecture displayed dyshesive tumor cell clusters lacking a central vascular core surrounded by retraction space. Solid architecture was defined as tumor cells arranged in sheets. Pseudoendometrioid/pseudoglandular architecture showed tumor growing in back-to-back nested pattern with punched out pseudo lumens.

### Statistical analysis

Primary statistical analyses of HGSOC EZH2 expression analyzed manual H-score continuous measurements. EZH2 scores were compared between groups using the Wilcoxon rank-sum test. The association of clinicopathologic and morphological characteristics and EZH2 expression with response to therapy (sensitive/resistant) were evaluated using logistic regression models. Associations with overall survival (hazard ratio, HR) were estimated using cox proportional hazards models. Adjusted models included significant clinicopathologic factors (*P* < 0.05) using forward stepwise selection with *P* < 0.10 as entrance criteria. Survival associations were additionally assessed for effect modification by entering interaction terms for EZH2 and morphological variables into the models, and stratified analyses were performed when significant (*P* < 0.05). As a sensitivity analysis, we secondarily performed all association analyses using digital EZH2 H-score to assess robustness of statistical findings to the method of EZH2 quantification. Statistical analyses were performed in SAS version 9.4 (SAS Institute Inc., Cary, NC, USA).

### Gene expression analysis in The Cancer Genome Atlas (TCGA)

We obtained molecular and clinical data for HGSOC samples from The Cancer Genome Atlas (TCGA). Clinical data were downloaded from the Genomic Data Commons data portal (https://portal.gdc.cancer.gov/) and molecular subtypes were annotated using classifications from the original TCGA mRNA expression analysis [17]. Level 1 (FASTQ) RNA sequencing data was downloaded using the gdc-client tool and aligned to the human reference genome build 37 (hs37d5) using tophat (version 2.0.13) and bowtie2, and gene-level reads was quantified based on GENCODE v25 gene model using HTSeq (version 0.6.1). The R package DESEQ2 was used to generate Fragments Per Kilobase of transcript per Million (FPKM) values from the raw expression counts. A total of 60,252 genes were quantified and analyses were limited to 336 HGSOC subjects with complete clinical data. Normalized relative protein expression was downloaded for 172 TCGA HGSOC subjects characterized by the Clinical Proteomic Tumor Analysis Consortium [22] and 8154 proteins quantified in at least 30% (*n* > 51) of subjects were included in the analysis. Tumor immune cytolytic activity metric was calculated as the geometric mean of *GZMA* and *PRF1* (mRNA) expression which correlates with tumor T cell activation and interferon-stimulated chemokines (CXCL9, CXCL10) [23].

We performed linear regression to test for differences in continuous (log2 transformed) *EZH2* expression by clinicopathologic factors, and logistic regression to estimate the association with RTT (resistant/sensitive). Cox proportional hazard modeling was used for survival analyses. All models evaluating EZH2 expression were adjusted for tumor purity using the consensus purity estimate [24]. Covariate adjustment for age, stage, residual disease, and molecular subtype were tested for significance (*P* < 0.10). Residual disease and subtype were included in RTT models while age and residual disease were included in survival models.

Co-expression analysis was limited to 31,238 genes with median expression >30th percentile. Gene expression correlations were calculated separately for mRNA and protein datasets using the partial correlation between EZH2 and each gene, controlling for tumor purity. Partial pearson correlations were calculated using log2 transformed mRNA expression levels. Protein expression levels were already normalized as previously described [22]. Differential co-expression analysis to statistically compare differences in EZH2 correlations by RTT and perform gene ontology enrichment analyses for differentially correlated genes was performed using the R package DGCA [25]. Briefly, DGCA computes and transforms correlation coefficients to z-scores and uses differences in z-scores to calculate empirical *p*-values of differential correlation between genes using permutation testing. We modified the DGCA pipeline to compare partial correlations by inputting residual expression values after the effect of tumor purity was regressed out. The DGCA wrapper function to perform gene ontology (GO) enrichment analysis is based on the GOstats R package (version 2.52) [26] and org. Hs.eg.db GO annotation R package (version 3.10.0). To visualize the differentially expressed EZH2 co-expression networks, we used the MEGENA R package (version 1.3.7) [27] to build a planar filtered network from differentially correlated gene pairs (*P* < 0.05) and identify hierarchal gene modules in the network using default parameters. We then constructed a graph of the gene expression correlation modules using Cytoscape (version 3.8.2) [28]. Statistical analyses were performed using R version 3.6.2 software (www.r-project.org).

## Results

### Clinicopathologic characteristics

Clinical and pathological characteristics of the 79 study participants with pathologically confirmed HGSOC samples are summarized in Table 1. The median age at diagnosis was 61 years and most (90%) were diagnosed at an advanced stage. Median survival was 4.1 years and the majority (75%) were platinum sensitive. Optimal debulking status (residual disease < 1 cm) was achieved during cytoreductive surgery for most patients and was similar between platinum sensitive and resistant cases (79% vs. 74%, respectively). Out of 21 cases with known BRCA germline mutation status, 11 were *BRCA1* or *BRCA2* mutation carriers. Carriers were significantly younger than non-carriers (49 years vs. 61 years, *P* = 0.01), but were otherwise similar across clinicopathologic characteristics and response to therapy (RTT) (see Table S1, Additional file 1).

**Table 1:** Clinicopathologic characteristics of study participants

	Overall^**a**^	Response to platinum-based therapy		
		Resistant	Sensitive	***P-***value^b^
**N**	79	19	59	
**Age at diagnosis, mean (SD)**	61.4 (11.1)	62.0 (12.4)	60.9 (10.6)	0.71
**Stage, n (%)**				
I,II	8 (10)	1 (5)	7 (12)	0.41
III,IV	71 (90)	18 (95)	52 (88)	
**Debulking status, n (%)**				
Suboptimal (Residual disease > 1 cm)	18 (24)	5 (26)	12 (21)	0.66
Optimal (NED or residual disease < 1 cm)	58 (76)	14 (74)	44 (79)	
**Lymphovascular Invasion, n (%)**				
Yes	62 (78)	15 (79)	46 (78)	0.93
No	17 (22)	4 (21)	13 (22)	
**Cystic Component, n (%)**				
Yes	54 (69)	13 (72)	40 (68)	0.72
No	24 (31)	5 (28)	19 (32)	
**STIC, n (%)**				
Yes	20 (28)	4 (22)	16 (31)	0.49
No	51 (72)	14 (78)	36 (69)	
**TIL, n (%)**				
Yes	32 (41)	10 (56)	22 (37)	0.17
No	46 (59)	8 (44)	37 (63)	
**Vital Status, n (%)**				
Alive	24 (31)	3 (16)	21 (36)	0.10
Died	54 (69)	16 (84)	38 (64)	
**Months of follow-up, mean (SD)**	60 (33)	36 (32)	58 (31)	0.008

*SD* standard deviation, *STIC* serous tubal intraepithelial carcinoma, *TIL* tumor infiltrating lymphocytes, *NED* no evidence of disease

^a^The total N for some variables does not total to 79 due to missing data

^b^Statistical significance testing was performed using the chi-square test for categorical variables and t-test for continuous variables

**Fig. 1:**
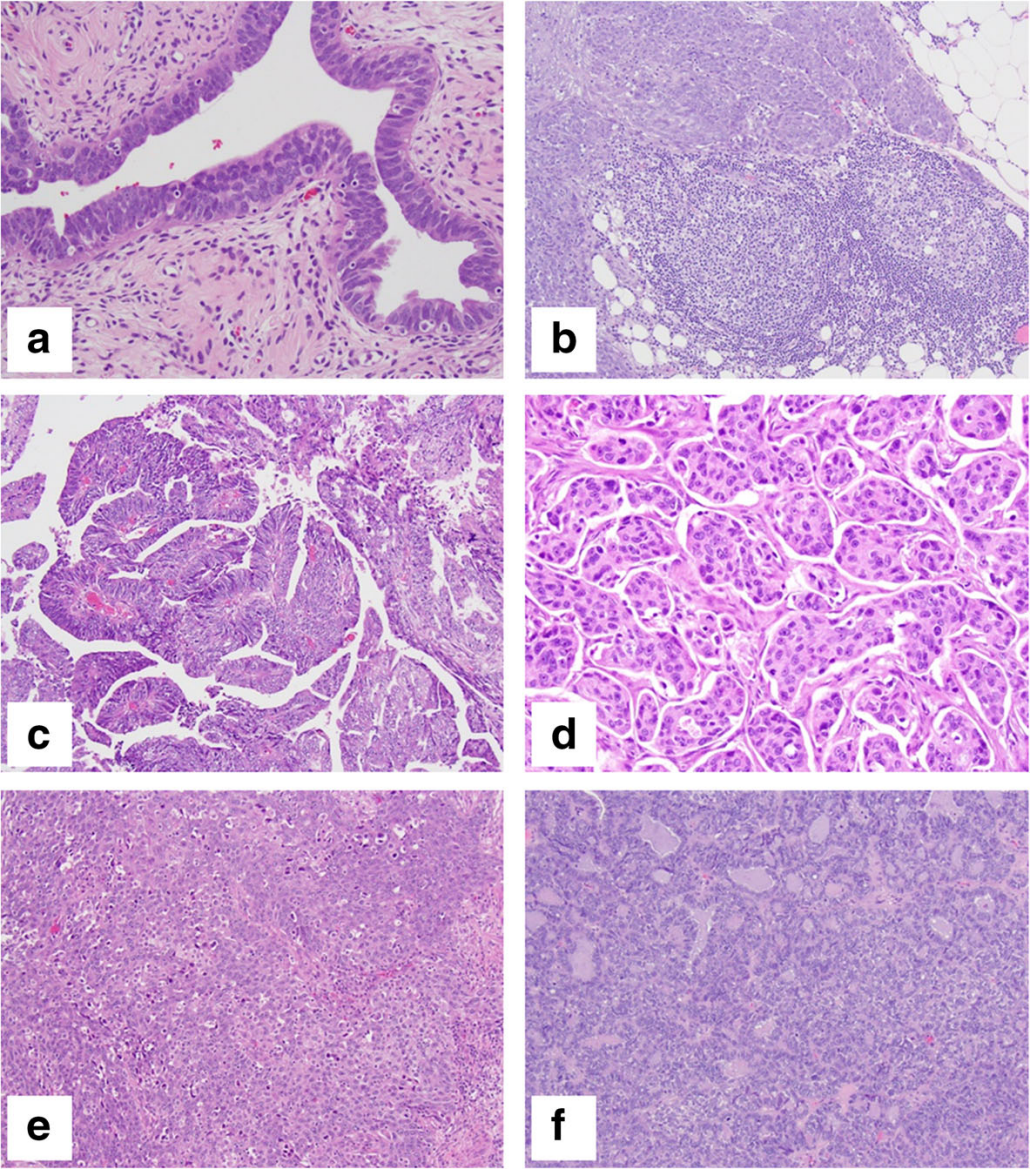
Representative slide images for (**a**) serous tubal intraepithelial carcinoma (STIC), (**b**) tumor-infiltrating lymphocytes (TIL), and tumor architectural growth patterns: (**c**) papillary, (**d**) micropapillary, (**e**) solid, and (**f**) pseudoglandular. Original magnification: 400x for **a**, **d**, **e** and 200x for **b**, **c**, **f**

Morphologic features assessed included presence of cystic component, lymphovascular invasion (LVI), serous tubal intraepithelial carcinoma (STIC; Fig. 1A), and intratumoral lymphoid aggregates within the peritumoral stroma measuring 1 mm or larger in size (tumor-infiltrating lymphocytes or TIL; Fig. 1B). Most tumors presented with LVI (79%) and cystic component (69%) features. LVI was less common among early stage cases than advanced stages (25% early vs. 85% advanced, *P* = 0.0009). TIL measuring 1 mm or larger were present in 41% of patient cases and did not vary by stage (*P* = 0.59). None of the pathologic features were associated with residual disease or RTT.

Tumor architectural growth patterns were characterized for all subjects which included papillary, solid, pseudoendometrioid/pseudoglandular, and micropapillary patterns (Fig. 1C-F). Patterns were reviewed across clinicopathologic factors (Table 2). The most predominant architectural patterns were papillary (76%) and pseudoglandular (76%) followed by solid (71%) and micropapillary (62%). Tumors often displayed multiple morphological features with most displaying three (50%) or all four patterns (22%) (see Table S2, Additional file 1). Micropapillary pattern was more common in subjects with advanced disease stage (*P* = 0.0002) and LVI (*P* < 0.0001). Papillary pattern was more common when a cystic component was present (*P* = 0.04). Subjects with STIC were more likely to have pseudoglandular pattern (*P* = 0.03). None of the architectural patterns or combination of patterns observed were associated with RTT.

**Table 2:** Tumor architectural growth patterns by clinicopathologic characteristics

	Overall	Micropapillary			Papillary			Pseudoglandular			Solid		
		No	Yes	P	No	Yes	P	No	Yes	P	No	Yes	P
N	79	30	49		19	60		19	60		23	56	
**Age at diagnosis, mean (SD)**	61.4 (11.1)	61.3 (9.1)	61.4 (12.2)	0.97	62.2 (7.4)	61.1 (12.1)	0.64	60.9 (11.3)	61.5 (11.1)	0.84	63.1 (13.0)	60.7 (10.3)	0.38
**Stage, n (%)**													
I,II	8 (10)	8 (27)	0 (0)	**0.0002**	2 (11)	6 (10)	0.33	2 (11)	6 (10)	0.33	2 (9)	6 (11)	0.32
III,IV	71 (90)	22 (73)	49 (100)		17 (89)	54 (90)		17 (89)	54 (90)		21 (91)	50 (89)	
**Debulking status, n (%)**													
Suboptimal	18 (24)	4 (14)	14 (30)	0.07	4 (21)	14 (25)	0.24	3 (17)	15 (26)	0.19	7 (32)	11 (20)	0.29
Optimal	58 (76)	25 (86)	33 (70)		15 (79)	43 (75)		15 (83)	43 (74)		15 (68)	43 (80)	
**Lymphovascular Invasion, n (%)**													
Yes	62 (79)	15 (50)	47 (96)	**<.0001**	16 (84.2)	46 (77)	0.21	14 (74)	48 (80)	0.20	17 (74)	45 (80)	0.53
No	17 (21)	15 (50)	2 (4)		3 (16)	14 (23)		5 (26)	12 (20)		6 (26)	11 (20)	
**Cystic Component, n (%)**													
Yes	54 (69)	20 (67)	34 (7)	0.70	9 (50)	45 (75)	**0.04**	14 (78)	40 (67)	0.16	16 (7)	38 (68)	0.68
No	24 (30)	10 (33)	14 (29)		9 (50)	15 (25)		4 (22)	20 (33)		6 (27)	18 (32)	
**STIC, n (%)**													
Yes	20 (25)	6 (23)	14 (31)	0.47	2 (12)	18 (33)	0.06	1 (7)	19 (34)	**0.03**	9 (43)	11 (22)	0.08
No	51 (65)	20 (77)	31 (69)		15 (88)	36 (67)		14 (93)	37 (66)		12 (57)	39 (78)	
**TIL, n (%)**													
Yes	32 (41)	13 (43)	19 (40)	0.74	10 (56)	22 (37)	0.15	6 (33)	26 (43)	0.45	7 (32)	25 (45)	0.30
No	46 (58)	17 (57)	29 (60)		8 (44)	38 (63)		12 (67)	34 (57)		15 (68)	31 (55)	
**Response to therapy**													
Sensitive	59 (76)	24 (83)	35 (71)	0.26	15 (79)	44 (75)	0.70	15 (79)	44 (75)	0.7	18 (78)	41 (75)	0.73
Resistant	19 (24)	5 (17)	14 (29)		4 (21)	15 (25)		4 (21)	15 (25)		5 (22)	14 (25)	
**Vital Status, n (%)**													
Alive	24 (30)	15 (50)	9 (18)	**0.003**	9 (47)	15 (25)	0.06	5 (26)	19 (32)	0.66	6 (26)	18 (32)	0.59
Dead	55 (70)	15 (50)	40 (82)		10 (53)	45 (75)		14 (74)	41 (68)		17 (74)	38 (68)	

*STIC* serous tubal intraepithelial carcinoma, *TIL* tumor-infiltrating lymphocytes, *SD* standard deviation

Clinical factors associated with overall survival included age at diagnosis (HR = 1.06, 95% CI = 1.03–1.09, *P* = 0.0001), advanced stage of disease (HR = 9.02, 95% CI = 1.24–65.44, *P =* 0.03), and platinum sensitivity (HR = 0.30, 95% CI = 0.16–0.58, *P* = 0.0003). After adjusting for these clinical factors, LVI and the papillary pattern were independently associated with poor survival (Table 3; HR = 3.36, *P* = 0.007 and HR = 2.39, *P* = 0.02, respectively). In stratified analysis, the association of papillary pattern with reduced survival was only observed in cases without a cystic component (P interaction = 0.02) in whom over a 5-fold higher hazard of death was observed when a papillary pattern was present (HR = 5.6, 95% CI = 1.5–20.4, *P* = 0.01). Further, the solid architectural pattern was associated with improved survival (HR = 0.24, 95% CI = 0.07–0.85, *P* = 0.03) but only in TIL- cases (P interaction = 0.004).

**Table 3:** Association of tumor morphological features with overall survival

	Unadjusted models		Adjusted Models^a^		Final Model^b^	
	HR (95%CI)	***p-***value	HR (95%CI)	***p***-value	HR (95%CI)	***p***-value
Pathological features						
STIC	1.38 (0.76–2.50)	0.29	1.35 (0.72–2.54)	0.35	–	–
TIL	0.92 (0.53–1.59)	0.76	0.84 (0.48–1.47)	0.54	–	–
Cystic component	0.95 (0.53–1.72)	0.88	1.10 (0.60–2.03)	0.76	–	–
LVI	**3.54 (1.51–8.31)**	**0.004**	**2.90 (1.21–6.97)**	**0.02**	**3.36 (1.40–8.07)**	**0.007**
Architectural pattern						
Micropapillary	**2.10 (1.15–3.82)**	**0.02**	1.61 (0.86–3.04)	0.14	–	–
Papillary	1.82 (0.91–3.62)	0.09	**2.01 (0.99–4.08)**	**0.05**	**2.39 (1.17–4.86)**	**0.02**
Pseudoglandular	0.97 (0.53–1.78)	0.92	0.87 (0.47–1.62)	0.67	–	–
Solid	0.88 (0.49–1.56)	0.66	0.89 (0.48–1.63)	0.70	–	–

*STIC* serous tubal intraepithelial carcinoma, *TIL* tumor-infiltrating lymphocytes, *LVI* lymphovascular invasion

^a^Models adjusted for clinical factors: age of diagnosis, stage (I/II vs. III/IV), and response to therapy (sensitive/resistant)

^b^Final model contains same  clinical factors as from adjusted model and all significant tumor features

### EZH2 expression and clinicopathologic features in HGSOC

**Fig. 2:**
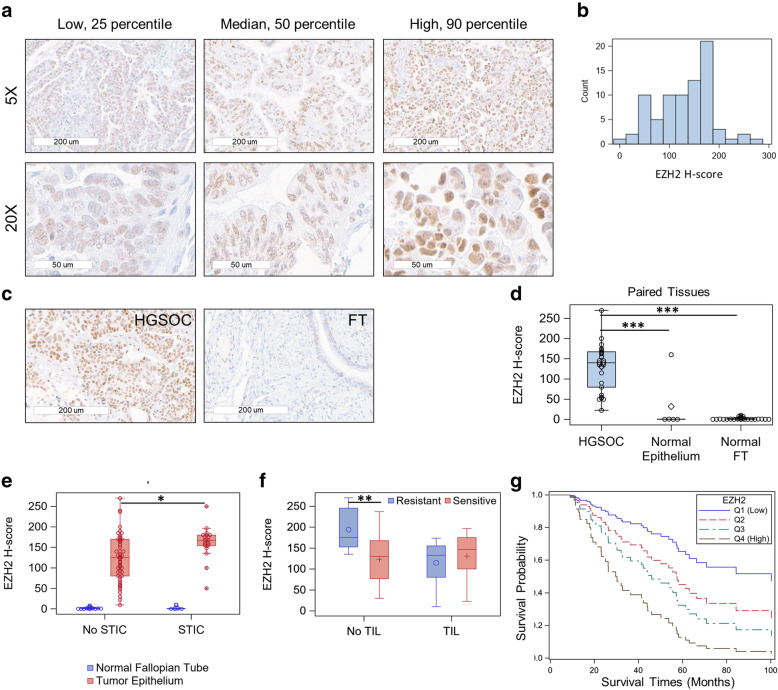
(**a**) Immunohistochemical (IHC) expression of EZH2 in low, median, and high expressing HGSOC samples at 5x and 20x magnification. (**b**) Distribution of EZH2 H-score in HGSOC tissues. (**c**) IHC expression of EZH2 in paired HGSOC and adjacent normal fallopian tube (FT) tissue in representative subject. (**d**) Boxplots of EZH2 H-score in HGSOC (*n* = 27) and paired normal epithelium (*n =* 5) and normal fallopian tube (FT, *n* = 24) tissues. Groups were compared using the paired t-test. (**e**) Boxplots of EZH2 H-score in HGSOC and normal appearing FT tissues for subjects with STIC (*n =* 20) and without STIC (*n =* 51). Groups were compared using the Wilcoxon two-sample test. (**f**) Boxplots of HGSOC EZH2 H-score grouped by response to therapy (sensitive/resistant) and presence/absence of TIL. The symbols in the boxplots indicate the mean for (o) resistant and (+) sensitive subgroups. Horizontal line in box interior represents the group median. Groups were compared using the Wilcoxon two-sample test. (**g**) Adjusted survival curve estimates for EZH2 expression for the lowest quartile (Q1) to the highest quartile (Q4) using cox proportional hazards regression. Adjustments include age, stage, and response to therapy. Statistical significance of boxplot comparisons is indicated as follows: *p < 0.05, **p < 0.01, ***p < 0.0001

EZH2 expression was evaluated in tumor epithelium of all subjects included in the study. Representative immunohistochemical staining of EZH2 is shown in Fig. 2A. Median H-score of EZH2 was 140 with a range of 10 to 270 in neoplastic tissue (Fig. 2B). For 24 cases with paired normal fallopian tube tissue, we observed significant overexpression of EZH2 within the neoplastic tissue (*P* = 4.4 × 10^− 10^; Fig. 2C and D). Five cases demonstrated similar overexpression of EZH2 in HGSOC compared to paired normal ovarian epithelium (*P* = 0.04, Fig. 2D). EZH2 expression was absent from normal stroma samples (*n* = 40) and was only quantified digitally.

Tumor EZH2 expression was slightly higher for cases with suboptimal debulking status (*P* = 0.06) and platinum resistance (*P* = 0.09), but it was not associated with disease stage (*P* = 0.92) or age of diagnosis (*P* = 0.23) (Fig. S1, Additional file 1). Overexpression of EZH2 was observed in HGSOC tissue from cases that were confirmed to have STIC present (*n* = 20) compared to HGSOC from cases without STIC (*n* = 51, *P* = 0.01) whereas expression did not differ in normal appearing fallopian tube tissue obtained from HGSOC cases with STIC (*n =* 5) compared to those without STIC (*n* = 14, *P* = 0.37, Fig. 2E). EZH2 was not differentially expressed based on other morphological characteristics or BRCA mutation status (*P* = 0.76).

### EZH2 expression and HGSOC prognosis

Among tumors without TIL, EZH2 upregulation was associated with platinum resistance (OR = 1.03, 95% CI = 1.01–1.05, *P =* 0.01; Fig. 2F); however, no association (OR = 0.99, 95% CI = 0.98–1.01, *P* = 0.41) was observed for tumors with TIL present (P interaction = 0.01). EZH2 was not significantly associated with RTT for any other morphologic subgroup. Expression of EZH2 was not associated with overall survival among all HGSOC (*P* = 0.79). When stratified by morphological characteristics, increasing EZH2 expression was nominally associated with reduced survival (HR = 1.00, 95% CI = 1.00–1.02, *P* = 0.04) among 62 cases with LVI present (P interaction = 0.02). For HGSOC with LVI, mean survival was 32 months in the top quartile of EZH2 expression compared to 42 months in the bottom quartile (Fig. 2G). This effect was slightly more pronounced in the majority (76%) of LVI cases with micropapillary pattern (HR = 1.01, 95% CI = 1.00–1.02, *P* = 0.02). For HGSOC without LVI, there was no association observed between EZH2 expression and overall survival (HR = 0.99, 95% CI = 0.98 to 1.00, *P* = 0.10).

Digitally quantified EZH2 H-scores ranged from 3 to 126 (median = 28) and were highly correlated to manually obtained H-scores (spearman *r* = 0.80, *P* < 0.001). Secondary analyses using digital H-score replicated our primary results for EZH2 upregulation in HGSOC tissues compared to paired fallopian tube (*P* = 2.8 × 10^− 5^). Digital quantification of EZH2 in normal stroma confirmed expression was largely not detected (Fig. S2, Additional file 1). Higher EZH2 expression was observed in patients with suboptimal debulking (*P* = 0.02), but differential expression was not observed for response to therapy (*P* = 0.50) or presence of STIC (*P* = 0.14). By morphological features, we observed that higher EZH2 expression in HGSOC without TIL was associated with platinum resistance (OR = 1.04, 95% CI = 1.01–1.07, *P =* 0.02) using digital H-score. EZH2 expression was associated with reduced survival (HR = 1.01, 95% CI = 1.00–1.03, *P* = 0.03) for HGSOC with LVI, but micropapillary pattern was not significant (*P* = 0.06).

### EZH2 mRNA and protein expression among HGSOC molecular subtypes in TCGA

**Fig. 3:**
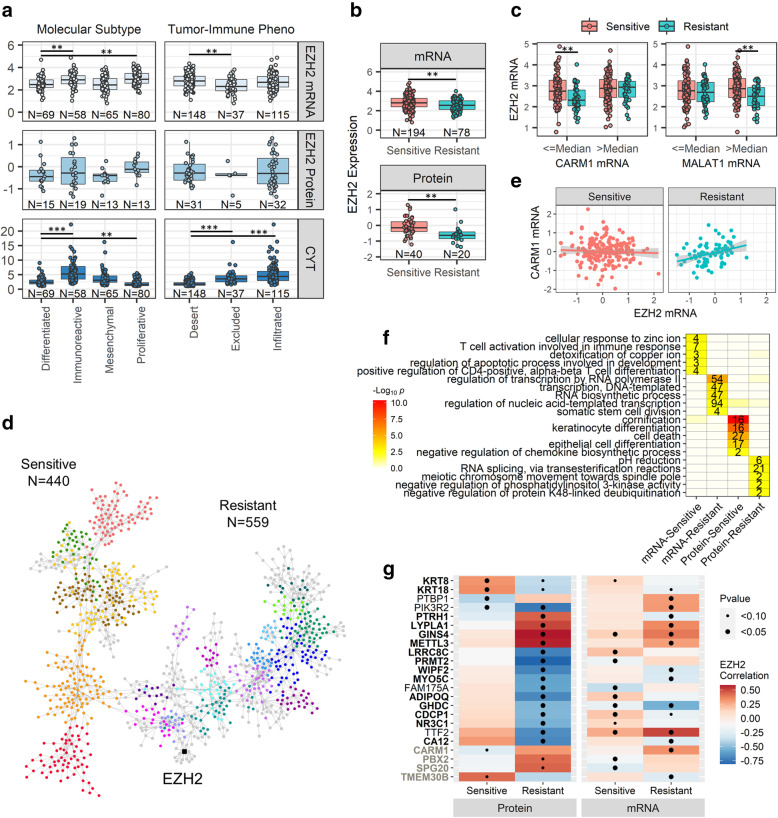
Evaluation of EZH2 expression in HGSOC TCGA samples. (**a**) Boxplots of expression levels for EZH2 mRNA, EZH2 protein and cytolytic activity (CYT) metric across the four molecular subtypes of HGSOC. Expression levels/scores were compared across groups with linear regression models adjusting for tumor purity. (**b**) Boxplots of EZH2 expression by response to therapy. Unadjusted group comparisons using Student’s t-test are represented. (**c**) *EZH2* mRNA expression in platinum sensitive and platinum resistant tumors, stratified by expression of top modifier genes *CARM1* and *MALAT1*. Unadjusted group comparisons using Student’s t-test are represented. (**d**) Planar filtered network of genes differentially correlated (*p* < 0.05, *n* = 1490) with EZH2 in platinum resistant compared to platinum sensitive HGSOC. Modules were identified at *P <* 0.05 in this network. Two distinct parent modules represent the distinct sensitive (*n* = 440) and resistant (*n* = 559) co-expression networks. Modules are identified with distinct colors for visualization. (**e**) Differential correlation between *EZH2* and *CARM1* in platinum resistant tumors. (**f**) GO enrichment for genes correlated with EZH2 in platinum sensitive network versus the resistant EZH2 network. mRNA and protein analyses are labeled separately. The number of genes represented in each GO Biological Process are annotated. (**g**) Differential correlation between EZH2 and genes that were identified in both mRNA and protein expression analysis. Statistical significance of boxplot comparisons is indicated as follows *p < 0.05, **p < 0.01, ***p < 0.0001

To understand the dynamics of EZH2, we explored transcriptome and proteomic co-expression networks across the clinicopathologic and molecular features of 336 clinically characterized HGSOC in TCGA. EZH2 transcript levels were correlated with protein levels in 72 cases with both mRNA and protein measurements (*r =* 0.29, *P* = 0.01). *EZH2* mRNA expression differed across the four molecular subtypes of HGSOC [17] exhibiting higher expression in immunoreactive and proliferative tumors (*P* = 6.7 × 10^− 5^); however, EZH2 protein levels did not differ (*P* = 0.52, Fig. 3A). Given the importance of TIL in our IHC analysis of EZH2, we evaluated EZH2 mRNA expression by cytolytic activity score, which correlates with T cell infiltration [29]. Cytolytic activity varied by molecular subtype (*P* = 2.2 × 10^− 16^) with immunoreactive tumors showing the highest cytolytic activity metric followed by mesenchymal, differentiated, and proliferative (Fig. 3A). EZH2 mRNA and protein levels did not correlate with cytolytic activity metric (mRNA *r =* 0.08, *P* = 0.15; protein *r =* − 0.16, *P* = 0.17). We also assessed EZH2 expression by molecularly characterized tumor-immune phenotypes. Tumors were classified as immune deserts (CD8+ T cells are either absent or present in very low numbers), immune excluded (infiltrating CD8+ T cells accumulate in the tumor stroma not the tumor epithelium), and immune infiltrated (CD8+ T cells infiltrate the tumor epithelium) using a machine-learning approach in a previously published analysis [30]. Cytolytic activity was highly associated (*P* = 4.9 × 10^− 24^) with tumor-immune phenotype where activity was increased in immune excluded tumors and even more so for immune infiltrated tumors compared to immune desert tumors (Fig. 3A). EZH2 mRNA expression was significantly downregulated in immune excluded tumors compared to immune deserts and immune infiltrated tumors (*P* = 0.001; Fig. 3A). No clinicopathologic factors (age, stage, or residual disease) were associated with differential EZH2 expression, cytolytic activity, or tumor-immune phenotypes (Table S3, Additional file 1).

### EZH2 expression and co-expression associated with prognosis in TCGA

We next evaluated EZH2 expression by clinical outcomes and found both EZH2 mRNA and protein levels were significantly lower in the 29% of HGSOC with platinum resistance (Fig. 3B) and were significantly associated with RTT after adjustment for tumor purity, residual disease, and molecular subtype (mRNA *P* = 0.003, protein *P* = 0.03). The association did not vary by molecular subtype for mRNA (P_interaction_ = 0.44) or protein (P_interaction_ = 0.46). We also examined whether the association between EZH2 expression and RTT varied by tumor immune features. The mRNA and protein associations did not vary by cytolytic activity (P interaction = 0.70 and 0.23, respectively) or by tumor-immune phenotype (P interaction = 0.85 and 0.32, respectively). Neither mRNA nor protein EZH2 expression was associated with overall survival (mRNA *P* = 0.44, protein *P* = 0.57) or HGSOC molecular subtype-specific survival.

To determine whether the relationship between *EZH2* and RTT was dependent upon the expression of other genes, we tested for genes that interacted with *EZH2* expression in the prognostic model. We classified 164 genes as ‘modifier’ genes that had a significant statistical interaction (*P* < 0.02; top 1%, see Table S4, Additional file 1) with *EZH2* expression levels in the RTT logistic model (see Methods). Top modifier genes included *CERS4* (P_interaction_ = 0.001) and *CARM1* (P_interaction_ = 0.001). Of the modifier genes, 72% (*n* = 118) negatively modified or decreased the prognostic significance of *EZH2*, including arginine methyltransferase *CARM1*, and 28% positively modified (increased) the prognostic significance of *EZH2* such as lncRNA *MALAT1* (Fig. 3C). Given that the top modifiers *CARM1* and *MALAT1* affected EZH2 prognostic value in opposite directions, we investigated their co-expression and found that *CARM1* and *MALAT1* (also known as *NEAT2*) were negatively correlated (*r =* − 0.15, *P* = 0.004) across HGSOC tumors. Since CARM1 transcriptionally downregulates lncRNA *NEAT1* by binding to its promoter [31], we assessed the correlation between *NEAT1* and downstream *MALAT1* and found a strong correlation (*r =* 0.74, *P* = 2.8 × 10^− 60^), suggesting CARM1 may also downregulate *MALAT1*. We further assessed the relationship of *CARM1* with BRG1 (*SMARCA4*), the catalytic subunit of the SWI/SNF chromatic remodeling complex that is known to physically interact with *MALAT1* and is an antagonist to the EZH2/PRC2 complex [32]. We found *CARM1* was strongly correlated with *SMARCA4* expression (*r =* 0.79, *P* = 4.9 × 10^− 71^).

We next performed genome-wide co-expression analysis for *EZH2* among all HGSOC samples, and by response to therapy to identify *EZH2* expression networks and how they may differ in platinum resistant versus sensitive tumors. Among all HGSOC samples, transcriptional *EZH2* was positively co-expressed with 1448 genes including known cofactors (i.e. proteins that physically interact with EZH2) *XRCC2*, *BRCA1, BRCA2*, and DNA methyltransferase *DNMT1* and negatively co-expressed with 990 genes including tumor suppressor gene *ZBTB4* [33] (Fig. S3; see Table S5, Additional file 1). Differential co-expression analysis was performed to compare EZH2 co-expression by RTT. We identified 1049 mRNA that were differentially correlated (*P* < 0.05) with *EZH2* in platinum resistant HGSOC compared to platinum sensitive (Fig. 3D; see Table S6, Additional file 1). This included 559 genes that were exclusively correlated with EZH2 in platinum resistant tumors (Fig. S4, Additional file 1) and 440 genes that were exclusively correlated with *EZH2* in platinum sensitive tumors (Fig. S5, Additional file 1). Notably, over 80% (*n* = 135) of the modifier genes identified in our previous interaction analysis were also differentially correlated with *EZH2* based on RTT, indicating that the change in *EZH2* prognostic value is mediated by the changes in its transcriptional network. For example, the modifier *CARM1* was positively correlated with *EZH2* in resistant tumors (*r =* 0.33, *P* = 0.003) but not in sensitive tumors (*r =* − 0.05, *P* = 0.49), representing a distinct correlation (Fig. 3E) that coincides with EZH2 downregulation in resistance (Fig. 3C). Gene ontology (GO) enrichment analysis was performed conditionally on the hierarchical structure of the GO database to reduce redundancy and identify enriched child term biological processes. This revealed that the platinum sensitive *EZH2* co-expression network was enriched for genes in immune-related biological processes (T cell activation/differentiation) and metal toxicity (detoxification/response to copper/zinc) while the network exclusive to platinum resistant tumors was enriched for transcriptional regulation processes and RNA synthesis, such as RNA polymerase II subunit *POLR2F* (Fig. 3F). Unconditional gene set enrichment analysis from the Molecular Signatures Database (v7.3) detected analogous biological process enrichments with larger GO parent terms detected and hence larger gene sets from co-expression networks represented (Fig. S6, Additional file 1).

We similarly performed co-expression analysis for EZH2 protein levels. Forty-nine genes had correlated protein expression with EZH2 at FDR of 15% (see Table S7, Additional file 1). Most proteins (*n* = 36) were positively co-expressed and included PARP2 (*r =* 0.46, *P* = 8.9 × 10^− 5^, FDR = 0.08). Differential correlation analysis with EZH2 identified 448 proteins exclusively correlated with EZH2 based on RTT (see Table S8, Additional file 1). Distinct from the transcriptional analysis, most differentially correlated proteins (76%, *n* = 339) were correlated with EZH2 in resistant HGSOC. The most significant difference in protein co-expression was observed for HDAC7 which was negatively correlated with EZH2 in resistant tumors but not correlated with EZH2 in sensitive tumors (sensitive r = 0.07, *P* = 0.67; resistant *r =* − 0.86, *P* = 1.4 × 10^− 6^). Interestingly, at the mRNA level, *HDAC7* was not differentially correlated with *EZH2* but was correlated with the modifier *CARM1* (*r =* 0.14, *P* = 0.01). Analysis of gene ontologies found that genes exclusively correlated with EZH2 in resistant tumors were enriched for protein regulation (Fig. 3F). Strikingly, only 23 of the genes that were differentially correlated with EZH2 at the mRNA level were also differentially correlated at the protein level (Fig. 3F). Further, nineteen of these genes displayed the same direction of correlation in both mRNA and protein expression including CARM1.

## Discussion

EZH2 plays a critical role in the development, progression, and chemo-resistance of EOC where it has been shown to have a prognostic significance [34–36]. We investigated the expression of EZH2 in HGSOC immunohistochemically, and consistent with prior studies, found EZH2 expression was predictive of chemotherapy resistance and shorter survival; however, we only observed this for HGSOC with distinct morphologic features, including those without infiltrating lymphocytes (TIL-) and those with lymphovascular invasion (LVI+). EZH2 overexpression is known to correlate with biologically aggressive EOC features yet prognostic associations have been independent of these factors. Now, our analysis has revealed a mixed prognostic ability for EZH2 dependent on immunogenic and angiogenic tumor properties.

To elucidate molecular correlates underlying the observed EZH2 association with RTT, we explored transcriptomic and proteomic co-expression networks within TCGA. In contrast to findings from our TMA, high EZH2 mRNA (and correlated protein) expression was associated with platinum response and longer survival times in TCGA tumors. This finding agrees with a recent study that found EZH2 protein levels were elevated in serous tumors that responded to platinum therapy and correlated with longer disease-free survival [37]. Furthermore, the association between high EZH2 mRNA expression and better survival was replicated by microarray expression data from 616 samples compiled from Gene Expression Omnibus (GEO) datasets, including the TCGA samples evaluated in this study, and confirms the association we observed in all available public data. Notably, the authors also performed an IHC quantification of EZH2 which failed to detect an increase in EZH2 among those with better survival. Our results similarly show a discrepancy between results from different quantification methods that may be due to the lower sensitivity of IHC. The discrepancy in our results may also reflect that TCGA data were generated from bulk tumor samples which capture a wide array of tumor cells that could have heterogeneous EZH2 expression. Indeed, loss of EZH2 expression at the tumor invasion front in colorectal cancer has been associated with a more aggressive phenotype and shorter survival due to reduced proliferation and acquisition of mesenchymal characteristics [38]. Moreover, cancer stem cells exhibit reduced EZH2 expression that is associated with higher transcriptional activity and cellular plasticity [39]. Considering these findings, the lower EZH2 expression in platinum resistant HGSOC may result from more invasive cellular phenotypes and higher prevalence of stem-like tumor cells that contribute to an intrinsic resistance to chemotherapy. Conversely, the TMA was, by design, a homogeneous sampling of tumor epithelium where higher EZH2 expression may contribute to chemotherapy resistance through separate mechanisms – conceivably through immune-related pathways since TIL presence affected the prognostic significance.

Presence of TIL indicates an active host immune response to tumor antigens and has been associated with favorable prognosis [14] as well as higher rates of complete chemotherapy response [40]. In our clinical series, EZH2 was overexpressed in patients with platinum resistance and TIL- tumors, suggesting that immune-evasion underlies the association. In fact, epigenetic silencing is a critical immune-evasion mechanism enlisted by tumors. EZH2-mediated methylation has been demonstrated to repress T helper 1 (Th1) chemokines, CXCL9 and CXCL10, resulting in reduced T-cell recruitment to the tumor microenvironment [41]. Thus, EZH2 has a pivotal role in TIL- tumors where higher expression contributes to immune-evasion and consequently, as our data indicate, an increased likelihood of platinum resistance. On the other hand, TIL+ HGSOC are absent the epigenetic mechanisms involving EZH2 which may explain its diminished significance for prognosis in these tumors. Although TCGA tumors were not pathologically reviewed for TIL characteristics, we were able to incorporate a cytolytic activity score and a transcriptome-based tumor-immune phenotype to evaluate EZH2 in context of the immune features. However, we did not observe an association between EZH2 expression and prognosis that was dependent upon these immune characteristics. It is possible that the metrics we used were not able to capture the complexity of T-cell spatial and quantity features. Further, the prognostic relevancy of EZH2 expression may only be within a particular habitat. Indeed, distinct immune microenvironments coexist within the same tumor and higher variability of T-cell infiltration and type (CD8+, CD4+ and Treg cells) exists within a tumor than across tumor sites and patients [42]. Thus, it is unclear how the prognostic value of EZH2 in TIL- regions can be recapitulated with bulk samples. It is of interest to delineate the associations between EZH2, tumor immune phenotypes, and prognosis in future studies.

We observed a molecular context-dependent relationship between *EZH2* expression and RTT in the TCGA data. Transcriptome analysis revealed that platinum resistant tumors exhibited lower bulk tumor *EZH2* expression with low *CARM1* (and high *MALAT1*) co*-*expression*. CARM1* mRNA levels were also strongly correlated with the *SMARCA4* (BRG1) subunit of the SWI/SNF complex, illustrating a distinct regulatory network in refractory tumors where SWI/SNF and PRC2 factors are positively co-expressed. The observed downregulation of these antagonistic complexes in association with chemoresistance is intriguing. CARM1 can methylate and displace SWI/SNF from target genes and consequently promote EZH2 placement and gene silencing [43]; however, *EZH2* was prognostic in low *CARM1*-expressing tumors, likely precluding this as an underlying mechanism. SWI/SNF complexes both activate and repress transcription through recruitment of histone deacetylases (HDACs) and have a critical role in promoting pluripotency in somatic and embryonic stem cells [44]. Interestingly, we also observed that EZH2 protein in platinum resistant tumors was negatively correlated with HDAC7 which is transcriptionally upregulated in ovarian cancer stem cells [45]. Thus, the downregulation of both SWI/SNF and PRC2 complex factors but upregulation of HDAC7 suggests EZH2-associated platinum resistance may reflect increased tumor stemness.

Finally, our assessment of our clinical samples included a detailed evaluation of morphologic patterns at primary and metastatic anatomical sites and demonstrated that none of the architectural patterns are associated with RTT in concordance with previous reports [46]. The correlation between micropapillary pattern and LVI has been documented in other tumor subtypes [47, 48] and it was associated with advance disease stage in our analysis. Micropapillary pattern and reduced e-cadherin expression were reportedly observed more commonly in recurrent tumors and the presence of cystic component and nonpapillary architecture in non-recurrent HGSOC samples [15]. In our series, presence of cystic component nullified the possible adverse effect of papillary architecture in overall survival suggesting that cystic component might contribute to disease confinement within the ovaries and avoiding disease spread through peritoneal surfaces. Another possible favorable prognostic pattern was the presence of solid component in the absence of TIL. The so-called SET pattern (Solid-Pseudoendometrioid and Transitional) with lymphocytic infiltrates was previously described in serous tumors of BRCA mutation carriers and it is associated with better survival results [16]. Overall, tumor behavior seems to be dependent on combination of multiple factors rather than a single morphologic feature.

In conclusion, we found that the expression of EZH2 depends upon complex interactions with immunologic pathways and local tumor microenvironment that alter its prognostic interpretation and may depend upon the subpopulation of tumor cells. The association between high EZH2 expression and platinum resistance in a low TIL microenvironment may have implications for therapeutic strategies. Epigenetic reprogramming of tumors with EZH2 inhibitors has been observed to improve the efficacy of T cell therapy and PD-L1 blockade therapy [49]. Our data suggest these combination therapies might be most efficacious in TIL- HGSOC which warrants further investigation.

## Supplementary Information

**Additional file 1: Table S1:** Characteristics of Study Participants by BRCA Status. **Table S2:** Cross frequency table for architectural growth patterns. **Table S3.** Associations between Molecular and Clinicopathologic Factors in TCGA. **Table S4:** List of Top 1% of Modifier Genes and their Interaction Term OR and Pvalues. **Table S5:** Significant EZH2 mRNA Correlations from TCGA HGOSC. **Table S6:** Genes with significant (*P* < 0.05) mRNA differential correlation (r2) with EZH2 in platinum resistant (PR) versus platinum sensitive (PS) HGOSC (TCGA). **Table S7:** Significant EZH2 Protein Correlations from TCGA HGOSC. **Table S8:** Genes with significant (*P <* 0.05) protein differential correlation (r2) with EZH2 in platinum resistant (PR) versus platinum sensitive (PS) HGOSC (TCGA). **Fig. S1.** Tumor EZH2 expression by Clinical Factors. **Fig. S2.** Digital Hscore for EZH2 expression by tissue type. **Fig. S3.** Volcano plot for co-expression of 31, 237 genes with EZH2 in TCGA HGSOC (*n* = 336). **Fig. S4.** Planar filtered network of genes correlated with EZH2 exclusively in platinum resistant HGSOC. **Fig. S5.** Planar filtered network of genes correlated with EZH2 exclusively in platinum sensitive HGSOC. **Fig. S6.** Gene set enrichment analysis for co-expression networks using Molecular Signatures Database.

## Data Availability

The tissue microarray datasets generated and analysed during the current study are not publicly available but are available from the corresponding author upon reasonable request. ‘The RNA sequencing dataset analysed during the current study is available with Level 1 access from the TCGA repository (https://cancergenome.nih.gov/). The gene-level FPKM data generated in this study from TCGA Level 1 RNA sequencing reads is available from the corresponding author upon reasonable request. The proteomics dataset for the TCGA samples is publicly available through the CPTAC repository (https://cptac-data-portal.georgetown.edu/).

## Abbreviations

CPTAC: Clinical Proteomic Tumor Analysis Consortium
EOC: Epithelial ovarian cancer
EZH2: Enhancer of zesta homologue 2
HGSOC: High-grade serous ovarian carcinoma
IHC: Immunohistochemistry
LVI: Lymphovascular invasion
OS: Overall survival
PRC2: Polycomb repressive complex 2
RTT: Response to therapy
TCGA: The Cancer Genome Atlas
TIL: Tumor-infiltrating lymphocyte aggregates ≥1 mm
TMA: Tissue microarray
