# Supplementary material for: Morphologic and molecular correlates of EZH2 as a predictor of platinum resistance in high-grade ovarian serous carcinoma
Source: BMC Cancer. 2021 Jun 17;21:714. doi: 10.1186/s12885-021-08413-3 (PMC8212453; doi:10.1186/s12885-021-08413-3)

Title: Morphologic and molecular correlates of EZH2 as a predictor of platinum resistance in high-grade ovarian serous carcinoma

Authors: Brett M. Reid[a], Shraddha Vyas[a], Zhihua Chen[b], Ann Chen[b], Peter A. Kanetsky[a], Jennifer B. Permuth[a], Thomas A. Sellers[c*], and Ozlen Saglam[d*]

Affiliations: a) Cancer Epidemiology, Moffitt Cancer Center, Tampa, FL, b) Biostatistics and Bioinformatics, Moffitt Cancer Center, Tampa, FL, c) Independent researcher, d) Department of Pathology, Moffitt Cancer Center, Tampa, FL

*These authors contributed equally to this work.

Table S1: Characteristics of Study Participants by BRCA Status

	All	BRCA Status		
		Carrier	Non-Carrier	P
N	21	11	10	
**Age at diagnosis, mean (std)**	55 (11.2)	49 (7.8)	61 (11.3)	0.01
**Stage**				0.33
I,II	1 (1)	1 (9)	0	
III,IV	20 (99)	10 (91)	10 (100)	
**Debulking status**				0.92
Suboptimal (Residual disease >1cm)	4 (19)	2 (18)	2 (20)	
Optimal (NED or residual disease <1cm)	17 (81)	9 (82)	8 (80)	
**Lymphovascular Involvement**				0.41
Yes	15 (71)	7 (64)	8 (80)	
No	6 (29)	4 (36)	2 (20)	
**Cystic Component**				0.89
Yes	6 (29)	3 (27)	3 (30)	
No	15 (71)	8 (73)	7 (70)	
**STIC**				0.28
Yes	9 (47)	3 (30)	6 (66)	
No	10 (53)	7 (70)	3 (33)	
**TIL**				0.86
Yes	8 (38)	4 (33)	4 (40)	
No	13 (62)	7 (66)	6 (60)	
**Response to Therapy**				
Sensitive	17 (81)	8 (67)	9 (90)	0.31
Resistant	4 (19)	3 (25)	1 (10)	

Table S2: Cross frequency table for architectural growth patterns

Micropapillary	Papillary	Pseudoglandular	Solid	N	%
0	0	0	1	3	3.8
0	0	1	0	1	1.27
0	0	1	1	3	3.8
0	1	0	0	1	1.27
0	1	0	1	3	3.8
0	1	1	0	4	5.06
0	1	1	1	15	18.99
1	0	0	0	1	1.27
1	0	0	1	4	5.06
1	0	1	0	1	1.27
1	0	1	1	6	7.59
1	1	0	0	2	2.53
1	1	0	1	5	6.33
1	1	1	0	13	16.46
1	1	1	1	17	21.52

Table S3. Associations between Molecular and Clinicopathologic Factors in TCGA

	EZH2 mRNA expression (log2)		P	Cytolytic activity (log2)		P	Immune Phenotype*			P
							**Desert**	**Excluded**	**Infiltrated**	
N										
	OR (se)			*OR (se)*			*mean (sd)*			
**Age at diagnosis, beta (se)**	1.00 (0.003)		0.78	1.00 (0.003)		0.55	60.4 (11.4)	61.7 (11.4)	58.7 (10.9)	0.42
**Stage**	*mean (sd)*	*OR (se)*		*mean (sd)*	*OR (se)*		*n (%)*			
I,II	2.95 (0.56)	ref	0.1	2.26 (0.95)	ref	0.31	4 (3%)	0 (0)	10 (9%)	0.07
III,IV	2.74 (0.67)	0.76 (0.17)		1.96 (0.66)	0.86 (0.15)		144 (97%)	37 (100%)	105 (91%)	
**Debulking status**	*mean (sd)*	*OR (se)*		*mean (sd)*	*OR (se)*		*n (%)*			
Optimal (NED or residual disease <1cm)	2.74 (0.67)	*ref*	0.74	1.95 (0.67)	ref	0.37	99 (73%)	25 (71%)	79 (77%)	0.48
Suboptimal (Residual disease >1cm)	2.71 (0.61)	0.94 (0.08)		2.00 (0.68)	1.07 (0.07)		37 (27%)	10 (29%)	24 (23%)	

*n=28 tumors were unclasssified for tumor-immune phenotype

Categorical pvalues were calculated using chiseq test (residual disease) and Fisher exact test (Immune phenotype)

EZH2 and CYT were modeled as dependent variables in a linear model with clinical factor entered as indendendent variable and adjusted for tum

Table S4: List of Top 1% of Modifier Genes and their Interaction Term OR and Pvalues

Gene	OR	Interaction Pvalue	Qvalue
CERS4	0.370878	0.00112066	0.999604
RP1-122P22.4	0.082197	0.00112453	0.999604
CARM1	0.207807	0.0013372	0.999604
LINC01356	0.02634	0.00144885	0.999604
TIMM44	0.2573	0.001752	0.999604
E2F5	0.191772	0.00187085	0.999604
RP11-110G21.1	0.143851	0.00203892	0.999604
ICAM5	0.375958	0.00214607	0.999604
FAM66B	0.016064	0.00231718	0.999604
AF186192.1	0.336261	0.00271743	0.999604
ZNF136	0.19011	0.0027333	0.999604
RP3-425C14.4	4.203933	0.00293623	0.999604
BRF2	0.194554	0.00295206	0.999604
ZNF57	0.104902	0.00307245	0.999604
LAYN	0.404004	0.00359139	0.999604
PGAP2	0.226333	0.00360336	0.999604
RIC3	0.33041	0.00365813	0.999604
SKIDA1	0.229652	0.00391661	0.999604
ZNF77	0.150317	0.003918	0.999604
CTD-2666L21.1	0.025052	0.00409623	0.999604
TMED1	0.257942	0.0042111	0.999604
ZNF561	0.25931	0.00433248	0.999604
MALAT1	1.845466	0.00457792	0.999604
ILVBL	0.343695	0.00472245	0.999604
CLVS1	0.041278	0.00472265	0.999604
RP11-1252D15.1	0.304026	0.00475611	0.999604
Y_RNA.795	2.578275	0.00476256	0.999604
DYNC1I1	0.18841	0.00486608	0.999604
ARHGEF19	0.376818	0.00502298	0.999604
CFD	2.030296	0.00514311	0.999604
FBXL12	0.237226	0.00547067	0.999604
NDUFA7	0.013856	0.00549794	0.999604
MYCN	0.587388	0.00554678	0.999604
CTC-543D15.8	0.384556	0.00561956	0.999604
TMCC2	0.364958	0.00562458	0.999604
RNF170	0.150834	0.00571438	0.999604
ZNF729	0.017613	0.00593747	0.999604
RP13-270P17.2	0.032363	0.00609269	0.999604
ZNF558	0.242138	0.00621174	0.999604
C2	2.022273	0.00621929	0.999604
MAP3K5	3.874304	0.00630696	0.999604
AC106875.1	0.062012	0.00653212	0.999604

TMEM74B	0.260213	0.00654436	0.999604
AF064858.11	0.134691	0.00676149	0.999604
ZNF414	0.352336	0.00685795	0.999604
HLA-L	4.567968	0.00689799	0.999604
CTC-325H20.8	0.284172	0.00698219	0.999604
LL22NC03-2H8.5	3.95141	0.00698769	0.999604
AC016995.3	0.146943	0.00720667	0.999604
CTD-2342J14.6	0.153258	0.00721171	0.999604
AF186192.5	0.348596	0.00749428	0.999604
CCNB1IP1	0.435392	0.00765335	0.999604
LHB	0.037979	0.00780749	0.999604
RP3-331H24.7	4.079105	0.00816701	0.999604
SFTA2	3.42042	0.00830806	0.999604
AC010884.1	0.061181	0.00844983	0.999604
LYPLAL1-AS1	0.058461	0.00855259	0.999604
TTC31	0.184012	0.00859644	0.999604
C8orf89	0.03439	0.00861629	0.999604
SCARNA11	0.122826	0.00877103	0.999604
RP11-582E3.6	3.587102	0.00899176	0.999604
TMEM155	0.053674	0.00918429	0.999604
MAOB	1.886926	0.00947844	0.999604
CNDP2	2.98854	0.00950406	0.999604
PCAT6	0.528596	0.00951085	0.999604
TNS2	2.301421	0.00957093	0.999604
PLIN1	3.752021	0.00966787	0.999604
DUS3L	0.285819	0.00971697	0.999604
KIF21A	4.869291	0.00984451	0.999604
RAVER1	0.316282	0.01000996	0.999604
SNORD93	0.348286	0.01007083	0.999604
RNU7-45P	2.515423	0.01008787	0.999604
RASSF8	2.848982	0.01011934	0.999604
RP11-293A21.1	0.316016	0.01017809	0.999604
ARPIN	4.000897	0.0102473	0.999604
ZNF266	0.325853	0.01067972	0.999604
TECTA	0.013424	0.01089862	0.999604
ZNF317	0.292514	0.0110411	0.999604
DPY19L4	0.233555	0.01123231	0.999604
RP11-7F18.2	0.131236	0.01147015	0.999604
RP11-84C13.2	0.03309	0.011548	0.999604
ZNF440	0.403739	0.01156506	0.999604
OXTR	0.620633	0.01159003	0.999604
KEAP1	0.275371	0.0116862	0.999604
ARFGAP3	4.112551	0.01171943	0.999604
LNP1	0.337268	0.01182129	0.999604
NAP1L4	0.222741	0.01219551	0.999604
INTS8	0.233087	0.01229191	0.999604
ZBTB8B	0.016838	0.01229617	0.999604

KLK14	1.628839	0.01234266	0.999604
RP11-707G14.8	0.041307	0.01261399	0.999604
RAB3A	0.368272	0.01266256	0.999604
SLC20A2	0.32491	0.01272627	0.999604
SULT1C2	2.653768	0.01294679	0.999604
ZNF426	0.242709	0.01300184	0.999604
GOLGA7	0.364819	0.0131681	0.999604
RP4-777L9.2	0.034643	0.01353826	0.999604
C9orf152	2.288042	0.01355413	0.999604
CRYGEP	0.449423	0.01392328	0.999604
SLFN5	2.347607	0.01392824	0.999604
AP3M2	0.238954	0.01397763	0.999604
ZNF559	0.234472	0.01400158	0.999604
RP3-522D1.1	0.096923	0.01408989	0.999604
RASSF8-AS1	2.747475	0.01423072	0.999604
GPT2	0.404788	0.01428479	0.999604
Y_RNA.175	4.190647	0.01432664	0.999604
RPS10	0.50792	0.01436184	0.999604
RP11-603B24.2	0.229679	0.01444495	0.999604
ZNF433	0.307752	0.01444989	0.999604
MAN2B1	0.299256	0.01454022	0.999604
CHST6	3.31308	0.01466835	0.999604
PXDNL	0.042854	0.01478456	0.999604
AC113608.1	0.142458	0.01499311	0.999604
MCOLN1	0.29773	0.01511988	0.999604
RP1-154K9.2	0.028374	0.01523507	0.999604
YIPF2	0.417269	0.01546072	0.999604
CTD-2012J19.2	2.242391	0.01565264	0.999604
CDCP1	2.251871	0.01572728	0.999604
AC007204.2	0.138775	0.0158315	0.999604
ZNF627	0.387066	0.01611944	0.999604
RP1-122P22.2	0.089984	0.01614892	0.999604
PPP1R1B	1.469468	0.01616295	0.999604
BAALC-AS1	0.200146	0.01630559	0.999604
KRI1	0.328145	0.01633728	0.999604
PPAN	0.184889	0.01639124	0.999604
CTD-2650P22.1	0.033134	0.01651605	0.999604
ILF3	0.312983	0.01658116	0.999604
LINC00997	4.505899	0.01691369	0.999604
Y_RNA.495	0.161497	0.01703023	0.999604
ATG4D	0.404636	0.01711919	0.999604
POLB	0.38261	0.01713994	0.999604
TMEM176A	1.613981	0.01750932	0.999604
RP11-505E24.2	0.286871	0.01754565	0.999604
SPTSSB	3.418954	0.01761111	0.999604
HIST1H2APS3	0.295134	0.01764276	0.999604
HMGCS2	2.680482	0.01764305	0.999604

GPAT4	0.236533	0.01766082	0.999604
SPIDR	0.206394	0.01770626	0.999604
CTA-989H11.1	3.869313	0.01770708	0.999604
LINC01285	0.172632	0.01773287	0.999604
FARSA	0.364058	0.01787498	0.999604
ELL	0.26189	0.01791527	0.999604
BNIP3P30	0.252742	0.01792488	0.999604
SLC16A2	2.132167	0.01800334	0.999604
UTRN	2.886773	0.01818636	0.999604
EIF3G	0.383753	0.01829257	0.999604
CA12	1.818055	0.01835949	0.999604
RP11-930P14.2	0.142771	0.01843864	0.999604
ITIH6	0.014087	0.01868392	0.999604
AC069200.1	2.698922	0.01874266	0.999604
FAM175A	0.229095	0.01884133	0.999604
WISP2	4.892435	0.01886197	0.999604
TMEM181	4.045369	0.01903438	0.999604
RPS10P3	0.165006	0.01915067	0.999604
ITPR1-AS1	0.0791	0.01916726	0.999604
LRP11	2.301653	0.01918961	0.999604
PLIN4	3.000775	0.01925009	0.999604
RP11-983P16.4	2.742097	0.01925302	0.999604
AC087491.2	4.460003	0.01937093	0.999604
UBE2E2-AS1	0.063843	0.01946757	0.999604
RP11-214N9.1	0.280845	0.01971525	0.999604
RP11-359M6.2	3.539636	0.01976288	0.999604
QARS	0.286064	0.01979281	0.999604
ZNF823	0.332774	0.01999485	0.999604

Table S5: Significant EZH2 mRNA Correlations from TCGA HGOSC

Gene	Partial Correlation	Pvalue	FDR
NCAPG2	0.667133684	1.70E-44	1.50E-40
KIF15	0.605403593	7.00E-35	4.11E-31
NUSAP1	0.594976159	1.85E-33	8.15E-30
KIF20A	0.592360594	4.13E-33	1.45E-29
XRCC2	0.591063814	6.14E-33	1.80E-29
HJURP	0.584294472	4.70E-32	1.18E-28
CASC5	0.582585221	7.79E-32	1.71E-28
KIF4A	0.581719513	1.01E-31	1.97E-28
CDC7	0.580875938	1.29E-31	2.27E-28
KIF23	0.57743943	3.52E-31	5.62E-28
DNA2	0.576844371	4.18E-31	6.12E-28
SPC25	0.574849479	7.43E-31	9.72E-28
CLSPN	0.57470924	7.73E-31	9.72E-28
DLGAP5	0.572724733	1.37E-30	1.60E-27
KIFC1	0.568522122	4.50E-30	4.94E-27
CDC45	0.567298653	6.34E-30	6.56E-27
ASPM	0.55701969	1.08E-28	1.05E-25
TPX2	0.554827619	1.94E-28	1.80E-25
IQGAP3	0.553425404	2.83E-28	2.49E-25
FANCI	0.550824792	5.65E-28	4.74E-25
KNTC1	0.547522706	1.35E-27	1.08E-24
KIF2C	0.546551867	1.74E-27	1.33E-24
GAS2L3	0.543800891	3.56E-27	2.61E-24
CDC25A	0.542189558	5.40E-27	3.80E-24
TOP2A	0.53985164	9.83E-27	6.65E-24
RACGAP1	0.538953005	1.24E-26	8.06E-24
CDK1	0.538315716	1.45E-26	9.14E-24
DEPDC1	0.537302621	1.88E-26	1.14E-23
BUB1	0.536250517	2.45E-26	1.44E-23
GPSM2	0.533905159	4.43E-26	2.51E-23
PRC1	0.533436801	4.98E-26	2.74E-23
TROAP	0.532812895	5.82E-26	3.10E-23
LMNB1	0.531886951	7.33E-26	3.79E-23
MELK	0.527562031	2.13E-25	1.07E-22
CDCA7	0.526743618	2.61E-25	1.27E-22
NDC80	0.524586772	4.41E-25	2.10E-22
CENPA	0.52433984	4.68E-25	2.17E-22
RRM2	0.524120383	4.94E-25	2.23E-22
ORC1	0.522441509	7.41E-25	3.26E-22
ECT2	0.520119296	1.29E-24	5.56E-22
SKA1	0.519749753	1.41E-24	5.92E-22
CKAP2L	0.519199075	1.61E-24	6.60E-22
DONSON	0.518642706	1.84E-24	7.36E-22

RAD54L	0.517119901	2.64E-24	1.03E-21
KIF18B	0.516167255	3.31E-24	1.27E-21
PIF1	0.515513836	3.87E-24	1.45E-21
ZNF367	0.515185871	4.18E-24	1.53E-21
CDC25C	0.511870852	9.08E-24	3.26E-21
NCAPG	0.509971647	1.41E-23	4.96E-21
CDKN3	0.508779611	1.86E-23	6.38E-21
CDCA8	0.508712662	1.89E-23	6.38E-21
KIF11	0.508493245	1.98E-23	6.59E-21
BUB1B	0.502877701	7.15E-23	2.33E-20
NCAPH	0.501545697	9.65E-23	3.04E-20
TCF19	0.501538608	9.67E-23	3.04E-20
POLE2	0.501260341	1.03E-22	3.17E-20
STIL	0.501197394	1.04E-22	3.17E-20
NOM1	0.49966564	1.47E-22	4.39E-20
ARHGAP11	0.498861451	1.76E-22	5.16E-20
GTSE1	0.49721095	2.54E-22	7.33E-20
NASP	0.494121811	5.03E-22	1.43E-19
PLK1	0.493115385	6.27E-22	1.75E-19
CDCA3	0.491639691	8.66E-22	2.38E-19
BIRC5	0.490796616	1.04E-21	2.82E-19
HELLS	0.490305797	1.16E-21	3.08E-19
AURKB	0.490221669	1.18E-21	3.08E-19
CHEK1	0.490173372	1.19E-21	3.08E-19
CUL1	0.489761605	1.30E-21	3.29E-19
MCM7	0.489732549	1.31E-21	3.29E-19
CDC20	0.489084578	1.51E-21	3.73E-19
SKA3	0.488586025	1.68E-21	4.10E-19
KIF14	0.487254496	2.24E-21	5.39E-19
MYBL2	0.486886871	2.42E-21	5.75E-19
ZWINT	0.485799966	3.05E-21	7.16E-19
DBF4	0.483917109	4.56E-21	1.06E-18
MKI67	0.48378348	4.69E-21	1.06E-18
CCNE2	0.483759732	4.72E-21	1.06E-18
CENPF	0.482593778	6.04E-21	1.34E-18
ANLN	0.482412929	6.27E-21	1.38E-18
PTTG1	0.481812389	7.12E-21	1.55E-18
ARHGEF39	0.48087497	8.67E-21	1.86E-18
EXO1	0.480146585	1.01E-20	2.14E-18
MCM10	0.477403205	1.79E-20	3.76E-18
E2F8	0.475933089	2.43E-20	5.03E-18
FBXO5	0.475721356	2.54E-20	5.20E-18
UHRF1	0.474195846	3.48E-20	7.04E-18
BARD1	0.473825322	3.76E-20	7.51E-18
TICRR	0.473619161	3.92E-20	7.75E-18
CCNB2	0.473364611	4.13E-20	8.07E-18
MCM4	0.473287583	4.20E-20	8.11E-18

WDR76	0.472584794	4.85E-20	9.27E-18
STRIP2	0.472503537	4.93E-20	9.32E-18
NUF2	0.471822702	5.66E-20	1.06E-17
WDHD1	0.471681166	5.83E-20	1.08E-17
CCNA2	0.470864643	6.88E-20	1.26E-17
CENPE	0.469526254	9.03E-20	1.64E-17
TMPO	0.469080272	9.89E-20	1.77E-17
PAXIP1	0.467716333	1.30E-19	2.31E-17
CENPU	0.467263545	1.43E-19	2.51E-17
MCM6	0.466260138	1.74E-19	3.04E-17
HMGB2	0.463480034	3.04E-19	5.21E-17
CENPK	0.463460787	3.05E-19	5.21E-17
E2F1	0.462118636	3.98E-19	6.73E-17
KIAA1524	0.462055098	4.03E-19	6.75E-17
SGO1	0.46047928	5.50E-19	9.12E-17
NEK2	0.46043399	5.55E-19	9.12E-17
FOXM1	0.460046067	5.99E-19	9.75E-17
TTK	0.459599978	6.54E-19	1.06E-16
E2F7	0.458526448	8.07E-19	1.29E-16
BLM	0.456995369	1.09E-18	1.72E-16
RFC4	0.454352605	1.81E-18	2.85E-16
WDR62	0.452905177	2.40E-18	3.71E-16
CENPI	0.452887421	2.40E-18	3.71E-16
ERCC6L	0.452562783	2.56E-18	3.91E-16
NUP205	0.452458957	2.61E-18	3.96E-16
ESPL1	0.452036091	2.83E-18	4.26E-16
CASP2	0.451113228	3.38E-18	5.03E-16
RAD51AP1	0.450992127	3.45E-18	5.11E-16
SMC4	0.450167513	4.04E-18	5.93E-16
ASF1B	0.449731965	4.39E-18	6.38E-16
CEP55	0.449433005	4.65E-18	6.70E-16
DTL	0.449102049	4.95E-18	7.08E-16
GMNN	0.448626402	5.41E-18	7.68E-16
GINS1	0.448037859	6.05E-18	8.52E-16
PSRC1	0.447580944	6.60E-18	9.21E-16
CDC6	0.446836456	7.59E-18	1.05E-15
POLQ	0.44578378	9.25E-18	1.27E-15
CIT	0.445623796	9.53E-18	1.30E-15
TYMS	0.440973328	2.26E-17	3.06E-15
FEN1	0.439723831	2.85E-17	3.82E-15
FAM111B	0.437179741	4.53E-17	6.04E-15
ZBTB4	-0.436828834	4.83E-17	6.39E-15
PCNA	0.434367555	7.55E-17	9.91E-15
KIAA1841	0.433657915	8.58E-17	1.12E-14
CDCA5	0.433203794	9.31E-17	1.20E-14
UBE2T	0.432871707	9.89E-17	1.27E-14
NEIL3	0.431967466	1.16E-16	1.48E-14

SGO2	0.431722168	1.21E-16	1.54E-14
MND1	0.430657941	1.47E-16	1.85E-14
HMMR	0.429458293	1.82E-16	2.27E-14
CCNB1	0.429353787	1.85E-16	2.30E-14
FANCA	0.42713014	2.75E-16	3.38E-14
TACC3	0.426990383	2.81E-16	3.44E-14
GINS2	0.426895047	2.86E-16	3.47E-14
UBE2C	0.425955047	3.38E-16	4.07E-14
CCNF	0.425573184	3.61E-16	4.32E-14
FAM83D	0.425226177	3.84E-16	4.56E-14
ARHGAP11	0.420175773	9.22E-16	1.09E-13
DEPDC1B	0.420151735	9.26E-16	1.09E-13
BRIP1	0.418640545	1.20E-15	1.40E-13
KIAA0101	0.418311722	1.27E-15	1.47E-13
CDCA2	0.418101916	1.32E-15	1.51E-13
SFPQ	0.416609315	1.70E-15	1.94E-13
RHEB	0.416359566	1.77E-15	2.01E-13
RFC2	0.41592176	1.91E-15	2.15E-13
DEK	0.415087841	2.20E-15	2.46E-13
AC004381.	0.413481794	2.88E-15	3.19E-13
FAM72D	0.413481102	2.88E-15	3.19E-13
CHRNA5	0.411448448	4.06E-15	4.46E-13
POLA2	0.409119902	5.98E-15	6.54E-13
RTKN2	0.408240449	6.92E-15	7.51E-13
ATAD2	0.407341851	8.03E-15	8.66E-13
RBL1	0.407131718	8.31E-15	8.92E-13
KIF18A	0.406762382	8.83E-15	9.42E-13
USP1	0.406264053	9.59E-15	1.02E-12
AURKA	0.404724873	1.23E-14	1.30E-12
SPAG5	0.404673359	1.24E-14	1.30E-12
PRIM1	0.402703402	1.72E-14	1.79E-12
KPNA2	0.402500936	1.77E-14	1.83E-12
CENPL	0.402126904	1.88E-14	1.94E-12
MXD3	0.401132622	2.21E-14	2.26E-12
SPC24	0.401101675	2.22E-14	2.26E-12
FAM72B	0.40046921	2.46E-14	2.49E-12
ABCF2	0.399638391	2.82E-14	2.83E-12
E2F2	0.39935812	2.95E-14	2.94E-12
ESCO2	0.399295775	2.97E-14	2.96E-12
CTB-193M1	0.39687578	4.38E-14	4.33E-12
RAD51	0.396700897	4.50E-14	4.43E-12
Y_RNA.657	0.395620659	5.35E-14	5.23E-12
CDT1	0.395511054	5.44E-14	5.29E-12
DSN1	0.394300152	6.59E-14	6.37E-12
STMN1	0.39323095	7.80E-14	7.50E-12
OIP5	0.393171322	7.87E-14	7.53E-12
RMI1	0.390748232	1.15E-13	1.09E-11

POLE	0.38999272	1.29E-13	1.22E-11
ZNF398	0.388192897	1.71E-13	1.61E-11
CDK2	0.386814175	2.12E-13	1.98E-11
SLFN13	0.384188459	3.17E-13	2.95E-11
RFC5	0.383058872	3.76E-13	3.48E-11
TRA2B	0.381780022	4.57E-13	4.21E-11
H2AFV	0.381570521	4.71E-13	4.32E-11
ORC6	0.38144826	4.80E-13	4.38E-11
WHSC1	0.380411155	5.62E-13	5.09E-11
UBE2S	0.379963389	6.01E-13	5.42E-11
PRR11	0.379533043	6.41E-13	5.75E-11
KIF22	0.379269269	6.67E-13	5.95E-11
DNMT1	0.378150353	7.88E-13	7.01E-11
ZNF212	0.377876064	8.21E-13	7.26E-11
FANCG	0.377103454	9.22E-13	8.11E-11
UBE3C	0.376087426	1.07E-12	9.38E-11
CNTLN	0.375886436	1.10E-12	9.62E-11
PBXIP1	-0.375742375	1.13E-12	9.78E-11
MCM8	0.37567935	1.14E-12	9.82E-11
TIMELESS	0.375242338	1.22E-12	1.04E-10
CHCHD3	0.373915057	1.48E-12	1.26E-10
RP5-894A1	0.373671284	1.53E-12	1.30E-10
AGK	0.372832173	1.73E-12	1.47E-10
PBK	0.372663553	1.78E-12	1.50E-10
RP5-894A1	0.372599527	1.79E-12	1.50E-10
Y_RNA.673	0.37246435	1.83E-12	1.53E-10
GEN1	0.371666213	2.06E-12	1.71E-10
ZNF767P	0.371008691	2.26E-12	1.87E-10
CKS2	0.370754117	2.35E-12	1.93E-10
RANBP1	0.369675432	2.75E-12	2.25E-10
ZWILCH	0.368481059	3.27E-12	2.66E-10
TMPO-AS1	0.367904802	3.55E-12	2.88E-10
ABCB8	0.367240726	3.91E-12	3.15E-10
NBR1	-0.36702315	4.03E-12	3.24E-10
DSCC1	0.366323253	4.46E-12	3.57E-10
SUV39H2	0.36590046	4.74E-12	3.77E-10
CENPN	0.365696578	4.88E-12	3.86E-10
WWC2-AS2	-0.365678344	4.89E-12	3.86E-10
RP11-324E	0.36522806	5.22E-12	4.10E-10
DMC1	0.365074889	5.33E-12	4.17E-10
DEPDC1-AS	0.364903637	5.46E-12	4.25E-10
CENPO	0.364799386	5.54E-12	4.30E-10
FAM64A	0.363954133	6.25E-12	4.83E-10
ARHGAP33	0.363521814	6.65E-12	5.11E-10
CCDC18	0.362793257	7.38E-12	5.64E-10
FAM72A	0.36194216	8.32E-12	6.34E-10
CHAF1A	0.361863706	8.41E-12	6.38E-10

PDIA4	0.361572576	8.77E-12	6.62E-10
DHFR	0.36046241	1.03E-11	7.71E-10
DNAJB6	0.36041331	1.03E-11	7.73E-10
KNSTRN	0.359973426	1.10E-11	8.19E-10
SPRYD3	-0.35957078	1.16E-11	8.62E-10
FBXO43	0.359306241	1.21E-11	8.91E-10
FANCC	0.358896428	1.28E-11	9.40E-10
RP11-650L1	0.358706036	1.31E-11	9.61E-10
DNMT3B	0.358491951	1.35E-11	9.86E-10
ACTL6A	0.357653713	1.52E-11	1.10E-09
ITM2B	-0.357606822	1.53E-11	1.11E-09
CNOT9	0.357560474	1.54E-11	1.11E-09
KIF20B	0.356254579	1.84E-11	1.32E-09
MCM3	0.356108119	1.88E-11	1.35E-09
TRAIP	0.355641601	2.01E-11	1.43E-09
NUB1	0.354523406	2.34E-11	1.66E-09
HMGN2	0.35422462	2.44E-11	1.72E-09
SKP2	0.353943311	2.54E-11	1.79E-09
DDX12P	0.35360773	2.66E-11	1.86E-09
CTD-2510F	0.353060667	2.86E-11	2.00E-09
HNRNPA2B	0.35246369	3.11E-11	2.16E-09
MICB	0.352079292	3.28E-11	2.27E-09
C5orf34	0.351985315	3.32E-11	2.29E-09
HMGB3	0.350568448	4.02E-11	2.77E-09
PHF19	0.350219609	4.22E-11	2.89E-09
SCML2	0.350124116	4.27E-11	2.91E-09
KIF24	0.350086057	4.30E-11	2.92E-09
BRCA2	0.349155984	4.87E-11	3.30E-09
C2orf48	0.348823278	5.10E-11	3.44E-09
RP11-269F	0.348457603	5.35E-11	3.60E-09
CTPS1	0.348278919	5.48E-11	3.67E-09
TCERG1	0.348001351	5.69E-11	3.79E-09
SHCBP1	0.345742613	7.71E-11	5.12E-09
ACBD7	0.345384157	8.08E-11	5.35E-09
RP3-337H4	0.344788821	8.75E-11	5.77E-09
MCM2	0.344717564	8.84E-11	5.80E-09
MASTL	0.34422912	9.43E-11	6.17E-09
RP11-932O	0.343854329	9.91E-11	6.46E-09
RP11-443B	0.343680208	1.01E-10	6.58E-09
ANP32E	0.343534363	1.03E-10	6.69E-09
SNORA2.2	0.342810102	1.14E-10	7.33E-09
CKAP2	0.342490875	1.19E-10	7.62E-09
DNAJC9	0.342068724	1.25E-10	8.03E-09
SMC2	0.341838901	1.29E-10	8.24E-09
REPIN1	0.341727382	1.31E-10	8.34E-09
CHAF1B	0.341633356	1.33E-10	8.41E-09
NDE1	0.341566178	1.34E-10	8.45E-09

VAT1	-0.340877924	1.47E-10	9.22E-09
TRIP13	0.339379721	1.79E-10	1.12E-08
GINS3	0.338686938	1.95E-10	1.22E-08
CHEK2	0.33785439	2.18E-10	1.35E-08
CENPM	0.337496693	2.28E-10	1.41E-08
ACRV1	0.336687767	2.53E-10	1.56E-08
CEP152	0.335814118	2.84E-10	1.74E-08
LRR1	0.335782395	2.85E-10	1.75E-08
CCDC150	0.335746483	2.86E-10	1.75E-08
ANXA9	-0.335668407	2.89E-10	1.76E-08
RP11-119K	0.335646953	2.90E-10	1.76E-08
CDKN2B-AS	0.335616075	2.91E-10	1.76E-08
FIGNL1	0.335395514	2.99E-10	1.80E-08
RP4-584D1	0.335229726	3.06E-10	1.84E-08
RP4-616B8	0.334835583	3.22E-10	1.92E-08
PLK4	0.334369851	3.41E-10	2.04E-08
TEX30	0.334072357	3.55E-10	2.11E-08
AC009403.	0.333657271	3.74E-10	2.22E-08
C1orf112	0.333559655	3.79E-10	2.24E-08
PSIP1	0.333393031	3.87E-10	2.28E-08
ACBD4	-0.333140618	3.99E-10	2.34E-08
MTFR2	0.331853458	4.71E-10	2.75E-08
LUC7L2	0.331288681	5.06E-10	2.95E-08
TOPBP1	0.331192068	5.12E-10	2.97E-08
ARL6IP1	0.330584256	5.53E-10	3.20E-08
STX8P1	0.33004807	5.92E-10	3.41E-08
CEP85	0.329992774	5.96E-10	3.42E-08
PARPBP	0.329762743	6.13E-10	3.51E-08
SRSF2	0.329396176	6.42E-10	3.67E-08
GPR19	0.328846734	6.88E-10	3.92E-08
CHAC2	0.328521322	7.17E-10	4.07E-08
CPQ	-0.328360674	7.31E-10	4.14E-08
SUMF1	-0.328163528	7.50E-10	4.23E-08
FASTK	0.327610824	8.03E-10	4.52E-08
SSX2IP	0.327031412	8.64E-10	4.84E-08
CDCA4	0.326299564	9.46E-10	5.29E-08
TK1	0.325882095	9.97E-10	5.55E-08
DDX39A	0.325661671	1.02E-09	5.69E-08
SSBP1	0.325545633	1.04E-09	5.75E-08
FANCL	0.325277257	1.07E-09	5.93E-08
BORA	0.324619619	1.17E-09	6.41E-08
CEP78	0.324508708	1.18E-09	6.48E-08
PSMC3IP	0.323733588	1.30E-09	7.11E-08
SASS6	0.32307054	1.41E-09	7.67E-08
SRRT	0.323063784	1.41E-09	7.67E-08
INCENP	0.321791496	1.65E-09	8.94E-08
INSIG1	0.321501331	1.71E-09	9.23E-08

PPL	-0.320511285	1.93E-09	1.04E-07
EEF2	-0.320236488	2.00E-09	1.07E-07
LDOC1	-0.319858442	2.09E-09	1.12E-07
ZNF620	0.319614374	2.15E-09	1.14E-07
C9orf40	0.319614014	2.15E-09	1.14E-07
XRCC3	0.319168549	2.27E-09	1.20E-07
CENPP	0.319149076	2.28E-09	1.20E-07
PLA2R1	-0.318815599	2.37E-09	1.25E-07
RP11-16O9	0.31834231	2.51E-09	1.32E-07
APH1B	-0.318208784	2.55E-09	1.34E-07
SPDL1	0.317998985	2.62E-09	1.37E-07
RECQL4	0.317987924	2.62E-09	1.37E-07
RNU7-20P	0.317805793	2.68E-09	1.39E-07
MTBP	0.317371996	2.83E-09	1.46E-07
GNPTG	-0.316878019	3.00E-09	1.55E-07
ZDHHC1	-0.316794636	3.03E-09	1.56E-07
PKMYT1	0.316069073	3.30E-09	1.69E-07
TSPAN31	-0.31540238	3.58E-09	1.83E-07
ALYREF	0.314181462	4.14E-09	2.11E-07
TMEM9B	-0.314111568	4.17E-09	2.12E-07
HAUS6	0.313905079	4.28E-09	2.17E-07
RNASEH2A	0.313804671	4.33E-09	2.19E-07
FAM57B	0.313598658	4.44E-09	2.23E-07
TUBA1B	0.313595308	4.44E-09	2.23E-07
SLC1A4	0.313306837	4.59E-09	2.30E-07
MEF2D	-0.313270297	4.61E-09	2.31E-07
ZNF746	0.313179653	4.66E-09	2.32E-07
LINC01224	0.313147927	4.68E-09	2.33E-07
TNFSF12	-0.312389795	5.12E-09	2.54E-07
snoU13.23	0.312295077	5.18E-09	2.56E-07
PASK	0.311811614	5.48E-09	2.70E-07
MSH2	0.311493724	5.69E-09	2.80E-07
ASNS	0.311350661	5.79E-09	2.84E-07
SMC6	0.311330216	5.80E-09	2.84E-07
ITGB4	-0.310684289	6.26E-09	3.05E-07
NUP85	0.310359225	6.51E-09	3.16E-07
GOLGA8UP	0.310094459	6.71E-09	3.25E-07
LMBR1	0.309825319	6.93E-09	3.35E-07
MYO3A	0.309420018	7.26E-09	3.50E-07
RP11-527J8	0.309333179	7.34E-09	3.53E-07
E2F3	0.309231139	7.43E-09	3.56E-07
EWSR1	0.309103507	7.54E-09	3.60E-07
SERINC3	-0.308624356	7.97E-09	3.80E-07
ZNF786	0.308447789	8.14E-09	3.87E-07
MYBL1	0.30822343	8.35E-09	3.96E-07
DIAPH3	0.308126455	8.45E-09	3.99E-07
TRMU	0.307915131	8.66E-09	4.08E-07

CENPW	0.307453218	9.13E-09	4.30E-07
FANCD2	0.307287584	9.31E-09	4.37E-07
RP5-967N2	0.307184098	9.42E-09	4.41E-07
KCTD11	-0.306715133	9.95E-09	4.64E-07
RP11-303E	0.306469643	1.02E-08	4.77E-07
AC073043.	0.306433601	1.03E-08	4.77E-07
H2AFY	0.306363267	1.04E-08	4.80E-07
SRSF3	0.306127217	1.07E-08	4.92E-07
GMPS	0.306019785	1.08E-08	4.97E-07
ATAD5	0.305817608	1.10E-08	5.07E-07
GHDC	-0.305374639	1.16E-08	5.32E-07
AKAP5	0.304904632	1.23E-08	5.59E-07
MIEF2	-0.304901493	1.23E-08	5.59E-07
TCAM1P	0.304473296	1.29E-08	5.86E-07
PXMP2	0.3043725	1.30E-08	5.91E-07
ARNTL2	0.304242532	1.32E-08	5.99E-07
CDH24	0.302971951	1.53E-08	6.90E-07
TFDP1	0.302960749	1.53E-08	6.90E-07
AC005229.	0.302887739	1.55E-08	6.94E-07
TOLLIP-AS1	-0.302392746	1.64E-08	7.32E-07
SKA2	0.302351518	1.64E-08	7.33E-07
PRSS1	0.302335346	1.65E-08	7.33E-07
TMEM38B	0.302157357	1.68E-08	7.46E-07
NR1D1	-0.301098963	1.89E-08	8.39E-07
C1orf116	-0.300755609	1.97E-08	8.70E-07
BUB3	0.300721279	1.98E-08	8.72E-07
PHTF2	0.300490963	2.03E-08	8.92E-07
MPHOSPH9	0.300455696	2.04E-08	8.94E-07
NATD1	-0.300429397	2.04E-08	8.94E-07
HAUS8	0.300316848	2.07E-08	9.03E-07
FMOD	-0.300226232	2.09E-08	9.10E-07
CDKN2A	0.299650418	2.23E-08	9.69E-07
RP11-381E	0.299432255	2.29E-08	9.91E-07
VPS11	-0.299183656	2.35E-08	1.02E-06
TFRC	0.298160188	2.64E-08	1.14E-06
METTL21B	-0.297647266	2.79E-08	1.20E-06
CKAP5	0.297111969	2.97E-08	1.27E-06
RELT	0.296931062	3.03E-08	1.29E-06
RB1	-0.29692198	3.03E-08	1.29E-06
SAE1	0.296915542	3.03E-08	1.29E-06
RP11-546D	0.29682651	3.06E-08	1.30E-06
KLHL23	0.296731094	3.10E-08	1.31E-06
CTC-453G2	0.296395661	3.21E-08	1.36E-06
H2AFX	0.296012143	3.35E-08	1.41E-06
LINC01529	0.29589502	3.40E-08	1.43E-06
HNRNPR	0.295727297	3.46E-08	1.45E-06
TPRG1L	-0.295648026	3.49E-08	1.46E-06

SLC35B4	0.295636502	3.50E-08	1.46E-06
MIR17HG	0.295031322	3.74E-08	1.56E-06
MMP24-AS	-0.294987604	3.76E-08	1.56E-06
SNX21	-0.294964998	3.77E-08	1.56E-06
TMEM237	0.294598216	3.92E-08	1.62E-06
INTS7	0.294548369	3.94E-08	1.63E-06
LBR	0.294389538	4.01E-08	1.65E-06
TBC1D31	0.294292019	4.06E-08	1.67E-06
MMS22L	0.294152975	4.12E-08	1.69E-06
ZNF282	0.294000138	4.19E-08	1.71E-06
FAM136A	0.293161903	4.60E-08	1.88E-06
RP11-541N	-0.293062403	4.65E-08	1.89E-06
DHFRL1	-0.292900378	4.73E-08	1.92E-06
MSLN	-0.292696484	4.84E-08	1.96E-06
RP11-649A	0.292445303	4.97E-08	2.01E-06
RP11-726G	0.292424722	4.98E-08	2.01E-06
UBA52P6	0.291746022	5.37E-08	2.16E-06
CHST15	-0.291240286	5.67E-08	2.28E-06
POC1A	0.290967305	5.85E-08	2.34E-06
RP4-694A7	0.290685885	6.03E-08	2.41E-06
RP11-383F	0.290676477	6.03E-08	2.41E-06
RP11-148K	0.290532726	6.13E-08	2.44E-06
BICDL2	-0.290489484	6.16E-08	2.45E-06
TOLLIP	-0.29015301	6.39E-08	2.53E-06
NEMP1	0.289975685	6.51E-08	2.57E-06
RP11-244M	0.289891175	6.57E-08	2.59E-06
SIRT3	-0.289852873	6.60E-08	2.60E-06
ARL4D	-0.289827942	6.62E-08	2.60E-06
FBXO45	0.289772243	6.66E-08	2.61E-06
HNRNPAB	0.2897312	6.69E-08	2.61E-06
HNRNPM	0.289543712	6.82E-08	2.66E-06
NCAPD2	0.289219964	7.07E-08	2.75E-06
TTF2	0.289023845	7.22E-08	2.80E-06
AUNIP	0.287899134	8.15E-08	3.16E-06
PLLP	-0.287190824	8.80E-08	3.40E-06
MIS18BP1	0.286883031	9.09E-08	3.51E-06
GCH1	0.286730677	9.24E-08	3.56E-06
RP5-1121A	0.286411615	9.57E-08	3.67E-06
ITGB3BP	0.286363055	9.62E-08	3.69E-06
HAUS5	0.28618623	9.80E-08	3.75E-06
SRSF7	0.286139218	9.85E-08	3.75E-06
DUTP2	0.286120594	9.87E-08	3.75E-06
LLNLR-276I	0.286114608	9.88E-08	3.75E-06
FAM63A	-0.285987182	1.00E-07	3.80E-06
MIR25	0.28571319	1.03E-07	3.90E-06
TIPIN	0.285631253	1.04E-07	3.93E-06
RP11-968A	0.285551511	1.05E-07	3.95E-06

KRBA1	0.285148249	1.09E-07	4.12E-06
MAP6D1	0.285093821	1.10E-07	4.13E-06
DLEU2	0.284730345	1.14E-07	4.29E-06
NRM	0.284146343	1.22E-07	4.55E-06
KCNK15	-0.283894031	1.25E-07	4.66E-06
Y_RNA.476	0.283851071	1.26E-07	4.68E-06
RP5-1074L	0.283491717	1.31E-07	4.85E-06
PLPP2	-0.283341839	1.33E-07	4.91E-06
CRTAP	-0.283112535	1.36E-07	5.02E-06
EXOG	0.282997995	1.38E-07	5.07E-06
BRAF	0.282788085	1.41E-07	5.18E-06
ZNF695	0.282423276	1.46E-07	5.37E-06
SNX33	-0.282351803	1.47E-07	5.40E-06
RRM1	0.282274365	1.49E-07	5.43E-06
MTF2	0.282128994	1.51E-07	5.50E-06
ZNF174	-0.281891808	1.55E-07	5.63E-06
POLD1	0.28144493	1.62E-07	5.89E-06
DDX11	0.281037848	1.69E-07	6.13E-06
RBM28	0.281022321	1.69E-07	6.13E-06
LMBRD1	-0.281008814	1.70E-07	6.13E-06
LMNB2	0.280990544	1.70E-07	6.13E-06
RPP21	0.280914643	1.71E-07	6.16E-06
HDAC5	-0.280652226	1.76E-07	6.32E-06
NPRL3	-0.280580643	1.77E-07	6.35E-06
ZNF783	0.280555016	1.78E-07	6.35E-06
TLR5	-0.280551107	1.78E-07	6.35E-06
LTBP3	-0.280466198	1.80E-07	6.40E-06
TMEM25	-0.280303351	1.83E-07	6.49E-06
CAMK2N2	0.28027712	1.83E-07	6.50E-06
MCM5	0.280235359	1.84E-07	6.51E-06
CBX2	0.280218617	1.84E-07	6.51E-06
HYLS1	0.280112117	1.86E-07	6.57E-06
MAD2L1	0.279930353	1.90E-07	6.68E-06
CRY2	-0.279906739	1.90E-07	6.69E-06
HERC2P10	0.2797665	1.93E-07	6.77E-06
DTYMK	0.279626311	1.96E-07	6.86E-06
TRIM24	0.279361268	2.02E-07	7.04E-06
SLFN11	0.279337258	2.02E-07	7.04E-06
NPDC1	-0.279319126	2.02E-07	7.04E-06
GSG2	0.279304981	2.03E-07	7.04E-06
VPS51	-0.279164626	2.06E-07	7.13E-06
KIAA1147	0.279079918	2.08E-07	7.17E-06
ZNF319	-0.279069068	2.08E-07	7.17E-06
RMI2	0.279041362	2.08E-07	7.17E-06
CTIF	-0.278518931	2.20E-07	7.56E-06
LIG1	0.278403496	2.23E-07	7.64E-06
TMEM150A	-0.278290036	2.25E-07	7.71E-06

NMU	0.278256744	2.26E-07	7.72E-06
MUC5B	-0.277940606	2.34E-07	7.97E-06
PTPRG-AS1	0.277783111	2.37E-07	8.08E-06
NDC1	0.277743894	2.38E-07	8.10E-06
ITGB1BP2	0.277605425	2.42E-07	8.20E-06
ACYP1	0.277023169	2.57E-07	8.69E-06
PIGB	-0.276737517	2.65E-07	8.93E-06
DISP1	-0.276687979	2.66E-07	8.95E-06
SAPCD2	0.276687108	2.66E-07	8.95E-06
SEC31A	-0.275926566	2.88E-07	9.66E-06
RIF1	0.275801646	2.91E-07	9.76E-06
C3orf52	0.275667748	2.95E-07	9.88E-06
CENPH	0.275598884	2.97E-07	9.93E-06
RFWD3	0.275375276	3.04E-07	1.01E-05
CALCOCO1	-0.274974805	3.17E-07	1.05E-05
MYO19	0.274900447	3.20E-07	1.06E-05
FAM175A	-0.274886645	3.20E-07	1.06E-05
AC114776.	0.27487098	3.21E-07	1.06E-05
RIPPLY3	0.274637205	3.28E-07	1.08E-05
POLA1	0.274617379	3.29E-07	1.08E-05
HS1BP3	-0.274595522	3.30E-07	1.08E-05
HMSD	0.274508642	3.33E-07	1.09E-05
RP11-485G	0.274140015	3.45E-07	1.13E-05
DEAF1	-0.273899246	3.54E-07	1.16E-05
ACSS3	-0.27389217	3.54E-07	1.16E-05
RBM15	0.273848527	3.56E-07	1.16E-05
SLC20A1	0.273729598	3.60E-07	1.17E-05
PLCD3	-0.273648456	3.63E-07	1.18E-05
POLD3	0.273593103	3.65E-07	1.18E-05
COCH	0.273456226	3.70E-07	1.20E-05
SNRPA1	0.273063925	3.85E-07	1.24E-05
MBNL2	-0.272995432	3.88E-07	1.25E-05
MB21D1	0.272897581	3.92E-07	1.26E-05
C21orf58	0.272864108	3.93E-07	1.26E-05
BAZ1B	0.272673826	4.01E-07	1.29E-05
OSBPL5	-0.272480013	4.09E-07	1.31E-05
PIGX	0.271992724	4.30E-07	1.37E-05
RP11-376O	0.271928988	4.32E-07	1.38E-05
PHGDH	0.271504044	4.51E-07	1.44E-05
THAP5P1	0.271359579	4.58E-07	1.45E-05
ACAT2	0.27135758	4.58E-07	1.45E-05
NUP153	0.271215991	4.65E-07	1.47E-05
SLC25A40	0.271182049	4.66E-07	1.47E-05
SRSF4	0.270826523	4.83E-07	1.52E-05
IL17RB	0.270739531	4.88E-07	1.53E-05
ABTB1	-0.270679451	4.91E-07	1.54E-05
PAFAH2	-0.270670737	4.91E-07	1.54E-05

NUP210	0.270573348	4.96E-07	1.55E-05
LRP8	0.270502745	4.99E-07	1.56E-05
CCDC115	-0.270432593	5.03E-07	1.57E-05
KPNA4	0.270358367	5.07E-07	1.58E-05
BRI3BP	0.270047328	5.23E-07	1.63E-05
KMT5A	0.269991245	5.26E-07	1.63E-05
MTHFD2	0.269819581	5.35E-07	1.66E-05
PTPRU	-0.269769502	5.38E-07	1.66E-05
ATP6V0E2-	0.269739483	5.39E-07	1.66E-05
EPB41L1	-0.269577901	5.48E-07	1.69E-05
GHET1	0.269438134	5.56E-07	1.71E-05
AC005624.	0.269360237	5.60E-07	1.72E-05
RP11-773H	0.269282221	5.65E-07	1.73E-05
CNIH2	0.269226711	5.68E-07	1.74E-05
RP11-744I2	0.269108031	5.74E-07	1.75E-05
PCK2	0.269069611	5.77E-07	1.76E-05
ZGRF1	0.268357182	6.19E-07	1.88E-05
RP11-507K	-0.267996492	6.42E-07	1.95E-05
ITPRIPL1	0.267677369	6.63E-07	2.01E-05
GLMN	0.267313216	6.87E-07	2.08E-05
RN7SL521P	0.2666012	7.37E-07	2.23E-05
ZNF530	0.266599294	7.37E-07	2.23E-05
CASP8AP2	0.266456013	7.48E-07	2.25E-05
UBL3	-0.26638681	7.53E-07	2.26E-05
RP4-794I6.	-0.26637144	7.54E-07	2.26E-05
CTD-3157E	-0.266280111	7.61E-07	2.28E-05
PCGF6	0.266115622	7.74E-07	2.31E-05
CAPS	-0.266112175	7.74E-07	2.31E-05
CCND1	-0.265983278	7.84E-07	2.34E-05
C11orf68	-0.265888967	7.91E-07	2.35E-05
MFN1	0.265872692	7.92E-07	2.35E-05
NUP107	0.265860773	7.93E-07	2.35E-05
CD81	-0.265855366	7.94E-07	2.35E-05
ZNF724P	0.265767156	8.01E-07	2.37E-05
C17orf107	-0.265756149	8.02E-07	2.37E-05
PNPT1	0.265413192	8.29E-07	2.44E-05
DCLRE1C	0.265384579	8.32E-07	2.45E-05
MSH6	0.265191037	8.48E-07	2.49E-05
PLD2	-0.265073194	8.57E-07	2.51E-05
RIBC2	0.264826198	8.79E-07	2.57E-05
PARP12	0.264392015	9.17E-07	2.68E-05
RP11-1023	0.264322289	9.23E-07	2.69E-05
SIDT2	-0.264213756	9.33E-07	2.71E-05
TDP1	0.264209031	9.33E-07	2.71E-05
HNRNPL	0.264171081	9.37E-07	2.72E-05
IFRD1	0.264096019	9.44E-07	2.74E-05
SNORA70.3	0.263915138	9.61E-07	2.78E-05

AC027601.	0.263804077	9.71E-07	2.81E-05
PCMTD1	-0.263764541	9.75E-07	2.81E-05
ZC3HC1	0.263624875	9.88E-07	2.85E-05
SCML1	0.263319093	1.02E-06	2.92E-05
DDIAS	0.263315725	1.02E-06	2.92E-05
SP2-AS1	-0.263203325	1.03E-06	2.95E-05
ANP32B	0.26316235	1.03E-06	2.96E-05
RPL13	-0.262677079	1.08E-06	3.10E-05
SAP30	0.262470514	1.11E-06	3.15E-05
CSTF2	0.262293354	1.13E-06	3.20E-05
FAF1	0.262030281	1.15E-06	3.28E-05
IL37	0.261723117	1.19E-06	3.38E-05
NUP155	0.261691639	1.19E-06	3.38E-05
FANCB	0.261618764	1.20E-06	3.40E-05
CDKN2C	0.261551191	1.21E-06	3.42E-05
UGT8	0.261507583	1.21E-06	3.43E-05
HSPA14	0.261190642	1.25E-06	3.53E-05
CCDC14	0.260979362	1.28E-06	3.59E-05
ABHD3	0.260865564	1.29E-06	3.63E-05
RP11-67L1	0.260842132	1.30E-06	3.63E-05
TXNIP	-0.260812049	1.30E-06	3.63E-05
ST5	-0.260604062	1.33E-06	3.70E-05
BORCS6	-0.260424159	1.35E-06	3.76E-05
AMMECR1	0.260237766	1.37E-06	3.82E-05
PDSS1	0.260230205	1.37E-06	3.82E-05
C21orf62-A	-0.260179267	1.38E-06	3.83E-05
C4orf46	0.26014029	1.39E-06	3.84E-05
CDK18	-0.260139869	1.39E-06	3.84E-05
FAM111A	0.260035339	1.40E-06	3.87E-05
HNRNPH1	0.259817083	1.43E-06	3.94E-05
RNASEL	-0.259725755	1.44E-06	3.97E-05
ZBTB7A	-0.259575242	1.46E-06	4.03E-05
BAALC-AS1	-0.259418882	1.49E-06	4.08E-05
ETF1P2	0.259325414	1.50E-06	4.11E-05
RDM1	0.259260458	1.51E-06	4.13E-05
RNH1	-0.259140256	1.53E-06	4.17E-05
SYCE2	0.25905162	1.54E-06	4.20E-05
ORAI3	-0.259031068	1.54E-06	4.20E-05
CAPN2	-0.259025109	1.54E-06	4.20E-05
DLEU2L	0.258903603	1.56E-06	4.24E-05
SLF1	0.258799769	1.58E-06	4.28E-05
SDS	0.258643671	1.60E-06	4.33E-05
ARNTL	-0.258359947	1.65E-06	4.45E-05
RP11-47A8	-0.258230932	1.67E-06	4.50E-05
TNPO3	0.258047117	1.70E-06	4.57E-05
ABHD14B	-0.257545074	1.78E-06	4.79E-05
CNTRL	0.257516739	1.78E-06	4.79E-05

BET1L	-0.257432972	1.80E-06	4.82E-05
TMC4	-0.257415637	1.80E-06	4.82E-05
AC079753.	0.25729936	1.82E-06	4.86E-05
ZBTB47	-0.25729682	1.82E-06	4.86E-05
TMEM106C	0.257248986	1.83E-06	4.88E-05
TMUB1	0.257154921	1.85E-06	4.91E-05
TRIM8	-0.257047209	1.87E-06	4.95E-05
TK2	-0.257040788	1.87E-06	4.95E-05
TMSB15A	0.256881823	1.89E-06	5.02E-05
HTRA1	-0.25685266	1.90E-06	5.03E-05
FAM161B	-0.256760986	1.92E-06	5.06E-05
CCNE1	0.256708811	1.93E-06	5.08E-05
EIF4B	-0.256581254	1.95E-06	5.14E-05
RP11-133K	0.256540171	1.96E-06	5.15E-05
CEBPG	0.256488022	1.97E-06	5.17E-05
DHRS12	-0.256452419	1.97E-06	5.18E-05
RN7SL280P	0.256405026	1.98E-06	5.19E-05
RP11-491F	0.256205541	2.02E-06	5.28E-05
TPP1	-0.256171889	2.03E-06	5.29E-05
IQSEC1	-0.256105485	2.04E-06	5.32E-05
FAM172A	-0.255765923	2.11E-06	5.48E-05
SH3BGRL	-0.255698302	2.12E-06	5.51E-05
ZNF287	-0.255632939	2.13E-06	5.54E-05
NEURL1B	0.255613686	2.14E-06	5.54E-05
TAF5	0.255551343	2.15E-06	5.56E-05
LINC00664	0.255535194	2.15E-06	5.56E-05
MDC1	0.255513582	2.16E-06	5.57E-05
ZNF730	0.255281835	2.21E-06	5.68E-05
ZNF843	-0.255091201	2.25E-06	5.78E-05
RP11-85I17	0.254956548	2.27E-06	5.84E-05
BBS12	-0.254923094	2.28E-06	5.85E-05
PMAIP1	0.254722963	2.33E-06	5.95E-05
SCAMP2	-0.254638032	2.34E-06	5.99E-05
MLYCD	-0.25457387	2.36E-06	6.02E-05
PRKAR1B	-0.254506088	2.37E-06	6.05E-05
HSPB11	0.254131728	2.46E-06	6.26E-05
LIN9	0.253833554	2.53E-06	6.43E-05
NFIX	-0.253563262	2.59E-06	6.58E-05
PSMC3	0.253436413	2.62E-06	6.64E-05
TMEM56	0.253433237	2.62E-06	6.64E-05
WDFY3-AS2	-0.253375574	2.64E-06	6.67E-05
METTL2B	0.253317765	2.65E-06	6.70E-05
ARHGAP5-A	-0.253269555	2.67E-06	6.72E-05
CRIM1	-0.253188213	2.69E-06	6.76E-05
LRRC40	0.253124755	2.70E-06	6.79E-05
WBP1L	-0.253072568	2.72E-06	6.81E-05
SRPK1	0.252927727	2.75E-06	6.90E-05

ANKHD1	0.252909032	2.76E-06	6.90E-05
SMIM1	-0.252892078	2.76E-06	6.90E-05
CTD-2020K	-0.25277899	2.79E-06	6.96E-05
ZFYVE28	-0.252573166	2.85E-06	7.09E-05
FCRLB	-0.252424156	2.89E-06	7.18E-05
VSTM2L	-0.252245829	2.93E-06	7.29E-05
RBKS	-0.252153704	2.96E-06	7.34E-05
DPF1	0.25214567	2.96E-06	7.34E-05
STRADB	0.252039271	2.99E-06	7.40E-05
RNF138	0.251924057	3.02E-06	7.47E-05
SLC9A3R2	-0.251869121	3.04E-06	7.50E-05
AC009948.	0.251826885	3.05E-06	7.52E-05
CSRP2	0.25172048	3.08E-06	7.58E-05
TRIM29	-0.251690266	3.09E-06	7.59E-05
CDC25B	0.251652906	3.10E-06	7.61E-05
RNF168	0.251593127	3.12E-06	7.64E-05
ELOVL2	0.251438899	3.16E-06	7.74E-05
RP11-351C	0.251421907	3.17E-06	7.74E-05
EXOSC8	0.251197221	3.23E-06	7.89E-05
DNAJC27-A	-0.250954715	3.31E-06	8.06E-05
RBM17	0.250927632	3.32E-06	8.07E-05
RBBP4	0.250835425	3.35E-06	8.13E-05
RP11-599J1	0.250642152	3.41E-06	8.25E-05
TAPT1-AS1	-0.25061046	3.42E-06	8.25E-05
RPL39L	0.250609825	3.42E-06	8.25E-05
EIF3J-AS1	-0.250605553	3.42E-06	8.25E-05
SENP5	0.250597273	3.42E-06	8.25E-05
MYL9	-0.250422034	3.48E-06	8.38E-05
A4GALT	-0.25027586	3.52E-06	8.48E-05
CDKAL1	0.250236015	3.54E-06	8.50E-05
YBX1P4	0.250222391	3.54E-06	8.50E-05
RP11-468E	0.250171901	3.56E-06	8.53E-05
NRF1	0.250101092	3.58E-06	8.57E-05
CYBRD1	-0.250048705	3.60E-06	8.60E-05
LINC00842	-0.249955495	3.63E-06	8.66E-05
RP11-9E13	0.24991027	3.64E-06	8.69E-05
TMEM127	-0.249728689	3.71E-06	8.82E-05
EME1	0.249711476	3.71E-06	8.82E-05
MEIS3P1	-0.249574573	3.76E-06	8.93E-05
UBXN6	-0.249284972	3.86E-06	9.15E-05
AC133680.	0.24920773	3.89E-06	9.21E-05
RP11-121C	0.249025951	3.95E-06	9.35E-05
PIGA	0.249001282	3.96E-06	9.36E-05
TOMM5	0.248981771	3.97E-06	9.36E-05
ATP5G1P4	0.248915805	3.99E-06	9.41E-05
GJC2	-0.24881166	4.03E-06	9.49E-05
HELQ	-0.248743668	4.06E-06	9.53E-05

HNRNPA3	0.248500704	4.15E-06	9.73E-05
RNF208	-0.248177209	4.28E-06	0.00010015
RHBDF1	-0.248067346	4.32E-06	0.00010103
DDX11-AS1	0.24802422	4.34E-06	0.000101296
JADE3	0.247928656	4.37E-06	0.000102052
EPB41L4A-/	-0.247790404	4.43E-06	0.000103216
RP13-735L	0.247745104	4.45E-06	0.000103508
PEX12	-0.247725302	4.46E-06	0.000103559
MXI1	-0.247595685	4.51E-06	0.000104657
RP11-149I2	0.247503778	4.55E-06	0.000105402
CNNM2	-0.247220419	4.67E-06	0.000108027
PRKDC	0.247198854	4.68E-06	0.000108098
AC005534.	0.247126166	4.71E-06	0.000108676
HN1	0.247055943	4.74E-06	0.000109232
SYS1	-0.247012742	4.76E-06	0.00010952
UNG	0.246992723	4.76E-06	0.000109577
CCAR1	0.246958748	4.78E-06	0.000109774
SCLT1	0.246786319	4.86E-06	0.00011137
CEP192	0.246733722	4.88E-06	0.00011176
TRIM4	-0.246394682	5.03E-06	0.000115117
RP11-506M	0.246372142	5.04E-06	0.000115204
PKN3	0.24620298	5.12E-06	0.00011684
AC117490.	0.245983959	5.22E-06	0.000119037
BLCAP	-0.245835569	5.29E-06	0.000120498
SLC27A1	-0.245772744	5.32E-06	0.000121031
TBC1D7	0.245653279	5.38E-06	0.000122193
RAB11FIP5	-0.245535295	5.44E-06	0.00012335
NSMCE1	-0.245473846	5.47E-06	0.00012388
CHST12	-0.245448877	5.48E-06	0.000124001
AP000439.	-0.245141296	5.64E-06	0.000127344
EPN2	-0.245126577	5.65E-06	0.00012735
RP11-10L1	-0.245094235	5.66E-06	0.00012756
PRELID2	0.245005204	5.71E-06	0.000128264
RP4-533D7	0.244992854	5.71E-06	0.000128264
SLC16A6	0.244991145	5.72E-06	0.000128264
CHN1	0.244928559	5.75E-06	0.000128828
TTC13	0.244865653	5.78E-06	0.000129399
TTC26	0.244645328	5.90E-06	0.000131836
C7orf73	0.244547565	5.95E-06	0.00013281
WDFY3	-0.244535842	5.96E-06	0.000132809
CSDC2	-0.244363528	6.05E-06	0.000134722
MMP28	-0.244318605	6.07E-06	0.000135024
FLII	-0.244310734	6.08E-06	0.000135024
EFCAB3	0.244188495	6.15E-06	0.00013635
RP11-159N	-0.244131007	6.18E-06	0.000136886
BORCS7	-0.244094639	6.20E-06	0.000137163
RP11-564D	0.243974553	6.27E-06	0.000138482

SEPT14P12	0.243838473	6.34E-06	0.000140015
IGBP1	-0.243797151	6.37E-06	0.000140361
TCF25	-0.243775038	6.38E-06	0.000140465
MAGOH	0.243756378	6.39E-06	0.000140525
RP11-3K16	0.243606232	6.48E-06	0.00014226
UPF3B	0.243567058	6.50E-06	0.000142584
AC008982.	0.243405005	6.60E-06	0.000144498
LRRN4CL	-0.243191199	6.72E-06	0.000147119
DNAJB2	-0.242795622	6.97E-06	0.000152247
CX3CR1	-0.242753592	6.99E-06	0.000152633
CYB5D2	-0.242547885	7.12E-06	0.00015528
ARHGEF26	0.242507996	7.15E-06	0.000155595
ACOT7	0.242497636	7.15E-06	0.000155595
RP11-641D	0.242397492	7.22E-06	0.000156803
CTF1	-0.242280534	7.30E-06	0.000158258
ZNF492	0.242082946	7.43E-06	0.000160882
LINC00634	0.242061682	7.44E-06	0.00016099
NFATC2IP	0.242007862	7.48E-06	0.000161568
SNORA2B	0.24197557	7.50E-06	0.000161836
RP11-46C2	-0.24166404	7.71E-06	0.000166198
SLC4A2	0.241590461	7.76E-06	0.000167088
EXOSC3	0.241521142	7.81E-06	0.000167919
PTCD3	0.241364296	7.92E-06	0.000170077
RP5-1074L	0.241322295	7.95E-06	0.000170506
ODF2	0.241228678	8.01E-06	0.000171725
RP11-539I5	-0.241171627	8.05E-06	0.00017239
SPOCK2	-0.24087795	8.27E-06	0.000176741
RP11-46H1	-0.240840546	8.30E-06	0.000177115
ASAH1	-0.240706433	8.40E-06	0.000179022
STXBP6	0.240645488	8.44E-06	0.000179776
RCC1	0.240610081	8.47E-06	0.000180125
EID1	-0.240586511	8.48E-06	0.000180174
AC000068.	0.240579817	8.49E-06	0.000180174
PTBP2	0.240503286	8.55E-06	0.00018116
PSMD2	0.240491255	8.56E-06	0.00018116
GBGT1	-0.240420246	8.61E-06	0.000182087
EIF3L	-0.240260845	8.73E-06	0.000184459
NCBP1	0.240034526	8.91E-06	0.000187973
LONRF2	-0.239918338	9.00E-06	0.000189634
HIRA	0.239908181	9.01E-06	0.000189634
SH3GL2	0.239853302	9.06E-06	0.000190331
SRSF1	0.239762267	9.13E-06	0.000191642
CCHCR1	0.23971087	9.17E-06	0.000192286
ISPD	-0.239661418	9.21E-06	0.0001929
PKIG	-0.23962267	9.24E-06	0.000193332
UBR7	0.239377572	9.44E-06	0.000197333
RP11-568K	-0.239350287	9.47E-06	0.000197575

GKAP1	0.239152745	9.63E-06	0.000200815
DOK7	-0.239105126	9.67E-06	0.000201422
ZNF850	0.239079012	9.70E-06	0.000201648
SUSD2	-0.23904301	9.73E-06	0.000202051
TMEM102	-0.238761967	9.97E-06	0.000206878
EIF3F	-0.238668404	1.01E-05	0.000208181
PLEKHM1	-0.238663969	1.01E-05	0.000208181
LOXL4	-0.238604779	1.01E-05	0.000209024
RP11-831F	0.238558082	1.02E-05	0.000209639
IQCC	0.238466227	1.02E-05	0.000211094
CDK5	0.238411781	1.03E-05	0.000211859
RIN2	-0.238385835	1.03E-05	0.000212095
MBTPS1	-0.238279767	1.04E-05	0.000213834
DPH1	-0.238203783	1.05E-05	0.000215016
RNU1-68P	-0.238130053	1.05E-05	0.000216162
RP11-306G	0.237927034	1.07E-05	0.000219775
RP5-981O7	0.237914916	1.07E-05	0.000219775
SNHG8	-0.237897676	1.08E-05	0.000219852
FMO4	-0.23787281	1.08E-05	0.000220077
CH507-24F	0.23767062	1.10E-05	0.000223758
SPIN4	0.237412312	1.12E-05	0.000228618
VRK1	0.237265027	1.14E-05	0.000231319
MRPL47	0.237156363	1.15E-05	0.000233261
RP11-428C	0.237031167	1.16E-05	0.000235558
RPL35AP4	0.236757171	1.19E-05	0.000240953
STAM-AS1	0.236745813	1.19E-05	0.000240953
OVOL3	0.236726883	1.19E-05	0.000241075
ZNF789	0.236626328	1.20E-05	0.000242922
SUFU	-0.23652378	1.21E-05	0.000244712
TBCD	0.236516007	1.21E-05	0.000244712
LRP1	-0.236500326	1.22E-05	0.000244766
CC2D2A	-0.236330391	1.23E-05	0.000248139
LA16c-380/	-0.2362726	1.24E-05	0.000249108
KRT18P5	0.236137362	1.26E-05	0.000251774
CTA-384D8	0.235993547	1.27E-05	0.000254658
RFC3	0.235913531	1.28E-05	0.000256147
RAD54B	0.235874867	1.28E-05	0.000256698
CCP110	0.235862736	1.29E-05	0.000256698
C3orf18	-0.235734492	1.30E-05	0.000259284
XXbac-BPG	0.235681543	1.31E-05	0.000260186
RP1-199J3.	-0.235667006	1.31E-05	0.00026022
FANCD2OS	0.235627292	1.31E-05	0.000260751
CDKN2D	0.235617597	1.31E-05	0.000260751
CAB39P1	0.235505015	1.33E-05	0.000263018
S100PBP	0.235423627	1.34E-05	0.000264579
GRPEL2	0.235410981	1.34E-05	0.000264579
C4orf3	-0.235368727	1.34E-05	0.000265254

RP11-227D	0.235335803	1.35E-05	0.000265715
SCN2B	-0.235203199	1.36E-05	0.00026849
SLBP	0.235074423	1.38E-05	0.000271203
KNDC1	-0.234883298	1.40E-05	0.000275426
CREG1	-0.234855912	1.40E-05	0.000275664
DCTD	-0.234847547	1.40E-05	0.000275664
RNA5SP323	0.234817876	1.41E-05	0.000276065
RP11-741G	0.23476152	1.41E-05	0.000277107
WDR53	0.234696073	1.42E-05	0.000278373
RP4-584D1	0.234666971	1.43E-05	0.000278625
SOS1-IT1	0.234659954	1.43E-05	0.000278625
RBM33	0.234462027	1.45E-05	0.000283124
BOK	-0.234442949	1.45E-05	0.000283278
PID1	-0.234239162	1.48E-05	0.000287994
NDUFA6-AS	-0.234187019	1.49E-05	0.000288975
IL34	-0.233663666	1.56E-05	0.000301993
ABCA2	-0.233392489	1.59E-05	0.000308791
FCER1A	-0.233353938	1.60E-05	0.000309476
FNDC3A	-0.233279661	1.61E-05	0.000311118
MYB	0.23325536	1.61E-05	0.000311427
RP1-228P1	0.233139432	1.63E-05	0.000314203
PRRT3	-0.233044989	1.64E-05	0.000316418
MIR548AA1	0.23295999	1.65E-05	0.000318389
TVP23B	-0.23289876	1.66E-05	0.000319455
UBTD1	-0.232895609	1.66E-05	0.000319455
Y_RNA.125	0.232771892	1.68E-05	0.000322515
FBXL16	-0.232715646	1.69E-05	0.000323607
HLF	-0.232707138	1.69E-05	0.000323607
PLA2G3	0.232628761	1.70E-05	0.000325437
FAR2	0.232612799	1.70E-05	0.000325528
C19orf57	0.232596055	1.70E-05	0.000325642
SLC16A4	-0.232539341	1.71E-05	0.000326876
FAM122B	0.23252563	1.72E-05	0.000326906
UBN2	0.232459086	1.72E-05	0.000328159
NANOGP1	0.232443379	1.73E-05	0.000328159
RP11-135F	0.232443188	1.73E-05	0.000328159
DMTF1	0.232344217	1.74E-05	0.000330598
RP11-649A	0.232306072	1.75E-05	0.000331323
MTHFD1	0.232243454	1.76E-05	0.000332747
DOK4	-0.232210243	1.76E-05	0.000333336
AC072052.	-0.231963129	1.80E-05	0.000340097
FAM161A	0.231738551	1.83E-05	0.00034632
FUS	0.231712317	1.84E-05	0.000346725
CCDC85A	-0.231608706	1.86E-05	0.000349434
MIS18A	0.231446758	1.88E-05	0.000353921
CLUAP1	-0.231345888	1.90E-05	0.0003566
PCIF1	-0.231326822	1.90E-05	0.000356799

ITGA3	-0.231302194	1.90E-05	0.000357168
CHID1	-0.231204563	1.92E-05	0.000359771
RPL3	-0.231173345	1.93E-05	0.000360347
HNRNPK	0.231097908	1.94E-05	0.000362286
AES	-0.230946105	1.96E-05	0.000366613
ASS1P12	0.230889517	1.97E-05	0.000367993
TOM1	-0.230862828	1.98E-05	0.000368363
DTX4	-0.230852856	1.98E-05	0.000368363
CENPQ	0.230832511	1.98E-05	0.000368611
RNF157	0.230780384	1.99E-05	0.00036986
RPL7	-0.230727705	2.00E-05	0.00037113
MAP2K3	-0.230688641	2.01E-05	0.000371973
NUDT18	-0.230497858	2.04E-05	0.000377659
PPP1R3D	-0.230351093	2.06E-05	0.000381997
ACADSB	-0.230313075	2.07E-05	0.00038283
ZNF567	0.230175466	2.10E-05	0.000386923
RNU6-875F	0.230152495	2.10E-05	0.000387272
MRS2	0.230095128	2.11E-05	0.000388754
NRDC	0.229781108	2.17E-05	0.00039865
KIAA0226L	-0.229774037	2.17E-05	0.00039865
CLU	-0.229724566	2.18E-05	0.000399906
CREBRF	-0.22970617	2.18E-05	0.000400112
CTD-3157E	-0.229589629	2.20E-05	0.00040366
BRICD5	0.229495919	2.22E-05	0.000406451
C1RL	-0.22945288	2.23E-05	0.00040751
HIST1H2BJ	0.229428646	2.23E-05	0.000407754
KDM5B	-0.229421266	2.23E-05	0.000407754
GLTSCR2	-0.229362891	2.25E-05	0.000409348
CTC-260E6	0.229288508	2.26E-05	0.000411504
RP11-617F	0.229238492	2.27E-05	0.000412464
PHLDA3	-0.229225555	2.27E-05	0.000412464
HCFC1R1	-0.229217263	2.27E-05	0.000412464
AMOTL2	-0.229212067	2.27E-05	0.000412464
RP5-1021I2	-0.229137852	2.29E-05	0.00041432
RP11-335L	-0.229134581	2.29E-05	0.00041432
RPL21P7	0.229118621	2.29E-05	0.000414453
CTD-2267D	-0.228821537	2.35E-05	0.000424544
PIP5K1C	-0.228774886	2.36E-05	0.000425781
RIPPLY1	0.228567246	2.40E-05	0.000432557
MVP	-0.228563336	2.40E-05	0.000432557
CXorf65	0.228536514	2.41E-05	0.000433092
RP11-531F	0.228495914	2.42E-05	0.000433428
CAMLG	-0.228493209	2.42E-05	0.000433428
SARAF	-0.228490975	2.42E-05	0.000433428
RP11-54O7	0.228410887	2.43E-05	0.000435918
RNF224	-0.228313215	2.45E-05	0.00043907
NCAPD3	0.228257293	2.46E-05	0.000440449

ILF3	0.228251824	2.47E-05	0.000440449
RP11-315D	0.228192448	2.48E-05	0.000442206
AFF1	-0.228168628	2.48E-05	0.000442645
TPT1	-0.228022841	2.51E-05	0.000447652
HDAC11	-0.227989881	2.52E-05	0.000448193
LRRC75A-A	-0.227984417	2.52E-05	0.000448193
PLEKHB1	-0.227918454	2.54E-05	0.00045023
RP11-260M	0.22784542	2.55E-05	0.000452545
RP4-751H1	0.227786825	2.56E-05	0.000454088
CLIC3	-0.227780905	2.57E-05	0.000454088
SMC1A	0.227627313	2.60E-05	0.00045952
BRCA1	0.227543276	2.62E-05	0.000462307
CMIP	-0.227486508	2.63E-05	0.000463901
PLAC1	0.227475532	2.63E-05	0.000463901
THRA	-0.227464891	2.63E-05	0.000463901
KCNH2	0.227454461	2.64E-05	0.000463901
FNDC11	-0.227367012	2.66E-05	0.000466848
C16orf59	0.227354431	2.66E-05	0.000466874
TTC19	-0.227328966	2.66E-05	0.000467405
SRSF11	0.227302319	2.67E-05	0.000467984
GJC1	0.227287727	2.67E-05	0.00046809
CTD-2291D	0.22697841	2.74E-05	0.00047973
IMPA2	0.226970691	2.75E-05	0.000479729
CEP41	0.226925587	2.76E-05	0.000481066
GAA	-0.226785939	2.79E-05	0.000486235
OTULIN	0.22676168	2.79E-05	0.00048674
ALKBH8	-0.226747509	2.80E-05	0.000486835
RP11-197M	0.226717263	2.80E-05	0.000487585
LIX1L	-0.226605365	2.83E-05	0.000491322
RP11-757F	0.226602271	2.83E-05	0.000491322
HAT1	0.226534054	2.85E-05	0.000493643
MAGOHB	0.226411412	2.88E-05	0.000498233
NUDT9	-0.226394549	2.88E-05	0.000498444
ANAPC7	0.22632517	2.90E-05	0.000500846
IFT140	-0.226246799	2.92E-05	0.000503637
ARHGAP12	-0.226233059	2.92E-05	0.00050372
PSMA4	0.226139153	2.94E-05	0.000507183
NLRC5	0.226124023	2.95E-05	0.000507326
SOWAHB	-0.226107504	2.95E-05	0.000507529
SIAE	-0.225925629	3.00E-05	0.000514776
SMS	0.225871119	3.01E-05	0.000516613
GBP1	0.22583948	3.02E-05	0.000517471
ANXA2	-0.225805015	3.03E-05	0.000518308
MFI2-AS1	0.22579668	3.03E-05	0.000518308
APOBEC3B	0.225697456	3.05E-05	0.000521577
RN7SL569P	0.225693735	3.05E-05	0.000521577
CHIC1	-0.225686121	3.06E-05	0.000521577

SAC3D1	0.225668741	3.06E-05	0.000521826
CAPN1	-0.225509247	3.10E-05	0.000528282
RP11-573D	0.225450351	3.12E-05	0.000530361
RPL4	-0.225305994	3.15E-05	0.000536242
VPS41	-0.225203045	3.18E-05	0.000540324
HESX1	0.225112389	3.21E-05	0.00054388
RNU6-583F	0.224975333	3.24E-05	0.00054957
KB-1440D3	0.224904354	3.26E-05	0.000551954
FLJ20021	-0.224899912	3.26E-05	0.000551954
FAM83G	-0.224805485	3.29E-05	0.000555757
DPY19L2P1	0.224743711	3.31E-05	0.000558073
HES4	0.224536523	3.36E-05	0.000567186
IDH2	0.224494645	3.37E-05	0.00056861
TRAF3IP2	-0.224455534	3.39E-05	0.000569908
IQCB1	0.224381366	3.41E-05	0.000572624
LYRM9	-0.224374945	3.41E-05	0.000572624
AC007204.	0.224332352	3.42E-05	0.000574097
CENPJ	0.224217456	3.45E-05	0.000579023
LHPP	-0.22408091	3.49E-05	0.00058486
CASP4	-0.224073021	3.49E-05	0.00058486
CTD-2339L	0.223971977	3.52E-05	0.000588949
PARVA	-0.223965635	3.53E-05	0.000588949
OPN1SW	0.223930886	3.54E-05	0.00059008
FLYWCH1	-0.223918186	3.54E-05	0.000590139
RNFT2	0.22386755	3.55E-05	0.00059205
PRNP	-0.223705386	3.60E-05	0.000598988
TPMT	0.223703375	3.60E-05	0.000598988
CIRBP	-0.223680744	3.61E-05	0.00059954
RP1-127H1	0.223555975	3.65E-05	0.000604981
CASC4	-0.223548262	3.65E-05	0.000604981
EXOSC9	0.223418382	3.69E-05	0.000610913
KIAA1211L	-0.223198657	3.76E-05	0.000621478
USP18	0.223153408	3.77E-05	0.000623209
ALKBH6	0.223114905	3.78E-05	0.000624599
UBXN2A	0.222933966	3.84E-05	0.00063286
PROZ	0.222932244	3.84E-05	0.00063286
UHRF2	0.222876999	3.86E-05	0.000635144
AC092159.	0.222722623	3.90E-05	0.000642647
FBXO44	-0.222621278	3.94E-05	0.000647202
MIR519A2	0.222613782	3.94E-05	0.000647203
UCP3	0.222600084	3.94E-05	0.000647326
IVD	-0.222544451	3.96E-05	0.000649681
ALDH3B1	-0.222489675	3.98E-05	0.000651999
RP11-1334	0.222437037	4.00E-05	0.00065421
CTD-2002J	0.222355562	4.02E-05	0.000657982
TMUB2	-0.222298679	4.04E-05	0.000660443
EP400NL	0.222283747	4.05E-05	0.000660638

LINC00847	-0.22221695	4.07E-05	0.000663648
KMT2C	0.222191359	4.08E-05	0.000664424
MANBA	-0.222145417	4.09E-05	0.000665708
RP11-22B2	0.222145199	4.09E-05	0.000665708
TMEM255A	-0.222068241	4.12E-05	0.000669297
C16orf86	-0.221895495	4.18E-05	0.000678198
SBF2	-0.221815464	4.21E-05	0.000681542
HIST1H4C	0.221802533	4.21E-05	0.000681542
ZNF131	0.221801549	4.21E-05	0.000681542
MFSD14B	0.221586822	4.29E-05	0.000692969
PTGER2	-0.221561678	4.29E-05	0.000693755
LINC00921	-0.221446244	4.34E-05	0.000699682
IL6ST	-0.2211477	4.44E-05	0.000716273
MARS	0.22109521	4.46E-05	0.000718685
CNTN1	-0.220976755	4.50E-05	0.00072499
CLCN3	-0.220919667	4.53E-05	0.000727703
AC079922.	0.220872481	4.54E-05	0.000729837
RNU6-558P	0.220859083	4.55E-05	0.000729967
EPS8L2	-0.220820797	4.56E-05	0.000731578
ST13	-0.220617503	4.64E-05	0.000743101
BRE	-0.220605502	4.64E-05	0.000743149
MIR130B	0.220566391	4.66E-05	0.000744838
CCDC137	0.220454358	4.70E-05	0.000750969
GPM6B	0.220369866	4.73E-05	0.00075492
HMGB1	0.22036743	4.73E-05	0.00075492
TMEM209	0.220262781	4.77E-05	0.000760105
ACOX2	-0.220260832	4.77E-05	0.000760105
RP11-41O4	0.220248333	4.78E-05	0.000760188
BTRC	-0.220188974	4.80E-05	0.00076317
HSPB2	-0.220146222	4.82E-05	0.000765131
JUP	-0.220125885	4.83E-05	0.000765703
C1orf56	-0.220104114	4.84E-05	0.000765925
CLN6	0.220091956	4.84E-05	0.000765925
FBXO2	-0.220089013	4.84E-05	0.000765925
RAD1	0.220043836	4.86E-05	0.000768047
RP11-77K1	-0.219930283	4.90E-05	0.000774455
RNF141	-0.219770258	4.97E-05	0.000783859
C11orf71	-0.219729163	4.98E-05	0.000785768
OR2W3	-0.219697758	5.00E-05	0.000787064
PRPF38B	0.21962675	5.03E-05	0.000790893
HSD11B1L	-0.219604502	5.04E-05	0.000791611
TUBA1C	0.219571134	5.05E-05	0.000793043
ZFP2	-0.219465638	5.09E-05	0.000799128
RP11-170K	-0.219399685	5.12E-05	0.000802686
MAPK3	-0.21932477	5.15E-05	0.000806843
FRY	-0.219280658	5.17E-05	0.000809005
LRRC45	0.219215762	5.20E-05	0.000812536

ASIC2	-0.219160161	5.22E-05	0.000815469
DNAJC4	-0.219148665	5.22E-05	0.000815502
TLE2	-0.21904863	5.27E-05	0.000821388
HRH1	-0.218911962	5.32E-05	0.000829763
SUV39H1	0.218802779	5.37E-05	0.000836362
OSER1-AS1	-0.218707902	5.41E-05	0.000841555
APBB1	-0.218704086	5.41E-05	0.000841555
EFEMP2	-0.218644176	5.44E-05	0.000844215
FITM2	-0.218643023	5.44E-05	0.000844215
GYPC	-0.218629001	5.45E-05	0.000844425
PGBD1	0.218587563	5.47E-05	0.000846503
TUBGCP3	0.218567348	5.47E-05	0.000847137
GGH	0.218471688	5.52E-05	0.000852371
SLC26A6	0.218469055	5.52E-05	0.000852371
MIR613	0.218218098	5.63E-05	0.000868997
FLT3	0.218107374	5.68E-05	0.000875938
ZNF726	0.218097356	5.69E-05	0.000875938
RP11-481H	-0.218064335	5.70E-05	0.000877498
IGIP	-0.218040547	5.71E-05	0.000878409
AP1S3	0.217984656	5.74E-05	0.000881592
ZNF25	-0.217957987	5.75E-05	0.000882451
POLR2D	0.217950803	5.75E-05	0.000882451
TBC1D22A	-0.217879139	5.79E-05	0.000886327
RP13-554M	0.217861807	5.79E-05	0.000886327
RN7SL559P	0.217853092	5.80E-05	0.000886327
YPEL3	-0.217852891	5.80E-05	0.000886327
CARMIL2	0.217797378	5.82E-05	0.000888773
AC067945.	0.217796939	5.82E-05	0.000888773
ZNF19	-0.217696666	5.87E-05	0.000895178
SENP1	0.217516592	5.96E-05	0.000907414
GATC	0.217455999	5.99E-05	0.000911043
LAYN	-0.217436333	6.00E-05	0.000911692
ARL6IP5	-0.217321578	6.05E-05	0.000919318
YEATS2-AS	0.217257356	6.08E-05	0.000923261
PI4K2B	0.217239929	6.09E-05	0.000923484
ZNF365	-0.217232809	6.09E-05	0.000923484
LRRCC1	0.217158705	6.13E-05	0.000928179
BNIP3P17	0.217114937	6.15E-05	0.000929844
ZNF682	0.21711483	6.15E-05	0.000929844
HSD17B1	-0.217056315	6.18E-05	0.000933406
IL18	-0.217018333	6.20E-05	0.000935443
ZFPM2	-0.216991622	6.21E-05	0.00093664
STUB1	-0.216920152	6.25E-05	0.000941202
RP11-195F	-0.216909383	6.25E-05	0.000941207
CTD-2517C	-0.216843765	6.29E-05	0.000945348
WFS1	-0.216796265	6.31E-05	0.000948133
BTD	-0.216773871	6.32E-05	0.00094902

CDIPT	-0.21671909	6.35E-05	0.00095237
Y_RNA.55	0.216708342	6.36E-05	0.000952376
FBXO31	-0.216652881	6.38E-05	0.00095579
WSB2	0.216628096	6.40E-05	0.000956869
SNRPA	0.216571853	6.42E-05	0.000960091
MXD4	-0.216564695	6.43E-05	0.000960091
NORAD	-0.216538083	6.44E-05	0.000961317
RP11-322D	0.216435535	6.50E-05	0.000968394
FBXL22	-0.216390215	6.52E-05	0.000971078
CCNDBP1	-0.216372594	6.53E-05	0.000971621
NRARP	0.216324319	6.55E-05	0.000974544
RP11-771K	0.216276589	6.58E-05	0.000977433
SORBS2	-0.216250483	6.59E-05	0.000978642
SLCO4C1	0.216150951	6.64E-05	0.000985117
ADPRM	-0.216146569	6.65E-05	0.000985117
EEF1A1	-0.216119425	6.66E-05	0.000985283
TNFAIP8L1	0.216118255	6.66E-05	0.000985283
RP11-849H	0.216112771	6.66E-05	0.000985283
HAUS1	0.216034875	6.71E-05	0.000990189
CSTF3	0.216025509	6.71E-05	0.000990189
RP11-551L	0.216018774	6.71E-05	0.000990189
RP11-474I1	-0.215998085	6.73E-05	0.000990602
ATP2A1-AS	0.2159925	6.73E-05	0.000990602
PEX11B	-0.215942118	6.76E-05	0.000993751
PHTF1	0.215915735	6.77E-05	0.000995008
MOV10	0.215849626	6.81E-05	0.000999421
TOP3A	0.215765969	6.85E-05	0.001005254
ZNF114	0.215701731	6.89E-05	0.00100956
ZFP82	0.215690096	6.89E-05	0.001009653
SF3A3	0.215635323	6.92E-05	0.001013215
RP11-697E	0.215526806	6.98E-05	0.00102114
PNP	0.215401379	7.05E-05	0.001030505
WNT10A	-0.215378883	7.07E-05	0.001031491
LINC00887	-0.21535531	7.08E-05	0.001032566
NEIL1	-0.215323766	7.10E-05	0.00103401
RP11-542K	0.215316826	7.10E-05	0.00103401
GNB4	0.215240103	7.14E-05	0.001038969
JADE2	-0.215224754	7.15E-05	0.001038969
RP11-13N1	0.215218501	7.16E-05	0.001038969
BZW1	0.215214861	7.16E-05	0.001038969
Y_RNA.493	0.215162974	7.19E-05	0.001042393
RP11-33N1	0.214997604	7.28E-05	0.001055279
CTD-2116N	0.214916946	7.33E-05	0.00106117
NACA	-0.214794076	7.40E-05	0.001070665
VN1R108P	-0.214746044	7.43E-05	0.00107386
LAMP3	0.214721346	7.44E-05	0.001075078
GOLPH3L	-0.214636292	7.49E-05	0.00108145

ATP6V0A2	0.214433901	7.61E-05	0.001097996
U4.1	0.214344084	7.67E-05	0.001104911
AC142472.	-0.214252142	7.72E-05	0.001112054
MAST2	0.214218764	7.74E-05	0.001114077
C11orf95	-0.214131035	7.80E-05	0.001120903
LINC00337	0.21405691	7.84E-05	0.001126421
FAM180A	-0.214048106	7.85E-05	0.001126421
RP11-1398	-0.213871142	7.96E-05	0.001141321
HAGLROS	0.213708784	8.06E-05	0.001155076
TNNT2	-0.213661984	8.09E-05	0.001157536
ZFAS1	-0.21366111	8.09E-05	0.001157536
ABO	-0.213611369	8.12E-05	0.001161135
FSCN2	-0.213560021	8.16E-05	0.001164892
ZDHHC21	0.213549127	8.16E-05	0.001164945
WDR60	0.21353824	8.17E-05	0.001164999
HAUS2	0.21344289	8.23E-05	0.001172823
CH507-154	0.213406707	8.26E-05	0.001175214
PLSCR4	-0.213393265	8.27E-05	0.001175507
AC073283.	-0.213270759	8.35E-05	0.001185927
LINC00398	-0.213184708	8.40E-05	0.001193011
TUBB	0.213170113	8.41E-05	0.001193135
RP11-690D	-0.21316286	8.42E-05	0.001193135
UTP11	0.213093322	8.46E-05	0.001198705
MIR4261	0.213047658	8.49E-05	0.001202041
ARHGAP23	-0.21302777	8.51E-05	0.001202041
PARL	0.213027209	8.51E-05	0.001202041
PKMP3	-0.21299479	8.53E-05	0.001204085
RPS2	-0.21298371	8.54E-05	0.001204085
R3HDM1	0.212974921	8.54E-05	0.001204085
WISP2	-0.212906601	8.59E-05	0.001209126
DCLRE1B	0.212901283	8.59E-05	0.001209126
TSPYL5	-0.212890814	8.60E-05	0.001209153
RACK1	-0.21284675	8.63E-05	0.001212372
GMEB1	0.212812587	8.65E-05	0.001213758
RP4-568C1	-0.212811833	8.65E-05	0.001213758
FAM134A	-0.212703047	8.73E-05	0.001223182
ZNF165	0.2126591	8.76E-05	0.001226426
DKK3	-0.212635463	8.77E-05	0.001227666
AP001505.	0.212625912	8.78E-05	0.001227666
COG7	-0.212547217	8.83E-05	0.001234279
HIST1H2BH	0.212501719	8.86E-05	0.001235229
PJA2	-0.21249951	8.87E-05	0.001235229
JKAMP	-0.212491855	8.87E-05	0.001235229
PRPF38A	0.212488688	8.87E-05	0.001235229
RP4-613B2	-0.212486792	8.87E-05	0.001235229
AC006273.	-0.212472544	8.88E-05	0.001235631
RP11-322D	0.212446473	8.90E-05	0.001237178

PGAP2	-0.212386308	8.94E-05	0.001240558
NOB1	-0.21238247	8.95E-05	0.001240558
TOM1L2	-0.212381402	8.95E-05	0.001240558
CREBL2	-0.212300772	9.00E-05	0.001247429
AP2A2	-0.212268428	9.03E-05	0.00124874
SMC1B	0.212264445	9.03E-05	0.00124874
RP1-63M2.	0.212257199	9.04E-05	0.00124874
A2M-AS1	-0.212163775	9.10E-05	0.00125632
RP11-499E	-0.212159763	9.10E-05	0.00125632
AC068831.	0.212093177	9.15E-05	0.001261888
CTD-3035D	0.21204921	9.18E-05	0.001265241
SHC2	-0.21196596	9.24E-05	0.001271685
AC083843.	0.211964187	9.25E-05	0.001271685
ZBTB2	0.211923074	9.27E-05	0.001274779
CTD-2256P	0.211886151	9.30E-05	0.001277463
LIN37	0.211857764	9.32E-05	0.001278729
RIN1	-0.211853493	9.33E-05	0.001278729
BDH2	-0.211755431	9.40E-05	0.001287553
TRIM13	-0.211708567	9.43E-05	0.001291266
GAS5	-0.21164664	9.48E-05	0.001296511
SNORD119	0.211625898	9.49E-05	0.001297602
NDUFB4P8	0.211597348	9.51E-05	0.001299485
EPT1	0.211576689	9.53E-05	0.001300571
TOE1	0.211406058	9.66E-05	0.001316966
SNRPG	0.211347813	9.70E-05	0.001321932
ZNF93	0.211322389	9.72E-05	0.001323528
BAZ1A	0.211264801	9.76E-05	0.001328449
SRSF12	0.211248713	9.78E-05	0.001329087
THNSL1	-0.21120478	9.81E-05	0.001332611
RP11-108M	0.211134046	9.86E-05	0.001338935
ZDHHC24	-0.211100147	9.89E-05	0.001341437
ATAD3B	0.210723714	0.00010183	0.001380214
CCDC102A	-0.21058347	0.00010295	0.001393371
GPC2	0.210581716	0.00010296	0.001393371
RP11-231P	0.210539743	0.0001033	0.001396843
NOP56	0.210498558	0.00010363	0.001400239
NUP62CL	0.210410777	0.00010434	0.001408547
CASP3	0.210402518	0.0001044	0.001408547
CLEC9A	-0.210389838	0.00010451	0.001408853
ZNF300	0.210266048	0.00010551	0.001421352
FYCO1	-0.210248644	0.00010566	0.001422181
CPSF4	0.210224648	0.00010585	0.001423739
MOB2	-0.210132777	0.00010661	0.001432815
SPERT	-0.21011255	0.00010678	0.001433966
FABP5P3	0.210020306	0.00010754	0.001443145
RP1-152L7.	0.209975669	0.00010791	0.001447037
MBLAC2	-0.209951889	0.00010811	0.001448599

RP11-44K6	0.209940743	0.00010821	0.001448746
KLHL4	-0.209922392	0.00010836	0.001449701
CCNI	-0.209853719	0.00010894	0.001456318
NCAPH2	0.209778039	0.00010958	0.001463482
CD81-AS1	-0.209770639	0.00010964	0.001463482
EFCC1	-0.209754277	0.00010978	0.001464224
CDS1	-0.209707972	0.00011017	0.001468363
RP4-621N1	0.209674011	0.00011046	0.001471108
ZNF205	-0.209611699	0.00011099	0.001476355
TRIQK	-0.209606483	0.00011104	0.001476355
KIFAP3	-0.209598591	0.00011111	0.001476355
LINC00607	-0.209575599	0.0001113	0.001477864
USP13	0.20955699	0.00011146	0.001478865
RP5-1142J1	0.209547312	0.00011155	0.001478865
SNX6P1	0.209521974	0.00011177	0.001480647
FMR1-IT1	0.20926904	0.00011397	0.001508334
CES2	-0.209262492	0.00011403	0.001508334
CTD-2366F	-0.209130491	0.00011519	0.001522623
RPL10A	-0.209100583	0.00011546	0.001524992
MZT1	0.209060679	0.00011582	0.001528543
TIGD3	0.209027171	0.00011612	0.001531346
TTLL1	-0.208971944	0.00011661	0.001536725
TCTN3	-0.208962044	0.0001167	0.001536746
LINC00920	-0.208937706	0.00011692	0.001538479
BDNF-AS	-0.208893216	0.00011732	0.001542606
MUM1L1	-0.208861476	0.00011761	0.001545078
RP6-206I17	-0.208853028	0.00011768	0.001545078
JMJD8	-0.208757899	0.00011855	0.001555274
PGAP3	-0.208741244	0.0001187	0.001555302
PRTFDC1	0.208738298	0.00011873	0.001555302
LINC01572	0.208712896	0.00011896	0.001557186
AMIGO1	-0.208685154	0.00011921	0.001559353
BEND3	0.208640965	0.00011962	0.001563501
TMEM87A	-0.208613969	0.00011987	0.001565588
ENG	-0.208574219	0.00012024	0.001569216
C1QTNF1	-0.208431095	0.00012157	0.00158524
PHYHIP	-0.208422773	0.00012164	0.00158524
CSH2	0.208169629	0.00012403	0.001615164
MUM1	-0.208107634	0.00012462	0.001621666
RP11-641D	0.208094651	0.00012475	0.001622083
NLRX1	-0.208074986	0.00012494	0.001623332
DNAJA1	0.208061871	0.00012506	0.001623767
FZD6	-0.208030616	0.00012536	0.001626464
VN1R20P	-0.207989117	0.00012576	0.001630446
AC007919.	0.207892513	0.0001267	0.001641358
RNF225	-0.207846613	0.00012714	0.00164593
COX15	-0.207757017	0.00012802	0.001654682

RPS12P31	0.207737639	0.00012821	0.001654682
UBA2	0.2077317	0.00012827	0.001654682
TBCK	-0.207730364	0.00012828	0.001654682
PDLIM2	-0.207729462	0.00012829	0.001654682
SCRN2	-0.207685244	0.00012872	0.001658305
QRICH2	0.207681768	0.00012876	0.001658305
PHF5A	0.207648865	0.00012908	0.001661272
IFI44	0.207635522	0.00012922	0.001661679
UBXN1	-0.207626571	0.0001293	0.001661679
RP4-781B1	-0.207461629	0.00013095	0.001681558
LA16c-313I	-0.207429754	0.00013127	0.001684433
CDNF	-0.207414624	0.00013142	0.001685154
KCND3	-0.20736838	0.00013188	0.001689892
ALDH1L2	0.207251819	0.00013306	0.001702841
RP11-476B	0.207249479	0.00013309	0.001702841
HPRT1	0.207238415	0.0001332	0.001703043
RP11-54O7	0.207228618	0.0001333	0.001703081
RP11-381E	0.207194188	0.00013365	0.001706328
RAB4A	-0.207166795	0.00013393	0.001708662
AC003006.	0.207023774	0.0001354	0.001726174
MFSD6L	-0.206831525	0.0001374	0.001750414
LEPROT	-0.206820336	0.00013752	0.00175064
PAPD7	0.20679139	0.00013782	0.00175324
DHRS7B	-0.206674281	0.00013906	0.001767684
FAM220A	-0.206605743	0.00013979	0.001775232
MGME1	0.206599412	0.00013986	0.001775232
ATRIP.1	0.206558298	0.00014029	0.001778529
XPO1	0.206556126	0.00014032	0.001778529
MR1	-0.206338839	0.00014266	0.001806892
RP11-131L	0.20625886	0.00014353	0.001816611
ZBTB22	-0.206246274	0.00014367	0.001817044
OBFC1	-0.206209217	0.00014407	0.001820863
EPHX2	-0.206181544	0.00014438	0.001823388
RNU6-387P	0.206131442	0.00014493	0.001829034
ZNF788	0.206118096	0.00014507	0.001829341
CCDC34	0.206110371	0.00014516	0.001829341
AC127904.	0.206056471	0.00014575	0.001835535
TMSB15B	0.206016398	0.0001462	0.001839815
TRBV10-1	0.205963771	0.00014678	0.001845864
MIR10B	0.205914457	0.00014733	0.001851467
PLXDC2	-0.20586104	0.00014793	0.001857664
ANXA2P2	-0.205720811	0.00014952	0.0018762
BBS2	-0.205609029	0.00015079	0.001890826
AC006273.	-0.205394847	0.00015326	0.0019204
KLHL36	-0.20537641	0.00015347	0.001921715
C7orf49	0.205348246	0.0001538	0.001924245
TIA1	0.205340254	0.00015389	0.001924245

GINS4	0.205320431	0.00015412	0.001925767
LDB1	-0.205271381	0.0001547	0.00193156
MKRN1	0.205159939	0.00015601	0.001946539
RPL23	-0.205148818	0.00015614	0.001946797
RASSF5	-0.205052018	0.00015729	0.001959715
SLC46A1	-0.205006524	0.00015783	0.001963971
SNRPD1	0.205004613	0.00015785	0.001963971
MINK1	-0.20492394	0.00015882	0.001974588
ARAP1	-0.204904367	0.00015905	0.001976114
ZER1	-0.204804117	0.00016026	0.001989733
CEP128	0.20477605	0.0001606	0.00199255
KCNMB3	0.204742409	0.00016101	0.001996211
RP11-258F	-0.204632676	0.00016235	0.002011399
TSPAN4	-0.204566414	0.00016316	0.002020058
SLC25A42	-0.204524529	0.00016368	0.002025027
RP11-164P	0.204468544	0.00016437	0.002032166
RP11-98F1	0.20442288	0.00016494	0.002037191
NHLRC4	-0.20441717	0.00016501	0.002037191
USP37	0.204396284	0.00016527	0.00203897
ZSCAN12P1	0.204357493	0.00016575	0.002043169
RP4-751H1	0.204346989	0.00016588	0.002043169
PRC1-AS1	0.204341115	0.00016596	0.002043169
GPR3	0.204306614	0.00016639	0.002047055
YARS	0.204263581	0.00016693	0.002052269
RP11-109G	0.20414437	0.00016844	0.002069332
ZHX3	-0.204115306	0.00016881	0.002072418
MMS19	-0.204066905	0.00016942	0.002078533
PATL1	0.20390069	0.00017155	0.002103223
STIM1	-0.203852968	0.00017217	0.002109315
TONSL	0.203834174	0.00017241	0.002110827
ALDH3A2	-0.203819111	0.00017261	0.00211175
PRPF40B	-0.203660801	0.00017467	0.002135539
AC002310.	0.203584488	0.00017568	0.002146323
XKR4	-0.203506342	0.00017671	0.002157455
CLCN2	0.20346099	0.00017732	0.00216331
PICALM	-0.203390574	0.00017825	0.002173264
KDM4A-AS	0.203351405	0.00017878	0.00217815
RP13-16H1	0.203327825	0.0001791	0.002180495
RP11-274B	0.203309009	0.00017935	0.002181873
OAS3	0.203300964	0.00017946	0.002181873
NAA15	0.203252287	0.00018011	0.002188339
EMC9	0.203118794	0.00018192	0.002208819
CLGN	0.203033611	0.00018309	0.002221426
CHMP3	-0.202941608	0.00018435	0.002235241
SLC30A1	-0.202880849	0.00018519	0.002243884
SNORA11.1	0.202825739	0.00018596	0.002251606
ZNF777	0.202799082	0.00018633	0.002254549

PPP3CA	-0.202703378	0.00018767	0.002267172
NIPAL3	-0.202699492	0.00018772	0.002267172
SESN1	-0.202696878	0.00018776	0.002267172
LRRC29	-0.202684533	0.00018793	0.002267709
FBXO36	-0.202675079	0.00018807	0.002267757
FTH1P3	0.202663462	0.00018823	0.002268132
DAZAP1	0.202654546	0.00018836	0.002268132
LINC01337	0.202580036	0.00018941	0.00227924
PCAT6	-0.202560958	0.00018968	0.002280099
CKS1B	0.202543017	0.00018993	0.002280099
RNU6ATAC	0.202539886	0.00018998	0.002280099
FUCA1	-0.202538425	0.00019	0.002280099
PAK3	-0.202522804	0.00019022	0.002281206
FUK	-0.202463184	0.00019107	0.002289828
RN7SL179P	0.202426684	0.00019159	0.002294515
CNIH3	-0.20233961	0.00019284	0.00230791
PNN	0.2022931	0.00019351	0.002314362
RP11-147L	0.202259655	0.00019399	0.002318569
TOMM7	-0.202165006	0.00019537	0.002333413
TRIM23	-0.202146343	0.00019564	0.002335078
SPAG7	-0.202127299	0.00019592	0.002336811
STAT5B	-0.202106596	0.00019622	0.002338836
RPSAP13	0.202054019	0.00019699	0.002346432
TCOF1	0.202007392	0.00019768	0.002353008
EDRF1	0.201986365	0.00019799	0.002355105
PRSS3P2	0.201964572	0.00019831	0.002357338
TNFRSF9	0.201878597	0.00019958	0.002370883
KDELC2	0.201858891	0.00019988	0.002372764
FOXO3	-0.201799177	0.00020077	0.00238173
PGPEP1	-0.201686119	0.00020247	0.002400239
CYB5R1	-0.201661954	0.00020283	0.00240294
DDX55	0.201576984	0.00020412	0.002415124
PSMB2	0.201568615	0.00020424	0.002415124
CACNA1H	-0.201566831	0.00020427	0.002415124
NEMP2	0.201505657	0.0002052	0.002424506
KIF1C	-0.201427316	0.0002064	0.002437029
CST3	-0.201369655	0.00020729	0.002445429
NRAS	0.20136296	0.00020739	0.002445429
C17orf53	0.201345948	0.00020765	0.002446882
DLG4	-0.201323622	0.000208	0.002449303
SPATA2L	-0.201275977	0.00020873	0.002456341
LSM8	0.201253221	0.00020909	0.002458851
H2AFZ	0.201232512	0.00020941	0.00246099
G2E3	0.201218729	0.00020962	0.002461865
RP5-1061H	-0.201202663	0.00020987	0.002463159
MPG	-0.201159551	0.00021055	0.002469407
ERLIN2	-0.201112078	0.00021129	0.002473751

RBMS2	-0.201109129	0.00021133	0.002473751
P2RY12	-0.201108951	0.00021134	0.002473751
DYNC1LI1	0.201078025	0.00021182	0.002477785
SPEG	-0.201057024	0.00021215	0.00248
CLCN7	-0.201021983	0.00021271	0.002484803
CTC-351M1	-0.20100486	0.00021298	0.00248631
NRIR	0.200867628	0.00021515	0.002510072
EIF2D	-0.200829161	0.00021577	0.00251557
IMPDH2	-0.200805793	0.00021614	0.002518261
C1S	-0.200784961	0.00021648	0.002520482
HNRNPA1P	0.200694531	0.00021793	0.002535743
SFXN1	0.200602235	0.00021943	0.002550269
WASF3	-0.20059953	0.00021947	0.002550269
SLC16A1	0.200467491	0.00022162	0.002573609
SNRPFP4	0.20044929	0.00022192	0.002575377
ZNF887P	0.200430431	0.00022223	0.002577272
DHRS7	-0.200317039	0.0002241	0.002596629
MAPRE3	-0.200303942	0.00022432	0.002596629
APOLD1	0.200302512	0.00022434	0.002596629
SMIM14	-0.200274674	0.00022481	0.002600267
PRDX3P2	0.200215411	0.00022579	0.002608434
AR	-0.200214452	0.00022581	0.002608434
TEAD2	0.200202935	0.000226	0.002608942
RP3-370M2	0.200160203	0.00022672	0.002613327
PXN	-0.20015446	0.00022681	0.002613327
Y_RNA.206	0.20015358	0.00022683	0.002613327
SLC37A3	0.200073893	0.00022817	0.00262703
CCT7P1	0.200052964	0.00022852	0.002629372
TERF2IP	-0.199983731	0.00022969	0.002641116
DALRD3	-0.199963961	0.00023002	0.002643245
CTD-2210P	0.199889586	0.00023129	0.002656055
RP11-490M	-0.199872873	0.00023158	0.002657165
PLA2G16	-0.199866235	0.00023169	0.002657165
NME7	-0.199818465	0.00023251	0.002664806
NUP188	0.199765464	0.00023342	0.002673499
DPAGT1	-0.19960123	0.00023626	0.002704293
ITIH5	-0.199588741	0.00023648	0.002705022
CEP131	0.199562898	0.00023693	0.002706805
ACTR3B	0.199554411	0.00023708	0.002706805
WDR5	0.199553338	0.00023709	0.002706805
GLUD1P2	-0.199531772	0.00023747	0.002709349
SECISBP2L	-0.199517173	0.00023773	0.002710506
PRIM2	0.199458896	0.00023875	0.002720397
RP11-146F	0.199436388	0.00023914	0.002723144
AIM2	0.199418262	0.00023946	0.002725016
EBPL	-0.199398104	0.00023982	0.002725462
YWHAG	0.199395623	0.00023986	0.002725462

AC020550.	0.199383545	0.00024008	0.002725462
EARS2	-0.199377725	0.00024018	0.002725462
SEC61A2	0.19937129	0.00024029	0.002725462
TNK2-AS1	0.199363406	0.00024043	0.002725462
AK1	-0.199317742	0.00024124	0.002732873
SFTPD	-0.199292501	0.00024169	0.002735647
GLG1	-0.199286454	0.0002418	0.002735647
ZNF624	-0.199270815	0.00024208	0.002737035
ALDOC	-0.19923211	0.00024277	0.0027426
EIF3E	-0.199225729	0.00024288	0.0027426
GPIHBP1	-0.199172284	0.00024384	0.00275028
RP11-392A	0.199170245	0.00024387	0.00275028
MRM1	-0.199140602	0.0002444	0.002754513
SPNS3	-0.199125781	0.00024467	0.00275575
RIN3	-0.199060829	0.00024584	0.002767163
TMEM130	-0.199050414	0.00024603	0.002767511
RP11-561N	0.198982702	0.00024726	0.002779533
SNRPF	0.198956809	0.00024773	0.002783045
DHDH	0.198946824	0.00024791	0.002783309
NKD1	-0.198897631	0.00024881	0.002791584
PSME4	0.198889017	0.00024896	0.002791584
RP6-74O6.(	-0.198809201	0.00025043	0.002806198
BCAR1	-0.198770338	0.00025114	0.00281242
EIF2S1	0.198758798	0.00025135	0.002813012
RP1-172H2	-0.198725031	0.00025198	0.002818196
MBP	-0.198712325	0.00025221	0.002819031
TARDBP	0.198676714	0.00025287	0.002824609
MARVELD1	-0.198641049	0.00025353	0.002830209
DCAF12	0.198611975	0.00025408	0.002834449
CCDC89	-0.198554831	0.00025514	0.002842975
SNORA42	0.198553712	0.00025516	0.002842975
LPCAT1	0.198511661	0.00025595	0.002849945
ELMO3	-0.198436645	0.00025736	0.002863838
MAPKAPK5	0.198351576	0.00025897	0.002879914
GTF2E1	0.198334154	0.0002593	0.002881768
SNHG19	-0.198266611	0.00026058	0.002894223
ARL8A	-0.19822821	0.00026132	0.002900537
ARNTL2-AS	0.19814489	0.00026292	0.002916432
CTB-50L17.	0.198098924	0.0002638	0.002924209
RP11-406D	0.198082821	0.00026411	0.002924209
BCL2L1	-0.198082637	0.00026412	0.002924209
RP11-156P	-0.198039529	0.00026495	0.002931595
SRSF8	-0.198015026	0.00026542	0.002935005
DERL3	0.197949752	0.00026669	0.002947188
CNOT10	0.197912295	0.00026742	0.002953411
PATE4	0.197855911	0.00026853	0.00296374
RANBP9	0.197823219	0.00026917	0.002968963

PHKB	-0.197801626	0.0002696	0.002971785
GART	0.197744335	0.00027073	0.002982373
ESD	-0.197662089	0.00027236	0.002998452
LTA4H	-0.197643866	0.00027272	0.003000564
DNAJB5	0.197618728	0.00027322	0.003001777
DNAJC2	0.197616019	0.00027327	0.003001777
EHD2	-0.197612637	0.00027334	0.003001777
WEE1	0.197516424	0.00027526	0.003021031
FUT3	-0.19750012	0.00027559	0.003022739
CLIP4	-0.197434393	0.00027692	0.003035365
LINC01135	-0.19742512	0.0002771	0.003035527
DNAJB11	0.19736277	0.00027837	0.003047453
VAMP2	-0.197265408	0.00028035	0.003067236
RNU1-103F	0.197224522	0.00028118	0.003074325
FAHD1	-0.197211845	0.00028144	0.003074325
KIAA1217	-0.197208114	0.00028152	0.003074325
LIN52	0.197190442	0.00028188	0.003076373
AC010525.	0.197154213	0.00028263	0.003082585
UPK3B	-0.197125614	0.00028321	0.003087095
UQCRC2	-0.197103779	0.00028366	0.00309009
H2AFZP6	0.197069049	0.00028438	0.003095992
AP000240.	0.197040952	0.00028496	0.003100409
PLCXD1	0.197001824	0.00028578	0.003107325
DPH7	0.196987469	0.00028608	0.00310865
FBXO32	-0.196930113	0.00028727	0.003119716
ZDHHC7	-0.196890639	0.0002881	0.003126752
TIMM23	0.196800284	0.00028999	0.003145414
EIF4EBP1	0.196715671	0.00029178	0.003162859
RP11-69M1	-0.196650923	0.00029316	0.003174163
MCUR1	0.196649588	0.00029319	0.003174163
MRPL53	0.196628294	0.00029364	0.003177119
RP11-567M	-0.196615106	0.00029392	0.003178208
LILRA2	-0.196470091	0.00029703	0.003209861
RP11-799D	-0.19645312	0.0002974	0.00321123
GK	0.196447288	0.00029752	0.00321123
C5orf60	0.196433603	0.00029782	0.003212448
KAZN	-0.196380018	0.00029898	0.003222827
TMEM106E	-0.196372228	0.00029915	0.003222827
EDA2R	-0.196346043	0.00029972	0.003226976
PIGV	-0.196330966	0.00030004	0.00322853
IL36G	0.196306892	0.00030057	0.003230405
CD274	0.196306094	0.00030059	0.003230405
EIF2B5	0.196160802	0.00030377	0.003262602
SH3BP4	-0.19607371	0.00030569	0.003281243
LAMA2	-0.196054946	0.0003061	0.003282348
RP11-545I5	-0.196052215	0.00030616	0.003282348
RP11-552D	-0.196038453	0.00030647	0.003282698

XYLT2	-0.196033921	0.00030657	0.003282698
GSTM5	-0.195991967	0.0003075	0.003290679
AC093627.	-0.195744738	0.00031305	0.00334802
RP11-547D	0.195728269	0.00031342	0.003349087
CYP2T1P	-0.195723521	0.00031353	0.003349087
POLR2H	0.195683406	0.00031444	0.003355497
ABLIM1	-0.195680273	0.00031451	0.003355497
SUOX	-0.195629235	0.00031568	0.003364019
CDH22	-0.195628392	0.00031569	0.003364019
KLHDC8A	-0.195612389	0.00031606	0.003365872
Y_RNA.801	0.195596984	0.00031641	0.003367582
PAFAH1B3	0.195560571	0.00031725	0.003374409
DDIT4L	-0.195525267	0.00031806	0.003379075
MRPL37	0.195524706	0.00031807	0.003379075
WFDC5	-0.195444533	0.00031991	0.00339664
TSC22D1	-0.195381572	0.00032137	0.00340933
TGM1	-0.195376146	0.0003215	0.00340933
SH3D19	-0.195306668	0.00032311	0.003424399
RAB3A	-0.195194932	0.00032573	0.003450025
PDE4A	-0.195169393	0.00032633	0.00345389
EEF1A1P19	0.195162711	0.00032648	0.00345389
HSPA13	0.195107044	0.0003278	0.003463882
ZNF670-ZN	0.195105952	0.00032782	0.003463882
UBE2R2-AS	0.195025811	0.00032972	0.003480493
PRELP	-0.195022888	0.00032979	0.003480493
GAB2	-0.194997192	0.0003304	0.003484851
IKZF3	0.194971344	0.00033102	0.003489252
PARD3B	-0.194948186	0.00033157	0.00349116
MOB3A	-0.194947121	0.0003316	0.00349116
ING3	0.194921097	0.00033222	0.003495616
GABARAPL	-0.194910313	0.00033248	0.00349624
F8	-0.194894225	0.00033286	0.003498201
CPEB2	-0.194861913	0.00033364	0.003504254
MAP6	-0.194783108	0.00033553	0.00352208
LINC00910	-0.194762924	0.00033602	0.003525095
PDK1	0.19475104	0.00033631	0.003526008
DAZAP2	-0.194735737	0.00033668	0.003527789
STYK1	0.194708514	0.00033734	0.003532599
PTMAP1	0.194677278	0.0003381	0.003537897
SYK	0.194671134	0.00033825	0.003537897
TTC3	-0.194594876	0.00034011	0.003555233
CRYAB	-0.194585972	0.00034032	0.003555397
IARS	0.194573657	0.00034062	0.003556433
APBA1	-0.194552575	0.00034114	0.003557942
HIST1H3F	0.194551253	0.00034117	0.003557942
TRBV6-9	0.194533536	0.00034161	0.003560364
AHNAK	-0.194494345	0.00034257	0.003566817

LYPD5	0.194491855	0.00034263	0.003566817
ODF2-AS1	0.194460377	0.00034341	0.003572778
C9orf38	0.194420653	0.00034439	0.003580869
FGF21	0.194407751	0.00034471	0.003582071
CTD-2196E	0.194398083	0.00034495	0.003582443
SHISA4	-0.194370694	0.00034563	0.003587378
CCT6A	0.194227969	0.00034919	0.003620938
SNHG7	-0.194224534	0.00034927	0.003620938
NUP62	0.194119429	0.00035192	0.003646193
CHRNA6	-0.194097807	0.00035246	0.00364903
TTL	0.194092165	0.0003526	0.00364903
RP11-120D	0.194066268	0.00035326	0.003653663
ZSWIM1	-0.194057711	0.00035348	0.003653757
AKR7L	0.194044992	0.0003538	0.003654726
PSKH1	-0.19403763	0.00035399	0.003654726
RP1-152L7.	0.194021577	0.00035439	0.003656788
CDH12	0.193998868	0.00035497	0.003659212
RP4-784A1	0.193995974	0.00035504	0.003659212
RHEBL1	0.193978856	0.00035548	0.003659342
RP1-313L4.	-0.193974371	0.00035559	0.003659342
RP11-426L	0.193970978	0.00035568	0.003659342
RPS6KA2	-0.193888956	0.00035778	0.003678758
RAET1K	0.193842991	0.00035896	0.003688733
AP000280.	-0.193796564	0.00036015	0.003698856
UTP14C	-0.193783097	0.0003605	0.003700264
RNASET2	-0.193573976	0.00036594	0.003753862
RAB3B	0.193547716	0.00036662	0.003758302
RP11-277L	-0.193541148	0.0003668	0.003758302
CTD-2007L	0.193519497	0.00036736	0.003759815
RP11-806L	0.193519239	0.00036737	0.003759815
AC007246.	-0.193489309	0.00036816	0.003765678
ARL6IP6	0.193317375	0.00037271	0.003810015
FXYD5	-0.193293564	0.00037334	0.003814284
RP4-802A1	0.193249741	0.00037451	0.003823415
RARA-AS1	-0.193243826	0.00037467	0.003823415
ZNF566	0.193229012	0.00037507	0.003825243
IGF2BP3	0.19319948	0.00037586	0.003831095
KDM6B	-0.193154145	0.00037708	0.003841287
ABCA8	-0.193137516	0.00037753	0.003841991
PPP2R3B	0.19313536	0.00037758	0.003841991
LMO4	0.193119078	0.00037802	0.003842327
FOXD2-AS1	0.193117935	0.00037805	0.003842327
BEND3P2	0.193085978	0.00037892	0.003848431
LRP2	0.193079505	0.00037909	0.003848431
RP11-634H	0.193042993	0.00038008	0.003852294
CH507-254	0.19304294	0.00038008	0.003852294
KLHL12	-0.193041194	0.00038013	0.003852294

CTD-2024I7	0.193018315	0.00038075	0.003855627
TBC1D5	-0.193012926	0.0003809	0.003855627
FBRS	-0.19299752	0.00038131	0.003857647
IFI30	0.19294577	0.00038272	0.003869683
TMEM99	-0.192928865	0.00038319	0.003872124
TMEM115	-0.19289435	0.00038413	0.003879435
ATG9B	0.192875368	0.00038465	0.003882458
AK8	-0.192836104	0.00038573	0.003891105
CD19	0.192776944	0.00038736	0.003905301
VSIG10	0.192764785	0.00038769	0.003906447
KIAA1551	-0.192735725	0.0003885	0.0039123
UFD1L	0.192717241	0.00038901	0.003915213
FBXO8	-0.192695959	0.0003896	0.00391769
RNU6-8	0.192692301	0.0003897	0.00391769
SFN	0.19265084	0.00039085	0.003927029
PRKAR1A	-0.192641156	0.00039112	0.003927494
RPSAP69	0.192514614	0.00039466	0.003960763
NMNAT3	-0.192486964	0.00039544	0.003966299
ABCG2	-0.192471045	0.00039588	0.00396853
SLC43A3	0.192448333	0.00039652	0.003972682
CXCL10	0.192413262	0.00039751	0.003980336
POP1	0.192322488	0.00040009	0.004002399
TTC39B	0.192313628	0.00040034	0.004002399
RP11-354K	-0.192304736	0.00040059	0.004002399
UQCRHL	0.192303518	0.00040063	0.004002399
UBE2N	0.192273094	0.0004015	0.004008786
RHOF	-0.19225738	0.00040194	0.004010988
ZNF257	0.192223632	0.00040291	0.004018338
DLGAP3	0.192211716	0.00040325	0.004019462
CDC5L	0.192073537	0.00040723	0.004056477
KRT6B	-0.192066679	0.00040742	0.004056477
HEIH	-0.192027393	0.00040856	0.004065504
CPSF6	0.191971625	0.00041018	0.004079318
CACNG4	-0.191945189	0.00041095	0.004084667
C12orf66	-0.191914353	0.00041185	0.0040913
SNORA57.2	0.191858666	0.00041348	0.004105173
UNC5B	-0.191742398	0.0004169	0.004136815
MAPKBP1	-0.191689078	0.00041848	0.004150135
PRSS3	0.191623505	0.00042043	0.004167109
RP1-101K1	0.191555809	0.00042245	0.00418362
SENP2	0.191551767	0.00042257	0.00418362
RP5-1158E	0.191541231	0.00042289	0.004184387
POLH	0.191509584	0.00042384	0.004188808
PHF21A	-0.191508501	0.00042387	0.004188808
RP11-70L8.	0.191498594	0.00042417	0.004188808
STARD4	0.191494571	0.00042429	0.004188808
RP11-498C	0.191397952	0.0004272	0.004215182

PDK2	-0.191337277	0.00042904	0.00423094
RPL21P110	0.191281335	0.00043074	0.004245332
NTS	0.191271766	0.00043103	0.004245826
MYOF	-0.19125952	0.0004314	0.004246487
RP11-122G	-0.191253737	0.00043158	0.004246487
ARHGAP44	-0.191243586	0.00043189	0.00424716
RP11-218M	-0.191225618	0.00043244	0.004247605
LMOD1	-0.191223561	0.0004325	0.004247605
AZIN2	-0.191218403	0.00043266	0.004247605
EGFL6	0.191210396	0.0004329	0.004247639
SSH3	-0.191185173	0.00043367	0.004252846
SNX22	0.191172595	0.00043406	0.004254257
TLDC1	-0.191164249	0.00043432	0.004254397
GAL3ST2	-0.191151849	0.0004347	0.004255757
RP11-762H	0.191082145	0.00043684	0.004274372
UPK3A	0.191074328	0.00043708	0.004274372
RP11-242C	0.190970359	0.00044031	0.004303478
YEATS2	0.190946618	0.00044104	0.004308304
REEP6	-0.190917487	0.00044195	0.004314776
NXT2	0.190906408	0.0004423	0.004315756
MED16	-0.190860902	0.00044372	0.004327237
TNFAIP8	0.190827733	0.00044476	0.004332192
AGBL3	0.190822628	0.00044492	0.004332192
TEPSIN	0.190821132	0.00044497	0.004332192
IL17RC	-0.190807888	0.00044538	0.004333843
ZMYND19	0.190791431	0.0004459	0.004336479
SNRNP40	0.190752281	0.00044713	0.004346067
EFCAB14-A	0.190630256	0.000451	0.004381197
RASGRP4	-0.190596211	0.00045208	0.004389297
SLC24A3	-0.190550092	0.00045355	0.004401155
LINC01436	-0.190514049	0.0004547	0.004407952
CAMTA2	-0.190512534	0.00045475	0.004407952
NFE2L1	-0.19050307	0.00045506	0.004408462
MRGPRF	-0.190491017	0.00045544	0.004409778
RNU6-574F	0.190412014	0.00045798	0.00443194
SNORD14E	0.190298985	0.00046164	0.004464882
PINK1	-0.190274253	0.00046245	0.004468891
MTA2	0.19027061	0.00046256	0.004468891
SLC8A1-AS1	0.190254025	0.0004631	0.004471652
NOCT	0.190225807	0.00046402	0.00447808
INHBE	0.190208589	0.00046459	0.004481048
PRICKLE1	-0.190198271	0.00046492	0.004481844
RNF32	0.190157544	0.00046626	0.004492236
PTGFR	-0.19012464	0.00046734	0.004500176
TRAF7	-0.190109198	0.00046784	0.004500438
NME3	-0.190108249	0.00046788	0.004500438
RP11-180M	0.190054924	0.00046963	0.004512089

MAD1L1	-0.190045419	0.00046995	0.004512089
RP11-575G	0.19004146	0.00047008	0.004512089
TOB2	-0.1900336	0.00047034	0.004512089
ATP1A1-AS	-0.190032617	0.00047037	0.004512089
SLC9B1	-0.18997942	0.00047213	0.004526424
SLC7A4	-0.189971957	0.00047238	0.004526424
HOXD3	0.189949094	0.00047314	0.004531231
NDUFV2	0.189928529	0.00047382	0.004535312
HEBP1	-0.189898164	0.00047483	0.004542523
LGR6	-0.189885194	0.00047527	0.004544191
CARD11	-0.189856142	0.00047624	0.004550027
LINC01215	0.189851438	0.00047639	0.004550027
NSMCE4A	0.189815757	0.00047759	0.004558964
TYMSOS	0.189701353	0.00048144	0.004591173
RP11-472N	0.189699973	0.00048148	0.004591173
EXD2	-0.18969016	0.00048182	0.004591846
RP11-867G	0.18966571	0.00048264	0.004595897
RP11-409K	0.189662151	0.00048276	0.004595897
SDAD1P1	-0.189635566	0.00048366	0.004601984
NOP58	0.189606388	0.00048465	0.004608917
RP11-299J3	-0.189581486	0.0004855	0.004614477
SEPHS1	0.189550963	0.00048654	0.004621865
MIR98	0.189507235	0.00048804	0.00463355
RP11-288H	0.189477493	0.00048905	0.004640714
RP11-644F	-0.189397471	0.0004918	0.004664288
RBM25	0.189375127	0.00049257	0.004666976
RP1-86C11	0.189373861	0.00049262	0.004666976
ATP9A	-0.189293888	0.00049538	0.004690658
RNASEH1	0.189282079	0.00049579	0.004692013
LY6G6C	-0.189227535	0.00049769	0.004707428
RAI1	-0.189184695	0.00049918	0.004718284
RP11-546J1	0.189164976	0.00049987	0.004718284
GIMAP5	0.189164068	0.0004999	0.004718284
RP11-387A	0.189163917	0.00049991	0.004718284
POLDIP3	-0.189149396	0.00050042	0.004720547
CUEDC1	-0.18913173	0.00050104	0.004723851
DTWD1	-0.189108351	0.00050186	0.004728506
C17orf100	-0.189102335	0.00050207	0.004728506
AC093838.	0.189002332	0.00050559	0.004758407
SNORD20	0.188990567	0.00050601	0.004758407
MURC	0.188989232	0.00050605	0.004758407
RINT1	0.18897812	0.00050645	0.004759561
SESN3	-0.188927354	0.00050825	0.004773928
SNORD46	0.18885174	0.00051094	0.004796653
NAA50	0.188838156	0.00051142	0.004798646
PORCN	-0.188798676	0.00051284	0.004806307
RP11-27K1	0.188793754	0.00051301	0.004806307

snoU13.274	0.188792427	0.00051306	0.004806307
ZNF652	-0.188735782	0.00051509	0.004822786
RP11-240G	0.188700679	0.00051636	0.004832047
SPATA7	-0.18862085	0.00051924	0.004856458
STRA13	0.188572163	0.00052101	0.004868322
ZNF280C	0.188570643	0.00052106	0.004868322
MICALL2	-0.188557658	0.00052154	0.004870147
LTBP4	-0.188540727	0.00052215	0.004873314
RP11-474O	-0.188454818	0.00052529	0.004899996
DYNC2LI1	-0.188388633	0.00052772	0.004918725
USP33	0.188380875	0.000528	0.004918725
CYP39A1	-0.18837728	0.00052814	0.004918725
PER1	-0.18831563	0.00053041	0.004937285
LA16c-380A	-0.188239986	0.00053321	0.004960743
AC005682.	-0.188228166	0.00053365	0.004962204
RP4-536B2	0.188191716	0.00053501	0.004972186
BCL2L12	0.188179653	0.00053546	0.0049736
CRYBB2P1	0.188172463	0.00053572	0.0049736
RP11-235D	-0.188144267	0.00053678	0.004978217
FAM134C	-0.188143979	0.00053679	0.004978217
STAG3L3	0.188134938	0.00053713	0.004978726
MCOLN3	0.18811394	0.00053791	0.004983382
SNRPB	0.188058179	0.00054	0.005000122
SLC22A18	-0.188026261	0.0005412	0.005008602
ZDHHC4	-0.187976123	0.00054309	0.005023456
RPS14P4	0.187963681	0.00054356	0.005025163
RRAGD	0.187868348	0.00054718	0.005054969
MECOM	0.187863511	0.00054736	0.005054969
IGSF23	0.187781418	0.00055049	0.005079889
QSER1	0.187777643	0.00055064	0.005079889
RP11-318C	0.187760071	0.00055131	0.005083429
ZNF326	0.18758961	0.00055788	0.005141264
PKP3	-0.187571459	0.00055858	0.005143274
SNORD3B-1	0.187563745	0.00055888	0.005143274
HPS6	-0.187554361	0.00055924	0.005143274
VTRNA1-3	0.18755382	0.00055926	0.005143274
MFAP3L	-0.187516496	0.00056071	0.005151936
RNA5SP245	0.187510397	0.00056095	0.005151936
RP11-554E	0.187506987	0.00056108	0.005151936
MISP	-0.187439222	0.00056373	0.005173511
RP1-267L14	0.187411905	0.0005648	0.005180619
FBXL2	-0.187380711	0.00056602	0.005189104
RP11-640M	-0.187373274	0.00056631	0.005189104
RIPK4	-0.187305298	0.00056899	0.005209351
TBCCD1	0.187298727	0.00056925	0.005209351
GOLGA2P8	0.187294575	0.00056941	0.005209351
RP11-715F	0.187282363	0.00056989	0.005211052

HEY1	0.187229067	0.000572	0.005227616
RP13-890H	-0.18722124	0.00057231	0.005227735
NRIP3	0.187210127	0.00057275	0.005229047
ACTG1P15	0.187202357	0.00057306	0.005229149
STAT1	0.187179613	0.00057396	0.005234679
AC114730.	-0.187138617	0.0005756	0.005246843
KCTD8	-0.18711709	0.00057645	0.005250662
RP11-478C	-0.187113154	0.00057661	0.005250662
HEXB	-0.187103029	0.00057702	0.005251626
CDKN2A-AS	0.187080798	0.0005779	0.005256996
WI2-80269	0.187051551	0.00057908	0.005264928
C15orf52	-0.187012021	0.00058066	0.005276626
CNOT4	0.186932269	0.00058388	0.005303086
RPL30	-0.186909524	0.00058479	0.005307298
GALNT10	-0.186905873	0.00058494	0.005307298
CTD-2527I2	0.186866033	0.00058656	0.00531814
SNHG1	0.18686146	0.00058674	0.00531814
UCP1	0.186853609	0.00058706	0.005318288
RNU5F-1	0.186822519	0.00058832	0.005326991
MIR149	-0.186784513	0.00058987	0.005338259
ANXA11	-0.18676379	0.00059072	0.005343162
PTMA	0.186706175	0.00059307	0.005361724
RBMS3	-0.18669565	0.00059351	0.005362869
RP11-121M	-0.186683246	0.00059401	0.005364712
SLC25A21-A	-0.186629636	0.00059622	0.005381854
EGLN1	-0.186616797	0.00059675	0.005383867
NFIC	-0.186560845	0.00059906	0.00540194
IRF9	0.186511765	0.00060109	0.005417499
TMEM120A	-0.186472244	0.00060273	0.005427523
MAPKAPK2	-0.186470148	0.00060282	0.005427523
CTD-2318C	0.186455751	0.00060342	0.00543014
PRPF39	0.186422248	0.00060482	0.005439926
OR6V1	0.186378871	0.00060663	0.005453139
RP1-92O14	0.186372271	0.00060691	0.005453139
CTAGE6	0.186346541	0.00060798	0.005460038
MRPS36P5	-0.186286102	0.00061052	0.005480046
WDR91	0.186165103	0.00061564	0.005523128
TM9SF3	-0.18613709	0.00061683	0.005529326
ST7	0.18613403	0.00061696	0.005529326
KCNQ1	-0.186120068	0.00061755	0.00553183
RP1-272L16	-0.186072226	0.00061959	0.005546599
ACP6	-0.186066588	0.00061983	0.005546599
TMEM159	-0.186010873	0.00062221	0.005564282
CTD-2152M	0.186005602	0.00062244	0.005564282
C16orf58	-0.185982934	0.00062341	0.005570143
AHNAK2	-0.185929794	0.00062569	0.005587719
RRAD	-0.185907106	0.00062667	0.005591392

RP1	-0.185903135	0.00062684	0.005591392
SMIM6	-0.185898135	0.00062706	0.005591392
FOXQ1	-0.185817945	0.00063053	0.005619479
TPPP3	-0.185689697	0.00063611	0.005665256
TMC3-AS1	0.185685239	0.00063631	0.005665256
SUGP2	0.185674122	0.0006368	0.005666719
SERINC1	-0.185650921	0.00063781	0.005670834
GALNT11	0.18564885	0.0006379	0.005670834
TRIM16	-0.185564766	0.0006416	0.005693726
CCSAP	0.185564401	0.00064162	0.005693726
VIT	-0.185563441	0.00064166	0.005693726
HMGN3-AS	-0.185556362	0.00064197	0.005693726
CBX3	0.185553501	0.0006421	0.005693726
AC006547.	0.185525729	0.00064332	0.005700591
RP11-494H	0.185521291	0.00064352	0.005700591
RP11-10J5.	0.185500029	0.00064446	0.005706048
RP11-802E	0.18546029	0.00064622	0.005718763
MAFK	-0.185443147	0.00064698	0.00572262
RBBP7	0.185401019	0.00064885	0.00573631
SUSD6	-0.185369741	0.00065025	0.005744691
RP11-351J2	-0.185365114	0.00065046	0.005744691
RAD21-AS1	-0.185339201	0.00065161	0.005752029
MRPS27	-0.185299563	0.00065339	0.005764802
MUTYH	0.18528635	0.00065398	0.005767138
AP000442.	0.185239018	0.00065611	0.00578048
C15orf39	-0.185238054	0.00065615	0.00578048
CCDC88A	0.185193944	0.00065814	0.005795091
SLC45A1	-0.185186347	0.00065848	0.00579521
RP11-121C	0.185133031	0.00066089	0.005813524
AC226118.	-0.185125298	0.00066124	0.0058137
SSBP3	0.185112937	0.0006618	0.005815722
VWA8-AS1	-0.18508654	0.000663	0.005823344
CTD-2530H	-0.18505821	0.00066429	0.005831749
FLJ37035	-0.185045304	0.00066488	0.005833998
C20orf85	-0.185025863	0.00066577	0.005838862
TTC7A	-0.185015386	0.00066624	0.005840143
CYP27A1	-0.184942468	0.00066958	0.005865932
LRRC23	-0.184930146	0.00067014	0.005865932
HSPB7	-0.184929251	0.00067019	0.005865932
AC016768.	-0.184917834	0.00067071	0.005865955
HES6	0.184911068	0.00067102	0.005865955
ATAD2B	0.184907413	0.00067119	0.005865955
RPL34	-0.184875345	0.00067266	0.00587593
RP11-80A1	-0.184839456	0.00067432	0.005884145
C16orf46	-0.184836984	0.00067443	0.005884145
VWA2	-0.184830562	0.00067473	0.005884145
ADAMDEC1	0.184825949	0.00067494	0.005884145

RP11-172H	0.184814466	0.00067547	0.005885854
NSUN5P1	0.184788046	0.00067669	0.005893587
CACNG1	-0.184721852	0.00067977	0.00591741
EIF2AK2	0.184667628	0.00068229	0.005933953
LPCAT2	-0.184666573	0.00068234	0.005933953
AP001055.	0.184636976	0.00068372	0.005943037
RP11-572C	0.184629596	0.00068407	0.0059431
GBP1P1	0.184589485	0.00068595	0.005956479
AC093590.	-0.184539032	0.00068832	0.005974107
RPL9	-0.18451892	0.00068926	0.005979374
GPR137C	0.184501161	0.0006901	0.005983684
RP11-150D	-0.184481771	0.00069102	0.005988665
BTBD9	-0.1844676	0.00069169	0.005991515
AC000068.	0.184444712	0.00069277	0.005997937
ENPP5	-0.184430198	0.00069345	0.006000933
GNPDA2	-0.184400336	0.00069487	0.006010229
RP11-750H	0.184326648	0.00069838	0.006037577
CEP76	0.184290969	0.00070008	0.006049328
BRIX1	0.18426734	0.00070121	0.006054383
RNU1-134F	0.184264345	0.00070135	0.006054383
STEAP1B	0.184221325	0.00070341	0.006069205
CHADL	-0.18421234	0.00070385	0.00606995
LRRC37A5F	-0.184201712	0.00070436	0.006071376
TRIM58	-0.184180233	0.00070539	0.006077302
NCLP1	0.184152901	0.0007067	0.006083363
RP11-420L!	-0.184150838	0.0007068	0.006083363
PNPLA2	-0.184144096	0.00070713	0.006083363
PRKCH	-0.184126678	0.00070797	0.006087618
BIN1	-0.184070757	0.00071067	0.00610789
BNIP3P34	0.183953803	0.00071636	0.006153768
UCHL1	0.183911991	0.00071841	0.006166766
BECN1	-0.183908507	0.00071858	0.006166766
RNF39	-0.183811396	0.00072335	0.006204661
SFXN3	-0.183792893	0.00072426	0.006209454
SPIN2A	0.183774402	0.00072517	0.006214248
GUSBP1	0.183694076	0.00072914	0.006245273
RNF130	-0.183631675	0.00073225	0.006268792
RAB25	-0.183615371	0.00073306	0.006272697
NOS1	-0.183600162	0.00073382	0.006276136
RPA4	0.183582437	0.0007347	0.006280654
PNRC1	-0.183566915	0.00073548	0.006284234
UFC1	-0.183536121	0.00073702	0.006290983
RP4-594I1C	-0.183532927	0.00073718	0.006290983
APOD	-0.183527321	0.00073746	0.006290983
NFKBIB	0.183522585	0.0007377	0.006290983
SLC9A8	-0.183507131	0.00073848	0.006294545
ANAPC1P1	0.183464246	0.00074063	0.00630805

LDHD	-0.183461354	0.00074078	0.00630805
RP11-10O1	0.18342651	0.00074253	0.006319946
ATP6V0E2	0.183409364	0.0007434	0.006324254
SCAMP1	-0.183385212	0.00074462	0.006331579
ADCY9	-0.183375619	0.0007451	0.006332647
TLX2	0.183319773	0.00074794	0.006353646
TRADD	-0.183298303	0.00074903	0.006357528
AC009336.	0.183294844	0.0007492	0.006357528
HSPA6	0.183289465	0.00074948	0.006357528
TMCC3	-0.183229288	0.00075255	0.00638048
BNC2	-0.183216674	0.00075319	0.00638287
TIMM10B	-0.183196646	0.00075422	0.006388476
PRSS35	-0.183140431	0.0007571	0.006409808
FAM160A2	-0.183132606	0.0007575	0.006410128
C2orf74	-0.183109097	0.00075871	0.006417274
FAM222B	-0.183077957	0.00076031	0.006425441
CTD-2547L	-0.183076182	0.00076041	0.006425441
VBP1	0.18306478	0.00076099	0.006427325
B3GALT2	-0.183015539	0.00076354	0.006445731
RPS13	-0.182903633	0.00076935	0.006491702
CCDC24	-0.182885901	0.00077028	0.006496392
Y_RNA.323	0.182847446	0.00077229	0.006508355
RP11-142E	0.182844606	0.00077244	0.006508355
CCM2L	0.182829696	0.00077322	0.006511814
RP11-252K	-0.18280969	0.00077427	0.006515998
AADAT	0.182801989	0.00077467	0.006515998
NAP1L2	-0.182799031	0.00077483	0.006515998
SERP2	-0.182766937	0.00077651	0.006527057
PTGDS	-0.182732373	0.00077833	0.006539229
RP11-161H	0.182689077	0.00078062	0.006555295
RP11-108L	0.182668396	0.00078171	0.006561347
RPL13A	-0.182646646	0.00078286	0.00656788
FAM114A1	-0.182628937	0.0007838	0.006572623
PFN4	0.182590154	0.00078586	0.006586756
CCT6P1	0.182577561	0.00078653	0.006589231
SHF	-0.182555765	0.00078769	0.00659403
PARP2	0.18255273	0.00078785	0.00659403
CRIP3	0.182505992	0.00079035	0.006610894
UTF1	-0.18250091	0.00079062	0.006610894
ENO3	0.182484267	0.00079151	0.006615195
RPEP4	0.182470365	0.00079225	0.006618273
PLEKHO1	0.182456876	0.00079298	0.006621168
OR13Z2P	-0.182425313	0.00079467	0.006632072
ARL15	-0.18241482	0.00079524	0.006632072
KBTBD3	-0.182411494	0.00079541	0.006632072
LIAS	-0.182366538	0.00079784	0.006646844
CTD-3032J	0.182364563	0.00079794	0.006646844

HUS1B	0.182319958	0.00080035	0.006663754
HHAT	-0.182262849	0.00080344	0.00668635
TPTE2P1	-0.182254088	0.00080392	0.006687145
PYGB	-0.18222244	0.00080564	0.006698287
PERP	-0.182144645	0.00080988	0.006730385
PP14571	-0.182126356	0.00081088	0.006735518
NR2F2-AS1	-0.182003252	0.00081765	0.006788499
IQCA1	-0.181971665	0.00081939	0.00679977
ANKRD36B	0.181958105	0.00082014	0.006802783
BAALC	-0.181932138	0.00082158	0.006811495
ECE2	0.18191008	0.0008228	0.006818421
PRELID3A	0.181870337	0.00082501	0.006832627
FOXL2	-0.181860364	0.00082557	0.006832627
LDHAL6B	0.181858263	0.00082568	0.006832627
TOR3A	0.181836649	0.00082689	0.006839371
TMEM52	0.18182695	0.00082743	0.006840628
AC073052.	0.181814436	0.00082812	0.006843184
TGOLN2	-0.181746091	0.00083195	0.006871543
ARID5B	-0.181725035	0.00083313	0.006878068
MMRN1	-0.181711666	0.00083388	0.006878216
FAM227B	-0.18171079	0.00083393	0.006878216
CYB5R3	-0.181649638	0.00083737	0.006903366
Y_RNA.790	0.181579267	0.00084135	0.0069329
RP11-84A1	0.181554334	0.00084276	0.006941294
RP11-156K	-0.181487301	0.00084657	0.006969412
FAM210B	-0.181454436	0.00084844	0.006981572
RP11-473M	-0.181390698	0.00085209	0.007008287
ZNF829	0.181383459	0.0008525	0.007008424
HIST1H3B	0.181360248	0.00085383	0.007016092
ETFBKMT	-0.181182398	0.0008641	0.00709714
IFIT1P1	0.181148267	0.00086608	0.007108403
MYNN	0.181144895	0.00086628	0.007108403
DESI2	0.18108787	0.0008696	0.007132349
CECR1	-0.181018641	0.00087366	0.007162234
PAK1IP1	0.181008955	0.00087422	0.007163553
RP11-28H5	0.18098052	0.00087589	0.007173894
LRSAM1	-0.180935699	0.00087853	0.007192153
AC092159.	0.180864507	0.00088274	0.007220195
RP11-567M	-0.180863817	0.00088278	0.007220195
HSPB8	-0.180830496	0.00088475	0.007232984
FAM90A20	-0.180820119	0.00088537	0.007234656
RP11-307C	0.180799507	0.00088659	0.007241297
RP11-361M	0.180762346	0.0008888	0.00725599
CGB2	0.180712764	0.00089176	0.007276765
MACC1	-0.180612319	0.00089778	0.007322251
CITF22-92A	-0.18060591	0.00089817	0.007322251
SPACA9	-0.180594645	0.00089885	0.007323389

C11orf54	-0.180589756	0.00089914	0.007323389
CBX7	-0.180545983	0.00090178	0.007341488
ORC2	0.180531649	0.00090265	0.007345137
SNHG16	-0.180515849	0.0009036	0.007349511
TDG	0.180507093	0.00090413	0.007350422
MOAP1	-0.180446692	0.00090779	0.007376789
TMEM89	0.18038117	0.00091178	0.007405779
THG1L	-0.180366816	0.00091266	0.007409472
RHOU	-0.180328608	0.00091499	0.007425005
GNS	-0.180319365	0.00091556	0.007426171
OPRL1	-0.180277595	0.00091812	0.00744351
RP11-303E	-0.180248122	0.00091993	0.007454756
C4orf36	-0.180210848	0.00092223	0.007469913
DDX46	0.180169989	0.00092475	0.007484987
RP3-477O4	0.180166911	0.00092494	0.007484987
NOA1	-0.180144351	0.00092633	0.007492832
CCNL1	0.180133642	0.000927	0.007494749
RANBP17	0.180111143	0.00092839	0.007502017
LINC01589	-0.180105381	0.00092875	0.007502017
TTC8	-0.180098069	0.0009292	0.007502238
NPIPA1	0.180087469	0.00092986	0.007503061
PIK3R3	0.180082692	0.00093016	0.007503061
LAMTOR3	-0.180069456	0.00093098	0.007506255
RP11-190C	0.180061182	0.00093149	0.007506964
RERG	-0.180016402	0.00093428	0.007525998
SMIM20	-0.179999291	0.00093535	0.007529717
PARP15	0.179995292	0.0009356	0.007529717
RP11-108M	0.17997497	0.00093687	0.007536493
AC144652.	0.179946428	0.00093866	0.007547414
PRPS1	0.179908296	0.00094105	0.007563189
RERG-IT1	-0.179869044	0.00094352	0.007579561
MAD2L2	0.179858851	0.00094416	0.007581256
RAD21	0.179812731	0.00094707	0.007600235
RN7SL535P	0.179801894	0.00094775	0.007600235
APOC1P1	0.179800856	0.00094782	0.007600235
RHOG	-0.179757164	0.00095058	0.00761894
CHP1	-0.179739688	0.00095169	0.007624349
WNT6	-0.179699026	0.00095428	0.007641566
AC026471.	-0.179676221	0.00095573	0.007646254
TBC1D2	-0.179676175	0.00095573	0.007646254
JAZF1	-0.179664719	0.00095646	0.007648616
LRRC61	0.179640439	0.00095801	0.007655578
RP11-101O	0.179637432	0.0009582	0.007655578
AC002128.	0.179588525	0.00096133	0.00767708
C3orf62	0.179570764	0.00096247	0.007682681
AC107081.	0.179525614	0.00096537	0.007699659
RP11-280O	0.179524006	0.00096547	0.007699659

POLL	-0.179501396	0.00096692	0.007703181
ATP6V0A1	-0.17949994	0.00096702	0.007703181
PFKFB1	0.179485472	0.00096795	0.007703181
ZRANB2	0.179484711	0.000968	0.007703181
IQCE	-0.179480718	0.00096826	0.007703181
PARK2	-0.17947635	0.00096854	0.007703181
TTLL7	0.179343757	0.00097712	0.007766729
ENDOD1	-0.179337289	0.00097755	0.007766729
OSBPL6	0.179332551	0.00097785	0.007766729
SOSTDC1	-0.179313366	0.0009791	0.007770004
PDCD5	0.179312648	0.00097915	0.007770004
RNF111	-0.179288591	0.00098072	0.00777894
ONECUT2	0.179229897	0.00098455	0.007805846
SETD7	-0.179188487	0.00098727	0.007823842
ANKRD18D	0.179173248	0.00098827	0.007828246
DCUN1D1	0.179138439	0.00099056	0.007842849
DLX3	-0.179126351	0.00099135	0.007845622
PRPF3	0.179073408	0.00099485	0.007869681
LY6G5B	0.179066742	0.00099529	0.007869681
GNG7	-0.179043326	0.00099684	0.007876111
AC008781.	0.179040925	0.000997	0.007876111
ZNF219	-0.179010588	0.00099901	0.007888457
KLHDC1	-0.178977506	0.00100121	0.007902262
RP11-353M	0.17894564	0.00100333	0.00791545
CTD-3131K	0.17893376	0.00100412	0.007918146
MYO1C	-0.178906495	0.00100594	0.007928938
CENPBD1	-0.178890615	0.001007	0.007933746
AP000251.	0.178873631	0.00100813	0.007938539
MFSD14C	0.178868028	0.00100851	0.007938539
NBR2	-0.178824437	0.00101143	0.007957977
CXCL13	0.178806955	0.00101261	0.007963652
RP11-181E	0.178719538	0.00101849	0.00800635
GNL2	0.178712875	0.00101894	0.00800635
PCK1	0.178688407	0.0010206	0.008015771
CPHL1P	0.178661071	0.00102245	0.00802673
TIMM50	0.178638172	0.001024	0.008035339
PWWP2B	-0.178628813	0.00102464	0.008036741
GFY	0.178592258	0.00102713	0.008052648
LRRC42	0.17858277	0.00102777	0.008052671
IMPDH1P1	0.178571272	0.00102855	0.008052671
C10orf54	-0.17856945	0.00102868	0.008052671
GNAT1	0.178565343	0.00102896	0.008052671
ZNF101	0.178542716	0.0010305	0.008060816
LSM4	0.178536666	0.00103092	0.008060816
CALML6	0.178500932	0.00103336	0.008076332
ZNF345	0.1784773	0.00103498	0.008085393
AC124789.	-0.178464522	0.00103585	0.00808582

RP11-521I2	0.1784631	0.00103595	0.00808582
LRRC32	-0.178419201	0.00103897	0.008102733
IQSEC2	-0.178418157	0.00103904	0.008102733
CTSA	-0.178332286	0.00104496	0.008143131
USP40	-0.178329648	0.00104515	0.008143131
NR1H2	-0.178261021	0.0010499	0.008176575
DKC1	0.178222097	0.00105261	0.008192049
LIPK	-0.178219064	0.00105282	0.008192049
PLEKHN1	-0.178170993	0.00105617	0.008214504
C7	-0.178148197	0.00105777	0.00822326
EPHA1	0.178135989	0.00105862	0.008226266
FANCM	0.178114394	0.00106013	0.008234382
IRS1	-0.178084011	0.00106227	0.008247297
PAX6	0.178034771	0.00106573	0.008270531
RP11-360L9	-0.178027991	0.00106621	0.008270589
RP4-669P1	-0.178020583	0.00106673	0.008270992
CEP57L1	0.177926945	0.00107335	0.00831865
FBXL4	-0.177919852	0.00107385	0.008318883
PIK3IP1	-0.177900026	0.00107526	0.008326116
PTTG1IP	-0.17788965	0.00107599	0.008328157
IL12RB2	0.177839245	0.00107958	0.008352245
ANKMY2	-0.177829616	0.00108027	0.008353249
BPIFB1	-0.177822331	0.00108079	0.008353249
CTC-339F2.	-0.177817453	0.00108114	0.008353249
AP001628.	0.177808421	0.00108178	0.008354562
KDSR	-0.177729793	0.00108741	0.00839434
CCL8	0.177683958	0.0010907	0.008416069
CBX5	0.177662904	0.00109222	0.00842407
SMPD4	0.177655237	0.00109277	0.008424637
RP11-667M	0.177627091	0.0010948	0.008436592
PRKAG2	0.177593594	0.00109722	0.008451545
RP11-677M	0.177572013	0.00109878	0.008459875
VGLL4	-0.177541001	0.00110103	0.008473479
ILKAP	0.177521383	0.00110246	0.008480733
C6orf223	0.17747662	0.00110571	0.008502069
WDR24	-0.177456199	0.0011072	0.008509797
RP11-977B	0.177340201	0.00111569	0.008571318
CDC42EP1	-0.177323213	0.00111694	0.008577165
ANKRD34A	-0.177316078	0.00111747	0.008577451
RP11-123B	-0.17723451	0.00112348	0.00861952
NEIL2	-0.177228497	0.00112393	0.00861952
AC144831.	-0.177213025	0.00112507	0.00862454
ERGIC3	-0.177145464	0.00113008	0.008655874
RP11-33E2.	0.177144662	0.00113014	0.008655874
RP11-19J3.	0.17712734	0.00113143	0.008661651
MPST	-0.177121286	0.00113188	0.008661651
PAXBP1	0.177101556	0.00113335	0.008669127

FDPSP8	0.177087412	0.00113441	0.008673422
RP11-575L7	0.177063736	0.00113617	0.008683162
CTSF	-0.176984385	0.00114212	0.008724784
VGLL1	-0.176951595	0.00114458	0.008738741
HADHB	-0.176946852	0.00114494	0.008738741
NOL8	0.176894134	0.00114891	0.008761922
APOC1	0.176888776	0.00114931	0.008761922
PEMT	-0.176886732	0.00114947	0.008761922
RASSF1	0.176849119	0.00115231	0.008779791
GTF3C5	0.176833607	0.00115349	0.008784935
CHAD	-0.17680691	0.00115551	0.008796541
AC007899.	0.176793205	0.00115655	0.008800652
TMEM61	-0.176776144	0.00115785	0.008806135
RP11-432I5	-0.176770548	0.00115827	0.008806135
RP11-197P	0.176720983	0.00116205	0.008831016
RBM43	-0.176685381	0.00116476	0.008847851
AL136115.1	0.176658711	0.0011668	0.008856358
SULT2B1	-0.176657578	0.00116689	0.008856358
BCAS3	-0.176644912	0.00116786	0.008857261
RNA5SP383	0.176642878	0.00116802	0.008857261
LINC00284	-0.176619401	0.00116982	0.008867091
RNU7-163P	0.176581871	0.0011727	0.008885117
BLOC1S4	-0.176490766	0.00117973	0.008934504
MBD5	-0.176456959	0.00118234	0.008946725
RPS9	-0.176456173	0.0011824	0.008946725
CORO1B	-0.176450222	0.00118287	0.008946725
RIC3	-0.176427128	0.00118466	0.008956425
RPL36A	0.176369662	0.00118913	0.008986349
SMIM10L2A	-0.176356473	0.00119015	0.008990255
AC019048.	0.176296012	0.00119488	0.009019712
PMS1	0.176293421	0.00119508	0.009019712
Z69720.3	-0.176217618	0.00120103	0.0090607
SLC34A2	-0.1762037	0.00120212	0.009065071
RNF170	-0.176174217	0.00120444	0.009075421
DGCR8	0.176173172	0.00120452	0.009075421
RP11-774O	-0.176144347	0.0012068	0.009088664
AP4M1	0.176105585	0.00120986	0.009106001
PABPN1	0.176102138	0.00121014	0.009106001
1-Sep	0.176076222	0.00121219	0.009117551
TYW5	0.176054018	0.00121395	0.009123375
RGS11	-0.176049208	0.00121433	0.009123375
SIK2	-0.176044194	0.00121473	0.009123375
FOXRED1	0.176040333	0.00121504	0.009123375
DOT1L	0.176003587	0.00121796	0.009139241
LGALS17A	0.175996488	0.00121853	0.009139241
Y_RNA.97	0.175994192	0.00121871	0.009139241
EGFEM1P	0.175907086	0.00122567	0.009187511

RP5-851M4	0.175898436	0.00122636	0.009188791
GNG12	-0.175878361	0.00122797	0.009196528
MAGI3	0.175872524	0.00122844	0.009196528
FAM65A	-0.175862818	0.00122922	0.009198448
RP13-93L1	0.175823771	0.00123236	0.009218022
PTPDC1	0.175809005	0.00123355	0.009222995
VMAC	-0.17568387	0.00124367	0.009294713
TEF	-0.175671143	0.0012447	0.009298488
USP39	0.175638744	0.00124734	0.009311146
BTF3L4	0.175632907	0.00124781	0.009311146
RP11-661A	0.175630792	0.00124798	0.009311146
RP11-399B	0.175605243	0.00125007	0.009322728
ANKRD37	-0.175540885	0.00125532	0.009351302
RP11-38M8	0.175538986	0.00125548	0.009351302
RP4-539M6	0.175532582	0.00125601	0.009351302
ORAI1	0.17553236	0.00125602	0.009351302
RP11-1084	0.175508906	0.00125795	0.009361655
UBE4B	-0.175493821	0.00125918	0.009366907
POT1	0.175474161	0.0012608	0.009374728
RP4-736L2	0.175468056	0.0012613	0.009374728
C9orf172	-0.175450341	0.00126276	0.009381598
RP11-495P	-0.175436544	0.0012639	0.009386077
USP49	0.175380837	0.00126849	0.009412991
WIPI2	-0.175379668	0.00126859	0.009412991
RAN	0.175318892	0.00127362	0.009446357
GNA12	-0.175288358	0.00127616	0.009461177
ZBED6CL	0.175257078	0.00127876	0.009476478
CYB561	-0.175233031	0.00128077	0.009487334
VWA8	-0.175189363	0.00128441	0.009507858
CSRNP3	-0.175186915	0.00128462	0.009507858
TMEM184A	-0.175155604	0.00128724	0.009523251
RN7SL481P	0.17514242	0.00128834	0.00952742
RPS23	-0.1751238	0.00128991	0.009534965
GCHFR	0.175070428	0.00129439	0.00956412
snoU13.36	0.175042636	0.00129674	0.009577406
ACTG1P20	0.175033838	0.00129748	0.009578868
METTL25	-0.175021809	0.00129849	0.009582345
DNM3	-0.174990092	0.00130118	0.009592815
C11orf45	-0.174986041	0.00130152	0.009592815
DBF4B	0.174985684	0.00130155	0.009592815
RP11-387A	0.174977823	0.00130221	0.009593701
RP11-400F	0.174964826	0.00130332	0.009597794
RP11-672A	-0.174943356	0.00130514	0.00960718
AP000640.	0.174925267	0.00130667	0.00961351
RP11-103J8	0.174917122	0.00130736	0.00961351
CTC-359D2	0.174913931	0.00130763	0.00961351
PITPNB	0.174879925	0.00131053	0.00963076

GDPD5	-0.174871351	0.00131126	0.009632105
RNF135	-0.174838303	0.00131408	0.009648786
AC093818.	0.174821674	0.0013155	0.009651602
VASN	-0.174813774	0.00131617	0.009651602
RP11-717D	0.174812174	0.00131631	0.009651602
FAM133B	0.17480814	0.00131666	0.009651602
MTHFSD	-0.174748774	0.00132174	0.009684861
ACTR3C	0.174711014	0.00132499	0.009704415
PSEN2	-0.174704898	0.00132552	0.009704415
KHDC1	0.174669295	0.00132858	0.009722828
KIAA0556	-0.174656885	0.00132965	0.00972662
B3GNT5	0.174597364	0.0013348	0.009760214
CEP97	0.174588436	0.00133558	0.009761813
GEMIN2	0.174577233	0.00133655	0.009764856
RP11-290L	0.174539597	0.00133981	0.009783138
FAM174A	-0.174535611	0.00134016	0.009783138
BNIP3P37	0.174520937	0.00134144	0.009788395
KSR2	-0.174508608	0.00134251	0.009792166
CTB-186G2	0.174485202	0.00134455	0.009798945
PDGFA	-0.17448516	0.00134455	0.009798945
CTB-161M	-0.17447499	0.00134544	0.009798951
CDK16	0.174467459	0.0013461	0.009798951
RP11-575F	-0.174466002	0.00134623	0.009798951
HERC5	0.174457496	0.00134697	0.009800304
RP11-881M	-0.174447608	0.00134783	0.009802536
FZD9	0.174415107	0.00135068	0.009819157
RP11-343L	0.17439355	0.00135257	0.009828829
BEAN1	-0.17432017	0.00135901	0.009871613
SYDE1	-0.174304876	0.00136036	0.009877323
HSD11B2	0.174292109	0.00136149	0.009878529
UEVLD	-0.174290258	0.00136165	0.009878529
RAD18	0.174279807	0.00136257	0.009881146
RP4-758J1	0.174229757	0.001367	0.009902224
GOLGA2P5	0.17422961	0.00136701	0.009902224
CTNNAL1	0.174226881	0.00136725	0.009902224
NDUFA3P4	0.174221483	0.00136773	0.009902224
PMEPA1	-0.174198009	0.00136981	0.009913211
RP11-484D	0.174179774	0.00137143	0.009920843
RP11-326L	0.174150579	0.00137403	0.009935531
TCEAL4	-0.174129561	0.0013759	0.00994497
RNF187	-0.17412322	0.00137646	0.00994497
PDPK2P	-0.174063794	0.00138177	0.009979199
RP11-395L	-0.17405104	0.00138291	0.009983341
SETP17	0.17403651	0.00138421	0.009988634
CCT8L2	0.174017723	0.00138589	0.009996683

Table S6: Genes with significant (P<0.05) mRNA differential correlation (r2) with EZH2 in platinum resistant (PR) versus platinum sensitive (PS) HGOSC (TCGA)

Gene	PS_r2	PS_pVal	PR_r2	PR_pVal	zScoreDiff	pValDiff	empPVals	pValDif	Classes
C16orf89	0.19	0.008167	-0.38	0.000509	-4.38	1.17E-05	1.92E-05	0.45	+/-
LINS1	0.21	0.002918	-0.33	0.003009	-4.12	3.86E-05	3.84E-05	0.45	+/-
RP4-569D19.5	0.16	0.026064	-0.37	0.000747	-4.07	4.78E-05	4.48E-05	0.45	+/-
OR7E89P	0.23	0.001276	-0.30	0.007046	-4.01	6.03E-05	5.76E-05	0.45	+/-
PHC1P1	-0.15	0.032497	0.35	0.001694	3.82	0.000135	0.000115	0.72	-/+
C21orf33	-0.22	0.001836	0.28	0.013555	3.76	0.00017	0.00015	0.7833	-/+
CTB-60B18.10	-0.15	0.034864	0.33	0.002875	3.66	0.000249	0.000246	0.9992	-/+
RP11-1252D15.	-0.07	0.313849	0.40	0.000304	3.63	0.000286	0.000285	0.9992	0/+
C2	0.09	0.201939	-0.38	0.000697	-3.58	0.000349	0.000343	0.9992	0/-
OR7E28P	0.16	0.022055	-0.31	0.006298	-3.54	0.000395	0.000387	0.9992	+/-
RP11-371A22.1	-0.29	5.59E-05	0.18	0.120645	3.47	0.000522	0.00049	0.9992	-/0
RP11-110G21.1	-0.24	0.000697	0.22	0.054901	3.44	0.000588	0.000547	0.9992	-/0
OR51E1	-0.10	0.162022	0.35	0.001608	3.43	0.000593	0.000547	0.9992	0/+
DYNC1I1	-0.15	0.031611	0.29	0.009305	3.36	0.000793	0.000797	0.9992	-/+
ARPIN	0.17	0.019786	-0.28	0.013044	-3.35	0.000807	0.00081	0.9992	+/-
RP11-697E2.12	0.31	1.48E-05	-0.14	0.22528	-3.35	0.000822	0.000826	0.9992	+/0
ZNF77	-0.11	0.142651	0.34	0.002617	3.34	0.000823	0.000826	0.9992	0/+
RP11-363E7.4	0.16	0.022252	-0.28	0.013355	-3.32	0.000902	0.000903	0.9992	+/-
RP5-968J1.1	-0.19	0.007098	0.25	0.028572	3.29	0.000994	0.000973	0.9992	-/+
ARMC6	0.04	0.560914	0.45	2.88E-05	3.29	0.001005	0.000976	0.9992	0/+
BRF2	-0.17	0.02133	0.27	0.015743	3.28	0.001049	0.001024	0.9992	-/+
KCNV2	0.15	0.04154	-0.29	0.010964	-3.25	0.001166	0.001146	0.9992	+/-
LINC01060	-0.09	0.206695	0.33	0.002758	3.22	0.00127	0.001255	0.9992	0/+
TMCC2	-0.17	0.020167	0.26	0.01951	3.22	0.001281	0.001261	0.9992	-/+
ILVBL	-0.14	0.050109	0.29	0.010878	3.21	0.001341	0.001306	0.9992	0/+
FAM66B	-0.20	0.005805	0.23	0.040895	3.20	0.001356	0.001316	0.9992	-/+
DDB2	0.23	0.001041	-0.19	0.087401	-3.20	0.001386	0.001348	0.9992	+/0
ZNF491	-0.04	0.548648	0.37	0.000788	3.19	0.001437	0.001399	0.9992	0/+
RP11-15B17.1	-0.25	0.000478	0.18	0.12275	3.17	0.001516	0.00145	0.9992	-/0
AC113608.1	-0.15	0.031888	0.27	0.017271	3.16	0.001555	0.001495	0.9992	-/+
RP4-646N3.1	-0.03	0.692807	0.38	0.000607	3.14	0.001684	0.001597	0.9992	0/+
C9orf152	0.15	0.043526	-0.27	0.015136	-3.14	0.001703	0.001617	0.9992	+/-
RP1-122P22.4	-0.20	0.00455	0.22	0.05626	3.13	0.001746	0.001658	0.9992	-/0
RPL12P13	-0.14	0.055345	0.28	0.013577	3.12	0.001829	0.001741	0.9992	0/+
RP11-332H18.7	0.10	0.16449	-0.31	0.005232	-3.12	0.001833	0.001745	0.9992	0/-
**POLR2F**	0.00	0.955051	-0.40	0.000323	-3.11	0.001884	0.001802	0.9992	0/-
RP11-250B2.6	0.05	0.524404	-0.36	0.001182	-3.11	0.001889	0.001812	0.9992	0/-
CTD-2199O4.6	0.22	0.001769	-0.19	0.091724	-3.10	0.001963	0.001866	0.9992	+/0
UBXN2B	-0.11	0.115094	0.30	0.007963	3.09	0.001968	0.00187	0.9992	0/+
SUMO2P15	0.19	0.008614	-0.23	0.045648	-3.09	0.001974	0.001873	0.9992	+/-
WI2-80269A6.1	0.11	0.119877	0.49	5.57E-06	3.09	0.001978	0.001873	0.9992	0/+
CKLF-CMTM1	-0.17	0.015567	0.24	0.034386	3.08	0.002052	0.001924	0.9992	-/+
ZNF57	-0.15	0.032482	0.26	0.022147	3.08	0.002068	0.001953	0.9992	-/+

RP11-61J19.4	-0.27	0.000195	0.15	0.198916	3.08	0.002087	0.001966	0.9992	-/0
CERS4	-0.11	0.119198	0.30	0.008383	3.07	0.002136	0.00201	0.9992	0/+
AP000344.4	0.16	0.023905	-0.25	0.028247	-3.06	0.002184	0.002049	0.9992	+/-
BACH1-IT2	0.23	0.001011	-0.18	0.124677	-3.05	0.002257	0.002161	0.9992	+/0
SPIDR	-0.05	0.455824	0.35	0.00185	3.05	0.002274	0.002174	0.9992	0/+
THEGL	-0.16	0.029847	0.25	0.026088	3.04	0.002332	0.002228	0.9992	-/+
RP11-798G7.8	0.12	0.093575	-0.28	0.01194	-3.03	0.002459	0.002359	0.9992	0/-
RP11-248J18.2	0.08	0.266455	-0.32	0.004274	-3.02	0.002497	0.002379	0.9992	0/-
RN7SL382P	0.21	0.003575	-0.20	0.082539	-3.02	0.002497	0.002379	0.9992	+/0
GHDC	-0.20	0.005383	-0.55	2.24E-07	-3.02	0.002559	0.002427	0.9992	-/-
GPR157	-0.19	0.008406	0.22	0.058248	3.01	0.002622	0.002462	0.9992	-/0
RP11-505K9.5	0.02	0.816241	0.40	0.000257	3.01	0.002641	0.002481	0.9992	0/+
CLEC12A	0.09	0.204075	-0.31	0.006314	-3.00	0.002709	0.002558	0.9992	0/-
LINC01389	0.21	0.003533	-0.19	0.09184	-2.98	0.002857	0.002747	0.9992	+/0
RP11-423H2.1	0.09	0.231642	-0.31	0.005915	-2.98	0.002899	0.002775	0.9992	0/-
SKIDA1	-0.14	0.052036	0.26	0.022294	2.97	0.002943	0.002833	0.9992	0/+
PTPN3	0.07	0.319222	-0.32	0.004107	-2.97	0.002946	0.002836	0.9992	0/-
CTD-2545G14.6	0.01	0.91611	0.39	0.0004	2.97	0.002947	0.002836	0.9992	0/+
ZNF729	0.03	0.668737	0.41	0.000193	2.97	0.00301	0.0029	0.9992	0/+
RGS7	0.04	0.599086	-0.35	0.001648	-2.96	0.003032	0.002916	0.9992	0/-
ITIH6	-0.14	0.044876	0.25	0.025605	2.96	0.00306	0.002948	0.9992	-/+
ZNF561	-0.15	0.035747	0.25	0.030558	2.95	0.003153	0.003044	0.9992	-/+
ICAM5	-0.26	0.000234	0.13	0.243538	2.95	0.003155	0.003044	0.9992	-/0
ZSCAN12P1	0.14	0.051763	0.50	4.00E-06	2.95	0.003194	0.003086	0.9992	0/+
RP1-16A9.1	-0.01	0.861068	0.37	0.000866	2.94	0.0033	0.003214	0.9992	0/+
RP11-644F5.10	0.15	0.040903	-0.25	0.02979	-2.93	0.003373	0.003288	0.9992	+/-
GRAPL	-0.08	0.253999	0.31	0.006369	2.93	0.003415	0.00331	0.9992	0/+
RP11-392O1.4	0.00	0.967144	-0.38	0.000675	-2.93	0.003416	0.00331	0.9992	0/-
RP11-879F14.2	-0.09	0.198133	0.30	0.008563	2.92	0.003502	0.003384	0.9992	0/+
RP11-219B4.3	-0.04	0.550512	0.34	0.002291	2.92	0.00353	0.003416	0.9992	0/+
LHB	-0.01	0.860058	0.37	0.000958	2.91	0.003564	0.003438	0.9992	0/+
NCR2	0.06	0.39365	-0.32	0.003875	-2.91	0.003578	0.003448	0.9992	0/-
TBL1XR1-AS1	0.16	0.027076	-0.23	0.041014	-2.91	0.003622	0.003477	0.9992	+/-
CTD-2020K17.1	0.15	0.042937	-0.24	0.030967	-2.91	0.003643	0.003496	0.9992	+/-
FTH1P2	0.03	0.629085	-0.35	0.00189	-2.91	0.003653	0.003509	0.9992	0/-
SCN9A	0.20	0.006211	-0.19	0.090905	-2.89	0.003854	0.003691	0.9992	+/0
MIR5190	0.18	0.012448	-0.21	0.066082	-2.89	0.003874	0.003701	0.9992	+/0
TGFBR3	-0.11	0.132948	0.28	0.014014	2.89	0.003897	0.003723	0.9992	0/+
RP11-397J20.1	-0.08	0.242419	0.30	0.007824	2.88	0.003934	0.003755	0.9992	0/+
TXNDC16	0.08	0.262088	0.44	5.20E-05	2.88	0.003967	0.003784	0.9992	0/+
AL359922.1	0.23	0.001315	-0.16	0.169553	-2.88	0.004024	0.003841	0.9992	+/0
CFD	0.08	0.273869	-0.30	0.007013	-2.88	0.004036	0.003848	0.9992	0/-
CARM1	-0.05	0.493452	0.33	0.003216	2.87	0.004051	0.00387	0.9992	0/+
DUS3L	-0.09	0.226278	0.30	0.008743	2.87	0.004069	0.003883	0.9992	0/+
AC016995.3	-0.07	0.314147	0.31	0.006021	2.87	0.004076	0.003886	0.9992	0/+
ITGAD	-0.06	0.398162	0.32	0.004465	2.87	0.004105	0.003912	0.9992	0/+
ARNTL2	0.47	4.56E-12	0.12	0.292838	-2.86	0.004178	0.003998	0.9992	+/0

XIAP-AS1	0.21	0.00289	-0.17	0.131902	-2.86	0.004188	0.004008	0.9992	+/0
RP11-1060J15.5	0.16	0.030683	-0.23	0.043542	-2.86	0.004214	0.004066	0.9992	+/-
NDUFA7	0.02	0.745516	0.39	0.000393	2.86	0.004226	0.004082	0.9992	0/+
RP11-453E17.4	-0.08	0.279467	0.30	0.007235	2.86	0.004242	0.004091	0.9992	0/+
GPD1	0.18	0.011975	-0.20	0.073406	-2.85	0.004304	0.004162	0.9992	+/0
YTHDF3	-0.13	0.062651	0.25	0.028287	2.85	0.004353	0.00421	0.9992	0/+
ARHGEF19	-0.09	0.199715	0.29	0.010745	2.85	0.004372	0.004232	0.9992	0/+
MAU2	-0.18	0.014421	0.21	0.067984	2.85	0.004383	0.004251	0.9992	-/0
CDCP1	0.18	0.012465	-0.20	0.073645	-2.85	0.004425	0.004274	0.9992	+/0
RP11-293A21.1	-0.14	0.050892	0.24	0.033148	2.85	0.00443	0.004277	0.9992	0/+
TUB	-0.25	0.000374	0.13	0.264014	2.84	0.004447	0.004293	0.9992	-/0
RP11-686D22.5	0.12	0.099988	-0.26	0.020344	-2.84	0.004449	0.004293	0.9992	0/-
INSM2	0.19	0.008834	-0.19	0.088018	-2.84	0.004517	0.004379	0.9992	+/0
HLA-L	0.20	0.004352	-0.18	0.122472	-2.83	0.004696	0.004504	0.9992	+/0
CLEC12B	0.17	0.01949	-0.21	0.062784	-2.82	0.004797	0.004616	0.9992	+/0
SAPCD1-AS1	0.06	0.446467	-0.32	0.004586	-2.82	0.00483	0.004667	0.9992	0/-
RASSF8	0.12	0.0972	-0.26	0.022924	-2.81	0.00491	0.004754	0.9992	0/-
GPAT4	-0.17	0.02126	0.21	0.061253	2.81	0.00492	0.004773	0.9992	-/0
AL022341.1	0.14	0.046143	-0.23	0.039425	-2.81	0.00499	0.004827	0.9992	+/-
TSPAN10	-0.16	0.030947	0.22	0.05066	2.81	0.005031	0.004879	0.9992	-/0
RNU6-824P	0.11	0.143237	-0.27	0.017238	-2.80	0.005096	0.004962	0.9992	0/-
AP000266.7	-0.03	0.634276	0.33	0.002793	2.80	0.0051	0.004975	0.9992	0/+
METTL7B	0.03	0.638845	-0.33	0.002855	-2.79	0.005248	0.005144	0.9992	0/-
OR7E90P	0.15	0.035724	-0.22	0.048508	-2.79	0.00527	0.00516	0.9992	+/-
LNP1	-0.10	0.162919	0.27	0.015893	2.79	0.005271	0.00516	0.9992	0/+
RP13-270P17.2	-0.23	0.00115	0.14	0.212315	2.79	0.005277	0.00517	0.9992	-/0
RP11-244H3.1	0.10	0.167166	-0.27	0.015625	-2.79	0.005305	0.005199	0.9992	0/-
CCDC144NL-AS	-0.07	0.31952	0.30	0.007912	2.79	0.005306	0.005202	0.9992	0/+
CTA-363E6.1	0.12	0.083308	-0.25	0.027993	-2.79	0.005343	0.005228	0.9992	0/-
AP001065.15	0.03	0.644882	0.39	0.000399	2.78	0.005356	0.00524	0.9992	0/+
RP11-88E10.4	0.21	0.003947	-0.17	0.140893	-2.78	0.005393	0.005279	0.9992	+/0
DDX49	-0.03	0.637314	0.33	0.002988	2.78	0.005427	0.005298	0.9992	0/+
RAB27A	0.19	0.008618	-0.19	0.102974	-2.78	0.005436	0.005314	0.9992	+/0
C22orf34	-0.07	0.309187	0.30	0.008472	2.78	0.005448	0.005333	0.9992	0/+
snoU13.32	0.01	0.872651	0.37	0.000817	2.77	0.00553	0.005452	0.9992	0/+
PCAT6	-0.33	3.24E-06	0.04	0.744159	2.77	0.005572	0.005497	0.9992	-/0
RP11-379B8.1	-0.13	0.068484	0.24	0.033554	2.77	0.005573	0.0055	0.9992	0/+
AP3S2	0.06	0.428032	-0.31	0.005835	-2.77	0.005651	0.005583	0.9992	0/-
HTR1E	-0.23	0.001579	0.15	0.204614	2.76	0.005807	0.005756	0.9992	-/0
HAUS8	0.19	0.007776	0.52	1.40E-06	2.76	0.005809	0.005756	0.9992	+/+
CTD-3214K23.1	0.05	0.511999	0.40	0.000287	2.76	0.005836	0.005785	0.9992	0/+
ALOX15	-0.10	0.182339	0.27	0.015936	2.76	0.005846	0.005791	0.9992	0/+
ADPRHL1	-0.06	0.421954	-0.41	0.000207	-2.75	0.005919	0.005858	0.9992	0/-
RPS2P41	0.25	0.000345	-0.11	0.321615	-2.75	0.005971	0.005922	0.9992	+/0
CMBL	0.14	0.055673	-0.23	0.041258	-2.75	0.005985	0.005938	0.9992	0/-
CTD-2192J16.2(	-0.13	0.071668	0.24	0.034973	2.75	0.006021	0.00598	0.9992	0/+
KAT6A	-0.22	0.001701	0.15	0.205176	2.75	0.006041	0.006009	0.9992	-/0

OR56B4	-0.22	0.001995	0.15	0.195289	2.74	0.006068	0.006047	0.9992	-/0
C1QTNF3-AMA	0.05	0.485908	0.40	0.000277	2.74	0.006096	0.006102	0.9992	0/+
FBXL12	-0.05	0.448173	0.31	0.005996	2.74	0.006116	0.006127	0.9992	0/+
DDHD2	-0.20	0.004562	0.17	0.146586	2.74	0.006139	0.006137	0.9992	-/0
KB-1507C5.4	0.00	0.947759	-0.35	0.001529	-2.74	0.006164	0.006172	0.9992	0/-
VDR	0.15	0.035528	-0.22	0.055989	-2.74	0.006173	0.006188	0.9992	+/0
CTD-2308L22.1	-0.27	0.000122	0.09	0.418117	2.74	0.006178	0.006191	0.9992	-/0
LINC01356	-0.10	0.163158	0.27	0.018704	2.74	0.006181	0.006191	0.9992	0/+
LYPLAL1-AS1	-0.18	0.014127	0.19	0.091296	2.74	0.006204	0.00622	0.9992	-/0
CTC-436P18.3	-0.03	0.653996	-0.38	0.000506	-2.74	0.006219	0.006242	0.9992	0/-
RP11-278A23.1	-0.05	0.522038	0.32	0.004944	2.73	0.006278	0.006287	0.9992	0/+
RP4-726N1.2	-0.23	0.001518	0.14	0.218884	2.73	0.006303	0.006306	0.9992	-/0
RP11-421F16.3	0.05	0.50454	-0.31	0.005281	-2.73	0.006355	0.006358	0.9992	0/-
RP11-288G3.4	-0.20	0.006105	0.17	0.135291	2.73	0.006426	0.006434	0.9992	-/0
SUGP1	-0.10	0.168535	0.27	0.01891	2.73	0.006427	0.006434	0.9992	0/+
ZKSCAN2	-0.23	0.001584	0.14	0.219532	2.72	0.006459	0.006479	0.9992	-/0
RP11-229P13.2	0.08	0.258707	0.42	0.000109	2.72	0.006538	0.006572	0.9992	0/+
THSD4	0.05	0.507943	-0.31	0.005453	-2.72	0.006587	0.00662	0.9992	0/-
RNU6-247P	0.03	0.654452	-0.33	0.003611	-2.72	0.00659	0.00662	0.9992	0/-
S100A10	-0.01	0.913304	-0.36	0.001156	-2.72	0.006597	0.006627	0.9992	0/-
RP11-316M1.3	-0.05	0.496851	0.31	0.005648	2.72	0.006599	0.00663	0.9992	0/+
RP11-556O9.3	0.06	0.3844	-0.30	0.008115	-2.71	0.006655	0.006694	0.9992	0/-
CTC-270D5.1	-0.07	0.366544	0.30	0.008683	2.71	0.006699	0.006735	0.9992	0/+
SNORD93	-0.15	0.0355	0.21	0.060521	2.71	0.00674	0.006771	0.9992	-/0
NPM1P9	0.00	0.974383	0.35	0.001606	2.71	0.006754	0.006777	0.9992	0/+
RP11-1060J15.4	0.13	0.067945	-0.23	0.040487	-2.71	0.006765	0.006793	0.9992	0/-
RBMS1P1	-0.07	0.325922	0.29	0.010142	2.71	0.006776	0.006806	0.9992	0/+
RPL9P16	0.15	0.038949	-0.22	0.057758	-2.71	0.006794	0.006831	0.9992	+/0
LINC00189	-0.09	0.22312	0.27	0.015455	2.70	0.006841	0.006876	0.9992	0/+
FAM175A	-0.37	1.31E-07	-0.02	0.872212	2.70	0.006886	0.006918	0.9992	-/0
ARMC1	-0.01	0.879476	0.34	0.002121	2.70	0.006919	0.006953	0.9992	0/+
RP4-569D19.8	0.05	0.479599	-0.31	0.006321	-2.70	0.006953	0.006975	0.9992	0/-
GOLGA7	-0.08	0.279788	0.28	0.012497	2.70	0.006997	0.007023	0.9992	0/+
NCOA5	-0.15	0.03471	0.21	0.063442	2.70	0.007008	0.007027	0.9992	-/0
RP1-128M12.3	0.13	0.064389	-0.23	0.043413	-2.70	0.007013	0.007036	0.9992	0/-
RP11-807H22.7	-0.16	0.029288	0.21	0.070207	2.69	0.007062	0.007084	0.9992	-/0
EEF1A1P6	-0.16	0.024747	0.20	0.077027	2.69	0.007083	0.007107	0.9992	-/0
SIGLEC18P	-0.09	0.230137	0.27	0.015565	2.69	0.007091	0.00712	0.9992	0/+
TOLLIP-AS1	-0.43	4.92E-10	-0.09	0.42617	2.69	0.007152	0.007164	0.9992	-/0
TMEM161A	-0.14	0.046623	0.22	0.054551	2.69	0.007189	0.007196	0.9992	-/0
PPP1R1B	0.16	0.031179	-0.21	0.069232	-2.69	0.00723	0.007228	0.9992	+/0
FOXA2	0.19	0.006839	-0.17	0.141321	-2.69	0.007235	0.007241	0.9992	+/0
RP11-63M22.1	0.12	0.083487	-0.24	0.037534	-2.68	0.007265	0.00727	0.9992	0/-
TP53TG3D	-0.07	0.306248	0.28	0.01186	2.68	0.007313	0.007299	0.9992	0/+
SPG20	-0.26	0.000214	0.10	0.405657	2.68	0.007345	0.007334	0.9992	-/0
RP11-624M8.1	-0.15	0.037883	0.21	0.062996	2.68	0.007359	0.00734	0.9992	-/0
CTD-2012J19.2	0.07	0.342341	-0.29	0.010571	-2.68	0.007411	0.007395	0.9992	0/-

RP11-355N15.3	0.08	0.264609	-0.28	0.014127	-2.68	0.007411	0.007395	0.9992	0/-
TRAPPC10	-0.08	0.270788	0.28	0.013865	2.68	0.007452	0.007414	0.9992	0/+
EPS15L1	-0.08	0.288946	0.28	0.012924	2.68	0.007454	0.007417	0.9992	0/+
LINC01165	-0.11	0.118505	0.25	0.029779	2.67	0.007502	0.007456	0.9992	0/+
RP11-236L14.1	-0.11	0.142498	0.25	0.025737	2.67	0.007533	0.007481	0.9992	0/+
GOLGA2P5	0.29	4.81E-05	-0.07	0.558129	-2.67	0.007594	0.00752	0.9992	+/0
ADAM32	-0.20	0.00557	0.16	0.159031	2.67	0.00761	0.007529	0.9992	-/0
AP000708.1	0.02	0.804573	-0.33	0.002917	-2.67	0.007635	0.007574	0.9992	0/-
ST3GAL5-AS1	-0.18	0.013067	0.18	0.112081	2.67	0.007657	0.007603	0.9992	-/0
PGM1	0.02	0.734436	-0.33	0.003513	-2.67	0.00766	0.007613	0.9992	0/-
TMED1	-0.10	0.154953	0.25	0.024393	2.67	0.007665	0.007622	0.9992	0/+
URAHP	-0.25	0.000527	0.11	0.338348	2.66	0.007805	0.007763	0.9992	-/0
SCARNA11	0.03	0.722378	0.37	0.000853	2.66	0.00781	0.007763	0.9992	0/+
RP11-563J2.3	0.13	0.066878	-0.23	0.047296	-2.66	0.0079	0.007881	0.9992	0/-
RASSF8-AS1	0.12	0.097163	-0.24	0.03645	-2.66	0.007901	0.007881	0.9992	0/-
CLNK	0.09	0.200285	-0.26	0.020002	-2.66	0.00791	0.007904	0.9992	0/-
AKR1C6P	-0.04	0.567847	0.31	0.005717	2.66	0.007921	0.007917	0.9992	0/+
CTD-2342J14.6	-0.12	0.100655	0.24	0.035636	2.66	0.007931	0.007926	0.9992	0/+
SEMA6D	-0.13	0.079781	0.23	0.042801	2.65	0.008066	0.008057	0.9992	0/+
RP11-718B12.2	-0.11	0.11303	0.24	0.033259	2.65	0.008084	0.008074	0.9992	0/+
MCOLN1	-0.16	0.023566	0.19	0.088392	2.65	0.008092	0.00809	0.9992	-/0
RP11-779O18.2	-0.06	0.397563	0.29	0.00973	2.65	0.00811	0.008112	0.9992	0/+
RP3-425C14.4	0.01	0.853836	-0.33	0.002784	-2.65	0.008128	0.008131	0.9992	0/-
RP11-94H18.1	0.07	0.318657	-0.28	0.012756	-2.65	0.008139	0.008141	0.9992	0/-
CTC-591M7.1	0.20	0.005925	-0.16	0.164818	-2.64	0.00825	0.008243	0.9992	+/0
RP11-88I21.1	-0.03	0.724487	0.32	0.003965	2.64	0.00827	0.008269	0.9992	0/+
RP11-807C20.2	0.18	0.010472	-0.17	0.131557	-2.64	0.008297	0.008294	0.9992	+/0
IFT80	0.19	0.006722	-0.16	0.158031	-2.64	0.008325	0.008326	0.9992	+/0
RP11-337N6.3	-0.01	0.923133	-0.35	0.001618	-2.64	0.008327	0.00833	0.9992	0/-
LL0XNC01-116E	-0.03	0.684501	0.32	0.004461	2.64	0.008362	0.008368	0.9992	0/+
UGCG	0.18	0.01063	-0.17	0.131979	-2.64	0.0084	0.0084	0.9992	+/0
RNU7-45P	0.17	0.015941	-0.18	0.110553	-2.63	0.008432	0.008426	0.9992	+/0
AC003956.1	-0.02	0.791379	0.33	0.003436	2.63	0.008464	0.008445	0.9992	0/+
CTA-390C10.9	-0.12	0.100674	0.23	0.038743	2.63	0.008646	0.008624	0.9992	0/+
EEF1A1P5	-0.17	0.020413	0.19	0.101092	2.62	0.008727	0.008717	0.9992	-/0
MIPEPP3	-0.08	0.242348	0.27	0.018509	2.62	0.008758	0.008746	0.9992	0/+
ARHGAP19	-0.10	0.151696	0.25	0.028504	2.62	0.008779	0.008755	0.9992	0/+
LDB1	-0.29	3.52E-05	0.06	0.630695	2.62	0.008786	0.008765	0.9992	-/0
GNRH2	-0.12	0.103544	0.23	0.038736	2.62	0.008832	0.008819	0.9992	0/+
TEX37	-0.03	0.704983	0.32	0.004602	2.61	0.008955	0.00896	0.9992	0/+
DCST1	0.19	0.009659	-0.17	0.144877	-2.61	0.008998	0.009002	0.9992	+/0
TAF9P3	-0.13	0.081676	0.23	0.047199	2.61	0.0091	0.009156	0.9992	0/+
GHc-362H12.3	-0.05	0.501934	0.30	0.008151	2.61	0.009137	0.009213	0.9992	0/+
PCDHGB3	-0.21	0.004092	0.15	0.203117	2.61	0.009138	0.009213	0.9992	-/0
CDC34	-0.12	0.092018	0.23	0.043724	2.61	0.009164	0.009229	0.9992	0/+
RP11-546D6.3	0.41	2.15E-09	0.09	0.457491	-2.60	0.009243	0.009277	0.9992	+/0
OR56A5	-0.17	0.018988	0.18	0.10976	2.60	0.009247	0.009284	0.9992	-/0

ZNF559	0.00	0.979204	0.34	0.002424	2.60	0.009344	0.009335	0.9992	0/+
FBXO4	0.20	0.006502	-0.16	0.174709	-2.60	0.00937	0.00936	0.9992	+/0
CASP12	-0.19	0.006791	0.16	0.172829	2.60	0.009443	0.009453	0.9992	-/0
RP4-561L24.3	-0.20	0.004336	0.15	0.204271	2.59	0.00948	0.009501	0.9992	-/0
ELL	-0.16	0.024313	0.19	0.099585	2.59	0.009509	0.009533	0.9992	-/0
NRSN1	0.15	0.041662	-0.20	0.074775	-2.59	0.00951	0.00954	0.9992	+/0
MIR4668	0.18	0.013739	-0.17	0.130942	-2.59	0.009585	0.009626	0.9992	+/0
SNORA14A	-0.03	0.663044	0.31	0.005625	2.59	0.009653	0.009716	0.9992	0/+
SYN1	0.12	0.090707	-0.23	0.046482	-2.59	0.009668	0.009722	0.9992	0/-
LRRFIP2	0.17	0.01554	-0.18	0.124868	-2.59	0.009674	0.009729	0.9992	+/0
RPS15AP12	-0.21	0.003098	0.14	0.233263	2.59	0.009736	0.009786	0.9992	-/0
RP4-777L9.2	-0.14	0.048526	0.21	0.069945	2.58	0.009742	0.009796	0.9992	-/0
RP11-142A23.1	-0.12	0.087298	0.22	0.048667	2.58	0.009865	0.009914	0.9992	0/+
RP11-16E12.1	-0.01	0.897643	-0.35	0.001905	-2.58	0.009891	0.009943	0.9992	0/-
FAR2P4	-0.07	0.368616	0.28	0.01347	2.58	0.009957	0.010023	0.9992	0/+
DDX11L5	-0.02	0.815622	0.32	0.004027	2.57	0.010076	0.010148	0.9992	0/+
HOXA11-AS	0.15	0.04156	-0.20	0.078977	-2.57	0.010105	0.010174	0.9992	+/0
ZNF439	-0.05	0.528148	0.30	0.008537	2.57	0.01012	0.010193	0.9992	0/+
RNA5SP219	0.08	0.283062	-0.27	0.018539	-2.57	0.010172	0.01027	0.9992	0/-
RP1-91J24.3	0.22	0.002067	-0.13	0.273294	-2.57	0.010194	0.010308	0.9992	+/0
ZNF426	-0.10	0.153877	0.24	0.033078	2.57	0.010294	0.010423	0.9992	0/+
ZNF708	-0.05	0.485949	0.29	0.009889	2.56	0.010368	0.010494	0.9992	0/+
AP3M2	-0.03	0.678381	0.31	0.005933	2.56	0.010415	0.010551	0.9992	0/+
WDR88	-0.18	0.013674	0.17	0.140396	2.56	0.010446	0.010577	0.9992	-/0
RPL23AP81	0.15	0.037625	-0.20	0.085909	-2.56	0.010447	0.010577	0.9992	+/0
LINC01140	-0.19	0.008698	0.16	0.169484	2.56	0.010467	0.010599	0.9992	-/0
Y_RNA.306	0.17	0.015957	-0.17	0.134342	-2.55	0.010764	0.010891	0.9992	+/0
RP4-671O14.7	0.11	0.144881	-0.24	0.036442	-2.55	0.010795	0.010932	0.9992	0/-
ARHGEF35	0.20	0.005809	-0.15	0.202209	-2.55	0.010817	0.010955	0.9992	+/0
RP11-39M21.2	-0.01	0.889069	-0.34	0.002118	-2.55	0.010882	0.011012	0.9992	0/-
RP1-293L8.2	-0.04	0.612213	0.30	0.007451	2.55	0.010911	0.011044	0.9992	0/+
RNA5SP465	0.00	0.997481	0.33	0.002841	2.55	0.010924	0.01106	0.9992	0/+
HEXIM1	0.00	0.968049	-0.33	0.003064	-2.54	0.010943	0.011089	0.9992	0/-
LMTK3	-0.20	0.005345	0.14	0.210192	2.54	0.010952	0.011099	0.9992	-/0
ZNF823	-0.03	0.64107	0.30	0.006965	2.54	0.010979	0.011127	0.9992	0/+
LINC00462	-0.19	0.007512	0.15	0.186084	2.54	0.010998	0.011147	0.9992	-/0
RP11-603B24.2	-0.18	0.010094	0.16	0.166325	2.54	0.011043	0.011191	0.9992	-/0
RP11-227G15.1	0.04	0.568467	-0.30	0.008536	-2.54	0.011098	0.011224	0.9992	0/-
MLLT10P1	-0.04	0.603664	0.30	0.007838	2.54	0.01117	0.01132	0.9992	0/+
RP11-379P15.1	-0.17	0.01707	0.17	0.134289	2.54	0.011174	0.01132	0.9992	-/0
CRYGEP	-0.11	0.119354	0.23	0.043916	2.54	0.0112	0.011329	0.9992	0/+
RP11-15I20.1	0.08	0.279095	-0.26	0.021019	-2.53	0.011268	0.0114	0.9992	0/-
CIDEC	0.18	0.014589	-0.17	0.145706	-2.53	0.011351	0.011454	0.9992	+/0
OXTR	-0.18	0.012059	0.16	0.159118	2.53	0.011461	0.011576	0.9992	-/0
RNU4-22P	0.01	0.8905	-0.32	0.003944	-2.53	0.011466	0.011588	0.9992	0/-
RP11-137N23.1	-0.24	0.000907	0.10	0.3708	2.53	0.011496	0.011633	0.9992	-/0
RP11-62E14.2	0.18	0.012985	-0.16	0.155156	-2.53	0.011545	0.011684	0.9992	+/0

PRMT8	0.04	0.554138	-0.29	0.009385	-2.52	0.011638	0.011777	0.9992	0/-
RP11-497G19.7	0.01	0.893464	-0.32	0.004015	-2.52	0.011693	0.011838	0.9992	0/-
CENPO	0.29	4.78E-05	0.57	6.98E-08	2.52	0.011701	0.011845	0.9992	+/+
CASC10	-0.15	0.034119	0.19	0.099787	2.52	0.011702	0.011845	0.9992	-/0
SNORA38	-0.02	0.774115	0.31	0.005406	2.52	0.011731	0.011861	0.9992	0/+
C8orf89	-0.27	0.000174	0.07	0.543593	2.52	0.011751	0.011893	0.9992	-/0
CTC-428G20.2	0.02	0.784495	-0.31	0.005367	-2.51	0.011904	0.012017	0.9992	0/-
CCPG1	-0.06	0.402551	-0.38	0.00054	-2.51	0.011949	0.012069	0.9992	0/-
RP11-5C23.2	0.17	0.017379	-0.17	0.140721	-2.51	0.01197	0.012091	0.9992	+/0
DISC1FP1	-0.05	0.477293	0.28	0.011992	2.51	0.011982	0.012104	0.9992	0/+
TMEM220	0.05	0.492643	-0.28	0.011488	-2.51	0.011983	0.012104	0.9992	0/-
SSPN	-0.07	0.344088	-0.39	0.000424	-2.51	0.011985	0.012107	0.9992	0/-
ZNF558	-0.01	0.90347	0.32	0.004056	2.51	0.012011	0.012117	0.9992	0/+
S100A6	-0.02	0.81129	-0.35	0.001977	-2.51	0.01202	0.012117	0.9992	0/-
EEF1A1P9	-0.18	0.012935	0.16	0.160386	2.51	0.012026	0.012117	0.9992	-/0
GRID1	-0.28	9.51E-05	0.06	0.617175	2.51	0.012098	0.012187	0.9992	-/0
RP11-620J15.4	0.11	0.143349	-0.23	0.041322	-2.51	0.012142	0.012238	0.9992	0/-
RPL17P26	-0.21	0.003663	0.13	0.256887	2.51	0.012152	0.012251	0.9992	-/0
CPZ	-0.21	0.002928	0.12	0.276078	2.51	0.012169	0.012261	0.9992	-/0
IMPG2	-0.20	0.004265	0.13	0.246102	2.50	0.012277	0.012395	0.9992	-/0
RP11-327P2.5	-0.29	5.78E-05	0.05	0.676596	2.50	0.012281	0.012395	0.9992	-/0
AC138517.6	-0.02	0.83347	-0.34	0.00216	-2.50	0.012291	0.012398	0.9992	0/-
RP11-89N17.4	0.06	0.397277	-0.27	0.01561	-2.50	0.012294	0.012414	0.9992	0/-
LYPLA1	0.03	0.713095	0.35	0.001562	2.50	0.012299	0.012418	0.9992	0/+
RP11-390F4.6	-0.11	0.145767	0.23	0.041346	2.50	0.012311	0.012427	0.9992	0/+
OBP2A	0.05	0.520829	-0.29	0.011046	-2.50	0.012381	0.012488	0.9992	0/-
PP14571	-0.30	2.58E-05	0.03	0.769807	2.50	0.012427	0.01253	0.9992	-/0
SMPD4P1	-0.08	0.251509	0.25	0.02595	2.50	0.012446	0.012555	0.9992	0/+
DUSP13	0.03	0.692679	-0.30	0.007142	-2.50	0.012476	0.012584	0.9992	0/-
ARHGEF18	-0.06	0.40051	0.27	0.015767	2.50	0.012512	0.012626	0.9992	0/+
ADAM20P1	0.02	0.80292	0.34	0.002048	2.50	0.012538	0.012651	0.9992	0/+
PLIN1	0.17	0.018158	-0.17	0.143279	-2.50	0.012548	0.01267	0.9992	+/0
GAPDHP14	-0.07	0.352639	0.27	0.018414	2.50	0.012578	0.012702	0.9992	0/+
MIR621	0.13	0.081405	0.44	6.75E-05	2.49	0.012599	0.012715	0.9992	0/+
AC073063.10	-0.09	0.199801	0.24	0.03257	2.49	0.012613	0.012722	0.9992	0/+
RP11-582E3.6	0.11	0.122921	-0.22	0.048251	-2.49	0.012614	0.012722	0.9992	0/-
CXADR	0.18	0.012782	-0.16	0.167644	-2.49	0.012656	0.012786	0.9992	+/0
RP11-463O9.9	-0.10	0.147622	0.23	0.042216	2.49	0.012697	0.012824	0.9992	0/+
KIAA0319L	0.02	0.816504	-0.31	0.005443	-2.49	0.01281	0.012978	0.9992	0/-
CDKN1C	-0.18	0.012923	0.16	0.168687	2.49	0.012834	0.013019	0.9992	-/0
SOX5	-0.18	0.0127	0.16	0.17038	2.49	0.012881	0.013077	0.9992	-/0
GPT2	0.05	0.493028	0.37	0.000845	2.49	0.012884	0.013077	0.9992	0/+
RP11-302B13.5	0.05	0.472865	-0.28	0.013209	-2.49	0.012884	0.013077	0.9992	0/-
RP11-527D7.1	0.10	0.161988	-0.23	0.039895	-2.49	0.012926	0.013106	0.9992	0/-
RP1-122P22.2	-0.21	0.002824	0.12	0.29166	2.48	0.012999	0.013183	0.9992	-/0
CTC-431G16.2	-0.10	0.159477	0.23	0.040674	2.48	0.013012	0.013205	0.9992	0/+
AC010127.3	0.00	0.957732	0.32	0.003952	2.48	0.013041	0.013224	0.9992	0/+

ADIPOQ	0.17	0.02136	-0.17	0.137524	-2.48	0.013066	0.013243	0.9992	+/0
GFAP	0.03	0.679392	-0.30	0.007853	-2.48	0.01312	0.013282	0.9992	0/-
HLA-DRB5	0.16	0.022764	-0.17	0.134015	-2.48	0.013123	0.013282	0.9992	+/0
ZNF415P1	0.10	0.182827	-0.24	0.036641	-2.48	0.013125	0.013282	0.9992	0/-
SDSL	0.08	0.249525	-0.25	0.02769	-2.48	0.013135	0.013304	0.9992	0/-
RP11-403F21.4	-0.19	0.009535	0.15	0.193872	2.48	0.013147	0.013317	0.9992	-/0
RP11-530N7.3	0.02	0.741304	-0.30	0.006765	-2.48	0.01316	0.013333	0.9992	0/-
MDP1	0.06	0.444644	0.37	0.000734	2.48	0.01317	0.013352	0.9992	0/+
CCDC27	-0.28	6.07E-05	0.05	0.693682	2.48	0.013211	0.013388	0.9992	-/0
ERV3-1	-0.24	0.000741	0.09	0.423888	2.48	0.013256	0.013439	0.9992	-/0
TMEM181	0.10	0.16017	-0.23	0.041533	-2.47	0.013331	0.013516	0.9992	0/-
KEAP1	-0.05	0.511889	0.28	0.012354	2.47	0.013342	0.013528	0.9992	0/+
ZNF627	-0.04	0.543947	0.29	0.011387	2.47	0.013391	0.013564	0.9992	0/+
RIN1	-0.12	0.105062	-0.43	0.000102	-2.47	0.013406	0.013602	0.9992	0/-
NINJ1	0.10	0.173662	-0.23	0.039118	-2.47	0.013415	0.013608	0.9992	0/-
ZNF136	-0.10	0.179886	0.24	0.038151	2.47	0.013472	0.013669	0.9992	0/+
SAC3D1	0.33	3.71E-06	0.00	0.988199	-2.47	0.013556	0.013752	0.9992	+/0
TMEM176A	0.11	0.136868	-0.22	0.047943	-2.47	0.013607	0.013807	0.9992	0/-
CA12	0.06	0.431144	-0.27	0.015942	-2.47	0.013684	0.0139	0.9992	0/-
RP3-521E19.3	0.27	0.000114	-0.05	0.636432	-2.46	0.013799	0.014034	0.9992	+/0
RP11-706O15.7	0.11	0.14151	-0.23	0.047387	-2.46	0.013799	0.014034	0.9992	0/-
AF186192.1	-0.09	0.196011	0.24	0.036385	2.46	0.013817	0.014041	0.9992	0/+
PPIAP30	0.18	0.011313	-0.15	0.188422	-2.46	0.013832	0.014053	0.9992	+/0
MAN2B1	-0.15	0.038307	0.18	0.1084	2.46	0.01384	0.014066	0.9992	-/0
RPL12P35	-0.15	0.034116	0.18	0.115226	2.46	0.013863	0.014095	0.9992	-/0
DOCK6	-0.16	0.024246	0.17	0.136519	2.46	0.013919	0.014185	0.9992	-/0
SLC15A4	0.09	0.211574	-0.24	0.034401	-2.46	0.013965	0.014226	0.9992	0/-
CTD-2256P15.3	0.17	0.016226	-0.16	0.164534	-2.46	0.014054	0.01429	0.9992	+/0
RP11-473C19.1	0.08	0.289385	-0.25	0.025812	-2.45	0.014104	0.014329	0.9992	0/-
RPL7L1P12	0.11	0.1363	-0.22	0.049948	-2.45	0.01414	0.014361	0.9992	0/-
CTD-2619J13.17	-0.06	0.419614	0.27	0.017129	2.45	0.014152	0.014374	0.9992	0/+
RP11-53B5.1	0.09	0.192998	0.40	0.00024	2.45	0.014154	0.014374	0.9992	0/+
ZFP69B	0.00	0.957906	0.32	0.004413	2.45	0.014198	0.014412	0.9992	0/+
ZNF557	-0.02	0.821197	0.31	0.006139	2.45	0.014214	0.014415	0.9992	0/+
RP11-3P17.4	0.05	0.456265	-0.27	0.015523	-2.45	0.014232	0.014441	0.9992	0/-
FAM32A	-0.02	0.813172	0.31	0.006318	2.45	0.014321	0.01454	0.9992	0/+
Y_RNA.293	0.04	0.615272	0.35	0.001459	2.45	0.014325	0.01454	0.9992	0/+
ZCRB1	0.00	0.955869	-0.32	0.004497	-2.45	0.014351	0.014582	0.9992	0/-
ZNF317	-0.04	0.619219	0.29	0.010206	2.45	0.014377	0.01461	0.9992	0/+
ALG10B	-0.01	0.88828	-0.33	0.003094	-2.45	0.014446	0.014706	0.9992	0/-
CIDEA	0.15	0.035428	-0.18	0.117137	-2.45	0.01446	0.01471	0.9992	+/0
RP11-322D14.1	0.30	1.75E-05	-0.02	0.863638	-2.44	0.01449	0.014751	0.9992	+/0
RAVER1	-0.06	0.425434	0.27	0.017379	2.44	0.014548	0.014809	0.9992	0/+
HSD17B13	0.26	0.00024	-0.07	0.569681	-2.44	0.014572	0.014838	0.9992	+/0
TP53BP2	-0.21	0.003476	0.12	0.295237	2.44	0.014576	0.014838	0.9992	-/0
KCNB2	0.03	0.680534	0.35	0.001825	2.44	0.014677	0.014921	0.9992	0/+
PRDM2	-0.22	0.001986	0.11	0.349896	2.44	0.01471	0.014956	0.9992	-/0

RDH5	0.07	0.335969	-0.26	0.023251	-2.44	0.014794	0.015033	0.9992	0/-
RP11-466C23.5	0.16	0.026708	-0.17	0.137287	-2.44	0.014816	0.015052	0.9992	+/0
SLFN13	0.47	5.87E-12	0.18	0.124412	-2.43	0.014981	0.015231	0.9992	+/0
CBLN1	0.00	0.961981	0.32	0.004689	2.43	0.014986	0.015231	0.9992	0/+
RP3-325F22.3	0.07	0.320253	-0.25	0.024871	-2.43	0.015013	0.015251	0.9992	0/-
AL356475.1	0.08	0.245009	-0.24	0.032469	-2.43	0.015018	0.015267	0.9992	0/-
C1R	-0.05	0.530647	-0.36	0.001206	-2.43	0.015075	0.015308	0.9992	0/-
BIRC3	0.04	0.553203	-0.28	0.012788	-2.43	0.015078	0.015311	0.9992	0/-
ZNF431	0.00	0.956685	0.32	0.00479	2.43	0.015086	0.015315	0.9992	0/+
ZNF253	-0.05	0.499024	0.27	0.014827	2.43	0.015141	0.015398	0.9992	0/+
CTC-543D15.8	-0.19	0.008564	0.14	0.224128	2.43	0.015159	0.01542	0.9992	-/0
C1QA	0.17	0.016114	-0.15	0.175955	-2.43	0.015262	0.015507	0.9992	+/0
VIPR1	0.15	0.037094	-0.18	0.11987	-2.43	0.015275	0.015516	0.9992	+/0
RP11-20J15.3	0.04	0.561063	-0.28	0.01274	-2.43	0.01529	0.015536	0.9992	0/-
RP11-184I16.3	0.15	0.04014	-0.18	0.115311	-2.43	0.015308	0.015555	0.9992	+/0
HNRNPA3P9	0.12	0.090151	0.42	0.000106	2.42	0.015328	0.015571	0.9992	0/+
CRAT37	0.02	0.733872	-0.30	0.008377	-2.42	0.015406	0.015651	0.9992	0/-
C12orf65	0.20	0.005666	-0.13	0.262693	-2.42	0.015466	0.015705	0.9992	+/0
TECTA	-0.05	0.487372	0.27	0.015683	2.42	0.015466	0.015705	0.9992	0/+
ZC3HAV1	0.21	0.002945	-0.11	0.322827	-2.42	0.015471	0.015718	0.9992	+/0
AC003003.5	0.07	0.305139	-0.25	0.027078	-2.42	0.015498	0.015753	0.9992	0/-
RP11-74M13.4	-0.21	0.003852	0.12	0.297973	2.42	0.0155	0.015753	0.9992	-/0
RP11-213G2.1	0.10	0.16153	-0.22	0.047992	-2.42	0.015502	0.015753	0.9992	0/-
ANKS1A	-0.21	0.002737	0.11	0.332086	2.42	0.015635	0.015891	0.9992	-/0
TMX3	0.06	0.398727	-0.26	0.020461	-2.42	0.015687	0.015955	0.9992	0/-
RIC3	-0.28	0.000108	0.05	0.682301	2.42	0.015688	0.015955	0.9992	-/0
ODF3L1	-0.02	0.807232	-0.33	0.002831	-2.41	0.015748	0.016025	0.9992	0/-
ILF3	0.15	0.03918	0.45	4.39E-05	2.41	0.015778	0.016054	0.9992	+/+
AQP7P1	0.19	0.006725	-0.13	0.251441	-2.41	0.015787	0.016073	0.9992	+/0
NANOGP5	0.17	0.018756	-0.16	0.169553	-2.41	0.015804	0.016089	0.9992	+/0
RPL29P2	-0.05	0.523905	0.28	0.014593	2.41	0.015805	0.016093	0.9992	0/+
ZNF700	0.12	0.109842	0.42	0.000142	2.41	0.015842	0.016121	0.9992	0/+
RP11-478C6.1	-0.27	0.000136	0.05	0.661692	2.41	0.015989	0.016307	0.9992	-/0
RP11-793H13.1	0.16	0.02832	-0.17	0.142158	-2.41	0.01599	0.016307	0.9992	+/0
LINC01597	-0.16	0.027875	0.17	0.143245	2.41	0.015993	0.016307	0.9992	-/0
FOXL2NB	-0.18	0.010464	0.14	0.216746	2.41	0.016028	0.016352	0.9992	-/0
AC010884.1	-0.20	0.004861	0.12	0.2835	2.41	0.016057	0.016381	0.9992	-/0
RP11-96L14.7	-0.17	0.019424	0.16	0.169125	2.41	0.016058	0.016384	0.9992	-/0
NDUFV2	0.26	0.000325	-0.07	0.564333	-2.41	0.016082	0.01641	0.9992	+/0
C2orf91	-0.01	0.844552	-0.33	0.003208	-2.41	0.016093	0.016422	0.9992	0/-
DSTYK	-0.26	0.000313	0.07	0.569186	2.41	0.016102	0.016426	0.9992	-/0
Y_RNA.73	0.18	0.011973	-0.14	0.206957	-2.41	0.016142	0.016461	0.9992	+/0
SQRDL	0.06	0.373769	-0.26	0.022775	-2.41	0.016151	0.01647	0.9992	0/-
RP11-265B8.5	0.18	0.011741	-0.14	0.209274	-2.40	0.01622	0.016531	0.9992	+/0
RP3-399L15.3	-0.02	0.774451	0.30	0.008153	2.40	0.016295	0.016611	0.9992	0/+
ATP5G1P4	0.33	2.34E-06	0.02	0.876423	-2.40	0.016322	0.01663	0.9992	+/0
NSMAF	-0.07	0.306927	0.25	0.028514	2.40	0.016343	0.01665	0.9992	0/+

AC114271.2	-0.02	0.78539	0.30	0.00799	2.40	0.01637	0.016678	0.9992	0/+
TMEM30B	0.06	0.41497	-0.26	0.020486	-2.40	0.016374	0.016682	0.9992	0/-
CHERP	-0.03	0.645406	0.29	0.011203	2.40	0.016389	0.016685	0.9992	0/+
KCNN4	0.02	0.73504	-0.29	0.009114	-2.40	0.016527	0.016806	0.9992	0/-
RP11-142O6.1	0.17	0.017946	-0.15	0.179277	-2.40	0.016568	0.016838	0.9992	+/0
FAM69B	-0.15	0.037712	0.17	0.127733	2.39	0.016631	0.016902	0.9992	-/0
RNU2-7P	0.01	0.866194	0.33	0.003542	2.39	0.016631	0.016902	0.9992	0/+
AC087239.1	0.17	0.017354	-0.15	0.182848	-2.39	0.016689	0.01697	0.9992	+/0
TRBV7-8	0.15	0.03244	-0.17	0.138109	-2.39	0.016704	0.016992	0.9992	+/0
RAB3A	-0.28	9.66E-05	0.04	0.715488	2.39	0.016715	0.017011	0.9992	-/0
ZNF14	-0.08	0.259702	0.24	0.034449	2.39	0.016726	0.017037	0.9992	0/+
RP11-430K21.2	0.01	0.840798	-0.30	0.00723	-2.39	0.016774	0.017072	0.9992	0/-
PRR4	0.09	0.224787	-0.23	0.039467	-2.39	0.016786	0.017088	0.9992	0/-
ASH2L	-0.09	0.199882	0.23	0.044076	2.39	0.016928	0.017255	0.9992	0/+
AC002398.11	-0.01	0.891616	0.31	0.006515	2.39	0.016979	0.017303	0.9992	0/+
IL6ST	-0.19	0.006847	-0.48	9.05E-06	-2.39	0.017018	0.017335	0.9992	-/-
AC092066.1	0.25	0.000487	-0.07	0.536429	-2.38	0.017084	0.017392	0.9992	+/0
AC006369.2	0.04	0.617206	-0.28	0.012636	-2.38	0.017096	0.017411	0.9992	0/-
CHRNA3	0.21	0.002983	-0.11	0.34324	-2.38	0.017105	0.017431	0.9992	+/0
RP11-383F6.1	0.35	6.42E-07	0.04	0.730596	-2.38	0.017173	0.017533	0.9992	+/0
RP5-1027O11.1	0.00	0.948744	0.32	0.004536	2.38	0.017198	0.017562	0.9992	0/+
RPS2P55	-0.22	0.002041	0.10	0.38333	2.38	0.017229	0.017604	0.9992	-/0
SNCB	-0.06	0.411818	0.26	0.021966	2.38	0.017278	0.017636	0.9992	0/+
NAGS	-0.04	0.55533	-0.35	0.001594	-2.38	0.017304	0.017661	0.9992	0/-
LAYN	-0.31	1.08E-05	0.00	0.981431	2.38	0.017442	0.017808	0.9992	-/0
RP11-227G15.8	0.06	0.410483	-0.26	0.022289	-2.38	0.017443	0.017808	0.9992	0/-
SNORA26.4	0.21	0.003471	-0.11	0.332964	-2.38	0.01748	0.017834	0.9992	+/0
GIPC1	-0.17	0.021584	0.16	0.172864	2.38	0.017492	0.017844	0.9992	-/0
COX20	-0.09	0.209208	0.23	0.044153	2.37	0.017609	0.018004	0.9992	0/+
DRD2	0.18	0.010124	-0.14	0.235339	-2.37	0.017632	0.018026	0.9992	+/0
RNF170	-0.28	9.00E-05	0.04	0.74162	2.37	0.01765	0.018036	0.9992	-/0
CCNB1IP1	-0.16	0.022604	0.16	0.171071	2.37	0.017708	0.018081	0.9992	-/0
ADIRF-AS1	-0.25	0.000539	0.07	0.535667	2.37	0.017731	0.018097	0.9992	-/0
AC006538.1	-0.06	0.371095	0.25	0.02554	2.37	0.017772	0.018132	0.9992	0/+
TGS1	-0.02	0.778554	0.29	0.009114	2.37	0.01795	0.018343	0.9992	0/+
NRARP	0.13	0.06183	0.43	9.08E-05	2.37	0.01801	0.018426	0.9992	0/+
RP5-836J3.1	0.15	0.03979	-0.17	0.13324	-2.36	0.01805	0.018468	0.9992	+/0
AC092614.2	0.00	0.963387	0.31	0.005969	2.36	0.018073	0.018494	0.9992	0/+
CTC-529I10.1	0.08	0.253543	-0.24	0.03824	-2.36	0.018074	0.018494	0.9992	0/-
RARA	-0.06	0.424604	-0.36	0.001092	-2.36	0.018077	0.018494	0.9992	0/-
CTC-543D15.3	-0.02	0.76777	0.29	0.009482	2.36	0.018155	0.01859	0.9992	0/+
RP11-1134I14.8	0.07	0.360781	-0.25	0.027004	-2.36	0.018172	0.018612	0.9992	0/-
CTC-499B15.5	0.03	0.727235	0.33	0.002818	2.36	0.018265	0.018737	0.9992	0/+
RP11-770J1.5	0.01	0.846558	0.32	0.003847	2.36	0.018324	0.01882	0.9992	0/+
ZNF430	-0.06	0.399822	0.25	0.024403	2.36	0.018398	0.018884	0.9992	0/+
LAMC3	-0.15	0.031829	0.16	0.150986	2.36	0.018402	0.018894	0.9992	-/0
KLHDC7A	0.02	0.798351	-0.29	0.008988	-2.36	0.018417	0.01891	0.9992	0/-

RB1	-0.38	5.46E-08	-0.08	0.500103	2.36	0.018472	0.018974	0.9992	-/0
CTD-2240E14.4	0.08	0.260372	0.38	0.000551	2.36	0.018493	0.019002	0.9992	0/+
RP11-351I24.1	-0.08	0.267092	0.24	0.037353	2.36	0.018508	0.019019	0.9992	0/+
GNAI2	0.07	0.302156	-0.24	0.033382	-2.35	0.018631	0.019172	0.9992	0/-
C1orf106	-0.27	0.000195	0.05	0.669765	2.35	0.018697	0.019246	0.9992	-/0
PGAM1P4	-0.16	0.026706	0.16	0.167288	2.35	0.018892	0.019441	0.9992	-/0
TIMM44	0.03	0.658884	0.34	0.002465	2.35	0.018957	0.019486	0.9992	0/+
MRPL15	-0.04	0.546361	0.27	0.017074	2.35	0.018975	0.019508	0.9992	0/+
H2BFWT	-0.01	0.924628	0.30	0.006963	2.35	0.018978	0.019511	0.9992	0/+
SLC35E1	-0.19	0.007999	0.13	0.270221	2.35	0.019022	0.019569	0.9992	-/0
SPG20-AS1	-0.15	0.040319	0.17	0.138762	2.34	0.019089	0.019614	0.9992	-/0
HEPACAM	0.07	0.3296	-0.24	0.03154	-2.34	0.019179	0.01971	0.9992	0/-
HLA-DOB	0.07	0.315716	-0.24	0.033087	-2.34	0.019247	0.019803	0.9992	0/-
ZNF763	-0.09	0.23125	0.23	0.044772	2.34	0.019411	0.019908	0.9992	0/+
C9orf50	0.02	0.765614	-0.29	0.010367	-2.34	0.019421	0.019918	0.9992	0/-
RP11-983P16.4	0.08	0.25467	-0.23	0.041281	-2.34	0.01951	0.019988	0.9992	0/-
CTD-3193O13.1	0.10	0.153178	0.40	0.0003	2.34	0.019516	0.019992	0.9992	0/+
HIST2H2BA	-0.16	0.029611	0.16	0.163881	2.34	0.019519	0.019992	0.9992	-/0
AC093724.2	-0.01	0.906176	0.30	0.007577	2.33	0.019599	0.020062	0.9992	0/+
SLFN11	0.34	9.69E-07	0.04	0.725911	-2.33	0.019616	0.020065	0.9992	+/0
RP11-696N14.1	-0.23	0.00107	0.08	0.487202	2.33	0.019651	0.020101	0.9992	-/0
HS6ST2	-0.06	0.429533	0.26	0.0242	2.33	0.019667	0.020123	0.9992	0/+
ZCCHC18	-0.02	0.752486	0.29	0.011036	2.33	0.019926	0.020353	0.9992	0/+
ZNF562	-0.04	0.62277	0.27	0.015019	2.33	0.019958	0.020385	0.9992	0/+
BORA	0.24	0.000827	0.51	2.01E-06	2.33	0.020004	0.02044	0.9992	+/+
RUNDC1	-0.06	0.434419	-0.36	0.001328	-2.32	0.020106	0.020558	0.9992	0/-
SYT12	0.06	0.436936	-0.25	0.024317	-2.32	0.020107	0.020558	0.9992	0/-
GCDH	0.00	0.973095	0.30	0.006714	2.32	0.020121	0.020571	0.9992	0/+
ZADH2	-0.02	0.802831	-0.32	0.003926	-2.32	0.020148	0.020622	0.9992	0/-
DDA1	-0.06	0.406086	0.25	0.026626	2.32	0.0202	0.02067	0.9992	0/+
HSPA6	0.24	0.000669	-0.07	0.550183	-2.32	0.02028	0.02077	0.9992	+/0
DDX18P5	0.18	0.010154	-0.13	0.261447	-2.32	0.020412	0.020907	0.9992	+/0
RP11-467L19.1	-0.07	0.334222	0.24	0.033281	2.32	0.020413	0.020907	0.9992	0/+
WIZ	-0.03	0.680204	0.28	0.013472	2.32	0.020426	0.02092	0.9992	0/+
CTD-2649C14.3	-0.22	0.002142	0.09	0.42131	2.32	0.020512	0.021051	0.9992	-/0
RP11-307C18.1	-0.04	0.550698	-0.34	0.002019	-2.32	0.020562	0.021103	0.9992	0/-
ZMYND19P1	0.11	0.131777	0.40	0.000274	2.31	0.020648	0.021202	0.9992	0/+
CENPBD1	-0.26	0.00021	0.05	0.695284	2.31	0.020783	0.021314	0.9992	-/0
SLC20A2	-0.24	0.000824	0.07	0.534502	2.31	0.020879	0.021403	0.9992	-/0
IQSEC1	-0.30	1.73E-05	0.00	0.991674	2.31	0.020888	0.021413	0.9992	-/0
AC006946.15	0.18	0.010633	-0.13	0.261902	-2.31	0.020933	0.021448	0.9992	+/0
BEND3	0.10	0.185292	0.39	0.000429	2.31	0.020969	0.021483	0.9992	0/+
BDKRB2	0.16	0.03083	-0.16	0.170596	-2.31	0.020979	0.021499	0.9992	+/0
NINL	-0.02	0.769324	0.29	0.011346	2.31	0.02102	0.021541	0.9992	0/+
SLC16A9	0.17	0.018698	-0.14	0.212455	-2.31	0.021088	0.021608	0.9992	+/0
CRIP2	-0.04	0.613044	-0.34	0.002527	-2.31	0.02113	0.02165	0.9992	0/-
BNIP3P30	-0.02	0.795419	0.29	0.010782	2.30	0.02117	0.021695	0.9992	0/+

C11orf80	0.00	0.978787	-0.30	0.007127	-2.30	0.021272	0.021826	0.9992	0/-
LL22NC03-2H8.	0.15	0.035188	-0.16	0.163279	-2.30	0.021435	0.02198	0.9992	+/0
CTC-459M5.1	0.03	0.671462	-0.28	0.01459	-2.30	0.021441	0.021986	0.9992	0/-
AC018878.3	0.17	0.017501	-0.14	0.221305	-2.30	0.021484	0.022021	0.9992	+/0
ACSL6	0.16	0.022092	-0.15	0.20151	-2.30	0.021524	0.02205	0.9992	+/0
TRBV11-1	0.18	0.013757	-0.13	0.243539	-2.30	0.021562	0.022079	0.9992	+/0
SCIN	-0.06	0.439012	-0.35	0.001501	-2.30	0.021589	0.022137	0.9992	0/-
CCDC127	0.02	0.764529	-0.28	0.011896	-2.30	0.021641	0.022188	0.9992	0/-
RP11-384P7.7	-0.18	0.011846	0.13	0.258417	2.30	0.021681	0.02222	0.9992	-/0
PIK3R2	0.01	0.905116	0.31	0.005617	2.29	0.021817	0.022351	0.9992	0/+
LINC01301	-0.18	0.012401	0.13	0.255817	2.29	0.021877	0.022399	0.9992	-/0
PPP1R2P6	0.21	0.003377	-0.10	0.389853	-2.29	0.022055	0.022546	0.9992	+/0
KDM5B	-0.33	3.07E-06	-0.03	0.799645	2.29	0.0221	0.022597	0.9992	-/0
HNF1B	-0.04	0.587479	-0.34	0.002519	-2.29	0.022199	0.022671	0.9992	0/-
6-Mar	0.17	0.019807	-0.14	0.216021	-2.29	0.022213	0.022681	0.9992	+/0
TP63	-0.18	0.012469	0.13	0.258495	2.29	0.022251	0.022726	0.9992	-/0
RP11-493L12.5	0.15	0.033103	-0.16	0.173864	-2.28	0.022352	0.02286	0.9992	+/0
TNFRSF1A	-0.09	0.216149	-0.38	0.00058	-2.28	0.022355	0.022863	0.9992	0/-
RPSAP69	0.24	0.000802	-0.07	0.558329	-2.28	0.022384	0.022889	0.9992	+/0
MT1M	0.23	0.001385	-0.08	0.49471	-2.28	0.022421	0.022921	0.9992	+/0
RP3-393E18.2	0.20	0.00426	-0.10	0.368786	-2.28	0.022422	0.022924	0.9992	+/0
ZNF266	0.04	0.575711	0.34	0.002484	2.28	0.022517	0.02303	0.9992	0/+
SLC25A42	-0.30	1.70E-05	0.00	0.978189	2.28	0.022575	0.023078	0.9992	-/0
AC078883.3	0.16	0.025944	-0.15	0.195662	-2.28	0.022616	0.023142	0.9992	+/0
RP11-30K9.1	0.00	0.976545	0.30	0.007001	2.28	0.022694	0.023215	0.9992	0/+
KNTC1	0.59	0	0.35	0.001773	-2.28	0.022765	0.023286	0.9992	+/+
OR7E22P	0.15	0.03631	-0.16	0.169136	-2.28	0.022776	0.023292	0.9992	+/0
AC005625.1	0.09	0.214838	0.38	0.000593	2.28	0.022786	0.023295	0.9992	0/+
RP4-765C7.2	-0.05	0.510656	0.26	0.023263	2.28	0.022883	0.023404	0.9992	0/+
CALR4P	0.08	0.288228	-0.23	0.043581	-2.28	0.022888	0.023407	0.9992	0/-
RP11-108M9.3	0.14	0.051533	0.42	0.000114	2.27	0.022983	0.0235	0.9992	0/+
EML5	-0.21	0.003368	0.10	0.400638	2.27	0.022997	0.023523	0.9992	-/0
SGMS2	0.01	0.912193	-0.29	0.009199	-2.27	0.023021	0.023539	0.9992	0/-
LINC01451	0.22	0.002067	-0.09	0.456319	-2.27	0.023061	0.023567	0.9992	+/0
RP11-262H14.3	0.06	0.409013	-0.24	0.030702	-2.27	0.023097	0.023603	0.9992	0/-
FAM225A	0.17	0.01655	-0.13	0.239473	-2.27	0.023164	0.023663	0.9992	+/0
RP1-91J24.1	0.19	0.007019	-0.11	0.324379	-2.27	0.023239	0.023744	0.9992	+/0
RP11-531A24.3	0.06	0.388605	0.36	0.001395	2.27	0.023264	0.023763	0.9992	0/+
HMGB3P22	0.14	0.047032	-0.16	0.151878	-2.27	0.023268	0.023769	0.9992	+/0
CHCHD7	-0.05	0.459302	0.25	0.027072	2.27	0.023282	0.023788	0.9992	0/+
TIGD7	-0.16	0.022589	0.14	0.213758	2.27	0.023493	0.023984	0.9992	-/0
HIST1H2BG	-0.07	0.348295	0.24	0.037218	2.27	0.023505	0.024003	0.9992	0/+
RP11-442O1.3	-0.18	0.012912	0.13	0.265781	2.26	0.023519	0.024009	0.9992	-/0
TNS2	-0.04	0.619982	-0.33	0.00301	-2.26	0.02354	0.024035	0.9992	0/-
RP11-554I8.2	0.14	0.044948	-0.16	0.156953	-2.26	0.023554	0.024064	0.9992	+/0
AC011816.1	0.14	0.045383	-0.16	0.156637	-2.26	0.023631	0.02416	0.9992	+/0
POLA1	0.34	1.06E-06	0.05	0.671319	-2.26	0.023704	0.024237	0.9992	+/0

OR5BT1P	0.00	0.991592	-0.30	0.008042	-2.26	0.023847	0.024393	0.9992	0/-
VWA8-AS1	-0.29	4.44E-05	0.01	0.932102	2.26	0.023999	0.024563	0.9992	-/0
CTD-2666L21.1	0.02	0.769044	0.32	0.004604	2.26	0.024044	0.024601	0.9992	0/+
LRP11	0.20	0.005223	-0.10	0.363649	-2.26	0.024053	0.024605	0.9992	+/0
SYNJ2	0.02	0.760324	-0.28	0.013703	-2.26	0.024054	0.024605	0.9992	0/-
DPY19L1P1	0.20	0.005327	-0.10	0.361638	-2.26	0.024065	0.024627	0.9992	+/0
RP11-568G11.4	0.19	0.009065	-0.12	0.306533	-2.25	0.024164	0.024755	0.9992	+/0
KCNK3	0.06	0.372657	-0.24	0.035831	-2.25	0.024216	0.0248	0.9992	0/-
AC062029.1	-0.29	4.98E-05	0.01	0.922289	2.25	0.024253	0.024861	0.9992	-/0
RP11-180P8.5	0.18	0.013786	-0.13	0.265448	-2.25	0.024257	0.024864	0.9992	+/0
ZNF440	0.04	0.549432	0.34	0.002561	2.25	0.024275	0.02488	0.9992	0/+
TMEM155	-0.01	0.923589	0.29	0.009613	2.25	0.024295	0.024896	0.9992	0/+
RP11-667K14.3	-0.04	0.568177	-0.34	0.002717	-2.25	0.024323	0.024922	0.9992	0/-
FCGR1B	0.14	0.049675	-0.16	0.15361	-2.25	0.024357	0.024979	0.9992	+/0
SHISA5	0.08	0.279232	-0.22	0.047979	-2.25	0.024367	0.024986	0.9992	0/-
RP11-214K3.18	-0.01	0.925987	0.29	0.009651	2.25	0.024472	0.025075	0.9992	0/+
RP1-313L4.3	-0.28	9.65E-05	0.02	0.845908	2.25	0.02448	0.025091	0.9992	-/0
ZNF724P	0.19	0.006669	0.47	1.79E-05	2.25	0.024572	0.025168	0.9992	+/+
RP11-712L6.5	0.01	0.860356	-0.29	0.011264	-2.25	0.024609	0.025194	0.9992	0/-
TESC-AS1	0.19	0.007529	-0.11	0.330163	-2.25	0.024641	0.025222	0.9992	+/0
MT2A	0.17	0.016104	-0.13	0.253476	-2.25	0.024644	0.025222	0.9992	+/0
PSTK	-0.05	0.465543	0.25	0.028453	2.25	0.024665	0.025235	0.9992	0/+
ZNF101P2	0.15	0.035021	-0.15	0.183847	-2.25	0.024706	0.025293	0.9992	+/0
AC007796.1	-0.18	0.011935	0.12	0.283678	2.25	0.024748	0.02536	0.9992	-/0
BHLHE41	-0.06	0.387077	-0.35	0.001526	-2.24	0.024774	0.025389	0.9992	0/-
AF186192.5	-0.04	0.60585	0.26	0.020289	2.24	0.024798	0.025421	0.9992	0/+
AAED1	0.23	0.001467	-0.07	0.517402	-2.24	0.024824	0.02545	0.9992	+/0
LMBRD2	0.05	0.483755	-0.25	0.027438	-2.24	0.024887	0.025523	0.9992	0/-
ZFYVE28	-0.29	4.59E-05	0.01	0.941405	2.24	0.024892	0.025527	0.9992	-/0
CTD-2313N18.7	-0.01	0.920042	0.29	0.010001	2.24	0.024895	0.025527	0.9992	0/+
OPN1LW	0.17	0.018978	-0.13	0.239804	-2.24	0.024901	0.02553	0.9992	+/0
AC005306.3	0.03	0.704491	0.32	0.004102	2.24	0.024915	0.025539	0.9992	0/+
CTD-3022L24.1	0.06	0.388981	-0.24	0.035327	-2.24	0.02492	0.025539	0.9992	0/-
RP11-359M6.2	0.16	0.025359	-0.14	0.213462	-2.24	0.024946	0.025571	0.9992	+/0
LINC01585	0.15	0.039797	-0.16	0.174632	-2.24	0.024948	0.025571	0.9992	+/0
SHC1P2	-0.08	0.288278	0.22	0.047821	2.24	0.024976	0.025603	0.9992	0/+
AC093107.7	-0.02	0.826702	0.28	0.012375	2.24	0.024976	0.025607	0.9992	0/+
AFMID	-0.18	0.014065	0.13	0.27063	2.24	0.025155	0.025779	0.9992	-/0
MTND4P9	0.07	0.349886	-0.23	0.040006	-2.24	0.025217	0.025847	0.9992	0/-
BCDIN3D-AS1	-0.11	0.12318	-0.39	0.000354	-2.24	0.025272	0.025908	0.9992	0/-
CD27-AS1	0.03	0.643233	-0.27	0.019039	-2.24	0.025292	0.025936	0.9992	0/-
MVB12A	-0.20	0.005265	0.10	0.375401	2.24	0.025335	0.025972	0.9992	-/0
CATIP	-0.05	0.481787	-0.34	0.002205	-2.24	0.025337	0.025978	0.9992	0/-
BCL2L14	0.06	0.392141	-0.24	0.035713	-2.24	0.025364	0.026004	0.9992	0/-
RNU6-1053P	-0.03	0.666097	0.27	0.018168	2.24	0.025414	0.026048	0.9992	0/+
LCMT1-AS1	0.01	0.843098	0.31	0.005963	2.23	0.02543	0.026052	0.9992	0/+
CBWD5	0.25	0.00037	-0.05	0.694279	-2.23	0.025474	0.026125	0.9992	+/0

RP11-563J2.2	0.20	0.005887	-0.10	0.364574	-2.23	0.025488	0.026128	0.9992	+/0
ZNF843	-0.37	9.14E-08	-0.09	0.447961	2.23	0.025521	0.026173	0.9992	-/0
DENND2C	-0.04	0.568084	0.26	0.022965	2.23	0.025523	0.026173	0.9992	0/+
CMAS	0.06	0.378499	-0.24	0.037366	-2.23	0.02553	0.026186	0.9992	0/-
TBC1D3P2	0.03	0.656784	0.32	0.003743	2.23	0.02554	0.026199	0.9992	0/+
RP11-423C15.3	0.08	0.267332	0.37	0.000955	2.23	0.02562	0.026279	0.9992	0/+
ASH1L-IT1	0.16	0.02852	-0.14	0.207482	-2.23	0.025637	0.026295	0.9992	+/0
S100A11	0.03	0.659387	-0.27	0.018693	-2.23	0.02569	0.026337	0.9992	0/-
RP11-337N6.2	0.03	0.697879	-0.27	0.017153	-2.23	0.025728	0.026375	0.9992	0/-
AEBP2	-0.01	0.939433	-0.30	0.007608	-2.23	0.025763	0.02642	0.9992	0/-
RP11-667K14.9	0.20	0.004472	-0.10	0.398169	-2.23	0.025794	0.026452	0.9992	+/0
MRPL4	0.00	0.991073	0.30	0.008576	2.23	0.025798	0.026458	0.9992	0/+
PTGR1	0.16	0.026816	-0.14	0.214297	-2.23	0.025833	0.02649	0.9992	+/0
SNORD3B-1	0.07	0.343389	0.36	0.00137	2.23	0.025863	0.026509	0.9992	0/+
COL13A1	0.19	0.006813	-0.11	0.352152	-2.23	0.025869	0.026513	0.9992	+/0
VSTM1	-0.04	0.576433	-0.33	0.003045	-2.23	0.025873	0.026513	0.9992	0/-
FCRLB	-0.36	3.70E-07	-0.07	0.550614	2.23	0.025895	0.026538	0.9992	-/0
RP11-33A14.3	0.03	0.707349	-0.27	0.016929	-2.23	0.025903	0.026541	0.9992	0/-
INTS8	-0.03	0.68017	0.27	0.018028	2.23	0.025929	0.026567	0.9992	0/+
RP3-406A7.7	0.23	0.001408	-0.07	0.536771	-2.23	0.02605	0.026682	0.9992	+/0
PKN1	-0.16	0.02439	0.14	0.224596	2.23	0.026075	0.026717	0.9992	-/0
C18orf15	0.20	0.005164	-0.10	0.386063	-2.22	0.026214	0.026862	0.9992	+/0
RP1-95L4.4	0.15	0.043406	-0.16	0.174783	-2.22	0.026247	0.0269	0.9992	+/0
RP4-705D16.3	0.04	0.579122	0.33	0.003151	2.22	0.026347	0.02699	0.9992	0/+
YWHAH	0.15	0.03739	-0.15	0.188859	-2.22	0.026488	0.027134	0.9992	+/0
DAP	0.05	0.465571	-0.24	0.031101	-2.22	0.026663	0.027268	0.9992	0/-
FMNL1	0.07	0.339938	-0.23	0.044041	-2.21	0.026813	0.027438	0.9992	0/-
POLB	-0.06	0.375068	0.23	0.039846	2.21	0.026824	0.027454	0.9992	0/+
RP11-209D14.2	-0.03	0.707544	0.27	0.017672	2.21	0.026836	0.027467	0.9992	0/+
RP11-701H16.4	0.19	0.008288	-0.11	0.339574	-2.21	0.026849	0.027495	0.9992	+/0
CTC-325H20.8	-0.07	0.349232	0.23	0.042963	2.21	0.02687	0.027518	0.9992	0/+
PDZK1IP1	0.01	0.914948	-0.29	0.011183	-2.21	0.026903	0.027563	0.9992	0/-
LUZP2	0.03	0.689829	-0.27	0.018456	-2.21	0.026911	0.027563	0.9992	0/-
TMEM74B	-0.05	0.47778	0.25	0.030551	2.21	0.02697	0.027633	0.9992	0/+
PTRH1	0.04	0.565305	-0.25	0.02476	-2.21	0.027051	0.027694	0.9992	0/-
ACKR1	0.01	0.940446	-0.29	0.010657	-2.21	0.02708	0.027719	0.9992	0/-
RN7SL409P	0.17	0.016808	-0.13	0.268334	-2.21	0.027185	0.027822	0.9992	+/0
ANK2	-0.17	0.01679	0.13	0.268496	2.21	0.027192	0.027825	0.9992	-/0
RNU6-711P	0.06	0.446073	-0.24	0.033462	-2.21	0.02721	0.027851	0.9992	0/-
RP11-4O1.2	0.19	0.008123	-0.11	0.345052	-2.21	0.027227	0.027867	0.9992	+/0
LINC01600	-0.16	0.030288	0.14	0.212115	2.21	0.027235	0.027876	0.9992	-/0
SLC6A14	0.06	0.386282	-0.23	0.039486	-2.21	0.027363	0.028004	0.9992	0/-
RP1-12G14.9	0.20	0.006472	-0.10	0.371574	-2.21	0.027378	0.028024	0.9992	+/0
MOB2	-0.30	1.76E-05	-0.01	0.910771	2.21	0.027443	0.028091	0.9992	-/0
CTD-3252C9.2	0.00	0.979655	0.29	0.009071	2.21	0.027447	0.028097	0.9992	0/+
LINC00381	-0.07	0.31267	0.22	0.049089	2.20	0.027494	0.028148	0.9992	0/+
FUT10	-0.22	0.002036	0.08	0.508932	2.20	0.027556	0.028209	0.9992	-/0

KLHDC10	0.22	0.002618	-0.08	0.478788	-2.20	0.027576	0.028222	0.9992	+/0
RP11-1026M7.2	-0.20	0.005199	0.10	0.398696	2.20	0.027627	0.028273	0.9992	-/0
PTAR1	0.18	0.012895	-0.12	0.299139	-2.20	0.027666	0.028318	0.9992	+/0
GMPR2	-0.24	0.000627	0.05	0.656956	2.20	0.027738	0.028411	0.9992	-/0
RP11-803D5.1	0.22	0.001909	-0.07	0.521371	-2.20	0.027983	0.028632	0.9992	+/0
PPP1R8P1	0.07	0.317368	-0.22	0.049448	-2.20	0.02805	0.02867	0.9992	0/-
CD8B	0.22	0.001963	-0.07	0.519331	-2.20	0.028112	0.028731	0.9992	+/0
TSPEAR-AS1	0.03	0.636143	0.32	0.004063	2.20	0.028126	0.028737	0.9992	0/+
EIF4BP3	-0.19	0.007948	0.11	0.356203	2.19	0.028243	0.028853	0.9992	-/0
C16orf46	-0.24	0.000941	0.06	0.613394	2.19	0.028362	0.028978	0.9992	-/0
RP4-740C4.7	0.15	0.043816	-0.15	0.185843	-2.19	0.028411	0.029013	0.9992	+/0
CTD-2369P2.8	-0.06	0.392903	0.23	0.040521	2.19	0.028469	0.02908	0.9992	0/+
TAF3	0.18	0.012436	-0.12	0.309423	-2.19	0.028496	0.029118	0.9992	+/0
C1QB	0.18	0.012054	-0.12	0.313278	-2.19	0.028563	0.02917	0.9992	+/0
TP53	-0.20	0.006082	0.10	0.389321	2.19	0.028567	0.029173	0.9992	-/0
AP000344.3	0.16	0.029049	-0.14	0.224482	-2.19	0.028603	0.029211	0.9992	+/0
KM-PA-2	0.00	0.976474	0.29	0.009587	2.19	0.028771	0.029406	0.9992	0/+
DDX39A	0.26	0.000343	0.51	2.14E-06	2.19	0.028787	0.029419	0.9992	+/+
LINC00640	0.14	0.049353	-0.15	0.17793	-2.19	0.028873	0.029509	0.9992	+/0
ERVK3-1	0.22	0.002059	-0.07	0.522403	-2.18	0.028954	0.029586	0.9992	+/0
RP11-481A20.1	0.01	0.854859	0.30	0.007327	2.18	0.028974	0.029618	0.9992	0/+
KCNK15-AS1	-0.19	0.008865	0.11	0.350533	2.18	0.029015	0.029653	0.9992	-/0
TTF2	0.26	0.000271	0.51	1.83E-06	2.18	0.029056	0.029695	0.9992	+/+
AC005682.6	-0.27	0.000124	0.02	0.877018	2.18	0.029059	0.029701	0.9992	-/0
RP11-1143G9.5	0.05	0.493986	-0.24	0.032	-2.18	0.029082	0.029714	0.9992	0/-
AC010761.14	0.00	0.947946	-0.28	0.011519	-2.18	0.029116	0.029752	0.9992	0/-
COL4A6	-0.24	0.000992	0.06	0.616035	2.18	0.029181	0.0298	0.9992	-/0
CTB-113D17.1	0.00	0.953329	-0.29	0.009319	-2.18	0.029288	0.029896	0.9992	0/-
CTC-510F12.2	-0.14	0.045513	0.15	0.187291	2.18	0.029306	0.029925	0.9992	-/0
RFC5	0.46	1.34E-11	0.20	0.078836	-2.18	0.02944	0.030053	0.9992	+/0
PRMT2	-0.17	0.015018	0.12	0.296546	2.18	0.029449	0.030069	0.9992	-/0
ZBED3	-0.16	0.025153	0.13	0.243771	2.18	0.029453	0.030079	0.9992	-/0
MIR3613	-0.06	0.399993	0.23	0.041418	2.18	0.029539	0.030168	0.9992	0/+
AC005013.5	-0.02	0.825437	-0.30	0.007056	-2.17	0.02965	0.030306	0.9992	0/-
RP11-797D24.3	-0.05	0.474206	-0.33	0.002736	-2.17	0.02977	0.030412	0.9992	0/-
PKLR	0.19	0.009488	-0.11	0.349242	-2.17	0.029786	0.030444	0.9992	+/0
RP11-91P24.5	0.04	0.576467	-0.25	0.027061	-2.17	0.029821	0.030485	0.9992	0/-
PDPK2P	-0.29	3.97E-05	0.00	0.973833	2.17	0.029892	0.030565	0.9992	-/0
MRPS31P5	-0.07	0.363737	0.23	0.046769	2.17	0.030151	0.030844	0.9992	0/+
MSN	0.05	0.484788	-0.24	0.034154	-2.17	0.030178	0.030873	0.9992	0/-
MRPS31P4	-0.04	0.586072	0.25	0.02686	2.17	0.030196	0.030885	0.9992	0/+
SLC36A1	-0.01	0.888202	0.28	0.013792	2.17	0.030244	0.030924	0.9992	0/+
TRPC5OS	-0.01	0.895084	-0.30	0.008593	-2.16	0.030423	0.031094	0.9992	0/-
VSIG10	0.27	0.000119	-0.01	0.899917	-2.16	0.030457	0.031145	0.9992	+/0
RTBDN	-0.01	0.884667	0.28	0.014065	2.16	0.030527	0.031231	0.9992	0/+
PTBP1	0.02	0.799448	0.30	0.006917	2.16	0.030569	0.031263	0.9992	0/+
ZNF778	-0.15	0.036046	0.14	0.216193	2.16	0.030664	0.031359	0.9992	-/0

ANGPTL6	0.02	0.748918	0.31	0.006178	2.16	0.030783	0.031519	0.9992	0/+
CYP4F11	0.05	0.532694	-0.24	0.031206	-2.16	0.030848	0.03159	0.9992	0/-
ZNF100	0.09	0.190658	0.37	0.000838	2.16	0.030955	0.031708	0.9992	0/+
RP11-227G15.1	0.00	0.975386	-0.28	0.011762	-2.16	0.030982	0.031727	0.9992	0/-
AC073343.13	-0.03	0.669762	0.26	0.022925	2.16	0.030988	0.03174	0.9992	0/+
RP11-497H16.2	0.03	0.677414	-0.26	0.022623	-2.16	0.031084	0.031859	0.9992	0/-
DHRS4L1	0.17	0.020089	-0.12	0.278047	-2.15	0.031191	0.031974	0.9992	+/0
ZNF887P	0.19	0.008014	0.45	3.35E-05	2.15	0.031219	0.032003	0.9992	+/+
RP11-343H5.6	-0.05	0.522412	0.24	0.032458	2.15	0.031246	0.032019	0.9992	0/+
RP11-1334A24.	0.06	0.382682	0.34	0.002149	2.15	0.031328	0.032115	0.9992	0/+
NDUFA13	-0.02	0.809713	0.27	0.017104	2.15	0.031329	0.032115	0.9992	0/+
CTA-384D8.36	0.27	0.000156	-0.02	0.876577	-2.15	0.031365	0.032147	0.9992	+/0
XXbac-BPG308I	-0.01	0.859557	0.27	0.015389	2.15	0.031385	0.03216	0.9992	0/+
CTD-2035E11.5	0.17	0.0211	-0.13	0.274214	-2.15	0.03139	0.032163	0.9992	+/0
RN7SL569P	0.29	5.28E-05	0.00	0.989298	-2.15	0.031398	0.032169	0.9992	+/0
RP1-80N2.4	-0.14	0.048136	0.15	0.193408	2.15	0.031449	0.03223	0.9992	-/0
PPHLN1	0.14	0.045716	-0.15	0.198501	-2.15	0.031515	0.032297	0.9992	+/0
RP11-696F12.1	0.17	0.016225	-0.12	0.303086	-2.15	0.031516	0.0323	0.9992	+/0
AC139099.5	0.01	0.840522	-0.27	0.016171	-2.15	0.031593	0.032377	0.9992	0/-
RP11-723C11.2	0.15	0.033406	-0.14	0.229223	-2.15	0.031657	0.032435	0.9992	+/0
LEF1-AS1	0.17	0.019856	-0.12	0.282386	-2.15	0.031658	0.032435	0.9992	+/0
UBQLN4	-0.18	0.011451	0.11	0.343157	2.15	0.031714	0.032496	0.9992	-/0
RP11-335O13.8	-0.19	0.007918	0.10	0.386181	2.15	0.031782	0.032553	0.9992	-/0
Y_RNA.733	-0.05	0.525293	0.24	0.032953	2.15	0.031843	0.032614	0.9992	0/+
CTD-2006C1.2	0.00	0.94864	0.29	0.010373	2.14	0.031989	0.032793	0.9992	0/+
NPIPA8	0.04	0.603396	-0.25	0.027707	-2.14	0.032049	0.032864	0.9992	0/-
LGALS12	0.03	0.6843	-0.26	0.023138	-2.14	0.032063	0.03288	0.9992	0/-
ITPR1-AS1	-0.18	0.012237	0.11	0.338328	2.14	0.03207	0.03288	0.9992	-/0
ETV6	0.01	0.912162	-0.28	0.014213	-2.14	0.032233	0.033053	0.9992	0/-
CORO2B	-0.08	0.289604	-0.35	0.001529	-2.14	0.032257	0.033088	0.9992	0/-
PDK2	-0.26	0.000223	0.02	0.84374	2.14	0.032417	0.033261	0.9992	-/0
AC016735.2	0.04	0.546412	-0.24	0.032018	-2.14	0.032419	0.033264	0.9992	0/-
CCDC183-AS1	0.03	0.712197	0.31	0.006082	2.14	0.032487	0.033331	0.9992	0/+
NRGN	0.14	0.048599	-0.15	0.197913	-2.14	0.032508	0.033347	0.9992	+/0
GLDN	0.21	0.003761	-0.08	0.483584	-2.14	0.032627	0.033453	0.9992	+/0
LRRC3	0.00	0.960712	0.29	0.011001	2.13	0.032765	0.033584	0.9992	0/+
RP11-803D5.4	0.22	0.002669	-0.07	0.52806	-2.13	0.032781	0.033597	0.9992	+/0
PCDHGA7	-0.21	0.003069	0.08	0.510575	2.13	0.032797	0.033613	0.9992	-/0
B3GALNT2	0.03	0.697874	0.31	0.005955	2.13	0.032832	0.033651	0.9992	0/+
RPL7P23	-0.17	0.016465	0.12	0.31109	2.13	0.0329	0.033712	0.9992	-/0
RP11-1094H24.	-0.03	0.651638	-0.31	0.005311	-2.13	0.032946	0.033763	0.9992	0/-
SULT1C2	0.17	0.020346	-0.12	0.2887	-2.13	0.03301	0.033837	0.9992	+/0
YWHAQP5	0.21	0.004003	-0.08	0.479722	-2.13	0.033062	0.033904	0.9992	+/0
ATG4D	0.12	0.108884	0.39	0.00049	2.13	0.033259	0.034087	0.9992	0/+
LCP1	0.15	0.039633	-0.14	0.221431	-2.13	0.033272	0.034096	0.9992	+/0
RP11-131L23.1	0.28	0.000108	-0.01	0.945085	-2.13	0.033293	0.034135	0.9992	+/0
UBE4B	-0.25	0.000365	0.03	0.790722	2.13	0.033315	0.034157	0.9992	-/0

SLC4A1	0.20	0.005189	-0.09	0.450357	-2.13	0.033379	0.034237	0.9992	+/0
LRRC4B	-0.18	0.013583	0.11	0.337271	2.13	0.033552	0.034397	0.9992	-/0
TLDC1	-0.28	7.28E-05	0.00	0.998315	2.12	0.033679	0.034535	0.9992	-/0
CSRP1	-0.02	0.778243	-0.30	0.007544	-2.12	0.033758	0.034631	0.9992	0/-
DNAJB13	-0.22	0.001876	0.06	0.582983	2.12	0.033806	0.034701	0.9992	-/0
PCP4L1	-0.17	0.018439	0.12	0.30471	2.12	0.033809	0.034705	0.9992	-/0
MAPKAPK2	-0.27	0.000174	0.02	0.891393	2.12	0.03384	0.034733	0.9992	-/0
RP5-867C24.4	0.18	0.012104	-0.11	0.352723	-2.12	0.033858	0.034746	0.9992	+/0
NLRP7	-0.17	0.021682	0.12	0.287401	2.12	0.033877	0.034772	0.9992	-/0
MDK	0.16	0.022223	-0.12	0.285325	-2.12	0.033971	0.034877	0.9992	+/0
RP11-181E10.3	0.25	0.000538	-0.04	0.748123	-2.12	0.034045	0.034961	0.9992	+/0
FAM66D	-0.19	0.007259	0.09	0.415569	2.12	0.034137	0.035031	0.9992	-/0
APOBEC3H	0.19	0.009767	-0.10	0.38018	-2.12	0.034167	0.035069	0.9992	+/0
A3GALT2	0.20	0.004396	-0.08	0.478147	-2.12	0.034212	0.035092	0.9992	+/0
SLC18A2	-0.20	0.005641	0.09	0.448653	2.12	0.034402	0.035319	0.9992	-/0
CTD-2357A8.2	0.15	0.034762	-0.13	0.240884	-2.12	0.034425	0.035348	0.9992	+/0
CTD-2319I12.1(	0.05	0.497777	0.32	0.003699	2.11	0.034669	0.035662	0.9992	0/+
MED10	0.22	0.001993	-0.06	0.584225	-2.11	0.034755	0.035755	0.9992	+/0
SYT16	0.06	0.444484	-0.23	0.044336	-2.11	0.034775	0.03578	0.9992	0/-
RP11-353M9.1	0.24	0.000665	-0.04	0.729913	-2.11	0.03495	0.035995	0.9992	+/0
RP11-76E17.3	-0.02	0.825924	-0.29	0.008864	-2.11	0.034978	0.03603	0.9992	0/-
ZNF699	-0.05	0.448384	0.23	0.044272	2.11	0.035029	0.036097	0.9992	0/+
RARRES3	0.03	0.64942	-0.25	0.027816	-2.11	0.035034	0.03611	0.9992	0/-
LY75	0.02	0.831466	-0.27	0.018801	-2.11	0.035039	0.036119	0.9992	0/-
CTD-2291D10.4	0.19	0.008534	0.45	4.44E-05	2.11	0.035051	0.036123	0.9992	+/+
IRGC	-0.01	0.888592	-0.29	0.010272	-2.11	0.035093	0.036174	0.9992	0/-
SAA1	-0.01	0.854906	-0.29	0.009535	-2.11	0.03514	0.036212	0.9992	0/-
RP11-150O12.3	-0.09	0.215345	-0.36	0.001201	-2.11	0.035197	0.036273	0.9992	0/-
LINC01521	-0.16	0.027894	0.13	0.268352	2.11	0.035201	0.036276	0.9992	-/0
AC010524.2	-0.03	0.717743	0.26	0.024162	2.11	0.035257	0.036344	0.9992	0/+
CCDC130	0.01	0.843678	0.29	0.009373	2.10	0.035362	0.036468	0.9992	0/+
TTC31	-0.14	0.047549	0.14	0.214968	2.10	0.035486	0.036558	0.9992	-/0
RPL21P75	-0.17	0.021429	0.12	0.298866	2.10	0.035487	0.036558	0.9992	-/0
RP11-227G15.1	-0.02	0.734319	-0.30	0.007282	-2.10	0.035509	0.03659	0.9992	0/-
RP11-236F9.2	0.20	0.004607	-0.08	0.483845	-2.10	0.035573	0.036641	0.9992	+/0
RP11-117D22.2	0.15	0.0389	-0.14	0.235742	-2.10	0.035598	0.036664	0.9992	+/0
STX10	-0.04	0.607513	0.24	0.031138	2.10	0.035627	0.036686	0.9992	0/+
ZNF840P	0.00	0.997228	0.28	0.013489	2.10	0.035652	0.036702	0.9992	0/+
AOC4P	0.01	0.922485	-0.27	0.015855	-2.10	0.035684	0.036734	0.9992	0/-
MAJIN	0.00	0.9625	-0.28	0.01245	-2.10	0.035849	0.03691	0.9992	0/-
FBP1	0.04	0.569491	-0.24	0.03419	-2.10	0.035872	0.036939	0.9992	0/-
SIAH3	-0.17	0.016783	0.11	0.329085	2.10	0.035884	0.036955	0.9992	-/0
PCDHA8	-0.20	0.006081	0.09	0.451839	2.10	0.035933	0.037035	0.9992	-/0
RP4-580N22.2	-0.15	0.040066	0.14	0.234769	2.10	0.035986	0.037089	0.9992	-/0
EEF1A1P12	-0.15	0.038745	0.14	0.238293	2.10	0.036001	0.037109	0.9992	-/0
RP11-89B16.2	0.16	0.029767	-0.13	0.266896	-2.10	0.036152	0.037269	0.9992	+/0
STC1	0.04	0.537365	-0.24	0.03713	-2.10	0.036152	0.037269	0.9992	0/-

RP11-163E9.2	0.04	0.58573	0.31	0.00509	2.09	0.036175	0.037288	0.9992	0/+
EXOC3	0.03	0.660983	-0.25	0.028231	-2.09	0.036217	0.037346	0.9992	0/-
LINC00051	-0.06	0.434209	0.22	0.047687	2.09	0.036268	0.037397	0.9992	0/+
SLFN5	-0.07	0.336518	-0.34	0.002257	-2.09	0.036333	0.037467	0.9992	0/-
UPP1	0.17	0.017558	-0.11	0.326886	-2.09	0.03634	0.037474	0.9992	+/0
RP11-180M15.7	0.26	0.000263	-0.02	0.86657	-2.09	0.036454	0.037618	0.9992	+/0
FAM105A	0.18	0.011242	-0.10	0.381178	-2.09	0.036587	0.03773	0.9992	+/0
MAP3K14	0.03	0.645638	-0.25	0.029558	-2.09	0.036593	0.037736	0.9992	0/-
CH25H	-0.01	0.926496	-0.28	0.01185	-2.09	0.036651	0.0378	0.9992	0/-
CYP4F30P	-0.04	0.607339	0.24	0.032293	2.09	0.036723	0.037864	0.9992	0/+
RP11-405L18.4	0.02	0.802125	0.29	0.008979	2.09	0.036751	0.03789	0.9992	0/+
RAB8A	0.00	0.985813	0.28	0.013611	2.09	0.036905	0.038043	0.9992	0/+
RP5-875H18.9	0.02	0.831691	-0.26	0.020107	-2.09	0.036963	0.038127	0.9992	0/-
LINC00612	-0.15	0.03316	0.13	0.260439	2.09	0.037067	0.038216	0.9992	-/0
CTAGE6	0.26	0.000298	-0.02	0.85713	-2.08	0.037122	0.03829	0.9992	+/0
SLCO2A1	0.16	0.027488	-0.12	0.281373	-2.08	0.037159	0.038316	0.9992	+/0
NVL	0.08	0.250093	0.35	0.001587	2.08	0.037217	0.038396	0.9992	0/+
RP11-283G6.4	0.03	0.700601	-0.25	0.026884	-2.08	0.037327	0.038533	0.9992	0/-
CTD-2371O3.2	0.04	0.615708	0.31	0.005791	2.08	0.03738	0.038594	0.9992	0/+
RP11-504P24.9	-0.04	0.611231	0.24	0.032702	2.08	0.037383	0.0386	0.9992	0/+
RP11-359K18.4	0.16	0.024999	-0.12	0.293357	-2.08	0.037403	0.038629	0.9992	+/0
GATS	-0.23	0.001061	0.05	0.692909	2.08	0.037447	0.038655	0.9992	-/0
RN7SL589P	-0.15	0.037736	0.13	0.249256	2.08	0.037551	0.03876	0.9992	-/0
PLA2G16	-0.11	0.127111	-0.37	0.000718	-2.08	0.037587	0.038783	0.9992	0/-
RAD23A	0.03	0.704688	0.30	0.007342	2.08	0.037611	0.038818	0.9992	0/+
RHEBL1	0.30	2.78E-05	0.02	0.843672	-2.08	0.037775	0.038969	0.9992	+/0
JAZF1	-0.10	0.146799	-0.37	0.000854	-2.08	0.037786	0.038978	0.9992	0/-
USP30-AS1	0.17	0.021126	-0.12	0.315531	-2.08	0.037908	0.03909	0.9992	+/0
LCN8	0.05	0.479662	-0.23	0.044911	-2.08	0.03793	0.039109	0.9992	0/-
SEC22C	0.16	0.027773	-0.12	0.285347	-2.07	0.038046	0.039218	0.9992	+/0
GCNT6	-0.04	0.538774	0.23	0.039336	2.07	0.038097	0.039263	0.9992	0/+
PPIP5K2	-0.03	0.715972	0.25	0.026687	2.07	0.038113	0.039289	0.9992	0/+
APOL2	0.06	0.441662	-0.22	0.04958	-2.07	0.038169	0.039334	0.9992	0/-
SSH3	-0.11	0.13619	-0.37	0.000799	-2.07	0.03821	0.039362	0.9992	0/-
CCDC150	0.24	0.000957	0.48	8.69E-06	2.07	0.038221	0.039378	0.9992	+/+
PPP2R5D	0.04	0.604104	0.31	0.005821	2.07	0.038331	0.039497	0.9992	0/+
GATAD2A	-0.03	0.715068	0.25	0.026952	2.07	0.038361	0.039519	0.9992	0/+
PROSER1	0.08	0.255725	0.35	0.001716	2.07	0.038396	0.039561	0.9992	0/+
WIPF2	-0.03	0.635102	-0.31	0.00635	-2.07	0.038455	0.039628	0.9992	0/-
ITGB2-AS1	-0.01	0.863822	-0.29	0.011019	-2.07	0.038499	0.03967	0.9992	0/-
CCL19	0.18	0.014206	-0.10	0.366159	-2.07	0.038499	0.039673	0.9992	+/0
MAP3K5	-0.03	0.701964	-0.30	0.007547	-2.07	0.038533	0.039708	0.9992	0/-
NCEH1	0.23	0.001116	-0.04	0.696923	-2.07	0.038583	0.039775	0.9992	+/0
KRI1	0.07	0.343715	0.34	0.002552	2.07	0.038593	0.039788	0.9992	0/+
CD83	0.25	0.000418	-0.03	0.827912	-2.07	0.038599	0.039798	0.9992	+/0
RP11-301O19.1	-0.05	0.450966	0.22	0.049091	2.07	0.038606	0.039804	0.9992	0/+
HSPA8P4	0.19	0.007904	-0.09	0.43973	-2.07	0.038636	0.039862	0.9992	+/0

RP11-434D11.4	0.33	2.64E-06	0.06	0.592785	-2.07	0.038639	0.039871	0.9992	+/0
IGFBP2	-0.04	0.593588	0.24	0.035377	2.07	0.038653	0.039894	0.9992	0/+
CECR6	0.14	0.047069	-0.14	0.231638	-2.07	0.038653	0.039894	0.9992	+/0
KDSR	-0.11	0.128814	-0.37	0.000771	-2.07	0.038857	0.040099	0.9992	0/-
IL4R	0.02	0.736598	-0.25	0.026174	-2.07	0.038874	0.040131	0.9992	0/-
TUSC5	0.15	0.034807	-0.13	0.265123	-2.07	0.038898	0.040143	0.9992	+/0
GAREM2	0.01	0.882861	0.28	0.011713	2.06	0.039029	0.0403	0.9992	0/+
MROH7	0.03	0.675209	-0.25	0.030004	-2.06	0.039066	0.040339	0.9992	0/-
HMGCS2	0.05	0.480723	-0.23	0.0464	-2.06	0.039117	0.040403	0.9992	0/-
IGHA1	0.17	0.019202	-0.11	0.334696	-2.06	0.039205	0.040528	0.9992	+/0
SHB	-0.07	0.307838	-0.34	0.002266	-2.06	0.03931	0.040614	0.9992	0/-
RP11-417B4.2	0.18	0.012914	-0.10	0.383379	-2.06	0.039332	0.040617	0.9992	+/0
RPL7AP10	-0.04	0.616276	0.24	0.034413	2.06	0.039384	0.040672	0.9992	0/+
PLAG1	-0.17	0.018178	0.11	0.342504	2.06	0.039413	0.040694	0.9992	-/0
Y_RNA.187	0.15	0.03967	-0.13	0.253674	-2.06	0.039428	0.040713	0.9992	+/0
HMCES	0.22	0.002238	-0.06	0.61133	-2.06	0.039433	0.04072	0.9992	+/0
LRCH4	-0.23	0.001648	0.05	0.652849	2.06	0.039487	0.040761	0.9992	-/0
RP11-106M7.1	-0.03	0.715827	-0.30	0.008124	-2.06	0.039629	0.040918	0.9992	0/-
RP11-644F5.11	-0.13	0.06854	-0.39	0.000405	-2.06	0.039704	0.040989	0.9992	0/-
FARSA	0.01	0.938319	0.28	0.013543	2.06	0.039716	0.041001	0.9992	0/+
RP11-172H24.4	0.16	0.023036	0.42	0.000139	2.06	0.039771	0.041075	0.9992	+/+
KLF1	-0.01	0.923907	0.27	0.018196	2.06	0.03978	0.041085	0.9992	0/+
CKAP4	0.00	0.980454	-0.27	0.014866	-2.06	0.039791	0.041101	0.9992	0/-
MORC2	-0.02	0.732161	0.25	0.027264	2.06	0.039878	0.041171	0.9992	0/+
PYCR1	0.05	0.494822	0.32	0.004524	2.05	0.039923	0.041222	0.9992	0/+
PRKDC	0.20	0.006451	0.44	4.52E-05	2.05	0.039971	0.041254	0.9992	+/+
PTPRQ	-0.18	0.013328	0.10	0.384451	2.05	0.040071	0.04135	0.9992	-/0
RP11-597D13.9	-0.20	0.004688	0.07	0.518288	2.05	0.040071	0.04135	0.9992	-/0
HSPA2	-0.05	0.477042	0.22	0.04817	2.05	0.040118	0.041398	0.9992	0/+
QARS	-0.21	0.003217	0.07	0.568743	2.05	0.040134	0.041405	0.9992	-/0
CABP4	0.00	0.958339	-0.28	0.014355	-2.05	0.040165	0.041424	0.9992	0/-
RPL7AP60	0.18	0.010767	-0.09	0.412159	-2.05	0.040231	0.041475	0.9992	+/0
CTB-113P19.5	-0.07	0.322692	-0.34	0.002513	-2.05	0.040344	0.041635	0.9992	0/-
KB-1562D12.1	-0.26	0.000222	0.01	0.928246	2.05	0.040419	0.04168	0.9992	-/0
RP5-858L17.1	-0.20	0.005803	0.08	0.493911	2.05	0.04054	0.041827	0.9992	-/0
HOXD13	0.18	0.010236	-0.09	0.420785	-2.05	0.040553	0.041834	0.9992	+/0
RP11-84C13.2	-0.16	0.029367	0.12	0.294496	2.05	0.040795	0.042096	0.9992	-/0
CTC-273B12.10	-0.02	0.733945	0.25	0.027991	2.04	0.040863	0.042192	0.9992	0/+
AC020550.7	0.21	0.003525	-0.07	0.562596	-2.04	0.040873	0.042192	0.9992	+/0
CASQ2	-0.18	0.012309	0.10	0.399974	2.04	0.040911	0.042218	0.9992	-/0
SLC9B2	-0.18	0.010033	0.09	0.426109	2.04	0.040952	0.042263	0.9992	-/0
ASNSP1	0.00	0.954391	0.27	0.017767	2.04	0.041027	0.042359	0.9992	0/+
TNFAIP8L3	-0.02	0.806009	-0.29	0.010586	-2.04	0.041131	0.042484	0.9992	0/-
RP11-275F13.3	0.24	0.000702	-0.03	0.783485	-2.04	0.041206	0.04258	0.9992	+/0
PRRT1	-0.23	0.001391	0.05	0.691533	2.04	0.041268	0.04265	0.9992	-/0
DDX39B-AS1	-0.03	0.668839	-0.30	0.007669	-2.04	0.041299	0.042688	0.9992	0/-
PADI3	0.02	0.8031	0.29	0.010604	2.04	0.041381	0.042768	0.9992	0/+

LINC01353	0.05	0.502115	-0.23	0.047264	-2.04	0.041529	0.042916	0.9992	0/-
AC092620.3	0.03	0.64096	-0.24	0.034764	-2.04	0.041534	0.042919	0.9992	0/-
RP11-44N21.1	-0.09	0.222507	-0.35	0.001643	-2.04	0.041623	0.043009	0.9992	0/-
DUSP8	-0.25	0.000371	0.02	0.872909	2.04	0.041655	0.043044	0.9992	-/0
ALDH1A1	0.04	0.626621	-0.24	0.036039	-2.04	0.041715	0.04314	0.9992	0/-
FUNDC2P2	-0.03	0.690816	0.24	0.031471	2.04	0.041757	0.043191	0.9992	0/+
VWA8	-0.26	0.000344	0.02	0.883791	2.04	0.041771	0.043201	0.9992	-/0
BTG3	0.22	0.002146	-0.05	0.637111	-2.04	0.041786	0.043217	0.9992	+/0
RFXAP	0.12	0.107042	0.38	0.000713	2.04	0.041844	0.043281	0.9992	0/+
NDST2	0.02	0.743304	0.29	0.009379	2.03	0.041873	0.043322	0.9992	0/+
KLKP1	0.15	0.035812	-0.12	0.27769	-2.03	0.041885	0.043329	0.9992	+/0
RP11-164P12.5	0.00	0.971174	-0.27	0.015624	-2.03	0.04193	0.043383	0.9992	0/-
RP5-856G1.2	-0.04	0.56382	0.23	0.041599	2.03	0.041944	0.043396	0.9992	0/+
EHD4-AS1	0.01	0.899261	0.28	0.013389	2.03	0.041945	0.043396	0.9992	0/+
Y_RNA.781	0.17	0.019963	-0.11	0.346984	-2.03	0.041979	0.043431	0.9992	+/0
GPBAR1	0.21	0.003804	-0.07	0.561648	-2.03	0.042036	0.043485	0.9992	+/0
EFR3B	0.09	0.196476	0.35	0.001437	2.03	0.042036	0.043485	0.9992	0/+
ALDH3B1	-0.18	0.012058	-0.43	8.62E-05	-2.03	0.042077	0.043527	0.9992	-/-
CTD-2332E11.2	-0.19	0.007982	0.08	0.463818	2.03	0.042115	0.043562	0.9992	-/0
VHL	-0.05	0.527696	0.23	0.045314	2.03	0.042117	0.043562	0.9992	0/+
AC011475.1	0.05	0.469757	0.32	0.004546	2.03	0.042173	0.043633	0.9992	0/+
AP000580.1	-0.15	0.039223	0.13	0.268947	2.03	0.042185	0.043633	0.9992	-/0
BICC1	-0.01	0.936662	-0.28	0.014649	-2.03	0.042226	0.043674	0.9992	0/-
RNU6-315P	0.18	0.012225	-0.09	0.409902	-2.03	0.042271	0.04371	0.9992	+/0
FOXD4	0.19	0.008531	-0.09	0.456375	-2.03	0.04229	0.043732	0.9992	+/0
RAB11FIP5	-0.27	0.000143	0.00	0.997344	2.03	0.042306	0.043764	0.9992	-/0
CTSE	0.05	0.508795	-0.22	0.047669	-2.03	0.042401	0.043838	0.9992	0/-
RP4-809F18.1	-0.02	0.83039	-0.28	0.011703	-2.03	0.042496	0.043969	0.9992	0/-
LGALS17A	0.21	0.003667	-0.07	0.57055	-2.03	0.042535	0.044052	0.9992	+/0
HBS1L	0.18	0.012408	-0.09	0.410212	-2.03	0.042606	0.044119	0.9992	+/0
C19orf45	0.01	0.931137	0.28	0.014667	2.03	0.04264	0.044142	0.9992	0/+
GINM1	0.01	0.917376	-0.26	0.020194	-2.03	0.042663	0.044161	0.9992	0/-
CAMK1G	-0.18	0.011483	0.09	0.421275	2.03	0.04278	0.044251	0.9992	-/0
LINC00202-2	0.03	0.67375	0.30	0.008171	2.03	0.042802	0.044267	0.9992	0/+
LIPC	-0.02	0.793088	-0.29	0.010889	-2.02	0.042884	0.044356	0.9992	0/-
PLEKHG7	0.01	0.926936	-0.26	0.019959	-2.02	0.042933	0.044398	0.9992	0/-
CARNMT1	0.01	0.898887	-0.26	0.021192	-2.02	0.042995	0.044446	0.9992	0/-
ACAD9	0.20	0.005961	-0.08	0.508891	-2.02	0.043014	0.044471	0.9992	+/0
RP11-603J24.4	-0.02	0.79734	-0.29	0.011052	-2.02	0.043043	0.044491	0.9992	0/-
ASPH	-0.04	0.590989	0.23	0.040426	2.02	0.043065	0.044513	0.9992	0/+
AC007919.18	0.27	0.000159	0.00	0.998383	-2.02	0.043296	0.044785	0.9992	+/0
DEF8	-0.18	0.013176	0.10	0.407652	2.02	0.043389	0.044878	0.9992	-/0
MCM4	0.41	3.71E-09	0.61	3.03E-09	2.02	0.043459	0.044945	0.9992	+/+
EIF4A2P1	-0.04	0.550926	0.23	0.044615	2.02	0.04346	0.044945	0.9992	0/+
METTL3	0.03	0.695688	0.29	0.00882	2.02	0.043478	0.044984	0.9992	0/+
RP3-467K16.7	-0.04	0.586938	0.23	0.041274	2.02	0.043503	0.045012	0.9992	0/+
RNF219-AS1	0.02	0.831249	0.28	0.012114	2.02	0.043516	0.045022	0.9992	0/+

TYK2	0.05	0.526519	0.31	0.005658	2.02	0.04356	0.045064	0.9992	0/+
MYO5C	-0.02	0.760652	-0.29	0.010352	-2.02	0.043642	0.045128	0.9992	0/-
RP11-554A11.8	0.01	0.888854	0.28	0.013837	2.02	0.043703	0.045208	0.9992	0/+
RP3-332B22.1	-0.23	0.001161	0.04	0.737271	2.02	0.043708	0.045214	0.9992	-/0
PLEKHA1	-0.21	0.003999	0.07	0.568469	2.02	0.04378	0.045275	0.9992	-/0
FGF16	0.18	0.014155	-0.10	0.401108	-2.02	0.043799	0.045291	0.9992	+/0
RIC8A	-0.23	0.001356	0.04	0.716995	2.02	0.043817	0.04531	0.9992	-/0
ANKLE1	0.02	0.739286	0.29	0.009907	2.02	0.043832	0.045329	0.9992	0/+
C1orf74	0.05	0.506492	0.31	0.005428	2.01	0.04406	0.045569	0.9992	0/+
CTC-559E9.6	-0.14	0.044918	0.13	0.262932	2.01	0.044071	0.045582	0.9992	-/0
TRAV17	0.18	0.012069	-0.09	0.423401	-2.01	0.044076	0.045589	0.9992	+/0
AP000255.6	-0.15	0.036238	0.12	0.287876	2.01	0.044132	0.045656	0.9992	-/0
ARFGEF1	-0.23	0.001275	0.04	0.728367	2.01	0.044169	0.045678	0.9992	-/0
ATF6B	-0.17	0.021638	0.11	0.350154	2.01	0.044193	0.045707	0.9992	-/0
ABI3	0.18	0.013173	-0.09	0.413816	-2.01	0.044345	0.045851	0.9992	+/0
STAG3L2	0.11	0.135039	0.36	0.001023	2.01	0.044448	0.045941	0.9992	0/+
CADM3	0.00	0.963731	-0.27	0.016662	-2.01	0.044532	0.046021	0.9992	0/-
AC005534.8	0.34	1.43E-06	0.08	0.493491	-2.01	0.044543	0.046024	0.9992	+/0
INSIG1	0.39	1.69E-08	0.14	0.222927	-2.01	0.044558	0.046043	0.9992	+/0
MCF2L2	0.19	0.009419	-0.09	0.45909	-2.01	0.044566	0.046053	0.9992	+/0
HIST1H2APS3	0.02	0.775989	0.29	0.01107	2.01	0.044638	0.046143	0.9992	0/+
S1PR2	-0.01	0.935991	0.26	0.020623	2.01	0.044671	0.046171	0.9992	0/+
RP11-960L18.1	0.05	0.516257	-0.22	0.049849	-2.01	0.044737	0.046245	0.9992	0/-
Y_RNA.495	-0.04	0.578354	0.23	0.043487	2.01	0.044763	0.04628	0.9992	0/+
RP13-16H11.8	0.13	0.069772	0.38	0.000511	2.01	0.044772	0.04629	0.9992	0/+
CTD-2192J16.2	-0.02	0.75874	0.25	0.02979	2.01	0.044805	0.046322	0.9992	0/+
DUSP4	-0.05	0.524592	-0.31	0.00587	-2.01	0.044822	0.046335	0.9992	0/-
MIR4653	0.00	0.991074	0.27	0.017834	2.01	0.044885	0.046418	0.9992	0/+
AL590452.1	0.01	0.930671	-0.26	0.021032	-2.00	0.04497	0.046495	0.9992	0/-
STAT3	-0.09	0.213657	-0.35	0.00178	-2.00	0.045047	0.046565	0.9992	0/-
LDLRAD4	-0.01	0.942764	-0.27	0.016194	-2.00	0.045066	0.046584	0.9992	0/-
PARP12	0.32	5.41E-06	0.06	0.606664	-2.00	0.045096	0.046626	0.9992	+/0
RP3-329A5.1	-0.01	0.845633	0.25	0.025155	2.00	0.04513	0.046668	0.9992	0/+
TSSC4	-0.23	0.001056	0.03	0.762498	2.00	0.04515	0.046684	0.9992	-/0
ZNF132	-0.14	0.044595	0.13	0.268954	2.00	0.04516	0.046696	0.9992	-/0
WTH3DI	-0.04	0.557522	0.23	0.046129	2.00	0.045283	0.04685	0.9992	0/+
snoU13.403	0.00	0.949316	0.27	0.016545	2.00	0.045323	0.046882	0.9992	0/+
RP11-145H9.3	-0.15	0.041899	0.12	0.277178	2.00	0.045375	0.046949	0.9992	-/0
GRK4	0.00	0.998636	0.27	0.018507	2.00	0.045416	0.047007	0.9992	0/+
AL133493.2	-0.04	0.605786	0.23	0.041818	2.00	0.045528	0.047132	0.9992	0/+
IFI35	0.15	0.03325	-0.12	0.305222	-2.00	0.045535	0.047145	0.9992	+/0
CTXN2	-0.02	0.748661	0.24	0.031053	2.00	0.045565	0.047193	0.9992	0/+
RP11-857B24.1	-0.04	0.569472	0.23	0.04532	2.00	0.04561	0.047228	0.9992	0/+
LRRC8C	0.20	0.006506	-0.07	0.5162	-2.00	0.045658	0.047301	0.9992	+/0
ADGRA3	-0.20	0.00608	0.07	0.526045	2.00	0.045749	0.047391	0.9992	-/0
PTPRT	0.04	0.58204	-0.23	0.04434	-2.00	0.04582	0.047449	0.9992	0/-
AC005682.5	-0.21	0.002783	0.05	0.635033	2.00	0.045951	0.047612	0.9992	-/0

EFCAB14-AS1	0.16	0.024741	0.41	0.000196	2.00	0.045954	0.047618	0.9992	+/+
KRTAP5-1	-0.15	0.034694	0.12	0.30233	2.00	0.045964	0.047637	0.9992	-/0
LGR4	-0.03	0.633569	0.23	0.039933	2.00	0.046025	0.047721	0.9992	0/+
AC004754.3	-0.04	0.605865	-0.30	0.007631	-2.00	0.046032	0.047734	0.9992	0/-
AC016716.2	0.22	0.002222	-0.05	0.667021	-1.99	0.046043	0.047737	0.9992	+/0
ANGPT4	-0.17	0.021701	0.10	0.360915	1.99	0.046133	0.047814	0.9992	-/0
GINS4	0.15	0.042479	0.40	0.000331	1.99	0.046256	0.047948	0.9992	+/+
RP11-91G21.1	0.00	0.987105	-0.26	0.019596	-1.99	0.046576	0.048268	0.9992	0/-
ZNF66	0.03	0.70169	0.29	0.009879	1.99	0.046609	0.04831	0.9992	0/+
MIR155HG	0.23	0.001561	-0.04	0.720669	-1.99	0.046628	0.048329	0.9992	+/0
CTC-428G20.6	0.01	0.89029	-0.26	0.023962	-1.99	0.046662	0.048351	0.9992	0/-
RP11-167N4.2	-0.21	0.003673	0.06	0.602517	1.99	0.046734	0.048447	0.9992	-/0
ZNF462	-0.24	0.000803	0.03	0.81432	1.99	0.04683	0.048547	0.9992	-/0
RRP1	0.07	0.348872	0.33	0.003529	1.99	0.04688	0.048598	0.9992	0/+
CMTM1	-0.22	0.002591	0.05	0.652926	1.99	0.046982	0.048697	0.9992	-/0
SERPINB1	-0.01	0.896566	-0.27	0.015552	-1.99	0.047057	0.0488	0.9992	0/-
RP11-676J12.8	0.24	0.000946	-0.03	0.79511	-1.98	0.04725	0.048972	0.9992	+/0
Z84812.4	0.01	0.849844	0.28	0.014143	1.98	0.047275	0.048998	0.9992	0/+
PPAN	0.01	0.907293	0.27	0.016047	1.98	0.04735	0.049078	0.9992	0/+
DNAJC27	-0.14	0.047897	0.13	0.271457	1.98	0.04746	0.049213	0.9992	-/0
PBX2	-0.14	0.048958	0.13	0.269184	1.98	0.047522	0.04927	0.9992	-/0
CGB2	0.11	0.121245	0.36	0.001021	1.98	0.047605	0.049363	0.9992	0/+
THAP1	-0.20	0.006163	0.07	0.537354	1.98	0.047619	0.049376	0.9992	-/0
CHD7	-0.03	0.69884	0.24	0.036405	1.98	0.047715	0.049462	0.9992	0/+
METTL17	0.09	0.188787	0.35	0.001691	1.98	0.047725	0.049475	0.9992	0/+
S100Z	0.00	0.981039	-0.27	0.018979	-1.98	0.047794	0.049549	0.9992	0/-
CTC-459F4.1	-0.04	0.557005	0.22	0.049199	1.98	0.047811	0.049555	0.9992	0/+
LINC01068	0.01	0.912962	0.27	0.016461	1.98	0.047822	0.049565	0.9992	0/+
HSDL1	-0.17	0.015162	0.09	0.417586	1.98	0.04791	0.049651	0.9992	-/0
NF1	-0.05	0.504366	-0.31	0.006114	-1.98	0.047913	0.049654	0.9992	0/-
SLC44A3	0.02	0.774172	-0.24	0.031459	-1.98	0.048022	0.049782	0.9992	0/-
RPL18A	-0.19	0.0087	0.08	0.493234	1.98	0.04808	0.04985	0.9992	-/0
EPHA4	-0.15	0.03264	0.11	0.320521	1.98	0.048083	0.04985	0.9992	-/0
CTD-2143L24.1	0.15	0.038325	-0.12	0.301078	-1.98	0.048118	0.049891	0.9992	+/0
RP11-575F12.2	-0.24	0.000962	0.03	0.801979	1.97	0.048382	0.050182	0.9992	-/0
RHOXF1P2	0.05	0.470636	0.31	0.00561	1.97	0.0484	0.050192	0.9992	0/+
WDR64	-0.14	0.045877	0.12	0.281597	1.97	0.048559	0.050397	0.9992	-/0
MSL3P1	-0.22	0.001719	0.04	0.722681	1.97	0.048578	0.050413	0.9992	-/0
SLC30A9	-0.19	0.009548	0.08	0.483983	1.97	0.048605	0.050451	0.9992	-/0
PPP2R2C	0.00	0.99219	-0.26	0.019877	-1.97	0.048635	0.050493	0.9992	0/-
HLF	-0.31	1.11E-05	-0.05	0.650276	1.97	0.048663	0.050525	0.9992	-/0
RP11-219G17.4	0.22	0.00211	-0.05	0.694727	-1.97	0.048686	0.050557	0.9992	+/0
CLEC5A	0.03	0.705734	-0.24	0.036815	-1.97	0.048718	0.050592	0.9992	0/-
BAALC-AS1	-0.33	2.29E-06	-0.08	0.503626	1.97	0.048768	0.050656	0.9992	-/0
RP1-290I10.5	-0.18	0.010116	0.08	0.477223	1.97	0.048777	0.050666	0.9992	-/0
RPRM	-0.14	0.049485	0.13	0.27393	1.97	0.048843	0.050762	0.9992	-/0
RBM17P4	-0.01	0.84547	-0.28	0.014736	-1.97	0.049053	0.051008	0.9992	0/-

RNU6-1238P	0.17	0.01686	-0.09	0.411237	-1.97	0.049204	0.051168	0.9992	+/0
RP11-227L6.1	-0.03	0.703701	0.24	0.037452	1.97	0.04924	0.0512	0.9992	0/+
RNASE13	0.15	0.037417	-0.12	0.309487	-1.97	0.049244	0.0512	0.9992	+/0
GNE	0.03	0.703417	-0.24	0.037523	-1.97	0.049293	0.051242	0.9992	0/-
RP1-154K9.2	-0.20	0.005774	0.07	0.55829	1.97	0.049337	0.051293	0.9992	-/0
TEPP	-0.17	0.014992	0.09	0.427874	1.97	0.049377	0.051335	0.9992	-/0
TCTEX1D2	0.19	0.009834	-0.08	0.485001	-1.97	0.049389	0.051361	0.9992	+/0
ACTBP13	0.16	0.02251	-0.10	0.374486	-1.97	0.049399	0.051373	0.9992	+/0
VPS13B	-0.15	0.041054	0.12	0.299085	1.96	0.049439	0.051428	0.9992	-/0
SH3BP5	-0.15	0.041574	0.12	0.298084	1.96	0.049552	0.051533	0.9992	-/0
VPS29	0.20	0.005119	-0.06	0.576728	-1.96	0.049572	0.051562	0.9992	+/0
RAB29	-0.18	0.011284	0.08	0.46796	1.96	0.049665	0.051652	0.9992	-/0
ADAMTS16	0.00	0.998926	-0.26	0.020827	-1.96	0.049693	0.051674	0.9992	0/-
VN1R2	0.04	0.591454	0.30	0.008222	1.96	0.049725	0.051709	0.9992	0/+
TMEM245	0.00	0.99671	-0.26	0.020969	-1.96	0.049776	0.05178	0.9992	0/-
CYP3A5	-0.02	0.759601	-0.28	0.012418	-1.96	0.049784	0.051793	0.9992	0/-
CAMK2N2	0.35	8.75E-07	0.09	0.42113	-1.96	0.049868	0.051886	0.9992	+/0
EDRF1	0.14	0.058488	0.38	0.00052	1.96	0.04992	0.05193	0.9992	0/+
RP11-135F9.3	0.14	0.04839	-0.12	0.281608	-1.96	0.049954	0.051975	0.9992	+/0

Table S7: Significant EZH2 Protein Correlations from TCGA HGOSC

Gene	Partial Correlation	Pvalue	FDR
LPCAT1	0.47	2.49E-05	0.062823
SMIM20	-0.50	2.62E-05	0.062823
KDM5C.1	0.48	3.40E-05	0.062823
NCAPH	0.45	7.37E-05	0.083308
ATG101	0.51	7.58E-05	0.083308
**PARP2**	0.46	8.86E-05	0.083308
KAT7	0.44	9.19E-05	0.083308
PAIP1	0.44	0.000101572	0.083308
PAIP1.1	0.44	0.000120316	0.087715
KIF11	0.44	0.000130711	0.087715
RRM2	0.44	0.000150209	0.092399
KDM5C	0.44	0.00018615	0.094073
RPS6KB1	0.44	0.000193688	0.094073
GSTK1	-0.43	0.00019383	0.094073
TACSTD2	-0.42	0.000203907	0.094073
CEP55	0.48	0.000269697	0.115483
TPX2	0.41	0.000291684	0.115483
TBC1D13	0.41	0.000297248	0.115483
HLA-DRA	-0.41	0.000338722	0.116632
PSMC3	0.41	0.000352429	0.116632
LRRC40	0.41	0.000361469	0.116632
SCYL3	-0.43	0.000375996	0.116632
LYPLA2	-0.41	0.000388753	0.116632
CD300A	0.50	0.000464644	0.116632
ATL2	0.40	0.000477661	0.116632
CD74	-0.40	0.000482915	0.116632
DENND2D	-0.40	0.000484443	0.116632
PDK3	0.40	0.000507116	0.116632
BAZ1A	0.41	0.000516167	0.116632
PPL	-0.40	0.000527614	0.116632
CD74.1	-0.40	0.000543704	0.116632
AKR1C4	0.40	0.000563936	0.116632
ACOT2	0.40	0.000571885	0.116632
ACOT1	0.40	0.000572502	0.116632
RAB21	0.40	0.000583381	0.116632
NHP2L1	-0.39	0.000598184	0.116632
HGNC:9982	-0.41	0.000600407	0.116632
EHMT1	0.39	0.000648016	0.119289
AKR1C2	0.39	0.000669741	0.119289
EED	0.39	0.000671748	0.119289
AKR1C1	0.39	0.000682544	0.119289
MED13L	0.51	0.000709194	0.119289

CD68	0.44	0.000711046	0.119289
RGCC	-0.41	0.000739595	0.121321
MSLN	-0.39	0.000770683	0.123672
AP5B1	0.49	0.000815366	0.128059
AKR1C3	0.38	0.000876782	0.134158
PRIM1	0.41	0.00089055	0.134158
TXNDC9	0.39	0.000928328	0.137052

Table S8: Genes with significant (P<0.05) protein differential correlation (r2) with EZH2 in platinum resistant (PR) versus platinum sensitive (PS) HGOSC (TCGA)

Gene	PS_r2	PS_pVal	PR_r2	PR_pVal	zScoreDiff	pValDiff	Classes
DAAM2	0.47	0.004962	-0.86	1.56E-05	-5.51358	3.52E-08	+/-
**HDAC7**	0.07	0.669026	-0.86	1.42E-06	-4.57607	4.74E-06	0/-
RUNX1	0.35	0.035039	-0.73	0.000355	-4.28239	1.85E-05	+/-
USP19	0.04	0.824708	-0.82	9.95E-06	-4.06362	4.83E-05	0/-
G3BP2	-0.28	0.079706	0.70	0.000546	3.963376	7.39E-05	0/+
MAP3K11	-0.21	0.309254	0.89	0.000258	3.945732	7.96E-05	0/+
EIF3I	0.17	0.290293	-0.75	0.000146	-3.9007	9.59E-05	0/-
TSC1	0.11	0.51532	-0.79	8.35E-05	-3.87195	0.000108	0/-
GTF2E2	-0.20	0.212869	0.72	0.0003	3.828588	0.000129	0/+
DOCK5	-0.27	0.108536	0.69	0.000835	3.751068	0.000176	0/+
GGA2	-0.29	0.079676	0.66	0.001616	3.668389	0.000244	0/+
CTTNBP2	0.70	0.000541	-0.52	0.065775	-3.65415	0.000258	+/0
RGN	-0.25	0.170962	0.78	0.001062	3.644927	0.000267	0/+
VILL	0.03	0.8825	-0.81	7.97E-05	-3.60227	0.000315	0/-
URI1	-0.24	0.141842	0.68	0.001366	3.571057	0.000356	0/+
CHI3L1	0.19	0.246137	-0.69	0.000698	-3.56457	0.000364	0/-
C18orf25.1	0.07	0.688406	-0.76	0.000152	-3.55964	0.000371	0/-
LETM2	-0.62	0.003545	0.60	0.031365	3.545208	0.000392	-/+
AIM1	-0.15	0.341897	0.71	0.000491	3.538244	0.000403	0/+
C18orf25	0.08	0.628452	-0.75	0.000207	-3.52793	0.000419	0/-
C10orf76	0.28	0.075646	-0.62	0.003815	-3.45038	0.00056	0/-
PSPC1	-0.21	0.196314	0.66	0.001492	3.437492	0.000587	0/+
NSFL1C	-0.11	0.481969	0.71	0.000429	3.434072	0.000595	0/+
MEX3A	0.56	0.002792	-0.48	0.052725	-3.40892	0.000652	+/0
RPS6KA4	0.35	0.03475	-0.58	0.009021	-3.39237	0.000693	+/-
CHD1L	0.23	0.160572	-0.64	0.00224	-3.38891	0.000702	0/-
IPO11	0.52	0.000649	-0.39	0.085533	-3.37182	0.000747	+/0
THOC5	0.24	0.133488	-0.63	0.003142	-3.34907	0.000811	0/-
CA12	0.31	0.104571	-0.69	0.004651	-3.32496	0.000884	0/-
GTF3C1	0.09	0.588603	0.79	4.04E-05	3.316382	0.000912	0/+
USP40	0.00	0.983957	0.77	0.000166	3.299972	0.000967	0/+
KRT13	0.09	0.589305	-0.71	0.000508	-3.29942	0.000969	0/-
SYNE2.1	0.32	0.049825	-0.61	0.009707	-3.27912	0.001041	+/-
WDR44	0.23	0.153589	-0.62	0.003828	-3.25123	0.001149	0/-
PPP2R5E	-0.16	0.320003	0.66	0.001681	3.237852	0.001204	0/+
ANKRD17	-0.20	0.221676	0.63	0.002746	3.229945	0.001238	0/+
ANLN	0.35	0.027736	-0.54	0.017736	-3.21937	0.001285	+/-
SLC38A10	0.13	0.465404	-0.70	0.001132	-3.21077	0.001324	0/-
SLC35F6	-0.12	0.48686	0.68	0.000934	3.210011	0.001327	0/+
KRT15	0.05	0.757516	-0.71	0.000453	-3.20029	0.001373	0/-
CCNL2	-0.33	0.120306	0.71	0.006474	3.192575	0.00141	0/+
PMS2	-0.29	0.101412	0.64	0.007531	3.186	0.001443	0/+
WIPI2	0.19	0.240324	-0.63	0.002958	-3.18206	0.001462	0/-

RYBP	0.36	0.064248	-0.60	0.011246	-3.17575	0.001494	0/-
PA2G4	0.32	0.042228	-0.53	0.016472	-3.15176	0.001623	+/-
PRPF38A	0.24	0.129194	-0.58	0.006985	-3.12599	0.001772	0/-
INTS9	-0.33	0.036166	0.51	0.020479	3.117169	0.001826	-/+
RNF126	-0.10	0.651695	0.75	0.000516	3.106901	0.001891	0/+
KRT75	0.41	0.008018	-0.44	0.053536	-3.10301	0.001916	+/0
IFRD2	0.46	0.016522	-0.54	0.038012	-3.10235	0.00192	+/-
ZFC3H1	0.17	0.334495	-0.65	0.002407	-3.09043	0.001999	0/-
QRSL1	0.33	0.036303	-0.50	0.02329	-3.0735	0.002116	+/-
USP14	-0.32	0.04101	0.51	0.021394	3.073104	0.002118	-/+
TTF2	0.25	0.244425	-0.67	0.00353	-3.05889	0.002222	0/-
CA8	0.48	0.0025	-0.37	0.111603	-3.05474	0.002253	+/0
STRN3	0.06	0.70121	0.74	0.000177	3.049207	0.002294	0/+
ELMSAN1	0.25	0.125154	-0.58	0.009506	-3.04635	0.002316	0/-
KIF1B	0.38	0.014454	-0.45	0.045556	-3.04279	0.002344	+/-
USMG5	0.14	0.391529	-0.63	0.002666	-3.03323	0.00242	0/-
PPP4R1	-0.13	0.410988	0.64	0.002596	3.02123	0.002517	0/+
TMEM199	-0.09	0.635308	0.71	0.001288	3.018774	0.002538	0/+
PDCD4	0.04	0.805644	-0.68	0.00092	-2.98113	0.002872	0/-
DSCR3	0.05	0.753638	-0.68	0.000947	-2.97783	0.002903	0/-
GABARAP	-0.21	0.220247	0.60	0.006602	2.977663	0.002905	0/+
C16orf62	-0.06	0.734429	-0.73	0.00027	-2.96937	0.002984	0/-
NLE1	0.15	0.340075	-0.61	0.004068	-2.96768	0.003001	0/-
CEP170B	0.14	0.438002	-0.70	0.00369	-2.95605	0.003116	0/-
DAB2IP	-0.29	0.124008	0.55	0.012001	2.95102	0.003167	0/+
NT5DC1	0.29	0.064989	-0.51	0.022419	-2.94435	0.003236	0/-
PNO1	0.14	0.417297	-0.62	0.003274	-2.93607	0.003324	0/-
GSDMB	0.15	0.423736	-0.70	0.003749	-2.93365	0.00335	0/-
KRT80	0.19	0.232133	-0.58	0.007309	-2.93048	0.003384	0/-
ZNF592	0.42	0.082541	-0.58	0.018511	-2.93035	0.003386	0/-
RNF31	0.15	0.350653	-0.61	0.004506	-2.92649	0.003428	0/-
RAB27B	0.31	0.065816	-0.52	0.023678	-2.92514	0.003443	0/-
TMEM57	-0.28	0.088444	0.62	0.018247	2.923883	0.003457	0/+
CCDC53	-0.47	0.003086	0.38	0.12461	2.921682	0.003481	-/0
TARDBP	-0.23	0.152324	0.55	0.011662	2.92051	0.003495	0/+
STRN3.1	0.10	0.537041	0.74	0.000177	2.919033	0.003511	0/+
ST14	0.44	0.004937	-0.37	0.110128	-2.91291	0.003581	+/0
SLC35A2	-0.20	0.269147	0.62	0.006337	2.908095	0.003636	0/+
WIPF2	-0.02	0.923285	-0.70	0.000553	-2.89571	0.003783	0/-
SMG8	0.06	0.724507	0.72	0.000332	2.89537	0.003787	0/+
PEX5	0.23	0.162521	-0.55	0.011675	-2.89419	0.003801	0/-
VEZF1	0.34	0.029274	-0.45	0.045244	-2.89176	0.003831	+/-
BCHE	0.27	0.217128	-0.64	0.007522	-2.88995	0.003853	0/-
CLMN	0.32	0.046685	-0.47	0.034325	-2.88091	0.003965	+/-
ERGIC2	0.19	0.236549	-0.57	0.008468	-2.87963	0.003981	0/-
TSEN54	-0.08	0.687145	0.72	0.001545	2.874629	0.004045	0/+
C3orf17	-0.05	0.778582	0.70	0.001757	2.87143	0.004086	0/+

KRT24	-0.16	0.442912	-0.85	0.000258	-2.87004	0.004104	0/-
PDRG1	0.02	0.915944	0.70	0.000669	2.868797	0.00412	0/+
CEP250	0.30	0.104889	-0.53	0.019088	-2.86793	0.004132	0/-
KRT20	0.05	0.776622	-0.66	0.001524	-2.86726	0.00414	0/-
RARS2	0.36	0.025684	-0.44	0.054761	-2.86109	0.004222	+/0
ARHGEF12	0.40	0.010128	-0.39	0.091187	-2.8504	0.004366	+/0
FCF1	-0.14	0.467518	0.67	0.004191	2.843849	0.004457	0/+
UBE4A	0.27	0.091955	-0.50	0.023978	-2.83073	0.004644	0/-
WDR5B	0.32	0.05704	-0.48	0.036119	-2.82173	0.004777	0/-
KIF1B.1	0.34	0.030137	-0.44	0.05407	-2.81967	0.004807	+/0
SLC25A23	-0.57	0.00073	0.26	0.312691	2.811941	0.004924	-/0
MORC3	0.49	0.001406	-0.28	0.232317	-2.80085	0.005097	+/0
WRAP53	0.41	0.021578	-0.41	0.083282	-2.77493	0.005521	+/0
RSRC1	-0.30	0.116332	0.55	0.023541	2.772944	0.005555	0/+
AP2M1	0.39	0.013324	-0.38	0.096876	-2.76981	0.005609	+/0
WWC3	-0.13	0.466993	0.65	0.005153	2.769379	0.005616	0/+
KIAA1524	0.08	0.644313	0.75	0.000595	2.767279	0.005653	0/+
LETM1	-0.35	0.027662	0.42	0.065711	2.765512	0.005683	-/0
ADIPOQ	0.05	0.766086	-0.69	0.002257	-2.76344	0.00572	0/-
ARHGEF12.1	0.40	0.010045	-0.37	0.113135	-2.76324	0.005723	+/0
KRT14	0.10	0.527763	-0.61	0.004591	-2.75215	0.00592	0/-
MAP3K21	-0.13	0.516588	0.76	0.00667	2.751861	0.005926	0/+
NME3	-0.20	0.209744	-0.77	8.15E-05	-2.74996	0.00596	0/-
QPRT	0.01	0.941188	0.67	0.001128	2.748877	0.00598	0/+
BLOC1S4	0.19	0.24132	-0.54	0.013356	-2.73145	0.006306	0/-
FGD4	0.22	0.180108	-0.52	0.018188	-2.7272	0.006388	0/-
DMXL1	-0.19	0.244424	0.54	0.013286	2.725814	0.006414	0/+
NEMF	0.00	0.995909	0.66	0.001418	2.725668	0.006417	0/+
TARBP1	0.05	0.77376	-0.65	0.002433	-2.7255	0.00642	0/-
SPAST	0.04	0.831627	0.70	0.000617	2.719113	0.006546	0/+
TRPV2	0.14	0.51487	-0.67	0.004321	-2.70834	0.006762	0/-
ZNF618	-0.07	0.690428	0.62	0.00322	2.705672	0.006817	0/+
RFX5	-0.17	0.300562	-0.75	0.000187	-2.70384	0.006854	0/-
BRPF1	0.36	0.028214	-0.42	0.076628	-2.7007	0.006919	+/0
MYO5A	0.27	0.092763	0.79	3.66E-05	2.698136	0.006973	0/+
HIST1H4G	0.11	0.496657	-0.59	0.006074	-2.69697	0.006997	0/-
CSTF3	0.01	0.959158	0.66	0.001458	2.69257	0.00709	0/+
SRPX	0.37	0.033064	-0.43	0.074545	-2.69152	0.007113	+/0
NELFCD	0.03	0.843657	-0.64	0.002459	-2.68745	0.0072	0/-
KRT19	-0.01	0.933204	-0.66	0.001392	-2.68691	0.007212	0/-
WDR6	0.23	0.247824	-0.61	0.015213	-2.68434	0.007267	0/-
SNRPF	-0.06	0.724924	0.63	0.003643	2.676742	0.007434	0/+
FAH	0.16	0.323569	-0.55	0.01149	-2.67516	0.007469	0/-
ATP6V1E1	-0.43	0.005102	0.31	0.186779	2.673537	0.007506	-/0
ECT2	0.09	0.611004	0.71	0.000494	2.673254	0.007512	0/+
NT5C	0.21	0.192383	-0.51	0.020195	-2.67206	0.007539	0/-
NFIC	0.09	0.581536	-0.60	0.005425	-2.65851	0.007849	0/-

EHBP1	-0.15	0.361082	-0.74	0.000255	-2.65548	0.00792	0/-
KRT18	0.34	0.029697	-0.40	0.083668	-2.65516	0.007927	+/0
HDAC5	-0.18	0.379018	0.62	0.008554	2.653371	0.007969	0/+
SMAD3	0.20	0.206415	-0.51	0.020266	-2.64833	0.008089	0/-
ACBD5	0.06	0.731804	0.69	0.000846	2.645979	0.008145	0/+
TTC5	0.23	0.19044	0.78	5.60E-05	2.632837	0.008467	0/+
KRT77	0.24	0.145194	-0.50	0.029703	-2.62929	0.008556	0/-
IRF2BP2	0.38	0.016164	-0.36	0.123202	-2.62901	0.008563	+/0
SNX4	0.26	0.102947	-0.46	0.039579	-2.62646	0.008628	0/-
MAPRE1	-0.33	0.034762	0.40	0.082867	2.622809	0.008721	-/0
RPLP1	-0.15	0.345465	-0.73	0.00028	-2.6226	0.008726	0/-
ELANE	0.40	0.009634	-0.33	0.15959	-2.622	0.008741	+/0
C11orf58	-0.03	0.847887	0.63	0.003123	2.617262	0.008864	0/+
ITPA	-0.41	0.008023	0.31	0.176232	2.613151	0.008971	-/0
SUDS3	0.23	0.171982	-0.49	0.027208	-2.60533	0.009178	0/-
OS9	0.12	0.452575	-0.57	0.009433	-2.60408	0.009212	0/-
BAG4	-0.18	0.367258	0.59	0.010725	2.591722	0.00955	0/+
CARD9	0.07	0.676023	-0.64	0.005577	-2.59006	0.009596	0/-
KIAA1211L	-0.23	0.234013	-0.83	0.000383	-2.58596	0.009711	0/-
FBXL8	-0.23	0.223199	0.58	0.023298	2.585902	0.009712	0/+
TUBB1	0.35	0.026436	-0.37	0.105943	-2.58551	0.009724	+/0
YTHDF1	0.04	0.809027	0.66	0.001479	2.582404	0.009811	0/+
TSTD1	0.11	0.504444	-0.57	0.008902	-2.57507	0.010022	0/-
RETSAT	0.10	0.552631	-0.58	0.00778	-2.57487	0.010028	0/-
ATP6V0D1	0.06	0.718877	0.67	0.00121	2.570563	0.010153	0/+
IRF2BP2.1	0.35	0.026496	-0.37	0.110245	-2.56843	0.010216	+/0
TCF7L2	0.52	0.01589	-0.33	0.199886	-2.56711	0.010255	+/0
WASF3	-0.24	0.227707	0.58	0.023992	2.561496	0.010422	0/+
XP_003120211	0.43	0.049668	-0.47	0.088786	-2.55039	0.01076	+/0
RAP2A	-0.18	0.278323	0.52	0.020098	2.550177	0.010767	0/+
KHDRBS3	0.16	0.311009	-0.52	0.018285	-2.54136	0.011042	0/-
USP10	0.11	0.504846	0.69	0.000721	2.535949	0.011214	0/+
TCF25	-0.19	0.236869	0.50	0.024892	2.534332	0.011266	0/+
MME	0.43	0.014852	-0.33	0.178275	-2.52913	0.011435	+/0
RAP2C	-0.20	0.215592	0.49	0.028396	2.52078	0.011709	0/+
VPS37A	0.01	0.959811	0.64	0.003066	2.515496	0.011887	0/+
RHBDF2	0.15	0.356391	-0.54	0.017078	-2.51449	0.01192	0/-
KRT8	0.32	0.042911	-0.38	0.097145	-2.50917	0.012102	+/0
PATL1	0.01	0.927065	0.64	0.002625	2.508314	0.012131	0/+
SRCAP	-0.03	0.848106	0.63	0.004184	2.506704	0.012186	0/+
CEP131	0.10	0.558483	-0.58	0.009267	-2.50574	0.01222	0/-
MRPL2	0.04	0.812201	-0.60	0.005105	-2.50142	0.01237	0/-
C15orf52	0.15	0.471042	-0.65	0.01228	-2.50047	0.012403	0/-
NR3C1	0.13	0.435002	-0.54	0.013632	-2.48989	0.012778	0/-
HLA-F	-0.45	0.027853	0.44	0.131958	2.487982	0.012847	-/0
PSMD6	0.32	0.042374	0.79	3.85E-05	2.487421	0.012867	+/+
NP_001070991	0.14	0.425344	-0.54	0.013916	-2.48722	0.012875	0/-

HNRNPC.1	0.01	0.935269	0.63	0.002898	2.486278	0.012909	0/+
NEBL.1	-0.42	0.006915	0.27	0.24441	2.485112	0.012951	-/0
MRPL3	0.01	0.948163	-0.63	0.004201	-2.47724	0.01324	0/-
C14orf1	0.11	0.500522	-0.57	0.013235	-2.47718	0.013243	0/-
HNRNPC	0.02	0.879176	0.64	0.002604	2.476842	0.013255	0/+
GJA1	0.40	0.022751	-0.34	0.160814	-2.47677	0.013258	+/0
C2CD2	0.40	0.029196	-0.39	0.133875	-2.47407	0.013358	+/0
GNAI1	0.15	0.356094	-0.52	0.019356	-2.47209	0.013433	0/-
DHX15	-0.22	0.181473	0.46	0.03893	2.465824	0.01367	0/+
NCKIPSD	0.13	0.449814	-0.55	0.014927	-2.46499	0.013702	0/-
LRRC57	0.11	0.509531	-0.55	0.012709	-2.46012	0.013889	0/-
PTRH1	-0.26	0.142957	0.45	0.048598	2.460009	0.013893	0/+
RPL34	-0.39	0.012213	0.30	0.204127	2.459667	0.013907	-/0
NAGLU	0.35	0.026304	-0.34	0.143797	-2.45604	0.014048	+/0
CRIP1	-0.17	0.284599	-0.71	0.00041	-2.45528	0.014077	0/-
WDFY4	-0.07	0.712851	-0.76	0.003855	-2.45511	0.014084	0/-
NKIRAS2	0.38	0.024904	-0.35	0.155192	-2.44723	0.014396	+/0
UBE2B	-0.06	0.727447	0.62	0.008151	2.44498	0.014486	0/+
RBM25	0.03	0.874887	0.63	0.002903	2.443285	0.014554	0/+
SMARCD2	0.07	0.646147	-0.56	0.00951	-2.43842	0.014752	0/-
MICAL1	-0.13	0.423041	-0.69	0.000791	-2.4367	0.014822	0/-
CNPY2	0.50	0.001163	-0.17	0.478455	-2.432	0.015016	+/0
UHRF1BP1L	0.09	0.67942	0.76	0.001787	2.431728	0.015027	0/+
CD99L2	0.61	0.000659	-0.14	0.610751	-2.43063	0.015073	+/0
POLR3D	-0.23	0.182013	0.47	0.042597	2.430566	0.015075	0/+
ADAR.1	-0.11	0.507528	0.54	0.01438	2.422673	0.015407	0/+
TAF1	-0.09	0.623757	-0.75	0.003142	-2.42208	0.015432	0/-
SMARCA2	0.04	0.792114	0.64	0.002561	2.419235	0.015553	0/+
DDX52	-0.18	0.269062	0.48	0.030713	2.418939	0.015566	0/+
ARIH2	0.39	0.013204	-0.29	0.215742	-2.41695	0.015651	+/0
RAB4B	-0.13	0.447466	-0.69	0.000985	-2.41102	0.015908	0/-
TOR3A	0.40	0.032828	-0.33	0.1645	-2.41037	0.015936	+/0
CHURC1-FNTB	0.48	0.001684	-0.18	0.447749	-2.4097	0.015966	+/0
SIGLEC1	-0.08	0.613809	0.56	0.011719	2.408538	0.016017	0/+
MAPK7	-0.50	0.023936	0.34	0.215852	2.403234	0.016251	-/0
BSG	0.21	0.188958	-0.45	0.044799	-2.40267	0.016276	0/-
SLU7	0.16	0.323664	-0.52	0.027454	-2.39925	0.016429	0/-
YTHDC1	-0.20	0.220872	0.46	0.039637	2.396303	0.016561	0/+
ADAR	-0.11	0.513998	0.53	0.015384	2.39545	0.0166	0/+
PARVB	-0.04	0.800661	-0.63	0.002866	-2.39388	0.016671	0/-
C14orf169	-0.13	0.416902	-0.68	0.000916	-2.39217	0.016749	0/-
SMAP1	-0.07	0.658226	0.56	0.01091	2.38661	0.017005	0/+
PSMC4	-0.06	0.735184	0.57	0.009123	2.383732	0.017138	0/+
RASSF7	0.13	0.556132	-0.64	0.01353	-2.38238	0.017201	0/-
ABCC1	0.06	0.701293	-0.56	0.010076	-2.37848	0.017384	0/-
SAP130	-0.05	0.781583	0.59	0.008225	2.376283	0.017488	0/+
CEP164	0.03	0.876681	0.84	0.009635	2.374887	0.017554	0/+

MTHFD1	0.43	0.00573	-0.23	0.323703	-2.37446	0.017575	+/0
DDC	0.50	0.012331	-0.28	0.297669	-2.37421	0.017586	+/0
CENPH	-0.14	0.518906	0.71	0.020656	2.37182	0.017701	0/+
ERMP1	0.15	0.355154	-0.50	0.026235	-2.37158	0.017712	0/-
HAX1	0.11	0.532012	-0.54	0.014961	-2.37108	0.017736	0/-
PRMT2	-0.11	0.561304	-0.81	0.00464	-2.37105	0.017738	0/-
LAMTOR4	-0.33	0.044624	0.38	0.127013	2.365275	0.018017	-/0
CEP55	0.64	0.000188	-0.05	0.866232	-2.36455	0.018052	+/0
MRPL32	0.10	0.537357	-0.54	0.016053	-2.36401	0.018078	0/-
LRRC8C	-0.11	0.519114	-0.71	0.001902	-2.35991	0.018279	0/-
UIMC1	0.03	0.859097	-0.61	0.006619	-2.35614	0.018466	0/-
SNRPD2	-0.16	0.327509	0.48	0.030249	2.353425	0.018601	0/+
CDCP1	0.11	0.502756	-0.52	0.018509	-2.33484	0.019552	0/-
RFWD2	0.49	0.011934	-0.25	0.323717	-2.33238	0.019681	+/0
TCEB3	-0.14	0.380567	0.49	0.02772	2.326063	0.020015	0/+
MYO5C	0.00	0.983269	-0.59	0.005749	-2.32236	0.020213	0/-
CXCL17	-0.28	0.240537	-0.86	0.000671	-2.32123	0.020274	0/-
ESYT1	0.44	0.004862	-0.21	0.382729	-2.31154	0.020803	+/0
TMF1	-0.12	0.449242	-0.66	0.001393	-2.31114	0.020825	0/-
QTRTD1	0.32	0.045056	-0.33	0.152694	-2.30455	0.021192	+/0
DOK2	-0.12	0.544642	-0.73	0.002754	-2.30238	0.021314	0/-
ACOX3	-0.61	4.21E-05	-0.02	0.931838	2.297748	0.021576	-/0
TBC1D9	0.20	0.251545	-0.64	0.046753	-2.29719	0.021608	0/-
XP_003119131	0.08	0.636059	0.64	0.002625	2.295392	0.021711	0/+
MTX3	0.32	0.048299	-0.39	0.136582	-2.29343	0.021823	+/0
RPP30	-0.19	0.245554	0.45	0.048302	2.289414	0.022055	0/+
TRAPPC8	-0.07	0.688084	-0.63	0.003122	-2.28669	0.022214	0/-
MTX3.1	0.33	0.045357	-0.37	0.14828	-2.28646	0.022227	+/0
AKAP9	-0.15	0.347122	-0.68	0.001046	-2.28496	0.022315	0/-
TPI1	0.09	0.587718	-0.52	0.018164	-2.27997	0.022609	0/-
LYPLA1	-0.11	0.508457	0.51	0.022614	2.274171	0.022956	0/+
FAM118B	0.52	0.003563	-0.16	0.525214	-2.27202	0.023085	+/0
CDK9	0.03	0.851051	0.60	0.004985	2.271298	0.023129	0/+
PPT1	0.15	0.345131	0.67	0.001094	2.270838	0.023157	0/+
KRT16	-0.05	0.747371	-0.62	0.003875	-2.26933	0.023248	0/-
GLA	0.09	0.58381	-0.52	0.019147	-2.26589	0.023458	0/-
SRSF3	-0.01	0.974498	0.58	0.007712	2.264134	0.023566	0/+
LACTB	-0.49	0.001435	0.13	0.585537	2.261866	0.023706	-/0
EMC3	-0.07	0.680214	0.53	0.015518	2.258401	0.023921	0/+
WDR12	-0.11	0.48322	0.50	0.025334	2.258312	0.023926	0/+
RAI1	-0.13	0.491206	-0.69	0.001141	-2.25636	0.024048	0/-
RPS9	0.49	0.00137	-0.13	0.597691	-2.25543	0.024107	+/0
USO1	-0.39	0.012659	0.24	0.302148	2.254493	0.024165	-/0
WASH1	-0.39	0.012747	0.24	0.302088	2.253168	0.024249	-/0
CNOT1	0.36	0.020643	-0.27	0.248317	-2.25272	0.024277	+/0
PHF14	0.07	0.664228	-0.53	0.015394	-2.25245	0.024294	0/-
MPI	0.00	0.982456	-0.58	0.007878	-2.25212	0.024315	0/-

TMEM147	0.21	0.335196	-0.58	0.039012	-2.24989	0.024456	0/-
TACC3	0.10	0.525321	0.64	0.002149	2.24605	0.024701	0/+
MESDC2	-0.09	0.568606	-0.63	0.002631	-2.24067	0.025047	0/-
GHDC	0.07	0.659037	-0.53	0.017389	-2.23784	0.025231	0/-
MRPL44	0.06	0.711074	-0.53	0.015399	-2.23759	0.025248	0/-
RAPGEF6	-0.35	0.035266	0.33	0.190806	2.232706	0.025568	-/0
ATP5I	-0.04	0.829474	-0.60	0.005405	-2.23216	0.025604	0/-
COMMD10	-0.35	0.031223	0.29	0.222428	2.229502	0.025781	-/0
ZFR	0.04	0.78866	0.60	0.004966	2.227617	0.025906	0/+
STX16	-0.32	0.044681	0.31	0.184586	2.225952	0.026017	-/0
STX16.1	-0.32	0.044846	0.31	0.184765	2.224603	0.026108	-/0
MICAL3	0.13	0.454235	-0.50	0.030118	-2.22273	0.026234	0/-
DNAJC2	0.10	0.554634	0.63	0.002718	2.220019	0.026417	0/+
SIAE	0.04	0.794266	-0.54	0.013678	-2.21428	0.026809	0/-
ESYT1.1	0.41	0.008237	-0.21	0.38028	-2.21354	0.026861	+/0
HMGB2	0.19	0.2437	0.69	0.000855	2.211845	0.026977	0/+
FOXJ3	-0.15	0.422299	0.51	0.031686	2.211696	0.026988	0/+
FARP2	0.13	0.446648	0.66	0.002052	2.210431	0.027075	0/+
CKMT1A	0.09	0.588083	-0.51	0.022489	-2.20919	0.027161	0/-
PTRH2	0.13	0.411513	-0.47	0.035712	-2.20709	0.027308	0/-
NRBP1	0.34	0.032912	-0.29	0.220879	-2.20638	0.027357	+/0
CCNT1	0.05	0.77652	-0.57	0.014502	-2.2061	0.027377	0/-
ZNF703	-0.11	0.524099	0.51	0.02639	2.205168	0.027442	0/+
FNTB	0.46	0.002927	-0.15	0.529431	-2.20465	0.027479	+/0
PRIM1	0.50	0.003923	-0.14	0.562546	-2.2035	0.027559	+/0
GUF1	0.00	0.988432	0.60	0.00797	2.202369	0.027639	0/+
GOLGA2	-0.32	0.042347	0.30	0.197572	2.200912	0.027742	-/0
FAHD2B	0.01	0.953722	0.57	0.008105	2.198926	0.027883	0/+
TRPS1	-0.52	0.00396	0.14	0.585799	2.198524	0.027912	-/0
PSMB7	0.25	0.121306	0.72	0.000392	2.196703	0.028042	0/+
HACL1	-0.11	0.534484	0.52	0.025566	2.196504	0.028056	0/+
NIP7	0.15	0.352771	-0.46	0.043723	-2.19541	0.028134	0/-
IGFBP4	0.37	0.045934	-0.34	0.196907	-2.19146	0.028418	+/0
NDUFAF7	-0.48	0.001838	0.12	0.610102	2.189971	0.028526	-/0
DCLK2	-0.06	0.738211	0.55	0.017506	2.18855	0.02863	0/+
SIGIRR	0.04	0.798747	-0.54	0.014723	-2.18758	0.0287	0/-
DDX6	0.13	0.428754	0.65	0.002042	2.187496	0.028706	0/+
WDFY1	-0.02	0.916867	0.55	0.011315	2.186786	0.028758	0/+
KIF20B	0.39	0.038459	-0.41	0.18729	-2.18523	0.028872	+/0
CENPV	0.18	0.276965	0.67	0.00114	2.179747	0.029276	0/+
AHDC1	-0.18	0.265369	-0.68	0.001081	-2.17895	0.029335	0/-
PEAK1	0.08	0.680213	-0.59	0.020994	-2.17348	0.029744	0/-
NMD3	0.01	0.958518	0.57	0.008923	2.172994	0.029781	0/+
RREB1	0.49	0.001247	-0.10	0.684475	-2.17226	0.029836	+/0
FAM175A	0.05	0.773739	-0.59	0.017029	-2.17118	0.029917	0/-
KRT1	0.32	0.046536	-0.30	0.201463	-2.16898	0.030084	+/0
LDHD	-0.03	0.893075	0.75	0.019799	2.167198	0.03022	0/+

CRKL	-0.13	0.426019	0.47	0.038476	2.166615	0.030264	0/+
LRSAM1	-0.10	0.559075	0.49	0.026825	2.163273	0.03052	0/+
CENPC	0.09	0.588793	0.62	0.003678	2.162392	0.030588	0/+
SARNP	-0.13	0.40955	0.46	0.040738	2.162282	0.030596	0/+
CARD11	0.06	0.807855	-0.64	0.013082	-2.16093	0.030701	0/-
UBE2Z	0.09	0.591419	-0.50	0.02584	-2.15979	0.030789	0/-
LYSMD2	-0.10	0.580058	-0.68	0.004011	-2.15881	0.030865	0/-
DNTTIP1	0.59	0.00021	0.02	0.933555	-2.15695	0.03101	+/0
ARPC1A	-0.06	0.734399	0.52	0.018718	2.156943	0.03101	0/+
THTPA	0.08	0.646782	0.62	0.004295	2.155794	0.0311	0/+
FTL	0.03	0.860086	0.58	0.007545	2.15468	0.031187	0/+
GSTM2	-0.01	0.937446	0.55	0.012029	2.153199	0.031303	0/+
PNN	0.03	0.851325	0.58	0.00759	2.146654	0.031821	0/+
C10orf32	0.11	0.487691	-0.47	0.034545	-2.1435	0.032073	0/-
SOX17	-0.10	0.550795	0.49	0.029903	2.143286	0.03209	0/+
CLN3	-0.11	0.536701	0.55	0.032921	2.141265	0.032253	0/+
AKAP17A	-0.13	0.417357	0.46	0.042353	2.140675	0.0323	0/+
LAMP1	-0.09	0.586731	0.49	0.02767	2.140309	0.03233	0/+
LRRC20	0.11	0.537445	0.68	0.004941	2.135951	0.032683	0/+
TMEM209	-0.14	0.40404	0.46	0.04529	2.135836	0.032693	0/+
CFDP1	-0.13	0.436191	0.47	0.04163	2.132917	0.032932	0/+
BRAF	0.26	0.116431	0.72	0.00055	2.130682	0.033115	0/+
RGS19	0.14	0.473691	-0.50	0.034039	-2.12961	0.033204	0/-
CRABP1	0.07	0.647337	-0.50	0.02487	-2.12844	0.0333	0/-
ACTR3	-0.36	0.023377	0.24	0.299778	2.127979	0.033339	-/0
LRRC16A	-0.17	0.304275	-0.66	0.00157	-2.12713	0.033409	0/-
MCEE	-0.47	0.002868	0.14	0.568037	2.125801	0.03352	-/0
ANKHD1-EIF4EBI	0.15	0.342216	0.65	0.001858	2.12467	0.033614	0/+
NEK9	0.31	0.049985	-0.29	0.213097	-2.12466	0.033615	+/0
U2SURP	-0.06	0.722467	0.51	0.021269	2.123697	0.033695	0/+
SMC6	0.06	0.729027	0.59	0.006096	2.123595	0.033704	0/+
RASA2	-0.11	0.523431	-0.63	0.002644	-2.12336	0.033723	0/-
TTC26	0.05	0.786824	0.61	0.006972	2.123039	0.033751	0/+
SUPV3L1	0.13	0.407731	-0.45	0.045863	-2.12133	0.033894	0/-
BRD1	-0.06	0.749155	0.55	0.021839	2.117195	0.034243	0/+
FTSJ1	-0.11	0.53912	0.52	0.032272	2.113753	0.034536	0/+
SIRT6	-0.03	0.874398	0.81	0.028181	2.11341	0.034566	0/+
SULT1A3	0.05	0.775635	-0.52	0.019629	-2.11107	0.034766	0/-
PIK3R2	-0.31	0.049194	-0.74	0.000215	-2.10926	0.034923	-/-
CD3EAP	0.09	0.581544	-0.48	0.030733	-2.10845	0.034992	0/-
NLRX1	-0.24	0.138071	-0.70	0.000653	-2.10318	0.03545	0/-
TMA16	0.09	0.573372	-0.48	0.031415	-2.10265	0.035497	0/-
PARD6B	-0.12	0.460814	0.46	0.042287	2.100113	0.035719	0/+
ATP5J2-PTCD1	0.10	0.558505	-0.48	0.033864	-2.09368	0.036288	0/-
HIST2H2AA3	0.02	0.892995	0.56	0.010052	2.090404	0.036582	0/+
HPR	0.35	0.027889	-0.24	0.301927	-2.0848	0.037088	+/0
MOB4	-0.03	0.859999	0.52	0.017716	2.084066	0.037154	0/+

FBXO22	0.12	0.477798	-0.46	0.042386	-2.0838	0.037178	0/-
CCDC88A	0.03	0.859911	0.60	0.011392	2.083685	0.037189	0/+
PYROXD1	-0.20	0.264544	-0.69	0.001183	-2.08228	0.037317	0/-
GTF3C3	-0.01	0.950787	0.54	0.014642	2.08163	0.037376	0/+
PSMA6	0.14	0.386501	0.64	0.002573	2.081426	0.037395	0/+
BAG5	-0.11	0.48355	0.46	0.042542	2.077306	0.037773	0/+
HNRNPR	0.02	0.912504	0.56	0.01097	2.077039	0.037798	0/+
SLC44A2	-0.35	0.025372	0.23	0.319752	2.075106	0.037977	-/0
INCENP	0.41	0.031769	-0.24	0.342366	-2.07448	0.038034	+/0
FAHD2A	0.00	0.977592	0.54	0.014167	2.073416	0.038134	0/+
PITPNC1	0.01	0.963896	-0.60	0.014306	-2.07205	0.038261	0/-
GIGYF1	0.10	0.569029	-0.48	0.035522	-2.06947	0.038502	0/-
DLG5	0.15	0.454445	-0.51	0.045194	-2.06649	0.038782	0/-
WIBG	0.35	0.027117	-0.24	0.316277	-2.06615	0.038814	+/0
DUSP23	0.03	0.868563	-0.52	0.018391	-2.06581	0.038846	0/-
SEMA4B	0.43	0.008064	-0.21	0.431819	-2.06488	0.038934	+/0
HAGH	-0.12	0.456476	0.45	0.047139	2.064755	0.038946	0/+
GTF2E1	0.07	0.677653	0.59	0.006538	2.064129	0.039005	0/+
TPP1	-0.10	0.522857	0.46	0.040254	2.062673	0.039144	0/+
RRP12	-0.02	0.906859	-0.55	0.011451	-2.05969	0.039428	0/-
MRE11A	-0.38	0.015861	0.20	0.393945	2.059329	0.039463	-/0
GEMIN2	0.07	0.674468	0.63	0.006959	2.059195	0.039476	0/+
PDCL	0.14	0.45718	0.69	0.003231	2.058149	0.039576	0/+
CD2BP2	-0.02	0.919906	0.53	0.016946	2.056237	0.03976	0/+
GRIPAP1	-0.11	0.499571	0.45	0.043842	2.052233	0.040147	0/+
SLC4A1AP	-0.10	0.545215	0.46	0.039543	2.050036	0.040361	0/+
ACPP	-0.05	0.805361	0.52	0.017905	2.050001	0.040364	0/+
FKBP15	-0.12	0.465807	-0.62	0.003773	-2.0496	0.040404	0/-
PTBP1	-0.41	0.008952	0.17	0.486302	2.047954	0.040565	-/0
PTBP1.1	-0.41	0.008948	0.17	0.486438	2.047872	0.040573	-/0
C6orf132	-0.10	0.562832	-0.63	0.005035	-2.04704	0.040654	0/-
AIF1L	-0.05	0.73891	0.50	0.025801	2.046748	0.040683	0/+
HIST1H2AA	-0.05	0.759102	0.50	0.024835	2.044792	0.040875	0/+
STAT4	0.00	0.997452	-0.91	0.034369	-2.0417	0.041182	0/-
TANC2	-0.05	0.802554	-0.66	0.007977	-2.04028	0.041323	0/-
SMAD1	0.11	0.480791	-0.45	0.047624	-2.03886	0.041464	0/-
PPP1R9A	-0.01	0.93921	-0.54	0.013209	-2.03748	0.041602	0/-
HNRNPH1	0.11	0.515807	0.61	0.004623	2.03543	0.041808	0/+
PMM1	-0.10	0.602846	0.51	0.031019	2.034183	0.041933	0/+
TSG101	0.08	0.619122	0.59	0.006211	2.03394	0.041958	0/+
HNRNPR.1	0.04	0.793404	0.56	0.009699	2.030416	0.042314	0/+
SETD1B	0.06	0.753808	-0.53	0.020869	-2.02918	0.04244	0/-
RBM33	0.07	0.668405	0.58	0.007181	2.028974	0.042461	0/+
FUBP1	0.07	0.648219	0.58	0.006837	2.028148	0.042545	0/+
HN1L	-0.34	0.030469	0.23	0.323317	2.027786	0.042582	-/0
TUBG2	-0.07	0.675443	0.48	0.031413	2.026954	0.042667	0/+
FLOT1	0.10	0.537259	-0.46	0.043015	-2.02658	0.042706	0/-

PROM2	-0.41	0.029118	0.23	0.368893	2.024193	0.04295	-/0
VPS54	0.13	0.520283	0.69	0.004205	2.022619	0.043112	0/+
RAB35	0.05	0.767481	-0.50	0.02616	-2.02108	0.043272	0/-
HIST2H2AB	-0.02	0.883312	0.51	0.020436	2.020728	0.043308	0/+
POLR2I	-0.02	0.917145	-0.54	0.013297	-2.01991	0.043393	0/-
NRCAM	0.06	0.708263	-0.50	0.029446	-2.01987	0.043397	0/-
POLA2	0.41	0.019181	-0.27	0.344552	-2.01637	0.043762	+/0
TK2	-0.10	0.583373	0.48	0.037778	2.014398	0.043968	0/+
HOXB4	-0.03	0.872945	-0.70	0.01743	-2.01238	0.04418	0/-
MYH15	-0.09	0.675151	-0.65	0.003346	-2.01044	0.044385	0/-
C2orf47	-0.02	0.918144	0.52	0.019702	2.008268	0.044615	0/+
CLIC1	-0.24	0.135997	-0.68	0.000925	-2.00763	0.044683	0/-
DDX19A	-0.07	0.662633	0.48	0.034185	2.007309	0.044717	0/+
ATXN2L	0.08	0.638198	0.58	0.007177	2.005798	0.044878	0/+
MAP2K5	-0.24	0.336439	-0.86	0.006595	-2.00494	0.04497	0/-
GINS4	0.03	0.875186	0.58	0.013695	2.003585	0.045115	0/+
CUL1	0.06	0.732776	0.57	0.009212	2.001884	0.045297	0/+
MAP1A	0.01	0.935169	-0.52	0.018501	-2.0003	0.045468	0/-
METTL3	0.07	0.675872	0.57	0.00816	1.996118	0.045921	0/+
SMAD5	-0.06	0.734383	-0.56	0.009642	-1.98905	0.046696	0/-
SCYL1	0.32	0.042318	-0.24	0.301859	-1.98824	0.046785	+/0
RNASE3	0.41	0.032889	-0.28	0.335506	-1.98659	0.046968	+/0
SH2D4A	-0.10	0.54659	0.45	0.047207	1.984973	0.047147	0/+
SLC2A5	0.44	0.025407	-0.17	0.474155	-1.98362	0.047299	+/0
CHMP2A	0.02	0.920564	-0.51	0.022031	-1.96959	0.048886	0/-
PBK	0.23	0.161781	0.70	0.001809	1.969271	0.048922	0/+
PSMG3	-0.06	0.723997	-0.57	0.010642	-1.96617	0.049279	0/-
XPA	0.42	0.038289	-0.22	0.387093	-1.9645	0.049472	+/0
PANK4	0.02	0.88018	0.54	0.014751	1.960854	0.049896	0/+

Figure S1. Tumor EZH2 expression by Clinical Factors

a) Optimal (O) vs Suboptimal (S) Debulking

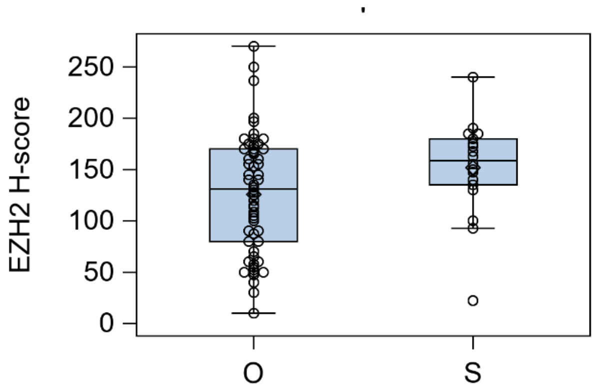

b) Stage

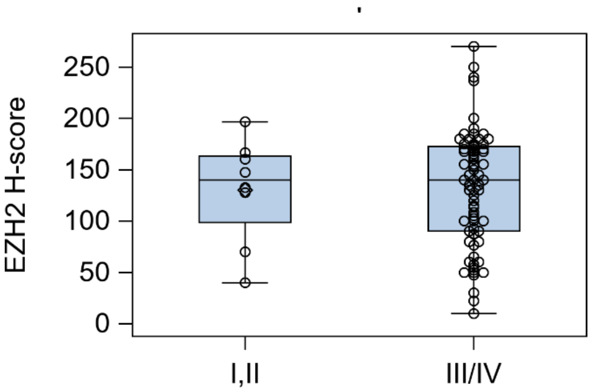

c) Response to therapy

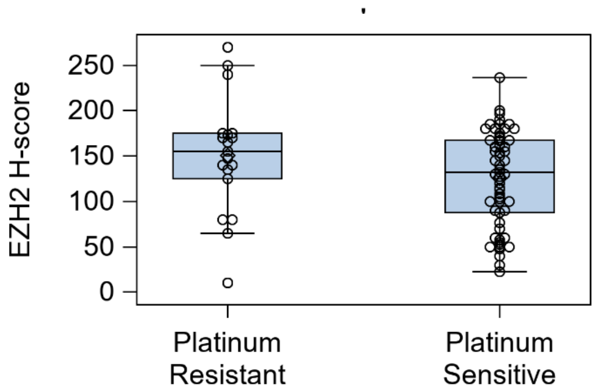

Figure S2. Digital Hscore for EZH2 expression by tissue type

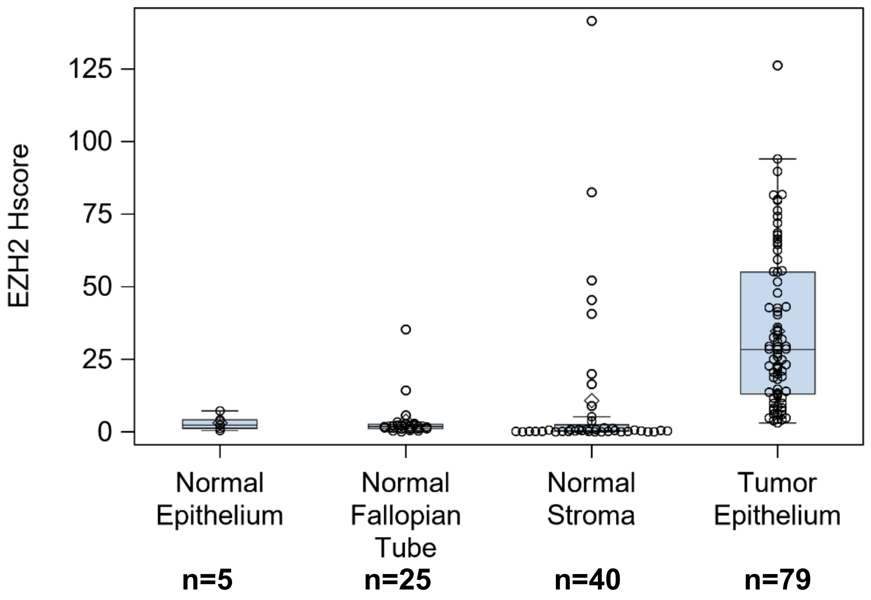

Figure S3. Volcano plot for co-expression of 31, 237 genes with EZH2 in TCGA HGSOC (n=336)

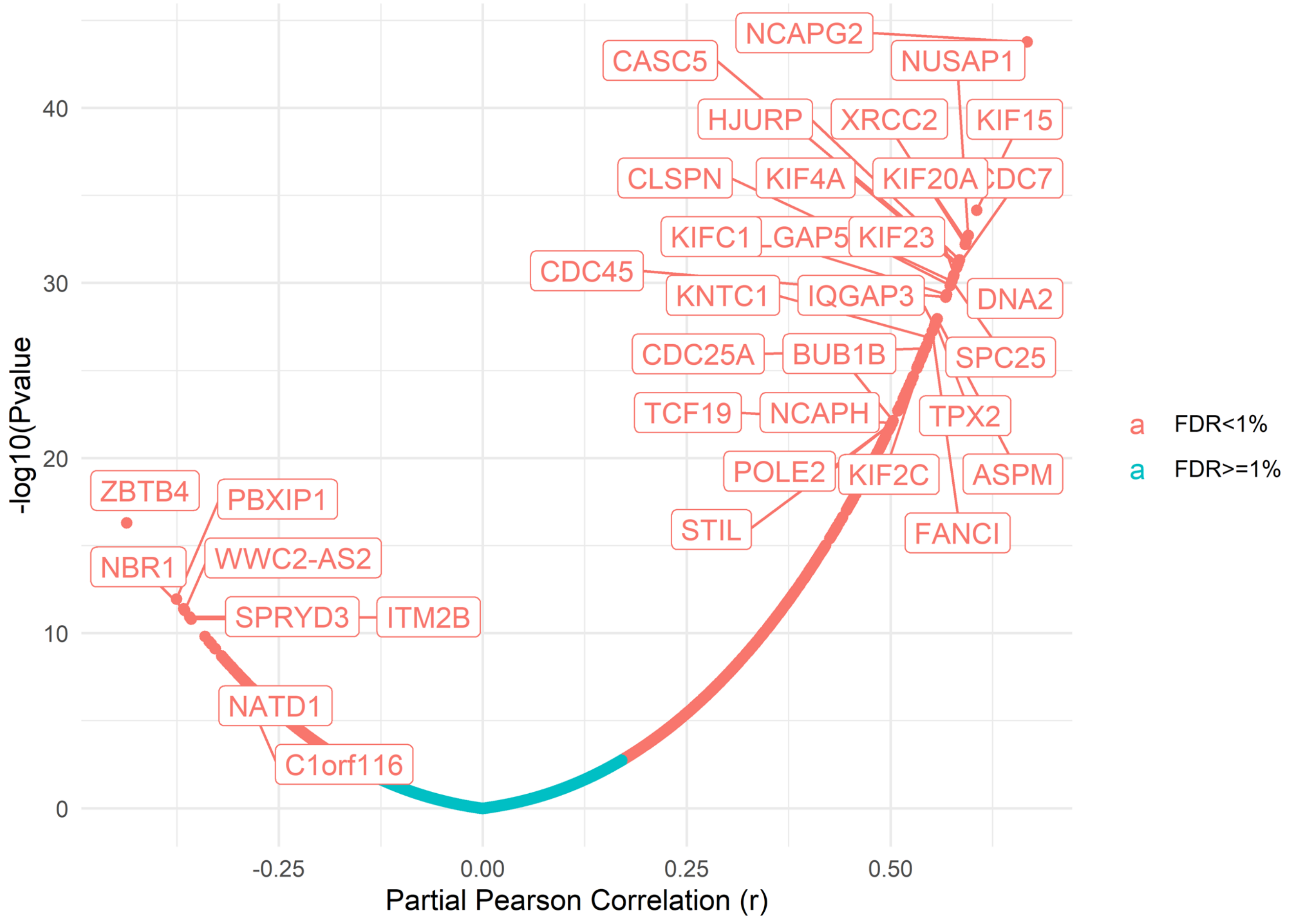

Figure S4. Planar filtered network of genes correlated with EZH2 exclusively in platinum resistant HGSOC

Expression of gene pairs with a significant gain in correlation ($p < 0.05$, n=559) in platinum resistant compared to platinum sensitive tumor samples (i.e. correlation exclusive to resistant samples only) was used to construct a planar filtered network (PFN) shown here. The node fill color represents the (z-score) difference in correlation with negative correlations with EZH2 colored as blue and positive correlations as red. Genes that were identified as modifiers of EZH2 prognosis in logistic regression modeling have diamond nodes with the border color representing whether the gene reduced (green) the prognostic value of EZH2 (i.e. toward the null) or increased the prognostic value (red). Modules were identified at $p < 0.05$ in this network. Network analysis was performed using R package MEGENA and the plot was made using Cytoscape (version 3.8.2).

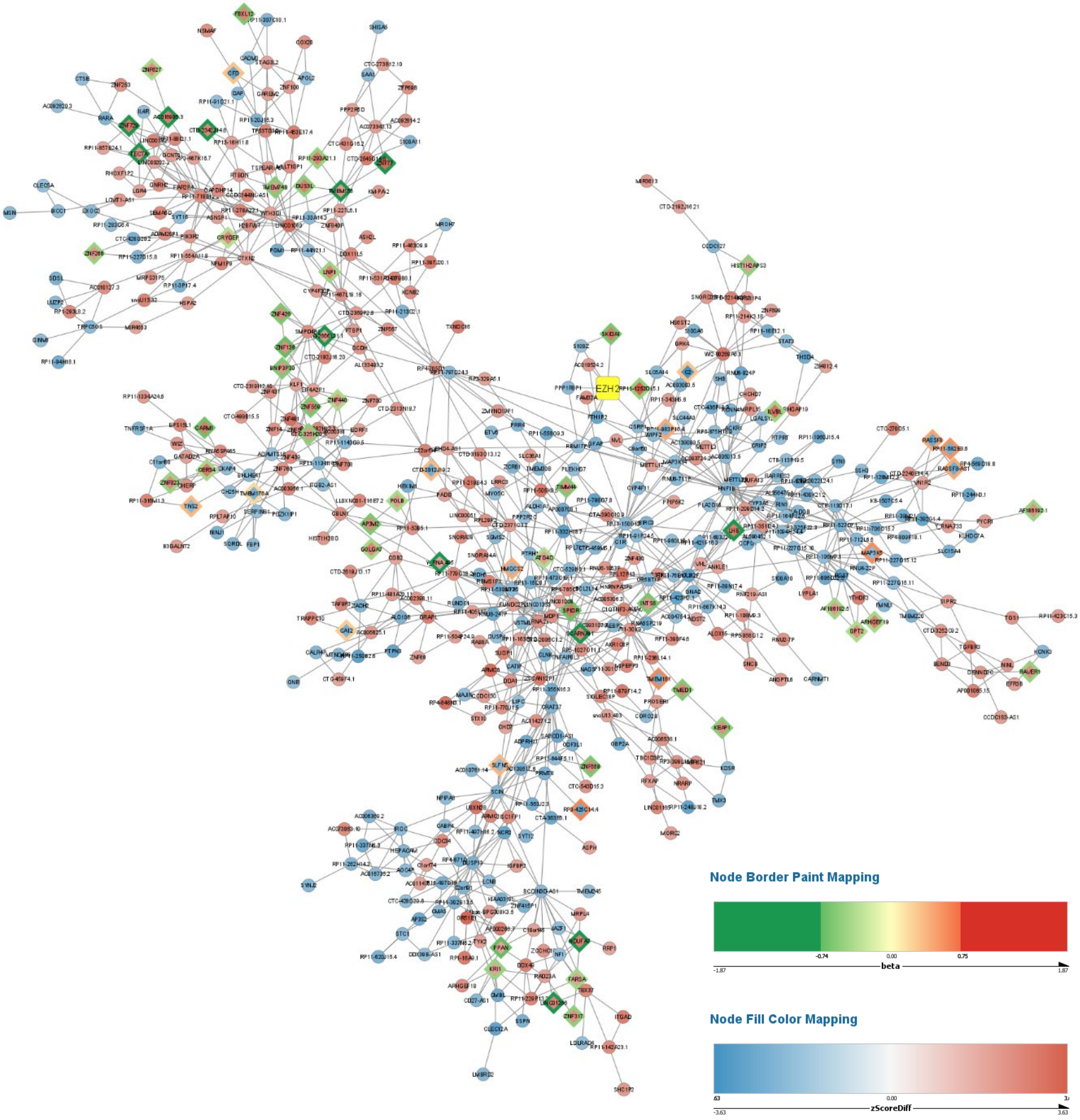

Figure S5. Planar filtered network of genes correlated with EZH2 exclusively in platinum sensitive HGSOC

Expression of gene pairs with a significant loss of correlation ($p < 0.05$, n=440) in platinum resistant compared to platinum sensitive tumor samples (i.e. correlation exclusive to sensitive samples only) was used to construct a planar filtered network shown here. The node fill color represents the (z-score) difference in correlation with negative correlations with EZH2 colored as blue and positive correlations as red. Genes that were identified as modifiers of EZH2 prognosis in logistic regression modeling have diamond nodes with the border color representing whether the gene reduced (green) the prognostic value of EZH2 (i.e. toward the null) or increased the prognostic value (red). Modules were identified at $p < 0.05$ in this network. Network analysis was performed using R package MEGENA and the plot was made using Cytoscape (version 3.8.2).

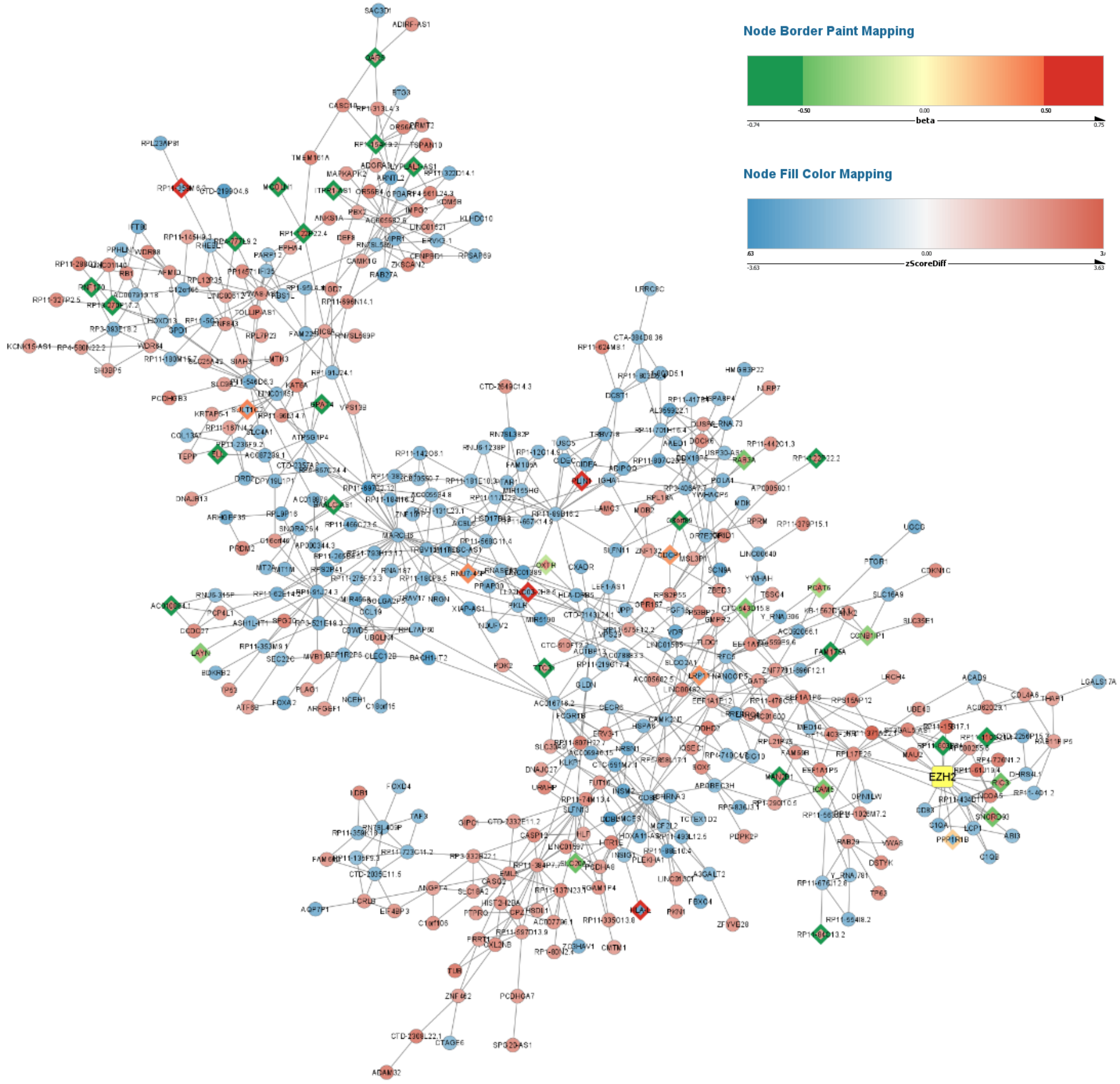

Figure S6. Gene set enrichment analysis for co-expression networks using Molecular Signatures Database

Results are presented for Molecular Signatures Database (v7.3) GSEA analysis for sensitive (n=440) and resistant (n=559) network gene sets. GSEA was run separately for mRNA and protein expression networks and tested for enrichment in Gene Ontology Biological Processes. Results for the top five, non-redundant terms are presented. The number of genes in the EZH2 networks are labeled for each GO Biological Process.

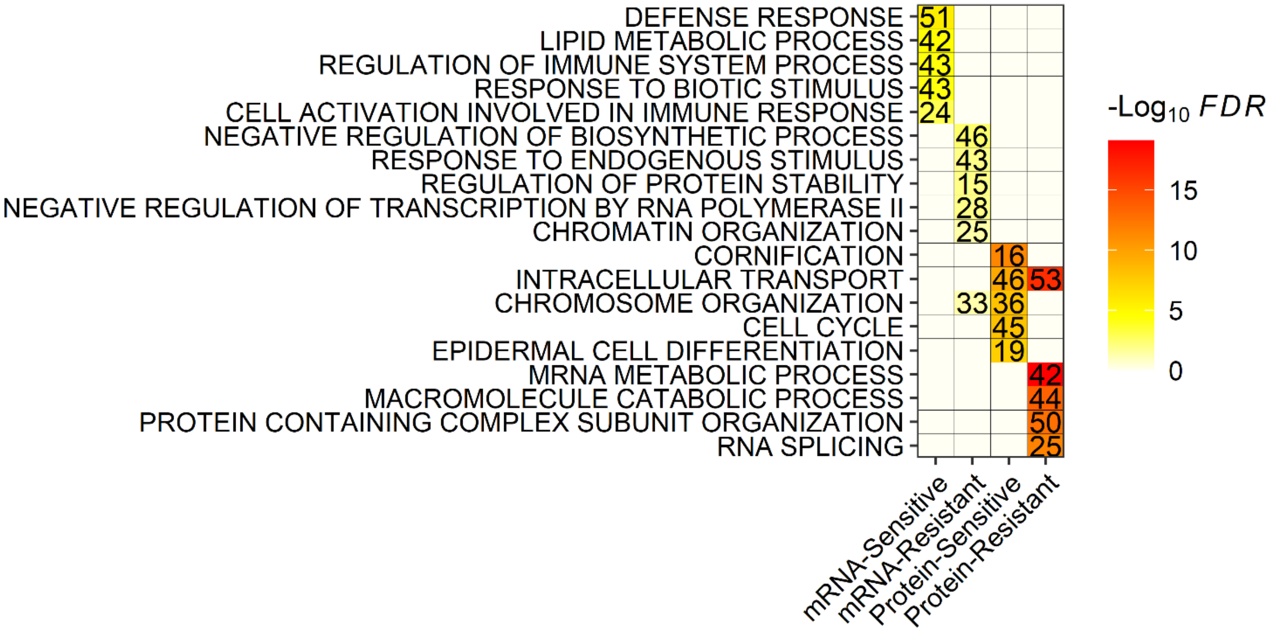

Supplement: Supplementary file 1 — Additional file 1: Table S1: Characteristics of Study Participants by BRCA Status. Table S2: Cross frequency table for architectural growth patterns. Table S3. Associations between Molecular and Clinicopathologic Factors in TCGA. Table S4: List of Top 1% of Modifier Genes and their Interaction Term OR and Pvalues. Table S5: Significant EZH2 mRNA Correlations from TCGA HGOSC. Table S6: Genes with significant (P < 0.05) mRNA differential correlation (r2) with EZH2 in platinum resistant (PR) versus platinum sensitive (PS) HGOSC (TCGA). Table S7: Significant EZH2 Protein Correlations from TCGA HGOSC. Table S8: Genes with significant (P < 0.05) protein differential correlation (r2) with EZH2 in platinum resistant (PR) versus platinum sensitive (PS) HGOSC (TCGA). Fig. S1. Tumor EZH2 expression by Clinical Factors. Fig. S2. Digital Hscore for EZH2 expression by tissue type. Fig. S3. Volcano plot for co-expression of 31, 237 genes with EZH2 in TCGA HGSOC (n = 336). Fig. S4. Planar filtered network of genes correlated with EZH2 exclusively in platinum resistant HGSOC. Fig. S5. Planar filtered network of genes correlated with EZH2 exclusively in platinum sensitive HGSOC. Fig. S6. Gene set enrichment analysis for co-expression networks using Molecular Signatures Database. [file 12885_2021_8413_MOESM1_ESM.pdf]
